# A revision of the spider genus *Selenops* Latreille, 1819 (Arachnida, Araneae, Selenopidae) in North America, Central America and the Caribbean

**DOI:** 10.3897/zookeys.105.724

**Published:** 2011-06-14

**Authors:** Sarah C. Crews

**Affiliations:** Berkeley City College, 2050 Center Street, Berkeley, CA, 94704

**Keywords:** new species, island, endemism, systematics, Mesoamerica, *Selenops*, Selenopidae

## Abstract

The spider genus *Selenops* Latreille, 1819 occurs in both the Old World and New World tropics and subtropics and contains nearly half of the species in the family Selenopidae Simon, 1897. In this paper the members of the genus *Selenops* found in North America, Central America, and on islands of the Caribbean are revised, excluding Cuban endemics. No taxonomic changes are currently made to the species from the southwestern United States. In total, 21 new species are described, including *Selenops arikok* **sp. n.**, *Selenops chamela* **sp. n.**, *Selenops amona* **sp. n.**, *Selenops baweka* **sp. n.**, *Selenops bocacanadensis* **sp. n.**, *Selenops enriquillo* **sp. n**, *Selenops ixchel* **sp. n.**, *Selenops huetocatl* **sp. n.**, *Selenops kalinago* **sp. n.**, *Selenops oviedo* **sp. n.**, *Selenops morro* **sp. n.**, *Selenops denia* **sp. n.**, *Selenops duan* **sp. n.**, *Selenops malinalxochitl* **sp. n.**, *Selenops oricuajo* **sp. n.**, *Selenops petenajtoy* **sp. n.**, *Selenops guerrero* **sp. n.**, *Selenops makimaki* **sp. n.**, *Selenops souliga* **sp. n.**, *Selenops wilmotorum* **sp. n.**, and *Selenops wilsoni* **sp. n.** Six species names were synonymized: *Selenops lunatus* Muma, 1953 **syn. n. =** *Selenops candidus* Muma, 1953; *Selenops tehuacanus* Muma 1953 **syn. n.**, *Selenops galapagoensis* Banks, 1902 **syn. n.** and *Selenops vagabundus* Kraus, 1955 **syn. n.** = *Selenops mexicanus* Keyserling, 1880; *Selenops santibanezi* Valdez-Mondragón, 2010 **syn. n.** = *Selenops nigromaculatus* Keyserling, 1880; and *Selenops salvadoranus* Chamberlin, 1925 **syn. n.** = *Selenops bifurcatus* Banks, 1909. Lectotypes are designated for the following three species: *Selenops marginalis* F. O. Pickard-Cambridge, 1900 (♂), *Selenops morosus* Banks, 1898 (♂), and *Selenops mexicanus* Keyserling, 1880 (♀). The female neotype is designated for *Selenops aissus* Walckenaer, 1837. The males of *Selenops bani* Alayón-García, 1992 and *Selenops marcanoi* Alayón-García, 1992 are described for the first time, and the females of *Selenops phaselus* Muma, 1953 and *Selenops geraldinae* Corronca, 1996 are described for the first time. Almost all species are redescribed, barring Cuban endemics and a few species recently described. New illustrations are provided, including those of the internal female copulatory organs, many of which are illustrated for the first time. A key to species is also provided as are new distributional records.

## Introduction

Spiders of the family Selenopidae Simon, 1897, also known as wall crab spiders or flatties, have a cosmotropical distribution. They are exceptional in that both their running and striking speeds place them amongst the world's fastest animals (Crews et al. 2008), and they are extremely dorsoventrally flattened (Figs 183–187). Prior to this work, the family currently comprises around 196 species in 5 genera (Corronca 1998; Platnick 2010): *Anyphops* Benoit, 1968, *Garcorops* Corronca, 2003, *Hovops* Benoit, 1968, *Siamspinops* Dankittipakul & Corronca, 2009, and *Selenops* Latreille, 1819. The first three genera are distributed in Africa and Madagascar, and *Siamspinops* in Southeast Asia. Five new genera are currently being described for a total of nine genera (Crews and Harvey 2011). *Selenops* is distributed throughout the tropics and subtropics worldwide, and the majority of the species in the family are placed in this genus. Walckenaer (1837) first recognized three groups based on characteristics of the chelicerae, labium and leg lengths. These characters were not substantiated by Simon (1880), who attempted to divide the family into Old and New World groups based on eye size. (Corronca (1998, 2002) provided diagnoses of the genera, however there are many variations. The monophyly of none of the genera has been formally tested.

In the Western Hemisphere, F. O. Pickard-Cambridge (1900) first distinguished species using eye size and position along with genitalic characters. (Petrunkevitch (1925, 1930) divided these species into groups and described new species based on leg proportions. Muma (1953) established six subgeneric species groups for species occurring from North and Central America and the Caribbean based on leg lengths, eye size and position, and genitalic characters. Current authors (Alayón-García 1992, 2001, 2003, 2005; Valdez-Mondragón 2007, 2010) still use these characters and groupings despite variation and species that do not fit into any group.

While selenopids are quite common in certain areas and are relatively large, museum collections of this family are often depauperate (Corronca 1998). As with most spiders, many species of the family are known from either a single male or single female specimen, known only from the type, many of which were collected over 100 years ago. Sexes can be difficult to match and even if two sexes were collected at the same place at the same time, this does not guarantee they are the same species, as many species have overlapping distributions. I have found that the best ways to determine if certain males and females are the same species are collecting both sexes from multiple localities, and use of independent data, such as DNA or other genetic data.

Revisionary and descriptive work for *Anyphops*, *Hovops* and *Garcorops* has been done by Lawrence (1940), Benoit (1968), and more recently by (Corronca (1996, 2000, 2003, 2005). The genus *Selenops* has been revised primarily by region, with the African species examined by (Lawrence (1940, 1942), Benoit (1968), and (Corronca (2001, 2002), and the South American species revised by (Corronca (1996, 1998). In the West Indies and Central America, the last major revisionary work was conducted by Muma (1953), while (Alayón-García (1992, 2001, 2003, 2005) revised the species in Cuba, and has completed other regional descriptions. Only a few species have been described from México (Valdez-Mondragón 2007, 2010) since Muma's (1953) work. The Asian and Australian species are concurrently being revised and described (Crews and Harvey 2011), but are still in need of collection, as only a handful are in museums and have been described (Simon 1901; Gravely 1931; Reimoser 1934; Tikader 1969).

Here a taxonomic revision of *Selenops* from Greater North America, including México, Central America and the Caribbean islands, except for Cuba, is presented. Twenty one new species are described, and the first descriptions of 2 males and 2 females from previously described species are included. Also, 6 species names are synonymized and comprehensive locality records are provided allowing a more thorough examination of distributional data. Additionally, valuable natural history data, and new illustrations, including those of internal female copulatory organs, which have largely been omitted in past revisions and descriptions, are provided. Many of the types are very old and in poor condition (e.g. - coloration and markings have faded, leaving the specimens an artificial orangey color; many of the hairs have worn off; in some cases abdominal setal tufts (Figs 179–180, 192) may actually be present on newer specimens). For many specimens, all the legs, or parts of the legs, have been disarticulated; in such cases, previous author's leg formulae have been used. Descriptions of these along with descriptions of newer specimens for comparison, as well as photographs of live specimens (Figs 177–195) have been provided. Information on the locality of types is presented. Finally, data independent of morphology (Crews and Gillespie 2010) have been used to place juvenile specimens for the purpose of (1) providing locality records, (2) examining of the validity of hypothesized species groups (Muma 1953) and associated morphological characters, and (3) examining monophyly and relationships of the genera that are based on morphological characters. A key to species is provided. The fossil species *Selenops beynai* Schawaller (1984) is not included in the key, but images from high resolution CT-scanning are included (Figs 111–118).

Despite the spiders being large and conspicuous, and subject to recent descriptive and revisionary work (Alayón-García 1992, 2001, 2003, 2005; Corronca 1996, 1998; Valdez-Mondragón 2007, 2010), several previously undescribed species have been found. There is no doubt that there are still more species to be discovered from the region. Even though there are some widespread species, there are also several species with very small ranges, and many are found from the same area. Of note are the Greater Antillean Islands of Cuba and Hispaniola, as well as mainland México, all of which are extremely species-rich.

## Material and methods

In addition to the examination of museum specimens, several new specimens were collected from over 200 localities primarily in the Caribbean, Southwestern United States, México and Central America. In total, over 1600 specimens were examined. Voucher numbers for new collections were placed in the vial and can be found in Crews and Gillespie (2010). These numbers begin with sel_, for *Selenops*.

Measurements were taken using a Leica dissecting microscope and ocular micrometer, or from photographs taken with the Microptics system. The photos were taken with a Minitool scale in place, and then the images were imported into Adobe Illustrator where they could then be measured. All measurements are in millimeters (mm).

The width and length of the carapace and sternum were measured at the widest and longest parts. Legs were measured along the dorsal side. Eye dimensions were measured across the lens, and eye group measurements and inter-eye distances were measured from the farthest distances.

Genitalic illustrations were made from photographs as well as actual specimens. The left palpus of the male was removed from each specimen and photographed using the Microptics imaging system. If only a right palpus was available, it was photographed and the photo was inverted horizontally for consistency using Adobe Photoshop. In most descriptive work and revisionary studies, the left palpus has been figured. However, Muma (1953) figured the right palpus. In this study, to adhere to convention and not frustrate workers that have already removed the left palpus, the left palpus is illustrated. The internal copulatory organs were not figured by Muma (1953), but I have found that the internal genitalic characters are important for identification and for assessing evolutionary relationships, thus they are figured here. The internal copulatory organs of many female *Selenops* species are highly sclerotized and full of strong tissues. Attempts to remove the tissues with forceps could potentially cause damage to the underlying structures. A digestive enzyme mixture of pancreatin and borax following Álvarez-Padilla and Hormiga (2008) was used to clean the copulatory organs. In many species, the epigynal plate folds inward and is directed anteriorly. This has been referred to as a ‘uterus externus' by Dankittipakul and Corronca (2009), though this seems to be an incorrect usage (see Snodgrass 1952 and Foelix 1996), and in this work it is referred to as the posterodorsal fold of the epigynum. This invagination covers the spermathecae or other parts of the internal copulatory organs. Thus, it can be difficult to distinguish characters. Where possible, half of the posterodorsal fold was removed, though sometimes it was removed in its entirety to allow the viewing of the internal copulatory organs. Great care was taken to not disturb the internal genitalic structures during removal. The left palpus is figured in ventral and lateral view, and the female copulatory organs are figured in ventral and dorsal view. The figures of the internal copulatory organs have been drawn so that the left half depicts the internal copulatory organs with the posterodorsal fold removed and the right half depicts the internal copulatory organs with the posterodorsal fold present. Terms used in reference to genitalic structures follow those of Corronca (1998) unless otherwise noted. Muma (1953) referred to the apex of the abdomen of some specimens as having a festooned appearance, caused by the dark coloration of the posterior lateral margins of the abdomen. The same terminology is used here. Descriptions were written primarily using CSRIO DELTA v1.04 for windows (Dallwitz et al. 2000), and edited manually. In many cases the types are quite old and in poor condition. In the instances where newer specimens were available, these are described alongside the type specimens. Immature specimens were identified to species using molecular genetic data (Crews and Gillespie 2010).

Abbreviations used in the text are as follows:

Eyes

AERanterior eye row

ALEanterior lateral eyes

AMEanterior median eyes

PERposterior eye row

PLEposterior lateral eyes

PMEposterior median eyes

Legs and palps

FMfemur

Mtmetatarsus

Ptpatella

Titibia

Tatarsus

Rright

Lleft

Leg spination

apapical

ddorsal

prprolateral

rtretrolateral

vventral

Male copulatory organs

MAmedian apophysis

RTAretrolateral tibial apophysis

Cconductor

Repositories

AMNHAmerican Museum of Natural History, New York, USA (N. Platnick, L. Sorkin)

BMNHBritish Museum of Natural History, London, England (J. Beccaloni)

CASCalifornia Academy of Sciences, San Francisco, CA, USA (C. Griswold, D. Ubick)

CNANColleción Nacional de Arácnidos, Instituto de Biología, Universidad Nacional Autónoma de México, Distrito Federal, México (O. Francke)

EMEEssig Museum of Entomology, University of California, Berkeley, Berkeley, CA, USA (R. Gillespie, C. Barr)

FSUFlorida State University Arthropod Collection, Tallahassee, FL, USA (G.B. Edwards)

IESInstituto de Ecología y Sistemática, Academia de Ciencias de Cuba, Havana, Cuba

IJNHMInstitute of Jamaica, Natural History Museum, Kingston, Jamaica (E. Morrison)

MCZMuseum of Comparative Zoology, Harvard University, Cambridge, MA, USA (L. Leibensperger)

MNHNMuséum National d'Histoire Naturelle, Paris, France (C. Rollard)

MNHNSDMuseo Nacional de Historia Natural, Santo Domingo, Dominican Republic (S. Medrano Cabral, D. Veloz)

PMPeabody Museum, Yale University, New Haven, Connecticut (W. Piel, R. Pupedis)

SMNHSStaatliches Museum für Naturkunde Stuttgart (G. Bechley)

USNMUnited States National Museum, Smithsonian Institution, Washington DC, USA (J. Coddington)

## Species groups

Muma (1953) defined six species groups (*banksi* group, *debilis* group, *insularis* group, *lindborgi* group, *mexicanus* group and *spixii* group) based on characters that even he stated were subject to some variation. There were several species which did not fit into any of the Muma (1953) groups. However, given the lack of a more robust framework, modern workers (Alayón-García 1992, 2001, 2003 2005; Valdez-Mondragón 2007, 2010) continue to place species in these groups without any evidence that these groups are phylogenetically valid. The first character, relative leg lengths, is problematic in that spiders tend to easily autotomize the legs to avoid being trapped or eaten, and this is especially common in selenopids, where they are missing legs on both sides of several specimens. If the spider is not yet an adult and will continue to molt, the leg will grow back, however, the leg will be shorter. Also, many of the specimens are in poor condition and legs are disarticulated from the specimen. Finally, leg lengths can differ between males and females of the same species, within the same sex of species, and sometimes on the left and right sides of a single specimen. (Corronca (1996, 1998) defines the genus *Selenops* as having the second legs longer than the fourth, however, Muma (1953) defines species groups by having the second legs longer than the others or the fourth leg shorter than the others. In this study, the leg formulae are given; however, this character appears to be misleading. The comparative eye size also is subject to variation and can differ between the sexes within a single species, and also within species of the same sex. Genitalic characteristics and genetic data appear to be the most powerful tools for species determination, or determination of evolutionary relationships in these spiders, however, it does appear that there is some genitalic homoplasy, as species in different genera may sometimes have superficially similar copulatory organs.

## Monophyly of species groups and the Genus *Selenops*

Previously, a comprehensive phylogenetic hypothesis was assembled for *Selenops* from North America, Central America and the Caribbean based on a large amount of molecular data (Crews and Gillespie 2010). Specific relationships and their association with Caribbean biogeography are discussed in that paper. Here I review those results in connection with previously defined species groups and the monophyly of the genus *Selenops*.

The monophyly of Muma's (1953) species groups cannot be tested thoroughly as there are some species from nearly all of the six species groups that I could not include in a previous study (Crews and Gillespie 2010). However, the data generated statistical support for only a few of Muma's (1953) species groups. The ‘*banksi*' group, consisting of three species, *Selenops banski*, *Selenops micropalpus* and *Selenops minutus* is statistically well-supported, though *Selenops minutus* was not included here (see Crews and Gillespie: fig. 6, clade F). The ‘*debilis*' group, consisting of *Selenops bifurcatus*, *Selenops abyssus*, *Selenops lepidus*, *Selenops buscki*, *Selenops scitus*, *Selenops debilis*, *Selenops actophilus* and *Selenops nesophilus* is recovered, but is lacking statistical support (see Crews and Gillespie 2010: fig. 6, clade I). The ‘*insularis*' group consisting of *Selenops insularis*, *Selenops trifidus*, *Selenops submaculosus*, *Selenops simius*, *Selenops alemani*, *Selenops vinalesi* and *Selenops candidus* is not recovered. The ‘*lindborgi*' group consisting of *Selenops lindborgi*, *Selenops hebraicus* and *Selenops formosus* is not statistically supported as *Selenops lindborgi* and *Selenops hebraicus* occur in distant parts of the tree. It is unknown which taxa were in Muma's ‘*spixii*' group, as these were not explicitly stated. The ‘*mexicanus*' group, originally consisting of only two species *Selenops mexicanus* and *Selenops gracilis*, and additional species, is statistically supported (see Crews and Gillespie 2010: fig. 6, clade H). Several ‘unplaced' species were not available for these analyses, but the following were: *Selenops phaselus*, *Selenops curazao*, and *Selenops marcanoi*. Muma (1953) did not recognize *Selenops phaselus* as fitting into any of the defined groups and its placement in all of the analyses is ambiguous and unsupported. Alayón-García (2001) could find few affinities between *Selenops curazao* and South American or Cuban taxa, but left this species unplaced. In all three analyses it is closely allied with species from Trinidad and Tobago and the northern South American mainland (see Crews and Gillespie 2010: fig. 6, clade C). Alayón-García (1992) also did not place *Selenops marcanoi*, which in all three analyses is closely allied with *Selenops insularis* and several other Hispaniolan species (see Crews and Gillespie 2010: fig. 6, clade M). Placing species in groups based on the aforementioned commonly used morphological characters is unfounded. Based on these analyses, the Muma (1953) species groups are not reliable, and the practice of placing species within this genus in these species groups should be abandoned.

Based on the results of my molecular (Crews and Gillespie 2010) and morphological studies (Crews and Harvey 2011) it would appear that the monophyly of the genus *Selenops* is somewhat questionable. In the molecular phylogenies, *Selenops* is either para- or polyphyletic, though these relationships are not supported. The para- and polyphyly occurs between the Old and New World selenopids. That is, the New World selenopids are monophyletic, and the Old World selenopids are not – either with respect to one another or with the New World selenopids. Benoit (1968, p. 118) noted that American *Selenops* are very different from the African ones, and that they do not have anything at the generic level in common with types from the Old World. He suggested that they should be the object of a new classification. Unfortunately, he did not elaborate further. This conundrum is discussed further in Crews and Harvey (2011), though no better conclusion than Benoit's is reached.

## Taxonomy

### Family Selenopidae

Simon, 1897

http://species-id.net/wiki/Selenopidae\according_to_Crews_2011

Selenopinae Simon 1897: 23.

#### Type genus.

*Selenops* Latreille, 1819.

#### Definition.

All members of the Selenopidae are extremely dorsoventrally flattened, have two tarsal claws and laterigrade legs. They are ecribellate, entelegynes, with eight eyes in two rows; with six in the first row and two in the second row (see also Jocqué and Dippenaar-Schoeman 2006).

#### Description.

See in Crews and Harvey (2011).

#### Distribution.

The Selenopidae occur worldwide and are primarily tropical and subtropical, though several species are found in deserts, and can be found from sea level to over 2500 meters.

### Genus Selenops

Latreille, 1819

http://species-id.net/wiki/Selenops\according_to_Crews_2011

Selenops Latreille 1819: 579 (type species *Selenops radiatus*Latreille 1819: 579).
Hypoplatea MacLeay 1839: 6 (type species *Hypoplatea celer* MacLeay, 1839, from Cuba). Synonymized by Walckenaer, 1842.
Orops Benoit 1968: 116 (type species *Selenops littoricola* Strand, 1913, from Africa). Synonymized by Corronca, 1996.

#### Diagnosis.

The genus *Selenops* can be distinguished from other genera of the family Selenopidae by the ventral leg spination of legs I and II. All members of this genus have three pairs of ventral spines on the tibiae I and II, and two pairs of ventral spines on the metatarsi I and II, denoted as 3–2. Spination for other genera is as follows: *Amamanganops* Leg I 4–3, Leg II 5–3; *Anyphops* 4 to 7 pairs on legs I and II; *Garcorops* 4–3; *Godumops* 7–4; *Hovops* 2–2; *Karaops* Leg I 5 to 6, Leg II 0–4, or unpaired; *Makdiops* Leg I 4–4, 3–3, or 3–2, Leg II 4–4, 4–3, 3–3, or 3–2; *Pakawops* Leg I 7–5 Leg II unknown; *Siamspinops* Leg I 11–15, Leg II 7–13.

#### Description.

Total length of males 4.00–13.00 mm, of females 5.00–18.00 mm; body yellowish to brownish to greyish, mottled with darker markings; legs with bands (Figs 180–181,191). *Cephalothorax*: Carapace in most specimens with dark mottling, dorsoventrally flattened, wider than long with distinct cephalic region, thoracic region laterally convex, fovea longitudinal with radiating grooves, hirsute, clypeus low. Eight eyes outlined in black, six eyes in the anterior row, straight or slightly recurved, two eyes in the posterior row. PLE usually the largest (Fig. 184), ALE usually the smallest. Sternum circular to ovoid with notched posterior projection extending between coxae IV. Chilum absent. Chelicerae geniculate, robust; patura setose with distinct lateral condyles. Fang large, cheliceral promargin with 3 teeth and retromargin with 2 teeth. Labium as wide as, or wider than long, rounded anteriorly, truncate posteriorly; endites ectally convex, subparallel with terminal scopulae. Female pedipalp with claw. *Abdomen*: Longer than wide, ovoid, extremely dorsoventrally flattened, slightly truncate posteriorly; mottled, sometimes with setal tufts along the posterior margin giving the tip of the abdomen a festooned appearance (Figs 179, 187, 190). One pair of book lungs and single tracheal spiracle near spinnerets. Six spinnerets, anterior pair adjacent; colulus absent. *Legs*: Laterigrade, long, tarsi with two claws, in most species the prolateral claw is toothed and the retrolateral claw is smooth, however both claws can also be toothed or not. Claw tufts present. Tarsal scopulae present in most species. Trichobothria present on all leg segments. Legs, especially femora, ringed in black in most species. Tibia and Metatarsus I and II with strong spines, in a 3–2 pattern, respectively. *Copulatory organs*: Male palpus complex, retrolateral tibial apophysis 2 or 3 branched, dorsal branch longer than ventral branch in most species; embolus long, median apophysis 1 or 2 branched, at least one branch sclerotized in most species. Conductor typically large and with a spine- or spur-like apex. Epigyne with lateral lobes or not, spermathecal openings at the caudal end of a median guide or in a median pit, many species with epigynal pockets. Internal ducts variable, spermathecae large and heavily sclerotized in most species, some species with posterodorsal fold.

#### Nomina dubia.

Franganillo (1926) described a subspecies of *Selenops radiatus*, *Selenops radiatus fuscus*, from Cuba, and mentions *Selenops radiatus* occurring in Cuba. However, it is clear that *Selenops radiatus*, or any subspecies, does not occur anywhere in the New World. Additionally, he provided no illustrations with his descriptions. Thus, *Selenops radiatus fuscus* is unrecognizable and should be considered a *nomen dubium*.

#### Distribution.

Tropical and subtropical areas of North, South and Central America, Africa, Madagascar and Asia.

#### Composition.

Currently there are 35 valid species of Old World *Selenops* and 85 recognized species of New World *Selenops*.

##### Key to species of Selenops found in the North America, Central America and the Caribbean, exclusive of Cuban endemics

**Figures 1–6.**
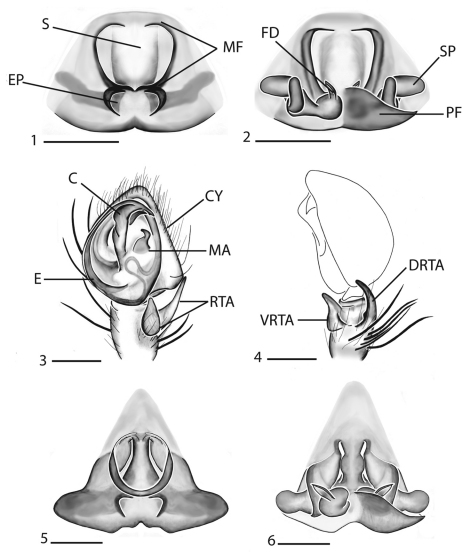
Copulatory organs of *Selenops arikok* sp. n. female holotype from Arikok National Park, Aruba (EME sel_068) **1–2** *Selenops curazao* Alayón-García male holotype from CarMaBI, Curaçao, Netherlands Antilles (MCZ) **3–4** female paratype from Piscadera Baai building, Curaçao, Netherlands Antilles (MCZ) **5–6,** **1, 5** epigyne, ventral view **2, 6** spermathecae, dorsal view **3** male pedipalp, ventral view **4** male pedipalp, retrolateral view. Scale bar = 0.40 mm (1–2), 0.30 mm (3–6). Abbreviations: **S** = septum, **MF** = median field, **EP** = epigynal pockets, **FD** = fertilization duct, **SP** = spermathecae, **PF** = posterodorsal fold, **C** = conductor, **CY** = cymbium, **MA** = median apophysis, **E** = embolus, **RTA** = retrolateral tibial apophysis, **VRTA** = ventral retrolateral tibial apophysis, **DRTA** = dorsal retrolateral tibial apophysis.

* denotes *Selenops actophilus*, *Selenops debilis* and *Selenops nesophilus*, which can be very difficult to separate morphologically owing to apparent variation (see text). The key was made using the type specimens.

(Terms used are illustrated in Figs 1–2 for females and 3–4 for males)

**Table:** 

1	Males	2
–	Females	38
2 (1)	MA with two branches (Figs 21, 79, 159)	3
–	MA with a single branch (Fig. 3)	7
3 (2)	Dorsal branch of RTA much longer than ventral branch	4
–	Dorsal branch of RTA only slightly longer than ventral branch, or the same length as ventral branch	6
4 (3)	Dorsal branch of RTA bifurcated distally with a small lobe projecting laterally in lateral view (Fig. 80)	*Selenops bifurcatus*
–	Dorsal branch of RTA otherwise	5
5 (4)	Dorsal branch of RTA of uniform width in lateral view, embolus very short and located behind another sclerite (Figs 21–22)	*Selenops gracilis*
–	Dorsal branch of RTA wider at base in lateral view, tapering distally; embolus longer (Figs 27–28)	*Selenops mexicanus*
6 (3)	Cymbium round in ventral view, embolus very short (Valdez-Mondragon 2010, Figs 3–4)	*Selenops aztecus*
–	Cymbium oval in ventral view, embolus at least half as long as cymbium, beginning at 7 o'clock, ending at 10 o'clock (Figs 159–160)	*Selenops enriquillo* sp. n.
7 (2)	Embolus with two branches (Figs 97, 105, 109)	8
–	Embolus with a single branch (Figs 3, 43, 91)	10
8 (7)	Base of MA very wide and quadrangular, dorsal branch of RTA very wide, widening distally (Figs 97–98, 109–110)	9
–	Tapering of MA more gradual, base of MA not quadrangular, dorsal branch of RTA uniform in width (Figs 105–106)	*Selenops wilmotorum* sp. n.
9 (8)	RTA with small, conical, medial branch, MA tapering very abruptly to small, slender hook (Figs 97–98)	*Selenops candidus*
–	RTA very large, u-shaped in ventral view, lateral branch bifid, MA tapering, but not as abruptly, forming a stout hook (Figs 109–110)	*Selenops wilsoni* sp. n.
10 (7)	Palpal tibia very long, embolus arising mediolaterally rather than basally (Figs 15–18)	11
–	Palpal tibia not as long, embolus not arising mediolaterally (Figs 7, 11)	12
11 (10)	MA a long, finger-like hook (Fig. 17)	*Selenops micropalpus*
–	MA small, conical, tapering to a small hook (Fig. 15)	*Selenops banksi*
12 (10)	MA small and directed ventrally (Figs 7, 11)	13
–	MA large and/or directed distally (Figs 33, 121)	14
13 (12)	Embolus beginning at 3 o'clock, terminating between 10 and 11 o'clock, both branches of RTA directed distally (Figs 7–8)	*Selenops geraldinae*
–	Embolus beginning at 5 o'clock, ending at 11 o'clock, fairly thick throughout, ventral branch of RTA directed ventrally (Figs 11–12)	*Selenops willinki*
14 (12)	Conductor arising from a long stalk near the center of the palpal bulb (Fig. 3)	15
–	Conductor otherwise	19
15 (14)	Conductor somewhat T-shaped (Figs 145, 167, 169, 175)	16
–	Stalk of conductor somewhat twisted, cymbium angular (Fig. 3)	*Selenops curazao*
16 (15)	Projection on stalk of conductor (Figs 145, 149, 167)	17
–	Projection on stalk of conductor absent	20
17 (16)	Tip of conductor projecting beyond cymbium (Fig. 167)	*Selenops morro* sp. n.
–	Tip of conductor does not project beyond cymbium (Figs 145, 149)	18
18 (17)	Dorsal branch of RTA distally pointed, MA shorter than stalk of conductor (Figs 145–146)	*Selenops kalinago* sp. n.
–	Dorsal branch of RTA rounded, MA nearly as long as stalk of conductor (Figs 149–150)	*Selenops souliga* sp. n.
19 (17)	Dorsal branch of RTA rounded distally, embolus and conductor extending beyond edge of cymbium (Figs 169–170)	*Selenops simius*
–	Dorsal branch of RTA truncate, embolus and conductor not extending beyond border of cymbium (Figs 175–176)	*Selenops submaculosus*
20 (16)	Conductor distally c-shaped, MA large and directed ventrally (Fig. 153)	*Selenops bani*
–	Conductor and MA otherwise	21
21 (20)	Conductor large, as long as palpal bulb, and angular, with no obvious connection to middle of bulb (Figs 53, 59, 61)	22
–	Conductor otherwise, with long embolus arising basally	24
–	Conductor otherwise, with very short embolus, arising medially	26
22 (21)	RTA with a stalk with a large quadrangular process distally	23
–	RTA forming a v-shape in lateral view (Fig. 60)	Selenops debilis*
23 (22)	RTA smooth, embolus beginning at 5 o'clock, terminating at 11 o'clock, MA with a long, finger-like process partially covering conductor (Figs 54)	Selenops actophilus*
–	RTA a ridge, embolus beginning at 6 o'clock, terminating at 11 o'clock, long, finger-like process of MA does not reach conductor (Figs 61–62)	Selenops nesophilus*
24 (21)	RTA with a stalk, terminating in a very large triangular process covering much of the cymbium in lateral view (Figs 69–70)	*Selenops lepidus*
–	RTA smaller, or without stalk and large distal process	25
25 (24)	RTA small and simple, ventral branch pointed in lateral view, dorsal branch truncate in lateral view, MA long and hook-like (Figs 75–76)	*Selenops petenajtoy* sp. n.
–	Ventral branch of RTA distally rounded and directed toward lateral branch in ventral view (Valdez-Mondragon 2007, Figs 4–5)	*Selenops juxtlahuaca*
26 (21)	Embolus very small, arising near center of bulb and terminating just above, MA located proximally (Fig. 141)	*Selenops baweka* sp. n.
–	Embolus and MA otherwise	27
27 (26)	Embolus curved around or toward lateral edge of cymbium (Figs 95, 121, 125)	28
–	Embolus directed distally, not curving around edge of cymbium (Figs 43, 91)	34
28 (27)	RTA very large and curving laterally, lateral branch with a small process arising basally (Figs 95–96)	*Selenops aissus*
–	RTA otherwise	29
29 (28)	MA with a large base, tapering abruptly (Figs 41, 49)	30
–	MA more uniform, tapering gradually (Figs 121, 135)	32
30 (29)	Embolus curving around edge of the cymbium, RTA truncate in lateral view (Figs 49–50)	*Selenops scitus*
–	Embolus not reaching edge of cymbium, RTA otherwise	31
31 (30)	Base of MA conical, tapering to a small hook, RTA with distally pointed processes in lateral view (Fig. 41)	*Selenops morosus*
–	Base of MA ovoid, tapering to a large hook, RTA quadrangular with a small pointed process in lateral view directed ventrally (Figs 131–132)	*Selenops trifidus*
32 (29)	RTA very small, arising distally on palpal tibia (Figs 161–162)	*Selenops phaselus*
–	RTA larger, arising basally on palpal tibia	33
33 (32)	Dorsal branch of RTA truncate, only widening slightly distally, conductor forming an arch at lateral and medial connections to bulb (Figs 125–126)	*Selenops marcanoi*
–	Dorsal branch of RTA slightly sinuous, slanted, widening distally, conductor forming a circle at lateral and medial connections to bulb (Figs 121–122)	*Selenops insularis*
34 (27)	Conductor slightly hammer shaped, directed retrolaterally (Fig. 135)	*Selenops denia* sp. n.
–	Conductor otherwise	35
35 (34)	Embolus arising mediobasally and terminating just over halfway up palpal bulb, RTA quadrangular with one small distal pointed projection (Figs 91–92)	*Selenops lindborgi*
–	Embolus longer, RTA otherwise	36
36 (35)	Conductor rounded at terminus (Fig. 33)	*Selenops marginalis*
–	Conductor pointed at terminus (Figs 37, 43)	37
37 (36)	Embolus arising from a round base, abruptly truncate, conductor pointed, tip extending beyond edge of cymbium, RTA claw shaped in lateral view (Figs 37–38)	*Selenops minutus*
–	Embolus tapering gradually, RTA on a small stalk with a small quadrangular process distally (Figs 43–44)	*Selenops nigromaculatus*
38 (1)	Epigynum with a distinct median septum (Figs 1, 5, 19, 23, 51, 55)	39
–	Epigynum without median septum, with central depression or openings (Figs 9, 45, 119)	55
39 (38)	Septum surrounded by sclerotized ring or square (Figs 1, 5, 19)	40
–	Septum not surrounded by sclerotized ring or square (23, 51, 55)	41
40 (39)	Septum surrounded by sclerotized ring, epigynal plate triangular in shape overall (Fig. 5)	*Selenops curazao*
–	Septum surrounded by sclerotized quadrangular area, epigynal plate squarish overall (Fig. 1)	*Selenops arikok* sp. n.
41 (39)	Median septum located distally (Fig. 19)	*Selenops micropalpus*
–	Median septum located medially or proximally (Figs 23, 25, 29)	42
42 (41)	Median septum hyaline, with epigynal pockets located laterally (Figs 23, 25, 29)	43
–	Median septum sclerotized (Figs 55, 57, 77, 85)	45
43 (42)	Median septum extending downward, touching sclerotized area where epigynal pockets are located (Figs 23, 25)	44
–	Median septum located medially, not extending downward to epigynal pockets (Fig. 29)	*Selenops mexicanus*
44 (43)	Spermathecae oval fertilization ducts located laterally, posterodorsal fold rectangular (Figs 23–24)	*Selenops gracilis*
–	Spermathecae oblong, posterodorsal fold medially depressed, forming a u-shape (Figs 25–26)	*Selenops malinalxochitl* sp. n.
45 (42)	Median septum extending beyond epigastric furrow (Valdez-Mondragon, 2007, Figs 7–8)	*Selenops juxtlahuaca*
–	Median septum not extending beyond epigastric furrow	46
46 (45)	Median septum extending to edge of epigynal plate (Figs 63, 77)	47
–	Terminus of median septum located more medially, or covered medially by lateral lobes (Figs 71, 85)	52
47 (46)	Proximal edge of epigynum sinuous, with raised areas laterally (Fig. 63)	Selenops nesophilus*
–	Proximal edge of epigynum not sinuous, lateral raised areas absent	48
48 (47)	Lower margin of epigynal plate extended laterally and upwards, sclerotized sides extend as far distally as median septum (Figs 81–82)	*Selenops bifurcatus*
–	Epigynal plate not extended laterally and distally	49
49 (48)	Internal copulatory organs with two long slender spermathecae (Figs 55, 57)	50
–	Spermathecae small and compact (Fig. 51)	51
50 (49)	Spermathecae with two branches on each side, directed distally (Fig. 55)	Selenops actophilus*
–	Spermathecae with one branch on each side, directed laterally (Figs 57–58)	Selenops debilis*
51 (49)	Median septum small and narrow, internal ducts branched once (Figs 51–52)	*Selenops ixchel* sp.
–	Median septum large and wide, internal ducts with two branches (Fig. 77)	*Selenops huetocatl* sp. n.
52 (46)	Internal ducts coiled (Figs 72, 74)	53
–	Internal ducts otherwise	54
53 (52)	Median septum seemingly covered by lateral lobes, located in center of plate, right lobe overlapping left (Fig. 71 )	*Selenops lepidus*
–	Median septum not covered, located in the lower third of plate, lateral lobes not overlapping (Fig. 73)	*Selenops chamela* sp. n.
54 (52)	Median septum ovoid, lateral lobes conspicuous proximally (Figs 83–84)	*Selenops buscki*
–	Median septum quadrangular to angular, lateral lobes not obvious (Figs 85–86)	*Selenops oricuajo* sp. n.
55 (38)	Epigynum with v-shaped median area, genital openings located at lateral margins of v (Figs 31, 87, 119)	56
–	Median area and genital openings otherwise (Figs 89, 93)	59
56 (55)	Internal ducts coiled extensively (Figs 87–88)	*Selenops amona* sp. n.
–	Internal ducts otherwise	57
57 (56)	Lateral lobes fused, not obvious (Fig. 31)	*Selenops abyssus*
–	Lateral lobes distinct	58
58 (57)	Large posterodorsal fold covering spermathecae (Figs 119–120)	*Selenops insularis*
–	Posterodorsal fold absent (Figs 163–164)	*Selenops phaselus*
59 (55)	Median area an anteriorly projecting lobe (Figs 107, 133)	60
–	Median area otherwise	61
60 (59)	Lateral lobe separation extending the length of anteriorly projecting lobe, posterodorsal fold extending less than 1/3 length of epigynal plate (Fig. 133)	*Selenops duan* sp. n.
–	Lateral lobes do not extend the length of anteriorly projecting lobe, posterodorsal fold huge, more than ? the length of epigynal plate (Fig. 107)	*Selenops wilsoni* sp. n.
61 (59)	Genital openings located anteriorly (Figs 13, 127, 129, 143)	62
–	Genital openings located elsewhere	65
62 (61)	Epigynal pockets located medially (Fig. 13)	*Selenops willinki*
–	Epigynal pockets located elsewhere or absent	63
63 (62)	Posterodorsal fold absent, genital openings located beneath a small slit (Figs 143–144)	*Selenops kalinago* sp. n.
–	Posterodorsal fold present	64
64 (63)	Genital openings located behind a small circular area, posterodorsal fold not entirely covering spermathecae, epigynal pockets small (Figs 127–128)	*Selenops bocacanadensis* sp. n.
–	Genital openings located in a large crescent shaped area, posterodorsal fold completely covering spermathecae, epigynal pockets large (Figs 129–130)	*Selenops oviedo* sp. n.
65 (61)	Median field a keyhole-shaped area (Fig. 45)	*Selenops makimaki* sp. n.
–	Median field otherwise	66
66 (65)	Internal copulatory organs coiled anteriorly to posteriorly (Figs 101–102)	*Selenops petrunkevitchi*
–	Internal copulatory organs otherwise	67
67 (66)	Posterior margin of epigynum extending downward into two points (Figs 93, 155)	68
–	Posterior margin of epigynum otherwise	69
68 (67)	Lateral lobes indistinct, spermathecae large and round, located medially (Figs 93–94)	*Selenops aissus*
–	Lateral lobes distinct, spermathecae small, oval, located laterally (Figs 155–156)	*Selenops pensilis*
69 (67)	Posterodorsal fold absent (Figs 40, 48, 148, 152)	70
–	Posterodorsal fold present	73
70 (69)	Median field a large, heart-shaped area, spermathecae huge (Figs 147–148)	*Selenops souliga* sp. n.
–	Median field otherwise	71
71 (70)	Genital openings located behind sinuous medial margin (Figs 151–152)	*Selenops bani*
–	Genital openings located elsewhere	72
72 (71)	Lateral lobes distinct, not touching medially, spermathecae small and round (Figs 39–40)	*Selenops minutus*
–	Lateral lobes indistinct, median area pinched anteriorly to posteriorly, spermathecae large (Figs 47–48)	*Selenops scitus*
73 (69)	Median field quadrangular, epigynal pockets absent (Figs 35)	*Selenops marginalis*
–	Median field otherwise	74
74 (73)	Posterodorsal fold completely covers spermathecae (Fig. 124)	*Selenops marcanoi*
–	Posterodorsal fold not as extensive	75
75 (74)	Spermathecae ovoid, sperm ducts coiling twice (Fig. 90)	*Selenops lindborgi*
–	Spermathecae and sperm ducts otherwise	76
76 (75)	Genital openings located along the margins of a median depression (Figs 9, 99)	77
–	Genital openings located beneath a straight to sinuous medial margin (Figs 103, 137, 165)	81
77 (76)	Median depression round to quadrangular, anterior to small epigynal pockets (Fig. 99)	*Selenops candidus*
–	Median depression otherwise	78
78 (77)	Median depression extending to nearly lateral margins of epigynal plate, lateral lobes come together medially, then curve outwards laterally (Figs 9–10)	*Selenops geraldinae*
–	Median depression not extending to lateral margins of epigynal plate	79
79 (78)	Posterior margin of median field sinuous, internal ducts coiled (Figs 173–174)	*Selenops submaculosus*
–	Posterior margin of median field smooth, median field u-shaped	80
80 (79)	Lateral lobes distinct, internal ducts strongly sclerotized, small round spermathecae located laterally (Figs 157–158)	*Selenops enriquillo* sp. n.
–	Lateral lobes indistinct, internal ducts strongly coiled (Figs 171–172)	*Selenops simius*
81 (76)	Median margin extending to lateral margins of epigynal plate (Figs 165–166)	*Selenops morro* sp. n.
–	Median margin not extending to lateral margins of epigynal plate	82
82 (81)	Median margin sinuous, lateral lobes fused in center of plate giving a pinched appearance to the openings and median margin (Figs 103–104)	*Selenops wilmotorum* sp. n.
–	Median margin angular to straight, lateral lobes indistinct	83
83 (82)	Median margin nearly straight, ducts not touching anteriorly, posterodorsal fold small (Figs 137–138)	*Selenops guerrero* sp. n.
–	Median margin angular, ducts touching anteriorly, posterodorsal fold large, centrally nearly half the length of the epigynal plate (Figs 139–140)	*Selenops baweka* sp. n.

##### Survey of species

Species are arranged by molecular phylogenetic similarity (see Crews and Gillespie 2010) or, for those species in which genetic data was not available, by similarities in the genitalic characteristics.

### Selenops arikok sp. n.

urn:lsid:zoobank.org:act:7224F8C2-50FD-41C0-B366-310D677A0FD9

http://species-id.net/wiki/Selenops_arikok

Figs 1–2 177 Map 1

#### Type material.

Holotype female: near Gran Tonel in valley Rooi Coashati, Arikok National Park, Aruba, 12°29.356'N, 69°55.461'W, ~96 m, 16.X.2004, S. Crews, F. Franken, under bark, SCC04_045, (EME sel_068).

#### Other material examined.

ARUBA: Bringamosa, house of R. Croes, 12°29.547'N, 69°58.077'W, 14.X.2004, S. Crews, under rocks, with egg sacs, SCC04_041, 1♀, 1 imm. (CAS sel_069-070); Luela, shooting range, 12°29.023'N, 69°57.778'W, ~30 m, 15.X.2004, S. Crews, under piece of wood, SCC04_044, 1 imm. (EME sel_072).

#### Etymology.

The specific epithet refers to the type locality, Arikok National Park, and should be treated as a noun in apposition.

#### Diagnosis.

This species is most similar to *Selenops curazao* and *Selenops isopodus*, but can be distinguished by the more quadrangular and more hyaline median field than *Selenops curazao*, and unlike *Selenops isopodus*, the median field is not completely enclosed anteriorly. The shape of the epigynal plate and the internal copulatory organs also differ significantly (Figs 1–2). Males unknown.

#### Remarks.

Even though only two adult female specimens have been collected, some variation is seen in the shape of border of the median field of the epigynum. In the holotype it is wider anteriorly, and more quadrangular in general, while in the other specimen, the median field is more rounded, and not wider anteriorly than it is posteriorly.

#### Description.

*Holotype female*: Color: Carapace brown-yellow with cephalic area primarily brown, dusky spots medially and laterally, with white setae; sternum orange-brown, darker around border; chelicerae dark brown, lightening anteromedially; maxillae light orange-brown, dark on outer distal edge, white on inner distal edge; labium light brown, lightening distally; abdomen dorsally cream-colored with some duskier flecks, and dark festoon caudolaterally; ventrally pale yellow, no markings; legs cream-yellow, darker distally, with annulations on all segments except the tarsi. Carapace: 0.92 times longer than broad. Eyes: AER slightly recurved; PER recurved; PME larger than AME, PLE largest, ALE smallest; eye diameters, AME 0.08, ALE 0.05, PME 0.15, PLE 0.40; interdistances AME-PME 0.13, PME-ALE 0.10, ALE-PLE 0.40. PME-PME 1.70. ALE-ALE 2.33; ocular quadrangle AME-AME 0.35, PLE-PLE 2.00; clypeus 0.56 high. Mouthparts: Chelicerae with stout setae medially and anteriorly; labium distally rounded. Sternum: as long as broad, posteriorly indented. Legs: leg I only slightly shorter than legs II, III and IV; leg formula 3241; scopulae present on all tarsi, as well as on first three metatarsi, and on the distal portion of the tibiae of legs I and II; tarsi I-IV with strong claw tufts; pr claw per foot with a few teeth; spination: leg I, Fm pr 1–1–0, d 1–1–1, rl 1–1–1; Ti d 0, v 2–2–2; Mt v 2–2; leg II, Fm pr 1–1–0, d 1–1–1, rl 1–1–1; Ti v 2–2–2; Mt v 2–2; leg III, Fm pr 1–1–0, d 1–1–1, rl 1–1–1; Ti v 2–2–0; Mt v 2–2–1; leg IV, Fm pr 1–1–0, d 1–1–1, rl 0–1–1; Ti v 2–2–0; Mt v 2–2. Abdomen: with terminal setal tufts. Pedipalp: claw with 11 teeth. Epigyne: epigynal plate subquadrangular, lateral lobes indistinct, hyaline median septum present, epigynal pockets present; spermathecae ovoid, directed laterally, posterodorsal fold present and covers much of the internal ducts (Figs 1–2). *Dimensions:* Total length 7.23. Carapace length 3.33, width 3.60. Width 1.72. Abdomen length 3.90, width 2.75. Pedipalp: Fm 1.00, Pt 0.35, Ti 0.30, Ta 1.00, total 2.65. Leg I: Fm 3.25, Pt 0.25, Ti 3.00, Mt 2.00, Ta 1.20, total 9.70. Leg II: Fm 3.75, Pt 1.30, Ti 3.25, Mt 2.25, Ta 1.25, total 11.80. Leg III: Fm 4.00, Pt 1.25, Ti 3.25, Mt 2.25, Ta 1.25, total 12.00. Leg IV: Fm 3.75, Pt 1.00, Ti 3.00, Mt 2.25, Ta 1.15, total 11.15.

#### Natural History.

Collected under rocks, bark, and debris on the ground, both near and away from human dwellings. The egg sac is a flat, white disc attached to the substrate and guarded by the female (Fig. 177).

#### Distribution.

**Map 1.**
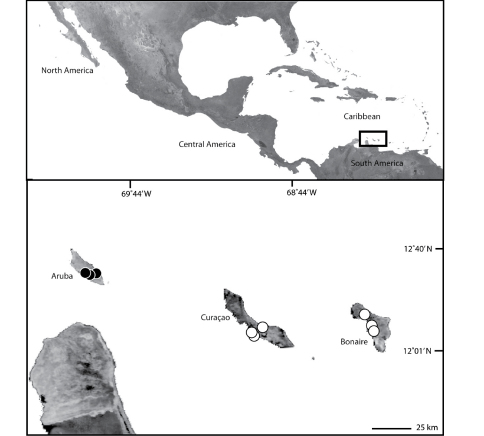
Map of Aruba, Curaçao and Bonaire (inset) showing the distribution of *Selenops*. *Selenops arikok* sp. n. (black circles), *Selenops curazao* Alayón-García (white circles).

Known only from the island of Aruba (Map 1).

### Selenops curazao

Alayón-García, 2001

http://species-id.net/wiki/Selenops_curazao

Figs 3–6 196 Map 1

Selenops curazao Alayón-García 2001: 17–20, Figs 1–4 (♂, ♀, examined).

#### Type material.

Holotype male from, CarMaBI (Caribbean Marine Biology Institute), Curaçao, Netherlands Antilles, H. Campbell, IX.1963 (MCZ, examined). Paratypes. Female from Piscadera Baai building, Curaçao, Netherlands Antilles, H. & L. Levi, 18–30.XII.1962 (MCZ, examined).

#### Other material examined.

NETHERLANDS ANTILLES: Bonaire: Altamira Ungu, along dirt road, 12°13.949'N, 68°20.703'W, ~100 m, 12.X.2004, S. Crews, under rocks in thornscrub, SCC04_038, 1♀, 2♂ (EME sel_059-060, 082); Nort di Saliña, Kaya Otomac, 12°10'39.86"N, 68°16'19.53"W, 11-12.X.2004, G. van Hoorn, in house, SCC04_037, 5♀, 4 imm. (EME sel_054-057, 061-065); Sabadaco, across street from cave, 12°11.587'N, 68°17.765'W, 11.X.2004, S. Crews, under rocks in pile from construction, SCC04_034, 1 imm. (EME sel_053). **Curaçao:** Carmabi Institute, 12°07.351'N, 68°58.132'W, sea level, 7.X.2004, S. Crews, under wood and rocks behind building, SCC04_026, 1♀, 2 imm. (CAS sel_047, 068, 217); Girouette Plantation, East of Schottegat Harbor, 12°07'57.38"N, 68°54'57.34"W, house of A. DeBrot, 8.X.2004, in house and in boulder pile outside of house, SCC04_032, 1♂, 4 imm. (CAS sel_048-052).

#### Diagnosis.

The females of this species most closely resemble *Selenops isopodus* found in Colombia and *Selenops arikok* sp. n., of which the males are unknown. Females can be distinguished from these species by the epigyne, as *Selenops curazao* has a narrow septum, and the internal copulatory organs are quite different (Figs 5–6). The male is easily differentiated from other species by the angular cymbium, the long twisted stalk of the conductor, and the shape of the tibial apophyses (Figs 3–4)

#### Description.

*Holotype male:* Color: carapace (holotype) brown-orange, duskier at lateral margins (recent) cephalic area not as contrasted with the rest of carapace, duskier in center and on sides of carapace, with white setae; sternum (holotype) orange-brown, darker at border (recent) pale yellow, slightly darker around border; chelicerae orange-brown with dusky longitudinal lines; maxillae (holotype) orangeish, lightening distally (recent) pale yellow, lightening distally; labium (holotype) orange-brown, lightening distally (recent) yellow-brown lightening distally; abdomen, dorsally cream-colored with some duskier flecks and dark festoon caudolaterally; ventrally, cream-colored; legs (holotype) orange-brown with light annulations which are darker ventrally and the metatarsi and tarsi are darker (recent) dusky yellow with faint annulations which are more visible ventrally, with the metatarsi and tarsi darker. Carapace: 0.88 times longer than broad. Eyes: AER slightly recurved; PER recurved; PME larger than AME, PME same as PLE, ALE smallest; eye diameters, AME 0.10, ALE 0.03, PME 0.28, PLE 0.28; interdistances AME-PME 0.03, PME-ALE 0.10, ALE-PLE 0.28. PME-PME 0.83 mm. ALE-ALE 1.45; ocular quadrangle AME-AME 0.30, PLE-PLE 1.53; clypeus 0.10 high. Mouthparts: chelicerae with a few stout setae medially and anteriorly; maxillae longer than broad, with tuft of conspicuous setae distally; labium distally rounded. Sternum: as long as broad, posteriorly indented. Legs: leg I much shorter than legs II, III and IV; leg formula 2431; scopulae present on tarsi of all legs, and metatarsi of I and II; tarsi I-IV with strong claw tufts; pr claw slightly toothed; spination: leg I, Fm pr 1–1–1, d 1–1–1, rl 1–1–1; Ti d 1–1–0, pr (R) 0–1–1, (L) 0–0–1, v (R) 1–1–2–2 (L) 2–1–2–2, rl 0–1–1; Mt pr 1–1–0, v 2–2–0, rl 1–0–0; leg II, Fm pr 1–1–0, d (R) 1–1–1 (L) 1–1–1–1, rl 1–1–1; Ti pr (R) 1–1–0 (L) 1–1–1, d 1–1–0, rl 0–1–1, v 2–2–2; Mt pr 1–1–0, v 2–2, rl 1–1–0; leg III, Fm pr 1–1–1, d 1–1–1, rl 1–1–1; Ti pr 1–1–0, d (R) 1–1–0 (L) 1–0–0, rl 1–1–0, v (R) 2–2–1 (L) 2–2–0; leg IV, Fm pr 1–1–1, d 1–1–1, rl 0–1–1; Ti pr 1–1–0, v 2–2–0, rl 1–1–0; Mt pr 1–1–0, v 2–2, rl 1–1–0. Abdomen: with terminal setal tufts. Pedipalp: Fm, spination d 0–1–4; cymbium triangular in ventral view. Conductor large, arising from a long, slightly twisted stalk in the center of bulb, pointed laterally toward the 1 o'clock position, not extending beyond edge of bulb, curving retrolaterally around bulb; embolus long, slender, curved tapering distally, beginning at 6 o'clock, terminating at 12 o'clock; MA located at 3 o'clock position, directed distally, with stout base, tapering, and terminating in curved, single hook; RTA with two tibial apophyses, ventral process shorter and more angular than retrolateral process, which is slightly curved ventrally, slender, pointed distally, with small tooth at the base; tibial apophyses barely reach cymbium in ventral view (Figs 3–4). *Dimensions:* Total length 5.28. Carapace length 2.83, width 3.23. Sternum length 1.25, width 1.25. Abdomen length 2.45, width 1.68. Pedipalp: Fm 1.00, Pt 0.25, Ti 0.25, Ta 0.75, total 2.25. Leg I: Fm 3.00, Pt 1.20, Ti 3.00, Mt 2.80, Ta 1.40, total 11.40. Leg II: Fm 3.75, Pt 1.25, Ti 3.25, Mt 3.15, Ta 1.30, total 12.70. Leg III: Fm 3.25, Pt 1.00, Ti 3.20, Mt 3.00, Ta 1.25, total 11.70. Leg IV: Fm 3.80, Pt 1.00, Ti 3.00, Mt 3.00, Ta 1.25, total 12.05.

*Paratype female*: carapace (type) brown-yellow, uniformly dark brown with white setae in cephalic region (recent) cephalic area not as contrasted with the rest of carapace, duskier medially and laterally; sternum pale yellow, darker around border; chelicerae (type) orange-brown, (recent) orange-brown with dusky medial area; maxillae pale yellow, lighter distally; labium dusky yellow, lightening distally; abdomen, dorsally cream-colored with some duskier flecks, dark festoon caudolaterally; ventrally, cream-colored; legs (type) tan with annulations from femora to metatarsi, tarsi uniformly tan; (recent) cream-yellow, darker distally, with annulations, except on tarsi, that don't completely encircle the leg. Carapace: 0.90 times longer than broad. Eyes: AER slightly recurved; PER recurved; PME larger than AME, PLE largest, ALE smallest; eye diameters, AME 0.13, ALE 0.05, PME 0.25, PLE 0.35; interdistances AME-PME 0.08, PME-ALE 0.11, ALE-PLE 0.30. PME-PME 1.18. ALE-ALE 1.98; ocular quadrangle AME-AME 0.40, PLE-PLE 2.00; clypeus 0.09 high. Mouthparts: chelicerae with stout setae medially and anteriorly; maxillae longer than broad, with tuft of conspicuous setae distally; labium distally rounded. Sternum:as long as broad, posteriorly indented. Legs:leg formula 3124; legs I and II with tarsal and metatarsal scopulae; tarsi I-IV with strong claw tufts; pr claw per foot slightly toothed; spination: leg I, Fm pr 1–1–0, d 1–1–1, r. 1–1–1; Ti d 0, v 2–2–2; Mt v 2–2; II, Fm pr 1–1–0, d 1–1–1, rl 1–1–1; Ti v 2–2–2; Mt v 2–2; III, Fm pr 1–1–0, d 1–2–1, rl 0–0–1; Ti v 2–2–0; Mt v 2–2–1; IV, Fm pr 1–1–0, d 1–1–1, rl 0–0–1; Ti v 2–2–0; Mt v 2–2–1. Abdomen:with terminal setal tufts. Pedipalp:claw with 7 teeth. Epigyne:epigynal plate subtriangular, abruptly widening caudally, lateral lobes indistinct, with a roundish median field encircling septum, genital openings located laterodistally with respect to median septum; internal copulatory organs large, heavily sclerotized, sperm ducts large, slightly twisted, leading to roundish spermathecae, posterodorsal fold present, covers part of the spermathecae (Figs 5–6). *Dimensions:* Abdomen damaged. Carapace length 3.50, width 3.90. Sternum length 3.85, width 3.85. Pedipalp: Fm 0.90, Pt 0.40, Ti 0.25, Ta 1.00, total 2.55. Leg I: Fm 3.15, Pt 1, Ti 2.25, Mt 2.20, Ta 1.00, total 9.60. Leg II: Fm 3.15, Pt 1.00, Ti 2.25, Mt 2.00, Ta 1.00, total 9.40. Leg III: Fm 3.30, Pt 1.10, Ti 3.00, Mt 2.25, Ta 1.25, total 10.90. Leg IV: Fm 3.00, Pt 1.00, Ti 2.25, Mt 2.15, Ta 1.00, total 9.30.

#### Natural history.

This species has been collected under wood, rocks, cactus and other debris on the ground, as well as on, in, and near buildings, including houses. In houses it is common at night around the ceiling molding where it hides during the day. It has been found in dry, thornscrub habitat, dominated by *Acacia* (Fig. 196). The egg sac is flat, white, and disc-shaped, and is guarded by the female. One specimen laid 30–40 eggs which hatched about 2 weeks later. After about a week, the spiderlings emerged from the egg sac. The mother died about a week later.

#### Distribution.

Netherlands Antilles islands of Curaçao and Bonaire (Map 1).

### Selenops geraldinae

Corronca, 1996

http://species-id.net/wiki/Selenops_geraldinae

Figs 7–10 177 Map 2

Selenops geraldinae Corronca 1996: 95, Figs 9–10 (♂ holotype from Mara, Venezuela (CAS, not available for examination).
Selenops geraldinae Corronca, 1998: 134, Figs 19–20 (♂).
Selenops willinki Corronca, 1998: 146, Figs 70–71 (♀ only, misidentification).

#### Note.

The holotype of *Selenops geraldinae* Corronca is stated to be deposited in the CAS (Corronca 1996: 95), but the specimen has not arrived at CAS. Nevertheless, the description and illustrations are adequate to recognize as this species the specimens described and listed below.

#### Material examined.

TRINIDAD AND TOBAGO: Gaspar Grande Island: Chaguaramas, trails above Bay View Resort, 10°39'46.5"N, 61°38'58.0"W, ~3-40 m, 12.VII.2005, SCC05_037, 4♂, 1♀, 4 imm. (EME sel_224-229, 237, 241, 254, 257, 260). **Huevos Island:** 10°41'28.3"N, 61°42'55.0"W, sea level, 13.VII.2005, S. Crews, F. (Max) Clunis, under palm frond on trunk, on top of previously hatched egg sac, SCC05_039, 1♂ (CAS sel_239). **Monos Island:** South Sea, 10°40'54.2"N, 61°41'21.6"W, sea level, 13.VII.2005, S. Crews, F. (Max) Clunis, under bark, SCC05_038, 1♂, 4 imm. (CAS sel_244, 247-250). **Chacachacare Island:** 10°41'24.2"N, 61°44'53.7"W, 13.VII.2005, S. Crews, F. (Max) Clunis, under pile of rocks in front of abandoned house, SCC05_040, 1 imm. (CAS sel_246). **St. George County:** Point Cord Road near Trinidad military base, off Chaguaramas Main Road, 10°40'47.1"N, 61°37'30.9"W, ~13 m, 11.VII.2005, S. Crews, C. Chaboo, SCC05_035, 6 imm. (CAS sel_218-223). **VENEZUELA:** **Bolívar:** Guri, South of Puerto Ordaz, ex: FIT, 3-15.VII.1998, H. & A. Howden, fairly wet forest, mostly lowland evergreen, 1♂ (AMNH).

#### Diagnosis.

**Figures 7–14.**
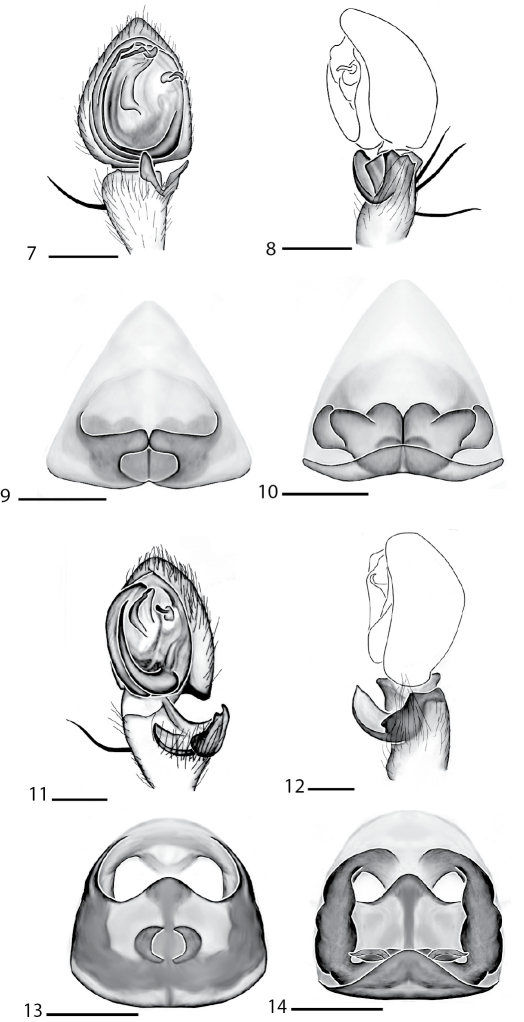
Copulatory organs of*Selenops geraldinae* Corronca male from Gaspar Grande Island, Trinidad and Tobago (EME sel_224) **7–8** female from Gaspar Grande Island, Trinidad and Tobago (EME sel_225) **9–10** *Selenops willinki* Corronca male from Little Tobago, Trinidad and Tobago (EME sel_251) **11–12** female from Little Tobago, Trinidad and Tobago (EME sel_236) **13–14,** **7, 11** male pedipalp, ventral view **8, 12** male pedipalp, retrolateral view **9, 13** epigyne, ventral view **10, 14** spermathecae, dorsal view. Scale bar = 0.45 mm (7–8), 0.35 mm (9–10, 13–14), 0.50 mm (11–12).

Males of this species can be distinguished from others by the small, finger-like MA directed distally, located distally on the palpal bulb (Figs 7–8). Females of *Selenops geraldinae* can be separated from others by the large central, lateral extending depression of the epigynum, lateral lobes that come together medially and diverge laterally (Figs 9–10).

#### Remarks.

The male of *Selenops geraldinae* was described by Corronca (1996). In Trinidad and several small nearby islands, I collected several males of *Selenops geraldinae* and only with females of what Corronca (1998) had previously described as *Selenops willinki*. Along with this natural history data, molecular genetic data (Crews and Gillespie 2010) confirmed that these males and females were conspecific, exclusive of any other nearby specimens (e.g., from Tobago).

In Corronca (1998), the records of *Selenops geraldinae* are given as Mara and Bolívar, Venezuela. They are shown on the map as being in Zulia, near Lago Maracaibo, in the east of the country. However, there are also localities with the same names in the western part of Venezuela. As these specimens have not been examined, it is unclear where exactly they are from. I have shown the distribution following Corronca (1998).

#### Description.

*Male (sel_224).* Color: carapace yellowish-orange, duskier on edge, around border of cephalic area; sternum pale yellow; chelicerae yellow, dusky u-shaped markings terminating halfway down; maxillae pale yellow; labium pale yellow; abdomen dorsally dusky yellow, some spots medially, darker laterally and posteriorly, festoon present; ventrally pale yellow, no markings; legs yellowish-orange, annulations not visible. Carapace: 1.03 times longer than broad; fovea longitudinal, broad, very shallow. Eyes:AER slightly recurved; PER recurved; PME larger than AME, PME same as PLE, ALE smallest; eye diameters, AME 0.18, ALE 0.05, PME 0.23, PLE 0.23; interdistances AME-PME 0.03, PME-ALE 0.08, ALE-PLE 0.28. PME-PME 0.88. ALE-ALE 1.38; ocular quadrangle AME-AME 0.28, PLE-PLE 1.50; clypeus 0.13 high. Mouthparts:chelicerae with stout setae medially and anteriorly; maxillae longer than broad, with tuft of conspicuous setae distally; labium distally rounded. Sternum:1.33 times longer than broad, posteriorly indented. Legs:leg I=leg IV, but only slightly shorter than leg II; leg formula 21=43; scopulae present on distal end of all 4 tarsi; tarsi I-IV with strong claw tufts; pr claw per foot slightly toothed; spination: leg I, Fm pr 1–1–1, d 1–1–1, rl 1–1–1; Ti pr 0–1–1, d 1–1–0, rl 1–0–1, v 2–2–2; Mt pr 1–1–0, rl 1–1–0, v 2–2; leg II, Fm pr 1–1–1, d 1–1–1, rl 1–1–1; Ti pr 0–1–1, d 1–1–0, rl 1–0–1, v 2–2–2; Mt pr 1–1–0, v 2–2, rl 1–1–0; leg III, Fm pr 1–1–1, d 1–1–1, rl 1–1–1; Ti pr 1–0–1, rl 1–0–1, v 2–2; Mt pr 1–1–0, rl 1–1–0, v 2–2; leg IV, Fm pr 1–1–1, d 1–1–1, rl 1–1–1; Ti pr 1–0–1, v 2–2–0, rl 1–0–1; Mt pr 1–0–1, rl 1–1–0, v 2–2. Abdomen:with terminal setal tufts. Pedipalp:Fm, spination dorsal 0–1–4; cymbium triangular in ventral view, angled posterolaterally, setae scattered, denser toward tip; conductor arising in center of bulb, quadrate, distally sinuous; embolus long, slender, arising at 3 o'clock ending at 10 o'clock, tapering distally; MA finger-like, slender, slightly hooked at tip, located at 2 o'clock, directed ventrally; RTA with single apophysis with 2 processes, the dorsal process wide,quadrangular, the ventral process narrower,rounded, barely reaching cymbium in ventral view (Figs 7–8). *Dimensions:* Total length 6.60. Carapace length 3.60, width 3.50. Sternum length 2.00, width 1.50. Abdomen length 3.00, width 2.00. Pedipalp: Fm 1.50, Pt 0.35, Ti 0.75, Ta 1.15, total 3.70. Leg I: Fm 3.50, Pt 1.50, Ti 3.00, Mt 2.90, Ta 1.60, total 12.50. Leg II: Fm 4.00, Pt 1.30, Ti 3.00, Mt 3.00, Ta 1.60, total 12.90. Leg III: Fm 4.00, Pt 1.00, Ti 3.00, Mt 2.80, Ta 1.30, total 12.00. Leg IV: Fm 4.00, Pt 1.00, Ti 3.00, Mt 3.00, Ta 1.50, total 12.50.

*Female (sel_225):* Color:carapace light yellow with dusky marks around edges, medially, mediolaterally and around the border of the cephalic area; sternum pale yellow; chelicerae yellow, dusky u-shaped markings present, terminating halfway down; maxillae pale yellow; labium pale yellow; abdomen dorsally cream to yellow, darkening laterally and distally, lanceolate stripe, widening in the middle, making a chevron pattern that extends caudally, several dusky patches laterally, festoon present; ventrally light grey; legs yellowish, faint annulations on femora, patellae and tibiae, duskier patches on metatarsi and tibiae retrolaterally on legs, particularly prominent on legs III and IV. Carapace: 0.99 times longer than broad; fovea longitudinal, broad, very shallow. Eyes:AER slightly recurved; PER lightly recurved; PME larger than AME, PME same as PLE, ALE smallest; eye diameters, AME 0.13, ALE 0.05, PME 0.25, PLE 0.25; interdistances AME-PME 0.08, PME-ALE 0.16, ALE-PLE 0.30. PME-PME 0.96. ALE-ALE 1.60; ocular quadrangle AME-AME 0.35, PLE-PLE 1.78; clypeus 0.10 high. Mouthparts:chelicerae with stout setae medially and anteriorly; maxillae longer than broad, with tuft of conspicuous setae distally; labium distally rounded. Sternum:0.98 times longer than broad, posteriorly indented. Legs:leg I only slightly shorter than legs II, III and IV; leg formula 2431; scopulae present on all 4 tarsi, metatarsi and tibia I and II; tarsi I-IV with strong claw tufts; pr claw per foot slightly toothed; spination: leg I, Fm pr 1–1–0, d 1–1–1, rl 0–1–1; Ti d 0, v 2–2–2; Mt v 2–2; leg II, Fm pr 1–0–0, d 1–1–1, rl 0–0–1; Ti v 2–2–2; Mt v 2–2; leg III, Fm pr 1–0–0, d 1–1–1, rl, 0–0–1; Ti v 1–1–0; Mt v 2–1; leg IV, Fm pr 1–0–0, d 1–1–1, rl 0–0–1; Ti v 1–1; Mt v 2–1. Abdomen:with terminal tufts of setae. Pedipalp:claw present with 9 teeth. Epigyne:lateral lobes distinct, coming together medially, then separating, central depression present, genital openings located medially behind lateral lobes, epigynal pockets present; internally, small posterodorsal fold covering part of internal ducts (Figs 9–10). *Dimensions:* Total length 7.45. Carapace length 3.75, width 3.80. Sternum length 1.80, width 1.75. Abdomen length 3.70, width 3.10. Pedipalp: Fm 1.80, Pt 0.50, Ti 0.50, Ta 1.00, total 3.80. Leg I: femur 3.50, patella 1.50, Ti 2.75, Mt 2.00, Ta 1.00, total 10.75. Leg II: Fm 3.80, Pt 1.50, Ti 3.50, Mt 2.50, Ta 1.00, total 12.30. Leg III: Fm 3.87, Pt 1.25, Ti 3.00, Mt 2.00, Ta 1.00, total 11.12. Leg IV: Fm 3.75, Pt 1.00, Ti 3.00, Mt 2.50, Ta 1.00, total 11.25.

#### Natural history.

Found under bark of *Bursera*, guava, and bay, on palms, and on bromeliads, as well as under rocks in wet and dry forests. The female guards her white, disc-shaped egg sac (Fig. 178).

#### Distribution.

**Map 2.**
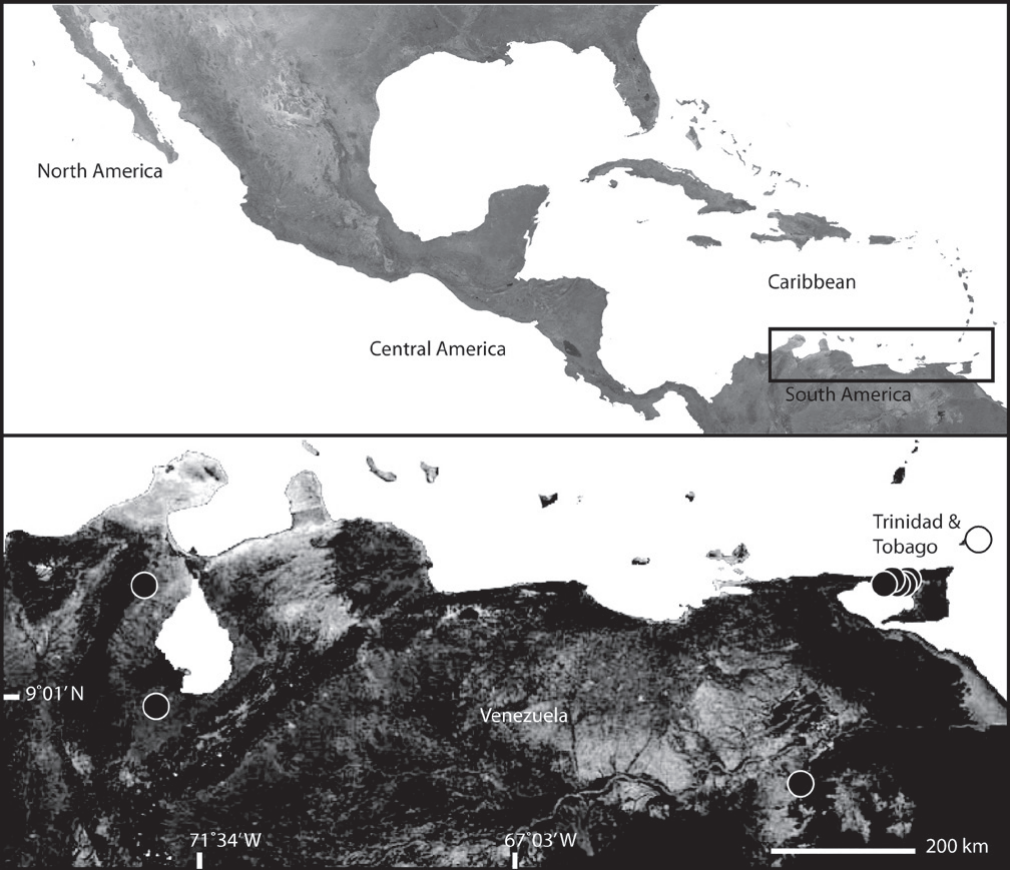
Map of Venezuela and Trinidad and Tobago (inset) showing the distribution of *Selenops*. *Selenops geraldinae* Corronca (black circles), *Selenops willinki* Corronca (white circle).

Mara and Bolívar in Venezuela, as well as Trinidad, including Gaspar Grande, Monos, Huevos and Chacachacare Islands (Map 2).

### Selenops willinki

Corronca, 1998

http://species-id.net/wiki/Selenops_willinki

Figs 11–14 179 197 Map 2

Selenops willinki Corronca 1998: 146, Fig. 69 (♂ holotype from Port of Spain, Goodwar Park, Trinidad (AMNH), not available for examination).

#### Note.

The male holotype of *Selenops willinki* Corronca, 1998, is stated to be deposited in the AMNH (Corronca 1998: 146) but the specimen has not arrived at AMNH. Nevertheless, the description and illustrations are adequate to recognize the specimens described and listed below as this species.

#### Material examined. 

TRINIDAD AND TOBAGO: Little Tobago:
11°18'03.7"N, 60°30'11.1"W, ~63 m, 16.VII.2005, S. Crews, C. Chaboo, C. Barr, B. Shepard, left trail along ridge, SCC05_041 4♀, 2♂, 18 imm. (CAS sel_230-236, 238, 240, 242-243, 245, 251-253, 255-256, 258-259, 261-263).

#### Diagnosis.

Males can be distinguished from other species by the sclerites which obscure the cymbium laterally, and the RTA is directed lateroventrally, and the very small, finger-like, distally directed MA (Figs 11–12). Females can be distinguished from all other species by the anteriorly located genital openings, and medially located epigynal pockets (Figs 13–14).

#### Remarks.

The female of *Selenops willinki* is described here for the first time. Corronca (1998) associated the female of *Selenops geraldinae* with this species. Additional collecting and molecular genetic data (Crews and Gillespie 2010) confirmed that the male and female described here as *Selenops willinki* are conspecific.

#### Description.

*Male sel_251*: Color:carapace yellow, dusky around edges, fovea and mediolaterally; sternum pale yellow; chelicerae yellow, dusky u-shaped markings terminating halfway down; maxillae pale yellow; labium pale yellow; abdomen dorsally light dusky yellow, becoming more orange 3/4 way down, 3 markings at the top, one in the middle and 2 on the sides, a pair of dots in the anterior 1/4, a pair located medially and some specks below and on the sides, edges dark and festoon present; ventrally yellowish, dark lateroposteriorly; legs light yellow, darkening slightly distally with light annulations visible. Carapace: 1.01 times longer than broad; fovea longitudinal, broad, very shallow. Eyes:AER slightly recurved; PER slightly recurved; PME larger than AME, PME same as PLE, ALE smallest; eye diameters, AME 0.20, ALE 0.05, PME 0.30, PLE 0.30; interdistances AME-PME 0.08, PME-ALE 0.08, ALE-PLE 0.35. PME-PME 1.95. ALE-ALE 1.58; ocular quadrangle AME-AME 0.48, PLE-PLE 1.78; clypeus 0.07 high. Mouthparts:chelicerae with a few stout setae medially and anteriorly; maxillae longer than broad, with tuft of conspicuous setae distally; labium distally rounded. Sternum:1.17 times longer than broad, posteriorly indented. Legs:leg I only slightly shorter than leg III; leg formula 3214; scopulae present on distal end of all 4 tarsi; tarsi I-IV with strong claw tufts; pr claw per foot slightly toothed; spination: leg I, Fm pr 1–1–1, d 1–1–1, rl 1–1–0; Ti d 1–1–0, pr 1–0–1, rl 1–0–1, v 2–2–2; Mt v 2–2; leg II, Fm pr 1–1–1, d 1–1–1, rl 1–1–1; Ti pr 1–0–1, d 1–1–0, rl 1–0–1, v 2–2–2; Mt v 2–2; leg III, Fm pr 1–1–1, d 1–1–1, rl 1–1–1–1; Ti pr 1–0–1, d 1–0–0, rl 1–0–1, v 2–2; Mt pr 0, rl 1–1–0, v 2–1; leg IV, Fm pr 1–1–0, d 1–1–1, rl 1–1–1; Ti pr 1–0–1, rl 1–0–1, d 1–0–0, v 2–2; Mt pr 1–1–0, rl 1–1–0, v 2–1. Abdomen:without terminal setal tufts. Pedipalp:Fm, spination dorsal 0–1–4; cymbium triangular in ventral view, angled posterolaterally; scopulae scattered, denser distally; conductor large, angular, arising lateromedially on bulb, c-shaped laterally, truncate and sinuate distally; embolus short, sickle-like, arising at 4 o'clock, ending at 10 o'clock, very stout basally, tapering distally, though fairly stout throughout; MA finger-like, wide basally, curved anteriorly, located at 2 o'clock, directed ventrally; RTA barely reaching cymbium in ventral view, 2 apophyses, ventral apophysis small, curved ventrally, dorsal apophysis very wide, angular (Figs 11–12). *Dimensions:* Total length 7.78. Carapace length 4.00, width 4.03. Sternum length 1.75, width 1.50. Abdomen length 3.78, width 2.78. Pedipalp: Fm 1.00, Pt 0.50, Ti 0.80, Ta 1.00, total 3.30. Leg I: Fm 3.90, Pt 1.50, Ti 3.50, Mt 3.25, Ta 1.65, total 13.80. Leg II: Fm 4.00, Pt 1.50, Ti 3.75, Mt 3.50, Ta 1.55, total 14.20. Leg III: Fm 4.65, Pt 1.50, Ti 3.75, Mt 3.50, Ta 1.35, total 14.75. Leg IV: Fm 4.50, Pt 1.00, Ti 3.65, Mt 3.40, Ta 1.00, total 13.55.

*Female* (sel_236): Color:carapace yellow-orange, 4 dusky spots on each side of carapace, the first, closest to the eyes, is black, dusky markings mediolaterally and laterally from the cephalic area to the posterior end of the cephalothorax; sternum yellow, darker around border; chelicerae same color as cephalothorax with dark marks anterolaterally; maxillae pale yellow, lighter distally; labium dusky yellow, lightening distally; abdomen dorsally cream-colored with 3 dark spots at top, medial dark stripe, 2 dark patches posteriorly, and a prominent festoon, and many dark splotches; ventrally cream-colored; legs orange to yellow, darkening slightly distally, with annulations becoming more prominent distally from patella to tibia. Carapace: 0.97 times longer than broad; fovea longitudinal, broad, very shallow. Eyes:AER slightly recurved; PER slightly recurved; PME larger than AME, PLE largest, ALE smallest; eye diameters, AME 0.15, ALE 0.08, PME 0.28, PLE 0.30; interdistances AME-PME 0.08, PME-ALE 0.10, ALE-PLE 0.30. PME-PME 1.00. ALE-ALE 1.75; ocular quadrangle AME-AME 0.40, PLE-PLE 1.90; clypeus 0.11 high. Mouthparts:chelicerae with a few stout setae medially and anteriorly; maxillae longer than broad, with tuft of conspicuous setae distally; labium distally rounded. Sternum: 1.06 times longer than broad, posteriorly indented. Legs:leg I only slightly shorter than legs II, III and IV; leg formula 3241; scopulae present on all 4 tarsi and metatarsi and tibiae I and II; tarsi I-IV with strong claw tufts; pr claw per foot slightly toothed; spination: leg I, Fm pr 1–1–0, d 1–1–1, rl 1–0–1; Ti d 0, v 2–2–2; Mt v 2–2; leg II, Fm pr 1–0–0, d 1–1–1, rl 1–0–1; Ti v 2–2–2; Mt v 2–2; leg III, Fm pr 1–0–0, d 1–1–1, rl 1–0–1; Ti v 2–2–0; Mt v 2–1; leg IV, Fm pr 0, d 1–1–1, rl 0; Ti v 2–1; Mt v 2–1. Abdomen:with terminal setal tufts. Pedipalp:claw present with 9 teeth. Epigyne:lateral lobes inconspicuous, sinuate ridge in anterior third of epigynal plate, with anteriorly projecting median portion, genital openings located at lateral margins of sinuate ridge, epigynal pockets located medially; internally, ducts long, extending laterally, beginning anteromedially, terminating posteriorly, sinuous, fertilization ducts located posteriorly, small posterodorsal fold present, covering posterior portion of internal ducts (Figs 13–14). *Dimensions:* Total length 8.53. Cephalothorax length 3.90, width 4.03. Sternum length 1.80, width 1.70. Abdomen length 4.63, width 4.08. Pedipalp: Fm 1.00, Pt 0.50, Ti 0.50, Ta 1.00, total 3.00. Leg I: Fm 3.50, Pt 1.60, Ti 3.00, Mt 2.50, Ta 1.00, total 11.60. Leg II: Fm 4.00, Pt 1.50, Ti 3.50, Mt 2.75, Ta 1.00, total 12.75. Leg III: Fm 4.00, Pt 1.40, Ti 3.50, Mt 3.50, Ta 1.00, total 13.40. Leg IV: Fm 4.00, Pt 1.00, Tj 3.00, Mt 2.75, Ta 0.87, total 11.62.

#### Natural history.

Found under the bark of *Bursera* and *Diaspyros* as well as on *Sabal* (Figs 179, 197).

#### Distribution.

Little Tobago and Port of Spain in Trinidad and Tobago (Map 2).

### Selenops banksi

Muma, 1953

http://species-id.net/wiki/Selenops_banksi

Figs 15–16 Map 3

Selenops banksi Muma 1953: 38, Figs 61–63 (♂, examined).

#### Type material.

Holotype male: Barro Colorado, Canal Zone, Panamá, 26.VII(no year), N. Banks, (MCZ, examined). Paratypes: Male, same data as holotype (MCZ).

#### Other material examined.

PANAMÁ: Canal Zone: Barro Colorado Island, II.2008, R. Duncan, 2 imm. (EME sel_1000-1001); **Juan Mina**, II.1945, C.D. Michener, 1 imm. (AMNH); **Panama City:** monsoon forest canopy fogging, 15-30.VII.1979, E. Brodhead et al., 1♂ (USNM). **GUYANA: Iwokrama Forest Research Station:** 1 km north of Kurupukari, canopy fog of *Mora* tree, 14-19.I.1996, W. Tschinkel, 1♂ (CAS). **PERU: Madre de Dios:** Rio Tambopata Reserve, 30 km (air) southwest Puerto Maldonato, 290 m, 12°50'S, 69°20'W, Smithsonian Institute Canopy Fogging Project, 12.VII.1984, T.L. Erwin et al., 1♂ (MCZ).

#### Diagnosis.

**Figures 15–20.**
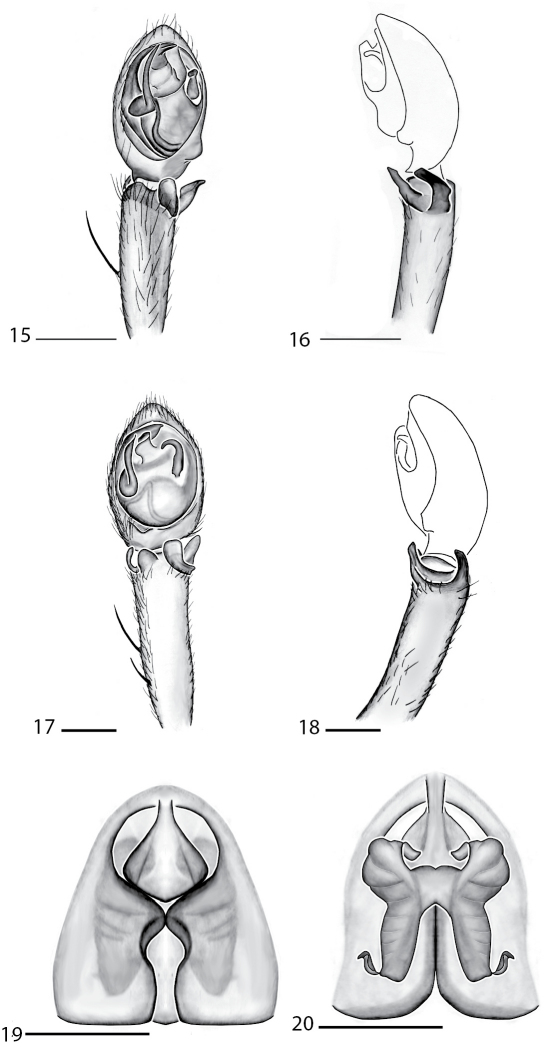
Copulatory organs of *Selenops banksi* Muma male from Barro Colorado Island, Panamá (MCZ) **15–16** *Selenops micropalpus* Muma holotype male from Laudat, Dominica (AMNH) **17–18** female from Laudat, Dominica (AMNH) **19–20, 15, 17** male pedipalp, ventral view **16, 18** male pedipalp, retrolateral view **19** epigyne, ventral view **20** spermathecae, dorsal view. Scale bar = 0.60 mm (15–18), 0.45 mm (19–20).

This species can be distinguished from all others by its yellowish and white abdomen with a darker foliate pattern, in addition to genitalic characteristics. The copulatory organs are most similar to *Selenops micropalpus* in that the palpal tibia is elongated, the embolus is short, and the RTA is small. These two species can be differentiated by the shape of the MA. In *Selenops banksi* the MA is conical with a very small, short, rounded distal hook (Figs 15–16). The dorsal branch of the RTA is also directed ventrally. Females unknown.

#### Description.

*Holotype male:* Color:carapace (holotype) brown-orange, with white setae, (recent) light dusky yellow with carapace and legs slightly darker; sternum (holotype) light yellow, (recent) light dusky yellow; chelicerae (holotype) orange-brown, (recent) dusky yellow, darker on sides; maxillae (holotype) light orange-brown, lightening distally, (recent) dusky yellow; labium (holotype) orange-brown, (recent) brown, lightening distally; abdomen dorsally (holotype) near white to dusky white with a narrow foliate basal lanceolate stripe, one or two interrupted dusky caudal chevrons and interrupted dusky remnants of the usual laterocaudal festoon, (recent) dusky yellow with white outlining lanceolate, foliate stripe, which begins anteriorly and terminates 1/4 way from the posterior end; ventrally light yellow; legs dusky yellow with annulations, leg bands indistinct except on the anteroventral faces of the femora, patellae and tibiae giving the impression of longitudinal stripes. Carapace: 0.87 times longer than broad. Eyes:AER slightly recurved; PER recurved; AME slightly larger than PME, PLE largest, ALE smallest; eye diameters, AME 0.23, ALE 0.08, PME 0.18, PLE 0.28; interdistances AME-PME 0.10, PME-ALE 2.50, ALE-PLE 2.25. PME-PME 0.95. ALE-ALE 1.53; ocular quadrangle AME-AME 0.43, PLE-PLE 1.53; clypeus 0.20 high. Mouthparts:chelicerae with a few stout setae medially and anteriorly; maxillae longer than broad, with tuft of conspicuous setae distally; labium distally rounded. Sternum:1.14 times longer than broad, posteriorly indented. Legs: leg I only slightly shorter than legs II, III and IV; leg formula 3241; scopulae present on distal end of all 4 tarsi; tarsi I-IV with strong claw tufts; pr claw per foot slightly toothed; spination: leg I, Fm pr 1–1–1, d 1–1–1, rl 1–1–1; Ti pr 1–1–0, d 1–1–0, rl 1–1–0, v 1–1–1–1–1; Mt pr 1–1–0, rl 1–1–0, v 2–2; leg II, Fm pr 0–1–1, d 1–1–1, rl 0–1–1; Ti pr 1–0–1, rl 1–0–1, v 2–2–2; Mt pr 1–1–0, v 2–2, rl 1–0–0; leg III, Fm pr 0–1–1, rl 0–1–1, d 1–1–1; Ti pr 1–0–1, rl 1–0–1, v 2–2; Mt pr 1–1–0, rl 1–1–0, v 2–2; leg IV, Fm pr 0–1–1, d 1–1–1, v 0–1–1; Ti pr 1–1–0, rl 1–1–0, v 2–1–0; Mt pr 1–1–1, rl 1–1–0, v 2–0–1. Abdomen:with terminal setal tufts. Pedipalp:Fm, spination dorsal 0–1–3; cymbium oval and slightly angled posterolaterally in ventral view; conductor stout, triangular structure arising distally; embolus very short, tapering suddenly from flat circular base, beginning at 9 o'clock, terminating at 12 o'clock; MA small, conical, slightly curved distally, forming a small hook, located at 2 o'clock position; RTA with two apophyses, both directed ventrally in lateral view, ventral one rounded distally in ventral view, lateral apophysis bent, quadrate, distally truncate, RTA barely reaching cymbium in ventral view; palpal tibia noticeably elongate (Figs 15–16). *Dimensions:* Total length 7.55. Carapace length 3.48, width 3.98. Sternum length 2.00, width 1.75. Pedipalp: Fm 1.75, Pt 0.60, Ti 1.00, Ta 0.90, total 4.25. Leg I: Fm 5.00, Pt 1.75, Ti 5.00, Mt 4.75, Ta 2.00, total 17.50. Leg II: Fm 5.75, Pt 1.75, Ti 5.50, Mt 4.75, Ta 2.00, total 19.75. Leg III: Fm 6.50, Pt 1.60, Ti 5.35, Mt 4.80, Ta 2.10, total 20.35. Leg IV: Fm 6.25, Pt 1.00, tibia 4.65, Mt 4.65, Ta 1.75, total 18.30.

#### Natural history.

This species, at least as an adult male, appears to live in the canopy, as it has only been collected by fogging. It is seemingly widespread, but has only rarely been collected. Juveniles have been collected from under bark.

#### Distribution.

**Map 3.**
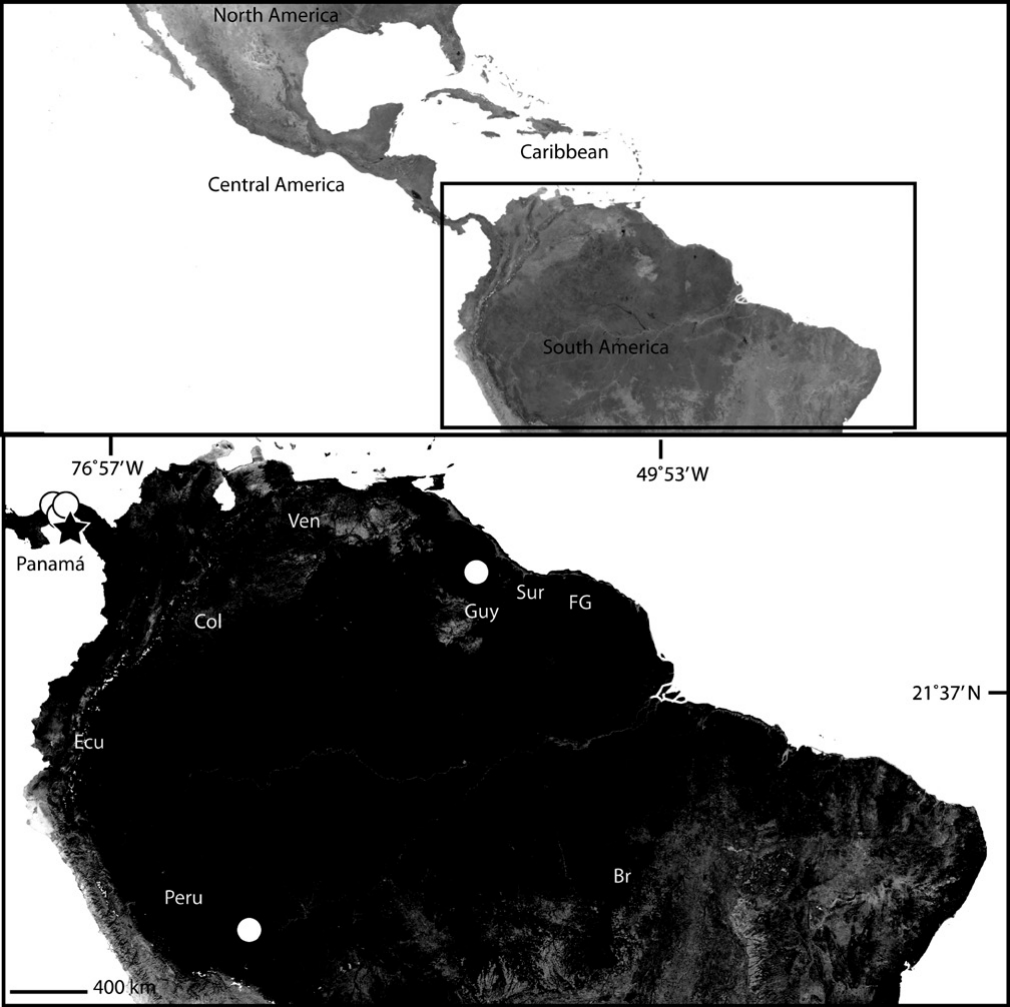
Map of Panamá and South America (inset) showing the distribution of *Selenops banksi* Muma (white circles) and *Selenops buscki* Muma (black star). (Col = Colombia, Ecu = Ecuador, Ven = Venezuela, Guy = Guyana, Sur = Surinam, FG = French Guyana, Br = Brazil).

Occurs from Panamá, south to Peru and east to Guyana (Map 3).

### Selenops micropalpus

Muma, 1953

http://species-id.net/wiki/Selenops_micropalpus

**Map 4.**
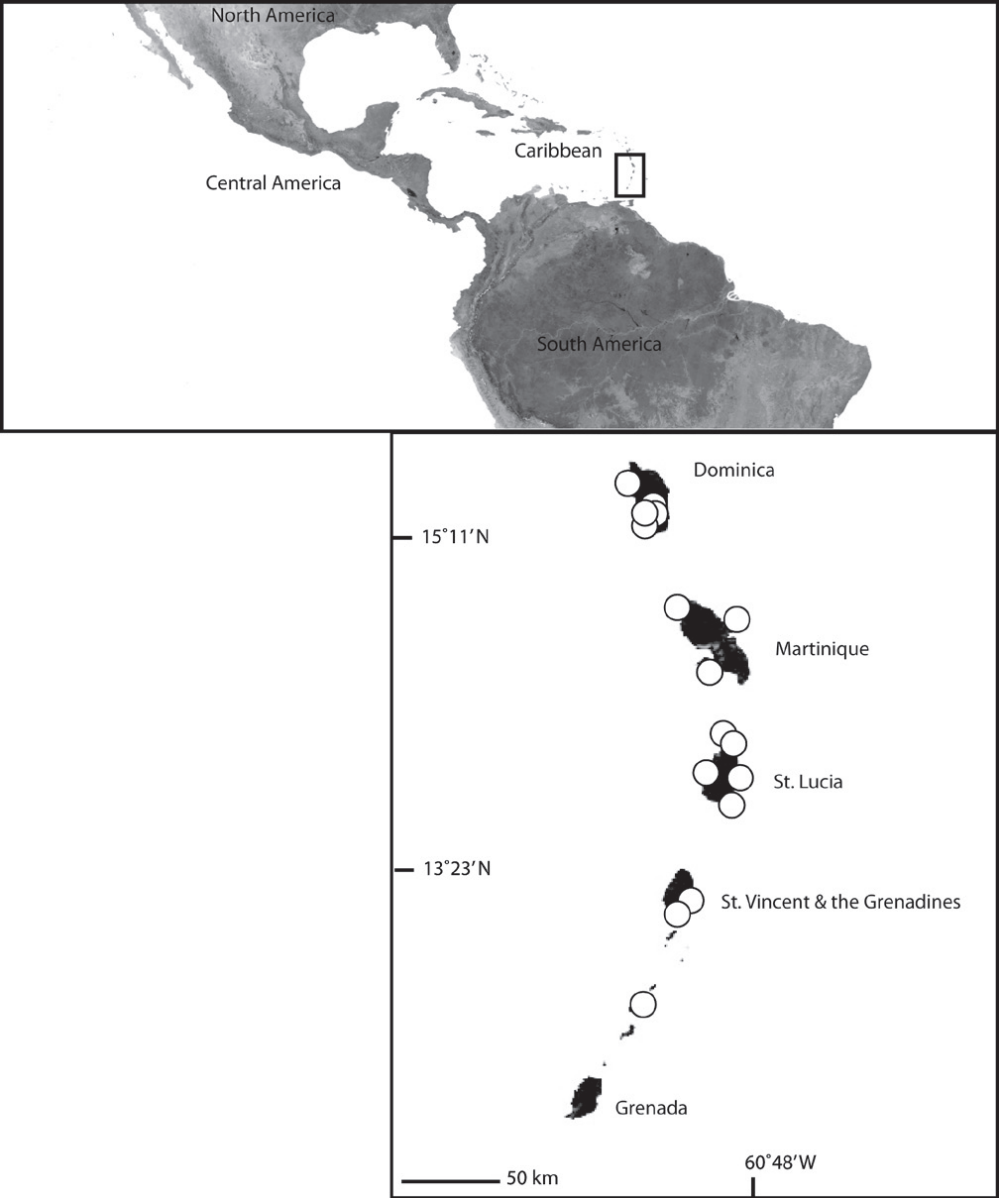
Map of Dominica, Martinique, St. Lucia, St. Vincent and the Grenadines and Grenada (inset) showing the distribution of *Selenops micropalpus* Muma (white circles).

Figs 17–20 180 Map 4

Selenops micropalpus Muma 1953: 39, Figs 66–68 (♂, ♀, examined).

#### Type material.

Holotype male: Laudat, Dominica, 13.VII.1911, F.E. Lutz, (AMNH, examined). Paratypes: Same data as holotype (AMNH, examined).

#### Other material examined.

DOMINICA: Long Ditton: 19.VII.1911, F.E. Lutz, 1♀ (AMNH); **Roseau:** 20.VII.1911, F.E. Lutz, 1 imm. (AMNH); Botanical Park, top of hill near shrine, 15°17.998'N, 61°22.754'W, 63 m, 1.XI.2004, S. Crews, under bark, SCC04_059, 5 imm. (EME sel_097-101). **Cabrits National Park:** trail to fort, 15°35.049'N, 61°28.371'W, 40 m, 2.XI.2004, S. Crews, A. James, S. Toussaint, under bark of *Bursera*, *Mastichodendron foetidissimum*, Campeche, mahogany, several egg sacs present, SCC04_060, 2♀, 4 imm. (EME sel_106-108, 110-111, 135). **Jimmit-Warner:** on top of hill with cell phone tower, 15°22.690'N, 61°24.003'W, 54 m, 2.XI.2004, S. Crews, A. James, S. Toussaint, dry transitional rainforest, under bark of bay, lagawenn, SCC04_061, 3 imm. (CAS sel_102-105). **MARTINIQUE: La Caravelle Reservé** **Naturelle:** trail to Pointe Caricoli, 14°46'09.3"N, 60°53'24.7"W, 8.III.2007, S. Crews, Nouree-Yvon, under bark, several egg sacs, guarded by female, SCC07_041, 3♀, 1♂, 3 imm. (EME sel_791-797). **Anse Céran:** off of road D-10, 14°50'01.5"N, 61°13'24.7"W, 9.III.2007, S. Crews, Nouree-Yvon, dry forest, under bark, SCC07_042, 2♀, 3♂, 1p♂ (CAS sel_798-803). **Le Diamant:** Grande Anse du Diamant, off road D37, 14°28'32.9"N, 61°02'13.4"W, sea level, 10.III.2007, S. Crews, D. Memia Zolo, dry forest, under bark of *Cocoloba*, SCC07_043, 4♀, 3 imm. (CAS sel_804-810). **ST. LUCIA:** **Gros Islet:** Pigeon Island, top of hill above construction, 14°05'31.3"N, 60°57'03.8"W, ~30 m, 12.III.2007, S. Crews, G. George, under bark of *Bursera*, SCC07_044, 4 imm. (EME sel_811-814). **Beausejour:** near Gros Islet, past cricket sponsor's office, 14°04'43.1"N, 60°56'31.1"W, ~60 m, 12.III.2007, S. Crews, G. George, under bark of *Bursera*, SCC07_045, 3♀, 2 imm. (EME sel_815-819). **Anse La Raye:** Ti-Kaye, 13°55'29.6"N, 61°02'41.3"W, ~100 m, 13.III.2007, S. Crews, G. George, on banana plants, SCC07_046, 1p♂, 3 imm. (CAS sel_820-823). **Dennery:** on road from Dennery south on East Coast Road, Eastern Nature Trail, Heritage Tourism Site, 13°53'50.8"N, 60°52'51.2"W, ~35 m, 13.III.2007, S. Crews, G. George, dry coastal forest, under bark of *Bursera* and sea grape, SCC07_047, 3♀, 1♂, 4 imm. (CAS sel_824-831); **Vieux Fort:** on hillside above airport, 13°44'20.2"N, 60°56'40.8"W, ~40 m, 13.III.2007, S. Crews, G. George, under bark of *Bursera*, SCC07_048, 1♀, 2 imm. (EME sel_832-834). **ST. VINCENT: Young Island:** south of Villa Beach, 13°07.895'N, 61°12.142'W, ~10 m, 24.X.2004, S. Crews, G. Alayón-García, under bark, SCC04_053, 1♀, 1 imm. (EME sel_091, 113). **King's Hill Forest Reserve:** 13°08.825'N, 61°10.021'W, ~202 m, 27.X.2004, S. Crews, G. Alayón-García, M. da Silva, under bark of *Bursera*, 1♂, 6 imm. (CAS sel_088, 090, 092-095, 112).

#### Diagnosis.

Males can be distinguished from all other species by the very long palpal tibia, the very short embolus, the small RTA and the distal hook of the MA being almost as long as the rest of the MA (Figs 17–18). In the female, the epigynum has an anteriorly located diamond-shaped septum (Figs 19–20).

#### Description.

*Holotype male*: Color: carapace (holotype) orange-brown, (recent) yellow-brown with darker areas; sternum (holotype) orange-yellow, (recent) pale yellow, darker around border; chelicerae (holotype) orange-brown, slightly darker laterally, (recent) brown, darker laterally; maxillae orange-brown, lightening distally; labium (type) orange-brown, lightening distally; abdomen dorsally (holotype) cream to tan, faint indications of festoon, (recent) yellow-grey with dark lines, darker caudally, festoon present; ventrally (holotype) dusky yellow, (recent) yellow; legs (holotype) orange-brown, (recent) yellowish, annulations slightly apparent, metatarsal annulations fused, entire metatarsus dark. Carapace: 0.90 times longer than broad. Eyes:AER nearly straight; PER slightly recurved; PME same size as AME, PLE largest, ALE smallest; eye diameters, AME 0.30, ALE 0.16, PME 0.30, PLE 0.40; interdistances AME-PME 0.08, PME-ALE 0.15, ALE-PLE 0.30. PME-PME 1.40. ALE-ALE 2.30; ocular quadrangle AME-AME 0.53, PLE-PLE 2.40; clypeus 0.08 high. Mouthparts:chelicerae with a few stout setae medially and anteriorly; maxillae longer than broad, with tuft of conspicuous setae distally; labium distally rounded. Sternum:1.06 times longer than broad, posteriorly indented. Legs:all legs much shorter than leg II; leg formula 2314; scopulae present on distal end of all 4 tarsi; tarsi I-IV with strong claw tufts; pr claw per foot slightly toothed; spination: leg I, Fm pr 1–1–1, d 1–1–1, rl 1–1–1; Ti pr 1–0–1, rl 1–0–1, d 1–1–0, v 2–2–2; Mt pr 1–1–0, rl 1–1–0, v 2–2; leg II, Fm pr 1–1–1, d 1–1–1, rl 1–1–1; Ti pr 1–0–1, d 1–1–0, rl 1–0–1, v 2–2–2; Mt pr 1–1–0, v 2–2, rl 1–1–0; leg III, Fm pr 1–1–1, d 1–1–1, rl 1–1–1; Ti pr 1–0–1, d 1–0–0, rl 1–0–0, v 2–2; Mt pr 1–1–0, rl 1–1–0, v 2–2; leg IV, Fm pr 1–1–1, d 1–1–1, rl 1–1–1; Ti pr 1–0–1, d 1–1–0, rl 1–0–1, v 2–2–1; Mt pr 1–1–0, rl 1–1–0, v 2–2. Abdomen:with terminal setal tufts. Pedipalp:Fm, spination d 0–1–3; cymbium oval in ventral view; conductor stout structure arising distally, slightly truncate distally; embolus very short, tapers abruptly from flat circular base, beginning at 9 o'clock, terminating at 12 o'clock; MA arising at 3 o'clock, directed anterolateraly, finger-like, curved forming long hook distally; RTA small with two processes, both slightly curved and distally truncate; tibial apophyses barely reaching cymbium in ventral view (Figs 17–18). *Dimensions:* Total length 10.50. Carapace length 5.20, width 5.75. Sternum length 2.65, width 2.50. Abdomen length 5.30, width 3.85. Pedipalp: Fm 2.50, Pt 0.75, Ti 1.75, Ta 1.00, total 6.00. Leg I: Fm 4.75, Pt 1.50, Ti 4.00, Mt 4.00, Ta 1.75, total 16.00. Leg II: Fm 5.75, Pt 2.00, Ti 5.00, Mt 4.50, Ta 2.00, total 19.25. Leg III: Fm 5.50, Pt 1.00, Ti 4.75, Mt 3.75, Ta 1.25, total 15.15. Leg IV: Fm 4.75, Pt 1.10, Ti 3.75, Mt 3.75, Ta 1.65, total 15.00.

*Paratype female*: Color:carapace (paratype) orange-brown, (recent) light yellow-brown to light orange-brown to dark brown, darker laterally, mediolateral stripes from cephalic area to posterior end of cephalothorax, and sides of cephalic area; sternum (paratype) orange-brown, (recent) light yellow; chelicerae (paratype) red-orange, darker laterally, (recent) light brown medially, dark brown laterally; maxillae (paratype) orange-brown, lightening distally, (recent) light brown, lightening distally; labium (paratype) red-brown, (recent) brown; abdomen dorsally (paratype) brownish, more yellow medially, festoon barely visible (recent) light greyish with two chevrons at top of abdomen, some darker flecks, festoon present; ventrally grey to brown with a pair of narrow stripes; legs (paratype) yellow-orange with orange-brown annulations, (recent) yellow-brown with annulations, metatarsal annulations fused, metatarsi dark. Carapace:0.90 times longer than broad. Eyes:AER nearly straight; PER slightly recurved; PME larger than AME, PLE largest, ALE smallest; eye diameters, AME 0.23, ALE 0.08, PME 0.30, PLE 0.38; interdistances AME-PME 0.13, PME-ALE 0.23, ALE-PLE 0.55. PME-PME 1.70. ALE-ALE 2.83; ocular quadrangle AME-AME 0.63, PLE-PLE 2.90; clypeus 0.06 high. Mouthparts:chelicerae with stout setae medially and anteriorly; maxillae longer than broad, with tuft of conspicuous setae distally; labium distally rounded. Sternum:1.40 times longer than broad, posteriorly indented. Legs:leg I only slightly shorter than legs II, III and IV; leg formula 2431; scopulae present on tarsi of all legs and metatarsi of legs I and II; tarsi I-IV with strong claw tufts; pr claw per foot slightly toothed; spination: leg I, Fm pr 1–1–0, d 1–1–1, rl 1–1–1; Ti v 2–2–2; Mt v 2–2; leg II, Fm pr 1–0–0, d 1–1–1, rl 1–0–1; Ti v 2–2–2; Mt v 2–2; leg III, Fm pr 1–0–0, d 1–1–1, rl, 0–0–1; Ti v 2–2–0; Mt v 2–2; leg IV, Fm pr 1–1–0, d 1–1–1, rl 0–0–1; Ti v 2–2–0; Mt v 2–1. Abdomen:with terminal setal tufts. Pedipalp:claw present with 9 teeth. Epigyne:lateral lobes conspicuous, coming together medially, then curving out, diamond-shaped septum present anteriorly, genital openings located anterolateraly at septum, epigynal pockets located medially; internally, ducts appear slightly coiled, extending posteriorly, fertilization ducts located posteriorly, posterodorsal fold absent (Figs 19–20). *Dimensions:* Total length 13.45. Carapace length 6.33, width 7.00. Sternum length 3.50, width 2.50. Abdomen length 7.13, width 4.24. Pedipalp: Fm 2.00, Pt 1.00, Ti 1.00, Ta 1.90, total 5.90. Leg I: Fm 6.50, Pt 2.75, Ti 5.75, Mt 4.75, Ta 1.75, total 21.50. Leg II: Fm 7.00, Pt 2.50, Ti 6.00, Mt 5.00, Ta 1.75, total 22.25. Leg III: Fm 6.75, Pt 2.50, Ti 6.00, Mt 4.75, Ta 1.85, total 21.85. Leg IV: Fm 7.00, Pt 2.50, Ti 5.75, Mt 5.00, Ta 1.75, total 22.00.

#### Natural history.

Found under the bark of various trees in rainforests, transitional rainforests and dry forests (Fig. 180). Some of those trees are *Bursera*, *Cocoloba*, *Mastichodendron foetidissimum*, Campeche, mahogany, bay, and lagawenn. The female guards the flat, disc shaped egg sac.

#### Distribution.

Southern Lesser Antilles from Dominica, south to Mayreau in St. Vincent and the Grenadines (Map 4).

### Selenops aztecus

Valdez-Mondragón, 2010

http://species-id.net/wiki/Selenops_aztecus

Map 5

Selenops aztecus Valdez-Mondragón 2010: 48, Figs 1–4 (♂ holotype male from 14 km east of Coatzacoalcos, Veracruz, toward Villahermosa, Tabasco, México, in CNAN, not examined).

#### Natural history.

This species was found on the bromeliad *Aechmea bracteata* (Valdez-Mondragón 2010).

#### Distribution.

**Map 5.**
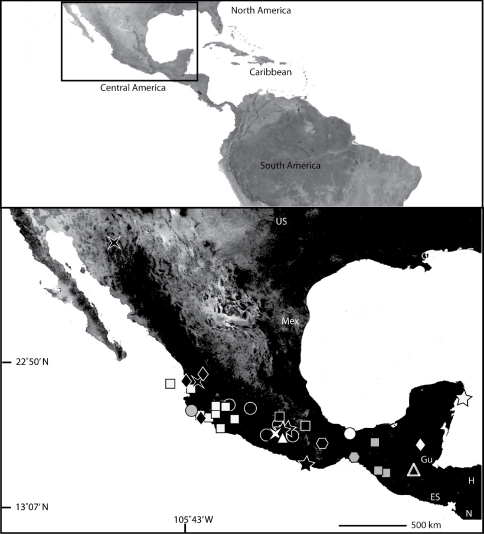
Map of México and Guatemala (inset) showing the distribution of several *Selenops* species in the region. *Selenops aztecus* Valdez-Mondragón (white circle), *Selenops gracilis* Muma (black circles), *Selenops malinalxochitl* sp. n. (black squares), *Selenops abyssus* Muma (white squares), *Selenops juxtlahuaca* Valdez-Mondragón (white triangle), *Selenops marginalis* F.O. Pickard-Cambridge(white X), *Selenops minutus* F.O. Pickard-Cambridge (black triangle), *Selenops morosus* Banks (black Xs), *Selenops nigromaculatus* Keyserling (black hexagon), *Selenops makimaki* sp. n. (grey hexagon), *Selenops scitus* Muma (black stars), *Selenops ixchel* sp. n. (white star), *Selenops petenajtoy* sp. n. (white diamond), *Selenops lepidus* Muma (black diamond), *Selenops chamela* sp. n. (grey circle), *Selenops huetocatl* sp. n. (grey squares). US = United States, Mex = México, Gu = Guatemala, ES = El Salvador, H = Honduras, N = Nicaragua.

Known from the type locality only (Map 5).

### Selenops gracilis

Muma, 1953

http://species-id.net/wiki/Selenops_gracilis

Figs 21–24 Map 5

Selenops gracilis Muma 1953: 10, Figs 12–14 (♂, ♀, examined).

#### Type material.

Holotype male: Ayotzinapa, Guerrero, México, 11.I.1941, (AMNH, examined). Paratypes: Female, Taxco, Guerrero, México, IV.1946, L. Isaacs (AMNH, examined).

#### Other material examined.

MÉXICO: Michoacan: Jiquilpan, 19°59' N, 102°41' W, 9.V.1963, W.J. Gertsch and W. Ivie, ♀s, imm. (AMNH); Janitzio, 13.X.1940, J.A. de Villar, 3♀ (AMNH); **Guerrero:** Taxco, X.1945, L. Isaacs, 2♀ (AMNH); Taxco, 5.VI.1943, F.H. Pough, 1♀ (AMNH); Arcelia, Campo Morado, 2♀ (CNAN sel_1014-1015).

#### Diagnosis.

**Figures 21–28.**
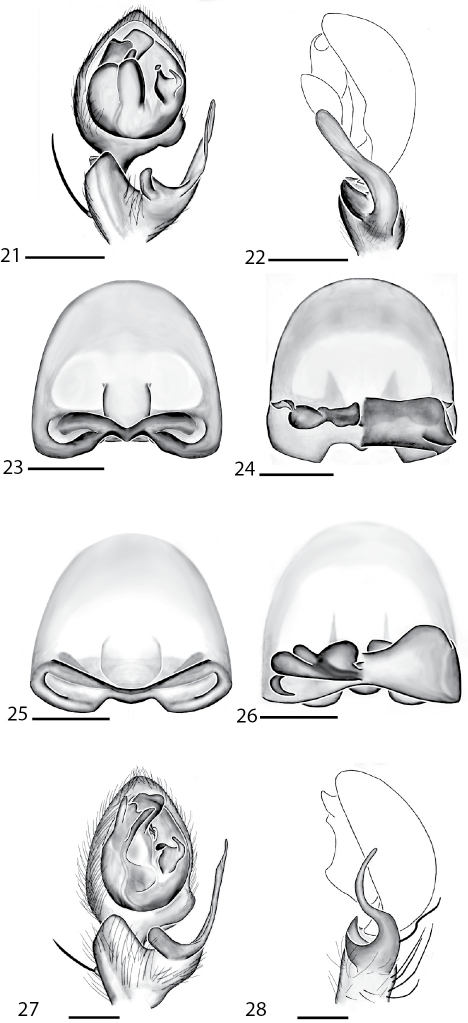
Copulatory organs of *Selenops gracilis* Muma holotype male from Ayotzinapa, Guerrero, México (AMNH) **21–22** female from Taxco, México (AMNH) **23–24** *Selenops malinalxochitl* sp. n. holotype female from Zapotitlan de las Salinas, Puebla, México (CNAN sel_1005) **25–26** *Selenops mexicanus* Keyserling male from Totogalpa, Alcadia Ocotal, Departmiento Madríz,Nicaragua (EME sel_934) **27–28, 21, 27** male pedipalp, ventral view **22, 28** male pedipalp, retrolateral view **23, 25** epigyne, ventral view **24, 26** spermathecae dorsal view. Scale bar = 0.90 mm (21–22), 0.50 mm (23–26).

This species is similar to *Selenops mexicanus*, *Selenops malinalxochitl*, *Selenops ecuadorensis* and *Selenops aztecus*. Males can be easily distinguished by a very short embolus which arises from behind a larger sclerite, and also by the long, distally rounded RTA, (Figs 21–22). Females can be distinguished from other species by a hyaline median septum that goes behind a slightly m-shaped posterior area with lateral indentations, coming to a point medially, and internally, having laterally directed, non-branching internal ducts, with fertilization ducts located and directed laterally, and a rectangular posterodorsal fold (Figs 23–24).

#### Remarks.

There is some variation in the external female copulatory organs. In all specimens viewed thus far, the posterormedial margin is always pointed, and internally, the ducts are all identical, unbranched, with an identically shaped rectangular posterodorsal fold.

#### Description.

*Holotype male*: Color:carapace orange-brown; sternum dusky yellow, darker around border; chelicerae dark red-brown; maxillae brown-yellow, lighter distally; labium orange-brown, lightening toward the distal edge; abdomen dorsally tan to cream with faint anterior lateral u-shaped markings and two w-shaped caudal lines, laterocaudal festoon present; legs orange-brown, markings indistinct; Carapace: 0.93 times longer than broad. Eyes:AER nearly straight; PER recurved; PME larger than AME, PLE largest, ALE smallest; eye diameters, AME 0.25, ALE 0.10, PME 0.28, PLE 0.40; interdistances AME-PME 0.15, PME-ALE 0.10, ALE-PLE 0.55. PME-PME 1.30. ALE-ALE 2.13; ocular quadrangle AME-AME 0.43, PLE-PLE 2.40; clypeus 0.13 high. Mouthparts:chelicerae with a few stout setae medially and anteriorly; maxillae longer than broad, with tuft of conspicuous setae distally; labium distally rounded. Sternum:1.38 times longer than broad, posteriorly indented. Legs:leg I only slightly shorter than legs II, III and IV; leg formula 2341; scopulae present on distal end of all 4 tarsi; tarsi I-IV with strong claw tufts; pr claw toothed, rl claw with fewer teeth; spination: Leg I, Fm pr 1–1–1, d 1–1–1, rl 1–1–1; Leg II, Fm pr 1–1–1, d 1–1–1, rl 1–1–1; Leg III, Fm pr 1–1–1, d 1–1–1, rl 1–1–1; Leg IV, Fm pr 1–1–1, d 1–1–1, rl 1–1–1; Ti pr 1–0–1, v 2–2–0, rl 1–0–1; Mt v 2–2. Abdomen:without terminal setal tufts. Pedipalp:Fm, spination d 0–1–4; cymbium oval in ventral view, slightly angled posterolaterally; conductor quadrate, arising distally; embolus very short, blunt, beginning at 9 o'clock, terminating at 10 o'clock; MA located at 3 o'clock, directed ventrally, with two branches, one bent distally, the other pointed distally; RTA with two branches, ventral apophysis angular, curving toward cymbium, small, dorsal process very long, sinuous in lateral view, distally rounded; RTA extending at least ½ length of cymbium in ventral view (Figs 21–22). *Dimensions:* Total length 10.63. Carapace length 5.00 width 5.40. Sternum length 2.75, width 2.99. Abdomen length 5.63, width 4.15. Pedipalp: Fm 1.25, Pt 0.40, Ti 0.75, Ta 1.40, total 3.70. Leg I: Fm 6.00, Pt 2.00, Ti, Mt and Ta missing. Leg II: Fm 6.75, Pt 2.40, Ti, Mt and Ta missing. Leg III: Fm 7.00, Pt 1.75, Ti, Mt and Ta missing. Leg IV: Fm 7.00, Pt 1.75, Ti 5.8, Mt 5.75, Ta 2.00, total 22.30.

*Paratype female:* Color:carapace (paratype) dark red-brown, (recent) brown to orange-brown, darker in cephalic area, with white setae, and darker patches; sternum (paratype) orange-brown, darker at border, (recent) pale yellow, slightly darker around border; chelicerae (paratype) dark red-brown, (recent) dark brown to red-brown; maxillae (paratype) red-brown lightening distally, (recent) light brown lightening distally; labium (paratype) red-brown, lightening distally, (recent) orange-brown, dusky on sides, lightening distally; abdomen dorsally (paratype) tan to light brown, seemingly discolored medially dark laterocaudal festoon present, (recent) cream to tan background with two darker u-shaped marks anterolaterally and two w-shaped marks posteriorly, lots of dark flecks; ventrally dusky orange-grey; legs (paratype) dusky orange, markings no longer distinct, (recent) dark double bands on femur I, lighter on other legs, fused on tibiae and metatarsi, half of each segment is very dark and very light, tarsi dark, lighter part of each leg yellowish. Carapce: 0.92 times longer than broad. Eyes:AER nearly straight; PER recurved; PME larger than AME, PLE largest, ALE smallest; eye diameters, AME 0.28, ALE 0.13, PME 0.33, PLE 0.50; interdistances AME-PME 0.13, PME-ALE 0.55, ALE-PLE 0.43. PME-PME 1.70. ALE-ALE 3.03; ocular quadrangle AME-AME 0.70, PLE-PLE 3.23; clypeus 0.09 high. Mouthparts:chelicerae with a few stout setae medially and anteriorly; maxillae longer than broad, with tuft of conspicuous setae distally; labium distally rounded. Sternum:as long as broad, posteriorly indented. Legs:leg formula 2413; scopulae present on tarsi of all legs and metatarsi of legs I and II; tarsi I-IV with strong claw tufts; both claws with same number of teeth; spination: leg I, Fm pr 1–1–0, d 1–1–1, rl 1–0–1; Ti d 0, v 2–2–2; Mt v 2–2; II, Fm pr 1–0–0, d 1–1–1, rl 1–0–1; Ti v 2–2–2; Mt v 2–2; III, Fm pr 1–0–0, d 1–1–1, rl 1–0–1; Ti v 2–2–0; Mt v 2–2; IV, Fm pr 1–0–0, d 1–1–1, rl 0–0–1; Ti v 2–2–0; Mt v 1–1. Abdomen:without terminal setal tufts. Pedipalp:claw with 13 teeth. Epigyne:lateral lobes indistinct, hyaline median field with quadrate septum, terminating behind posterior sclerotized m-shaped area, with lateral ridges and indentations, pointed medially, genital openings located posterolaterally at sides of septum, internally, ducts are simple, unbranched and directed laterally, with fertilization ducts located and directed laterally, posterodorsal fold present, strongly sclerotized, quadrate, completely covering internal ducts (Figs 23–24). *Dimensions:* Total length 12.30. Carapace length 6.10, width 6.60. Sternum length 2.75, width 2.75. Abdomen length 6.20, width 4.65. Pedipalp: Fm 1.75, Pt 0.90, Ti 1.00, Ta 1.80, total 5.45. Leg I: Fm 6.00, Pt 2.75, Ti 5.75, Mt 4.75, Ta 1.75, total 21.00. Leg II: Fm 9.00, Pt 3.00, Ti 7.00, Mt 5.50, Ta 1.80, total 26.30. Leg III: Fm 8.00, Pt 1.90, Ti 5.00, Mt 3.80, Ta 1.60, total 20.30. Leg IV: Fm 7.00, Pt 2.25, Ti 6.50, Mt 5.00, Ta 1.70, total 22.45.

#### Natural history.

No data.

#### Distribution.

South central México, from Michoacan to Guerrero (Map 5).

### Selenops malinalxochitl sp. n.

urn:lsid:zoobank.org:act:615D0716-84E7-4053-BB7B-6FAB75270B96

http://species-id.net/wiki/Selenops_malinalxochitl

Figs 25–26 Map 5

Selenops gracilis Muma 1953: 10, figs 12–14 (misidentification of ♀ specimen from Cuernavaca in AMNH, nec *Selenops gracilis* Muma, 1953).

#### Type material.

Holotype female: Mun. Zapotitlan de las Salinas, Puebla, México, 18°22'48.3"N, 97°30'23.9"W, 2473 m, 29.V.2005, U.O.G. Vázquez, under rock (CNAN sel_1005). Paratypes: Female, same data as holotype (CNAN sel_1002).

#### Other material examined.

MÉXICO: Morelos: Cuernavaca, Colonia Chamilpa, University of Morelos, 18°58'45.84"N, 99°'39.17"W, 11.II.2005, 1850 m, U.O.G. Vázquez, on wall at night, 1p♂ (EME sel_1010); Cuernavaca, 1♀ - Muma (1953) placed this specimen in *Selenops gracilis* (AMNH).

#### Etymology.

The specific epithet comes from the Nahua goddess Malinalxochitl, the goddess of desert-dwelling snakes and arthropods, and refers to the type locality and the indigenous Nahua people of this species' distribution. It is to be treated as a noun in apposition.

#### Diagnosis.

This species is most similar to *Selenops gracilis*, but the posterior sclerotized area is u-shaped, with the lateral indentations directed anteriorly, and the posteromedian area of the caudal margin is rounded, rather than pointed (Fig. 25). Internally, the posterodorsal fold is medially depressed and the internal ducts are branched (Fig. 26). Males unknown.

#### Remarks.

As in other closely related species, there is some epigynal variation. The internal ducts and posterodorsal fold are uniform across specimens examined.

#### Description.

*Holotype female:* Color: carapace red-brown, dusky near fovea and laterally; sternum orange-brown, darker around border; chelicerae dark-brown; maxillae orange-brown, lightening distally, darkening on anterolateral border, lightening on anteromedial border; labium orange-brown, lightening distally; abdomen dorsally brownish-grey with several medial chevrons, festoon present; ventrally dusky grey with no markings; legs orange to brown, darkening distally only slightly, annulations visible. Very dark and over half of tibiae, making lighter area appear very light. Cephalothorax: 0.83 times longer than broad; fovea longitudinal, broad, very shallow. Eyes:AER nearly straight; PER recurved; PME larger than AME, PLE largest, ALE smallest; eye diameters, AME 0.20, ALE 0.13, PME 0.30, PLE 0.55; interdistances AME-PME 0.03, PME-ALE 0.25, ALE-PLE 0.48. PME-PME 2.35. ALE-ALE 2.45; ocular quadrangle AME-AME 0.58, PLE-PLE 2.65; clypeus 0.07 high. Mouthparts:chelicerae with stout setae medially and anteriorly; maxillae longer than broad, with tuft of conspicuous setae distally; labium distally rounded. Sternum:1.38 times longer than broad, posteriorly indented. Pedipalp:claw with 12 teeth. Legs:leg I much shorter than legs II, III and IV; leg formula unknown (at least one leg missing); scopulae present on tarsi of all legs and metatarsi of legs I and II; tarsi I-IV with strong claw tufts; pr claw toothed, rl claws with fewer teeth; spination: leg I, Fm pr 1–1–1, d 1–1–1, rl 1–1–1; Ti d 0, v 2–2–2; Mt v 2–2; leg II, Fm pr 1–0–0, d 1–1–1, rl 1–1–1; Ti v 2–2–2; Mt v 2–2; leg III, Fm pr 1–0–0, d 1–1–1, rl 1–0–1; Ti v 2–2–0; Mt v 2–1. Abdomen:without terminal setal tufts. Epigyne:lateral lobes indistinct, hyaline median field with median septum terminating at heavily sclerotized posterior margin, lateral indentations directed anteriorly, posteromedian area u-shaped, genital openings located at posterolateral margins of median septum, internally ducts located posteriorly, directed laterally, branched, large heavily sclerotized posterodorsal fold covering nearly all of the internal ducts, medially depressed, rounded anterolaterally (Figs 25–26). *Dimensions:* Total length 9.33. Carapace length 4.73, width 5.75. Sternum length 2.75, width 2.00. Abdomen length 4.60, width 5.50. Pedipalp: Fm 1.00, Pt 0.70, Ti 0.75, ta 1.45, total 3.90. Leg I: Fm 4.75, Pt 2.00, Ti 4.00, Mt 3.00, Ta 1.00, total 14.75. Leg II: Fm 5.75, Pt 1.50, Ti 4.75, Mt 3.50, Ta 1.50, total 17.00. Leg III: Fm 5.80, Pt 1.75, Ti 4.75, Mt 3.75, Ta 1.00, total 17.05. Leg IV: Missing.

#### Natural history.

Collected under rocks and on the walls of human habitations.

#### Distribution.

The south central Mexican states of Morelos and Puebla (Map 5).

### Selenops mexicanus

Keyserling, 1880

http://species-id.net/wiki/Selenops_mexicanus

**Map 6.**
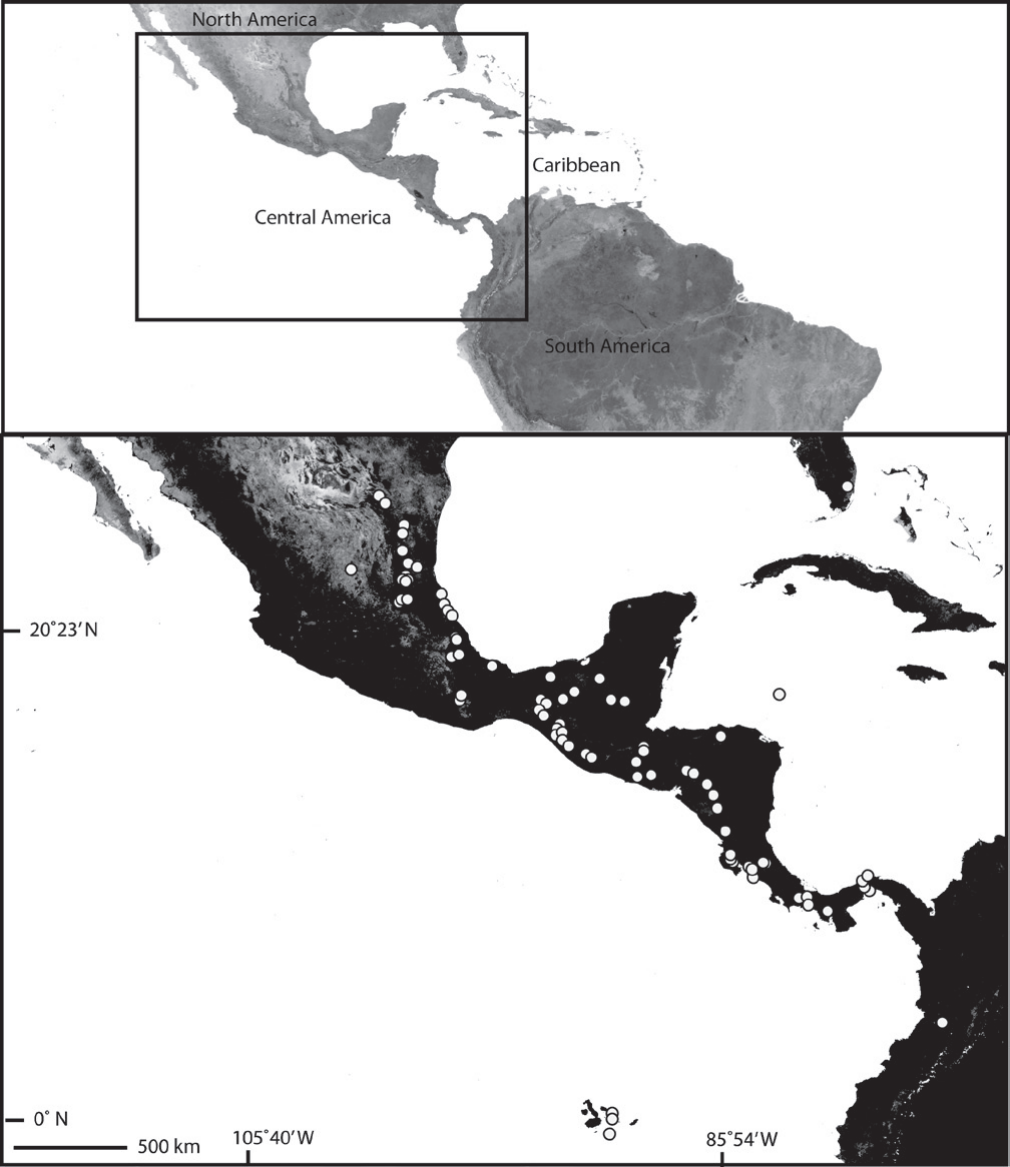
Map of North, Central and northern South America (inset) showing the distribution of *Selenops mexicanus* Keyserling (white circles). This species has also been found in various states in the US where it was imported on produce, as well as in St. Maarten, where it was likely imported on a palm. See text for details.

Figs 27 30 181 Map 6

Selenops mexicanus Keyserling 1880: 228, pl. 6, Fig. 125 (♂, ♀, not examined).
Selenops mexicanus :F.O. Pickard-Cambridge, 1900: 117, pl. 8, Figs 17–18 (♂, ♀).
Selenops aissus :Petrunkevitch 1925: 134, Figs 51–52 (♀).
Selenops mexicanus :Muma 1953: 7, Figs 1–4 (♂, ♀).
Selenops galapagoensis Banks 1902: 63, pl. 1, Fig. 8 (♀, examined), syn. n.*Selenops tehuacanus*Muma 1953: 8, Figs 5–6 (♂, examined), syn. n.*Selenops vagabundus*Kraus 1955: 53, Figs 142–144 (♂, ♀, not examined), syn. n.

#### Type material.

Holotype male (*Selenops mexicanus)*: México (BMNH), lost (see below), not examined. Lectotype female (here designated), México, Keyserling collection, (BMNH 90.41.3158.3100).

#### Notes.

Muma (1953) noted both male and female types as being in the collection of E. Simon, and deposited in the MNHN. Simon's descriptions were redescriptions of Keyserling's, and these are in the BMNH. The specimens from México include 2 females in one vial, and no males. In a second vial, there are a male and a female from San Jose, Costa Rica (BMNH 96.3.20.32–33). The holotype females of *Selenops galapagoensis* Banks, 1902, *Selenops tehuacanus* Muma, 1953 and *Selenops vagabundus* Kraus, 1955 are in every way identical to the lectotype female of *Selenops mexicanus* Keyserling 1880, and thus these species names are hereby new synonymies. See also comment below under ‘Remarks'.

#### Other material examined.

COLOMBIA: Valle: Cali, 1000 m, 1973–1974, W. Eberhard, 1♂ (MCZ). **COSTA RICA:** C. Bergdorf, 1♂, (USMN). **Guanacaste:** 1♀ (MCZ); Nicoya Peninsula, cave near Loma Bonita, 10°15'04.0"N, 85°17'30.5"W, ~31 m, 18.I.2008, S. Crews, SCC08_023, 1♂ (EME sel_994); Palo Verde National Park, Cueva las Tigres, 10°21'58.9"N, 85°21'14.2"W, ~31 m, 17.I.2007, S. Crews, under rock, under bark, dry limestone forest, SCC08_022, 1♂ (CAS sel_990). **Oricuajo:** Biolley, Tristan, several (MCZ). **San Joaquin:** Heredia Espinach, Tristan, 1 imm. (MCZ). **San Jose:** 1♀ (MCZ); Valerio (MCZ); E. Schmidt, several (AMNH). **ECUADOR: Galápagos Islands:** Isla Santa Cruz, Academy Bay, Charles Darwin Research Station, ~5 m, 12.III.1970, M. Silbergleid, 1♀ (MCZ); Isla Santa Cruz, Academy Bay, Charles Darwin Research Station, ~5 m, 9.III.1970, M. Silbergleid, 1♀ (MCZ); Isla Santa Cruz, near Caseta, WNW of Academy Bay, 200 m, M. Silbergleid, 1♀ (MCZ); Floreana, 1♂ (MCZ); Isla Santa Cruz, 3 km south Bellavista, transect Z, 115 m, 1-8.IV.1989, S. Peck, 1♀, 1♂ (AMNH). **EL SALVADOR: Chaletenango:** Mun. Tejutto, Rest. Eucalyptos, 5.I.2008, 14°12'20.5"N, 89°06'43.9"W, ~447 m, S. Crews, R. Duncan, J. Carver, P. Berea, under rocks, SCC08_008, 1♂, 1 imm. (EME sel_922-923). **San Salvador:** 1928, S. Caldero, 2♀ (USNM). **San Vicente:** Mun. Tepetitán, vic. Finca El Carmen 13°37'53.0"N, 88°50'19.5"W, ~732 m, 4.I.2008, S. Crews, R. Duncan, SCC08_005, 7♀, 1♂, 1p♂ (CAS sel_897-898, 900, 902, 906-909, 911). **GUATEMALA: Altaverapaz:** Lanquin, El Retiro hotel, 30.XII.2007, ~146 m, 15°35'01.9"N, 89°58'31.3"W, S. Crews, R. Duncan, SCC07_056, 1 imm. (EME sel_864). **El Paso:** Peten, 1931, C.L. Lundell, 1♀ (AMNH). **Petén:** Sta. Elena de la Cruz, Colonia del Bosque, cueva Actun Kan, ~142 m, 16°54'15.7"N, 89°53'54.6"W, 31.XII.2007, S. Crews, SCC08_001, 1♀ (EME sel_868). **Solola:** Olas de Moca, III.1945, H. Elishewitz, 1♂ (AMNH). **HONDURAS: Francisco Morazán:** Tegucigulpa, 29.VI.1917, F.J. Dyay, 1♀ (AMNH); Zamorano, 19.X.1946, T.D.A. Cockerell, 1♂ (AMNH). **Olancho:** Corozal, 9.V.1949, 1♂ (AMNH). **Swan Islands:** 1.IV.1913, G. Nelson, 1♀ (MCZ). **MÉXICO: Chiapas:** Bachajon, 90 airline km northeast San Cristobal, VII.1967, S. Roth, 1♀ (AMNH);Berriozabal, dirt road to Efrain A Gutierrez, 8 km north of Berriozabal, 16°51'16.7"N, 93°17'21.6"W, VIII.2007, I. Martinez-Solano, 1 imm. (CAS sel_848); Huixtla, Las Golondrinas, ~4667', 15°25.747'N, 92°39.270'W, 22.IX.2004, S. Crews, U.O.G. Vázquez, A. Mendoza, under concrete blocks stacked under shelter, SCC04_020, 1♂, 1p♂, 8 imm. (CNAN sel_031, 034-037, 039-042, 1011); La Esperanza, 800 m, 19.IX. 1939, C. Bolivar, D. Peiaez, 2♀ (AMNH); La Esperanza, 40 km north of Escuintla, 2.IV.1945, T.C. Schneirla, 1♀ (AMNH); La Reforma, Municipio de la Concordia, 15°54.212'N, 92°40.157'W, ~6310', 18.IX.2004, S. Crews, A. Mendoza, U.O.G. Vázquez, on fence post on side of road, during the day, SCC04_018b, p♀ (EME sel_044); La Zacualpa, 1.VIII.1909, A. Petrunkevitch, 1♀ (AMNH); La Zacualpa, VII-VIII.1909. A. Petrunkevitch, 1♀ (AMNH); Motozintla de Mendoza, Tolíman, ~1078 m, 15°18'59.3"N, 92°19'54.3"W, en tronco de arbol con *Anolis* y hormigas en la noche, A. Mendoza, SCC004_019a, 1 imm. (EME sel_032); Motozintla de Mendoza, Chevolcán, 21.IX.2004, 1752 m, 15°20'52.4"N, 92°19'25.4"W, U.O.G. Vázquez, A. Mendoza, SCC04_019, 1 imm. (EME sel_033); dirt road to Roberto Barrio, 4 km southwest Nuevo Sonora, 17° 23' 41.1'' N 91° 54' 10.7'' W, VIII.2007, on banana plant, I. Martinez-Solano, 1 imm. (CAS sel_849); Mapastepec, 1.III.1941, H. Wagner, 1♀ (AMNH); Prusia, 1000 m, III-IV. 1942, H. Wagner, 1♀, 1 imm. (AMNH); Tuxtla-Gutierrez: Cañon del Sumidero, A. Mendoza, 1p♂, 1♀ (CNAN sel_1016-1017). **Hidalgo:** Mun: Villa Flores, road El Rayo-Villa Flores, 21°13'30"N, 99°01'13.7"W, 2.XI.2007, 792 m, U.O.G. Vázquez, under rock beside river, 1 imm. (CNAN sel_1008). **Nuevo Leon:** Montemorelos, 23.V.1952, 1♀ (AMNH); Río Ramos, near Villa Santiago, under rocks near swift stream, 20.XII.1939, F. Norman, 1♂, 1♀ (AMNH). **Oaxaca:** Oaxaca, 1.III.1968, O'Rourke, 1♀ (AMNH). **San Luis Potosí:** Cueva de los Savinos, near Valles, 8.III-4.IV.1946, B.J. Dontzin, E. Ruda, 1♀ (AMNH); Pujal, 1♀ (AMNH); 3 mi southeast of Salto de Aqua, VIII.1955, T. Cohn, 3♀ (AMNH); 9 km north of Valles, Sotano de Tinajas, 11.I.1980, B.,V. Roth, 1♂ (AMNH); Tamazunchale, 20.V.1952, M. Cazier, W. Gertsch, R. Schrammel (AMNH); Valles, 21.V.1952, 1p♂ (AMNH). **Tabasco:** Boca del Cerro, 1.III.1945, M. Guerra, 1♂ (AMNH). **Tamaulipas:** 25 miles south of Victoria, 28.XII.1947, 1♀ (AMNH); 15.4 miles south of San Isidron on Mexico 85, 15.II.1961, D. and H. Campbell, 1♀ (AMNH); Mante, 22°45'N, 98°58'W, 17.IV.1963, W.J. Gertsch, W. Ivie, 1♂, 1♀, 1 imm. (AMNH); Rancho Milagro Cruillas, 1 imm. (MCZ); Villa Juarez, 5.VI.1941, 1♀ (AMNH). **Veracruz:** Alto Lucero, 12.V.1947, H. Wagner, several (AMNH); Atoyac, 13.XI.1941, C.B. and F.V., several (AMNH); Carrizal, 10.II.1948, H. Wagner, 1♀ (AMNH); Cinco Chorros, 25 miles west of Catamace, 18°30'N, 95°2'W, 13.II.1984, V. and B. Roth, 1♀ (AMNH); Fortin, 26.VI.1944, L.I. Davis, 1♀ (AMNH); Jalapa, 18.III.1948, H. Wagner, 1♂ (AMNH), Jalapa, 19.III.1948, H. Wagner, 1♂, 1♀ (AMNH); Tamalín, El Mamey, 3 imm. (CNAN sel_1018-1020). **Yucatan:** Copan, 4.XI.1902, R.V. Chamberlin, 1♀ (AMNH). **NICARAGUA: Altagracia:** Lago Nicaragua, Isla Ometepe, Charco Verde, Volcán Concepción, vic. Hotel Finca Vincenzia, 11°28'42.6"N, 35°38'20.6"W, ~670 m, 12.I.2008, S. Crews, R. Duncan, SCC08_014, 3♀, 2 imm. (EME sel_940, 944, 947, 949-950); Totogalpa, Alcadia Ocotal, Departmiento Madríz, 13°33'49.5"N, 86°29'54.6"W, ~672 m, 11.I.2008, S. Crews, R. Duncan, P. Berea, 1♂ (EME sel_934). **Boaco:** Aguascalientes, Alc. Teustepe, Camino La Cuesta, 12°22'57.8"N, 85°47'30.7"W, ~195 m, 15.I.2008, S. Crews, SCC08_020, 2♀ (EME sel_977, 986). **Madríz:** Alc. Ocotal, Totogalpa, 11.I.2008, 13°33'49.5"N, 86°29'64.6"W, ~672 m, S. Crews, SCC08_013, under bark of fence post, 1♀ (CAS sel_935). **Matagalpa:** Alc. San Ramon, Mata Palo, 12°56'16.5"N, 85°51'12.2"W, ~886 m, 14.I.2008, cloud forest, coffee, bananas, S. Crews, R. Duncan, P. Berea, SCC08_018, 1♀, 2 imm. (CAS sel_971, 973-974). **PANAMÁ:** 1♀ (MCZ). **Boquete:** 1-8.VIII.1950, A.M. Chickering, several (MCZ). **Canal Zone:** Ancon, VI.1906, Wheeler, 1♀ (MCZ); Balboa, 18. III. 1954, W.E. Lundy, 1♂, 1♀ (AMNH); Barro Colorado Island:W.C. Allee (MCZ); Barro Colorado Island, STRI, Galeta Pt. Plot F, 2005, C.J. Hayden, 5 imm. (EME sel_265-269); Fort Sherman, 15.VIII.1950, A.M. Chickering, several (MCZ); France Field, 29.XII.1965, B. Payne, 1♀ (AMNH). **Chiriqui:** El Volcan, 18.II. 1936, W.J. Gertsch, 2♀ (AMNH). **El Valle:** A.M. Chickering, 1950, several (MCZ). **Panama City:** 25.I.1945, C.D. Michener, 1♂ (AMNH); XI.1945, C.D. Michener, 1♂ (AMNH). **ST. MAARTEN: Philipsburg:** Front Street, on palm tree at night near entrance to cruise ship dock, 18°00.906' N, 63°02.587' W, ~6 m, S. Crews, SCC04_067, 1p♀, 1♂ (CAS sel_117-118). **UNITED STATES: Arizona:** Kingman, Safeway Store on bananas, 13.X.1981, Mrs. Hackley, 1 imm. (AMNH). **Florida:** Broward Co., Davis Holiday Park, 27.II.2002, S. Costello, 2♀. **Washington:** Yakima, on food store produce, 30.IV.1976, W. Hudson, 1♀ (USNM). **Wisconsin:** Wasau, on bananas, XII.1953, W. Levandoski, 1♀, 1♂ (MCZ).

#### Diagnosis.

**Figures 29–36.**
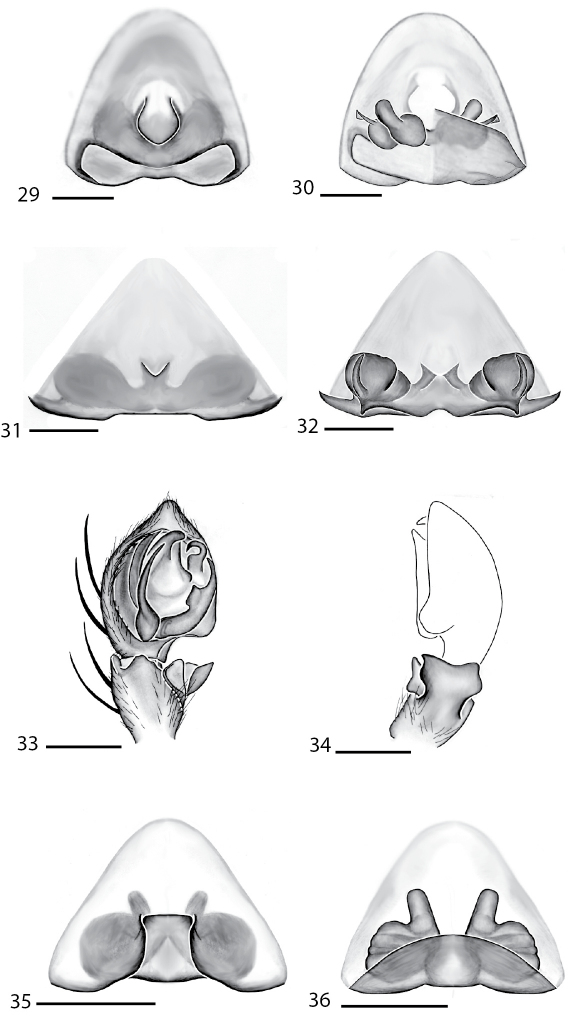
Copulatory organs of *Selenops mexicanus* Keyserling lectotype female from México (BMNH) **29–30** *Selenops abyssus* Muma, female holotype from Tizapan, Jalisco, México (AMNH) **31–32** *Selenops marginalis* F.O.Pickard-Cambridge lectotype male from Omilteme, Guerrero, México (BMNH) **33–34** paralectotype female Omilteme, Guerrero, México (BMNH) **35–36, 29, 31, 35** epigyne, ventral view **30, 32, 36** spermathecae, dorsal view **33** left pedipalp, ventral view **34** left pedipalp, retrolateral view. Scale bar = 0.50 mm (29–30, 33–36), 0.40 mm (31–32).

This species is most similar to *Selenops aztecus*, *Selenops gracilis*, and *Selenops malinalxochitl*. The males can be distinguished by the long, sinuous, dorsal branch of the RTA, pointed at the tip. Embolus fairly short, located medially, not covered by another sclerite (Figs 27–28). In females, the median field is more sclerotized than in the other species, and internally, the ducts are branched, with one branch directed anteriorly. The spermathecae can be seen just anterior to the margin of the posterodorsal fold in most specimens. The posterodorsal fold is large and quadrangular, though may be slightly rounded (Figs 29–30).

#### Remarks.

Several species are synonymized with *Selenops mexicanus*. The types of these species were compared with the available lectotype of *Selenops mexicanus* and/or DNA evidence from spiders from the same localities (Crews and Gillespie 2010) and these are being used as evidence for these synonymies. In the types of *Selenops tehuacanus* and *Selenops galapagoensis*, no differences were detected between these types and the types of *Selenops mexicanus*. The types of *Selenops vagabundus* were unable to be examined. However, from the illustrations of Kraus (1955), there are some differences in the male. I collected specimens that match the illustrations of Kraus (1955), but using DNA data, the specimens are deeply nested within the *Selenops mexicanus* clade. The differences include a shorter, blunter RTA and part of the MA is obscured by a tergite from which the embolus arises. From the illustration of the female copulatory organs (Kraus 1955) it is difficult to determine if there are any differences, however, new specimens were taken from the same locality and sequenced, and no differences were found.

There is some variation as might be expected in such a widespread species. Genitalic details reveal consistency in males in the RTA, MA and embolus, as well as in the internal copulatory organs in the female. Though the RTA may not be as long as in the holotype specimen (Muma 1953: figs 1–2), it is sinuous and tapers distally. MA with two branches in all specimens, with two bent, finger-like processes, though one branch may be obscured by the sclerite from which the embolus arises in some specimens. The lateral edge of the conductor is slightly sinuous in some specimens. The shape of the median septum and the degree of sclerotization of the median field varies. The median septum may be round to angular and the median field may only be lightly sclerotized or more strongly sclerotized. The area around the median field may be very defined or raised, or may be flat. The posterior indentations also differ in their shape and size. The posterodorsal fold is typically large and quadrangular, though may be rounded or smaller, but a portion of the spermathecae can always be seen above the anterior margin, and the fertilization ducts can usually be seen laterally.

It is clear that other closely related species may yet be discovered (e.g. the female of *Selenops aztecus*). The genitalic features mentioned above as well as DNA will be useful in distinguishing these new species from *Selenops mexicanus*.

This species appears to be able to escape detection on produce or landscape plants and has been introduced into the US and St. Maarten. In St. Maarten, a male and young female were collected on a palm that had been shipped from Florida, via México, indicating the species is well-suited for travel. In most areas where it has been found in the US, such as Wisconsin and Washington, climate would likely not allow the spider to become established. In Florida or St. Maarten, the climate is similar to the native range and the species may be able to become established. This could be detrimental to local or endemic species, such as *Selenops souliga* sp. n. in St. Maarten.

#### Description.

*Male (sel_934):* Color:carapace light brownish-orange with dusky marks medially and laterally; sternum light orange-brown; chelicerae brown; maxillae light yellow-brown, lightening to white distally; labium orange-brown, lightening toward the distal edge; abdomen dorsally light tan to grey with darker markings, lanceolate medial stripe, and chevrons, festoon present; ventrally cream-colored, dark on sides, and posteriorly; legs light yellow-brown, slightly more orange (darker) distally, annulations faint but visible. Cephalothorax: 0.81 times longer than broad; fovea longitudinal, broad, very shallow. Eyes:AER nearly straight; PER slightly recurved; PME larger than AME, PLE largest, ALE smallest; eye diameters, AME 0.18, ALE 0.08, PME 0.23, PLE 0.28; interdistances AME-PME 0.08, PME-ALE 0.10, ALE-PLE 0.53. PME-PME 1.20. ALE-ALE 1.80; ocular quadrangle AME-AME 0.45, PLE-PLE 2.33; clypeus 0.06 high. Mouthparts:chelicerae with stout setae medially and anteriorly; maxillae longer than broad, with tuft of conspicuous setae distally; labium distally rounded. Sternum:0.90 times longer than broad, posteriorly indented. Legs:leg I only slightly shorter than legs II, III and IV; leg formula 2341; scopulae present distally on all 4 tarsi; tarsi I-IV with strong claw tufts; pr claw toothed, rl claws with fewer teeth; spination: leg I, Fm pr 1–1–0, d 1–1–1, rl 1–1–1; Ti pr 1–0–1, d 0, rl 1–0–1, v 2–2–2; mt v 2–2; leg II, Fm pr 1–1–0, d 1–1–1, rl 1–1–1; Ti pr 1–0–1, d 0, rl 1–0–1, v 2–2–2; Mt v 2–2; leg III, Fm pr 1–1–0, d 1–1–1, rl 1–1–1; Ti pr 1–0–0, v 2–2, rl 1–0–0; Mt v 2–0; leg IV, Fm pr 1–0–0, d 1–1–1, rl 0–1–1; Ti pr 1–0–0, rl 1–0–0, v 1–1; Mt v 2–1. Abdomen:without terminal setal tufts. Pedipalp:Fm, spination dorsal 0–1–4; cymbium oval to round in ventral view, angled posterolaterally; scopulae scattered, denser toward tip; conductor quadrate, lightly sclerotized, arising toward top of bulb; embolus very short, arising at 9 o'clock, terminating at 11 o'clock, attached to a larger quadrate structure that is distally sinuous; MA two-branched with each branch slender and curved, one slightly more blunt than the other, located at 3 o'clock, directed ventrally; RTA with 2 processes, the dorsal process is long and sinuous, pointed at the tip, ventral process smaller; RTA extending at least 1/2 length of cymbium in ventral view (Figs 27–28). *Dimensions:* Total length 9.85. Carapace length 3.95, width 4.90. Sternum length 1.80, width 2.00. Abdomen length 5.90, width 4.50. Pedipalp: Fm 1.00, Pt 0.30, Ti 0.75, Ta 1.50, total 3.55. Leg I: Fm 5.00, Pt 2.00, Ti 4.60, Mt 4.00, Ta 1.75, total 17.35. Leg II: Fm 6.00, Pt 2.00, Ti 5.00, Mt 5.00, Ta 1.75, total 19.75. Leg III: Fm 6.00, Pt 1.50, Ti 5.00, Mt 4.65, Ta 1.75, total 18.90. Leg IV: Fm 5.75, Pt 1.50, Ti 4.50, Mt 4.50, ta 1.60, total 17.85.

*Lectotype female.* Color:carapace (lectotype) mahogany red, (recent) brownish-red; sternum (lectotype) light orange-brown, darker around border (recent) dark red-brown; chelicerae (lectotype) red-brown (recent) dark red-brown; maxillae orange-brown, lightening distally; abdomen (lectotype) yellowish-orange, faded, remnants of w-shaped markings from the top to 3/4 of the way to the posterior end, festoon present (recent) light with darker spots and chevrons, somewhat variable; ventrally (lectotype) yellowish, (recent) grey; legs orange to brown, darkening slightly distally, annulations visible, very dark on half of tibia, the lighter area very light (Fig. 69E); Carapace: 0.93 times longer than broad; fovea longitudinal, broad, very shallow. Eyes: AER nearly straight; PER slightly recurved; PME larger than AME, PLE largest, ALE smallest; eye diameters, AME 0.40, ALE 0.45, PME 0.15, PLE 0.55; interdistances AME-PME 0.10, PME-ALE 0.23, ALE-PLE 0.78. PME-PME 1.80. ALE-ALE 2.90; ocular quadrangle AME-AME 0.70, PLE-PLE 3.50; clypeus 0.08 high. Mouthparts:chelicerae with a few stout setae medially and anteriorly; maxillae longer than broad, with tuft of conspicuous setae distally; labium distally rounded. Sternum:0.92 times longer than broad, posteriorly indented. Legs:leg I only slightly shorter than legs II, III and IV; leg formula 2341 (Muma 1953); scopulae present on tarsi of all legs and metatarsi of legs I and II; tarsus I-IV with strong claw tufts; pr claw toothed, rl claws with fewer teeth; spination: leg I, Fm pr 1–1–0, d 1–1–1, rl 1–1–1; Ti d 0, v 2–2–2; Mt v 2–2; leg III, Fm pr 1–0–0, d 1–1–1, rl 0–1–1; Ti v 2–2–0; Mt v 2–1; leg IV, Fm pr 1–0–0, d 1–1–1, rl 0; Ti v 2–1; Mt v 2–0. Abdomen:without terminal setal tufts. Pedipalp:claw with 13 teeth. Epigyne:Median area slightly sclerotized, with diamond to lobe-shaped median septum; space between septum and more sclerotized area; genital openings located at the lateroposterior margins of median septum; large medial to lateral indentations; internally, branched ducts, directed laterally and anterolaterally, fertilization ducts located and directed laterally, large, heavily sclerotized posterodorsal fold covering the majority of the internal ducts (Figs 29–30). *Dimensions:* Total length 14.25. Carapace length 6.45, width 6.90. Sternum length 3.00, width 2.75. Abdomen length 7.80, width 6.95. Pedipalp: Fm 1.65, Pt 0.80, Ti 1.00, Ta 1.75, total 5.20. Leg I: Fm 5.50, Pt 2.70, Ti 4.50, Mt 3.75, Ta 1.30, total 17.75. Leg II: Missing. Leg III: Fm 7.00, Pt 3.00, Ti 4.75, Mt 4.40, Ta 1.75, total 20.80. Leg IV: Fm 7.00, Pt 2.00, Ti 5.00, Mt 4.00, Ta 1.75, total 19.75.

#### Natural history.

This species has been found under rocks, bark, concrete blocks and other debris, on fence posts, in houses, on trees (Fig. 181) and banana plants during the day as well as at night. It has been found in both wet areas and dry areas, from sea level to higher elevations. It is able to escape detection and has made it out of its natural range on several occasions. It is very widespread and, thus, often found with other species of selenopid, in particular *Selenops bifurcatus*.

#### Distribution.

This species is one of the most widespread species of selenopids, naturally occurring from northern México, south to northern South America and the Galápagos Islands (Map 6).

### Selenops abyssus

Muma, 1953

http://species-id.net/wiki/Selenops_abyssus

Figs 31–32 Map 5

Selenops abyssus Muma 1953: 21, Fig. 36 (♀, examined).

#### Type material.

Holotype female: Tizapan, Jalisco, México, 6.I.1946, F. Bonet (AMNH, examined).

#### Other material examined.

MÉXICO: Colima: Manzanillo, Mun. Manzanillo, 1.2–1.4 km East of La Central, 1♀ (CNAN sel_1004); Mun. Ixtlahuacán, Tamala, 1 imm. (EME sel_1013); **Jalisco:** 5 km southwest of Tecalitlán, 2.I.1999, 1200 m, E.S. Ross, R.E. Stecker, 1♀ (CAS); 12 miles northwest of Jiquilipan, 22.X.1973, S.C. Williams, C. L. Mullinex, 2♀ (CAS); **Michoacán:** 10 miles south of Uruapan, Hwy 37, 2.IX.1977, 1200 m, E.I. Schlinger, 1♀ (CAS); Mun. Coalcomán, Cerro La Penita, 1 imm. (CAS sel_1012); **Nayarit:** Islas Tres Marias, María Madre, 13-23.V.1925, H.H. Keifer, 1♀ (CAS); vic. Campostela, 16.X.1935, 1♀ (AMNH).

#### Diagnosis.

This species can be distinguished from others by the inconspicuous lateral lobes, and the triangular epigynal plate, with a central v-shape (Fig. 31). Internally, the spermathecae are large and round, located laterally (Fig. 32). Males unknown.

#### Description.

*Holotype female:* Color:Carapace (holotype) brownish-red (recent) dusky yellow, cephalic region brown, lateral margins dark; sternum (holotype) red-brown (recent) yellow-brown, darker at border; chelicerae (holotype) brown-red (recent) brown, darker laterally; maxillae (holotype) orange-brown, lightening distally (recent) light brown, darkening on the distolateral border, lightening on inner distal border; labium dark brown, lightening distally; abdomen dorsally (holotype) damaged (recent) dusky grey-brown with darker mottling, ventrally (holotype) dark grey (recent) cream-colored; legs (holotype) brown-red, annulations no longer visible (recent) yellowish, darkening distally, with annulations and specks, lighter on femora, with no markings retrolaterally. Carapace:0.98 times longer than broad. Eyes:AER slightly recurved; PER recurved; PME larger than AME, PLE largest, ALE smallest; eye diameters, AME 0.20, ALE 0.03, PME 0.30, PLE 0.45; interdistances AME-PME 0.13, PME-ALE 0.18, ALE-PLE 0.48. PME-PME 1.28. ALE-ALE 2.23; ocular quadrangle AME-AME 0.48, PLE-PLE 2.45; clypeus 0.15 high. Mouthparts:chelicerae with stout setae medially and anteriorly; maxillae longer than broad, with tuft of conspicuous setae distally; labium distally rounded. Sternum:As long as broad, posteriorly indented. Legs:Leg formula 4321 (Muma, 1953); scopulae present on tarsi of all legs and metatarsi of legs I and II; tarsi I-IV with strong claw tufts; pr claw toothed, rl claw with fewer teeth; spination: leg I, Fm pr 1–1–0, d 1–1–1, rl 1–1–1; Ti v 2–2–2; Mt v 2–2; II, Fm pr 1–1–0, d 1–1–1, rl 1–1–1; Ti v 2–2–2; Mt v 2–2; III, Fm pr 1–1–0, d 1–1–1, rl 1–1–1; Ti v 2–2–0; Mt v 2–2; IV, Fm pr 1–1–0, d 1–1–1, rl 1–1–1; Ti v 2–2–0; Mt v 2–2. Abdomen:Without terminal setal tufts. Pedipalp:claw with 13 teeth. Epigyne:Plate triangular, lateral lobes inconspicuous, small v-shaped area located medially, genital openings located laterally to v-shape, epigynal pockets absent; internally, ducts directed medially to laterally leading to laterally located spermathecae, fertilization ducts located laterally, directed anteriorly, small posterodorsal fold present, though it does not cover ducts (Figs 31–32). *Dimensions:* Total length 11.30. Carapace length 5.40, width 5.50. Abdomen length 5.90, width 2.60. Pedipalp: Fm 1.00, Pt 1.50, Ti 0.75, Ta 1.00, total 4.25. Leg I: Fm 5.30, Pt 2.00, Ti 2.96. Leg II: Absent. Leg III: Fm 5.00, Pt 2.20, Ti 4.25, Mt 4.25, Ta 2.00, total 17.70. Leg IV: Fm 6.50, Pt 2.00, Ti 5.31, Mt 4.50, Ta 2.00, total 20.31

#### Natural history.

Collected from lower elevations up to 1200 m.

#### Distribution.

Found in southwestern México from Nayarit to Michoacán (Map 5).

### Selenops juxtlahuaca

Valdez-Mondragón, 2007

http://species-id.net/wiki/Selenops_juxtlahuaca

Map 5

Selenops juxtlahuaca Valdez-Mondragón 2007: 65, Figs 1–8 (♂, ♀; ♂ holotype and ♀ paratypes from near the entrance Grutas de Juxtlahuaca, 5 km northwest of Colotlipa, Mun. Quechultenango, 17° 26' 32.4" N, 99° 09' 57.0" W, Guerrero, México, 17.I.2006, A. Valdez-Mondragón, H. Montaño, in CNAN, not examined).

#### Natural history.

Collected in and around the entrance to a cave, under limestone rocks, in tropical deciduous forest (Valdez-Mondragón pers. comm.).

#### Distribution.

Known only from the type locality (Map 5).

### Selenops marginalis

F. O. Pickard-Cambridge, 1900

http://species-id.net/wiki/Selenops_marginalis

Figs 33–36 Map 5

Selenops marginalis F. O. Pickard-Cambridge 1900: 117, pl. 8, Figs 15–16 (♂, ♀).
Selenops marginalis :Muma 1953: 43, Figs 74–75 (♂, ♀).

#### Type material.

Lectotype male (here designated) and male and female paralectotypes (here designated): Omilteme, Guerrero, México, Godman and Salvin (BMNH 680, examined).

#### Note.

Examination of the specimens described by F. O. Pickard-Cambridge (1900) revealed two males and one female in the type series vial with a ‘cotype' label. Muma (1953: 43) did not examine F. O. Pickard-Cambridge's types. In the interest of nomenclatural stability one male is designate as the lectotype and the other male and female as the paralectotypes.

#### Other material examined.

MÉXICO: Guerrero: same data as the lectotype, 1♂, 1p♂, 2p♀ (BMNH 1905.4.28-10-13).

#### Diagnosis.

Males can be differentiated from other species by the embolus which has a round base, and is bladelike distally. Embolus located more medially than laterally. Also, MA with quadrangular base and strongly hooked distally (Fig. 33). Females can be separated from other species by the quadrangular median area of the epigynum (Fig. 35).

#### Remarks.

In the illustration of the ventral view of the palp of the male by F. O. Pickard-Cambridge (1900: fig. 15), the MA looks somewhat different than on the lectotype. Everything else looks exactly the same. It is my conclusion that the MA was articulated differently, and that the equipment used to view the palp such a long time ago was perhaps not as sophisticated as equipment used today, and thus, this is definitely the same species. This species has been listed as occurring in Panamá (Nentwig et al. 1993), however, I believe this to be a misidentification.

#### Description.

*Lectotype male:* Color:carapace light orange; sternum light orange-brown; chelicerae orange-brown, with some dusky markings; maxillae orange-brown; labium orange-brown to brown, lightening distally; abdomen dorsally yellow-grey with remnants of a lanceolate foliate pattern, chevron at 1/3 the length and at 2/3 the length of the abdomen, horizontal stripes at distal end, dark laterally, terminally, festoon present; ventrally dusky grey with no markings; legs orange, no markings visible any longer. Carapace: 0.88 times longer than broad; fovea longitudinal, broad, very shallow. Eyes:AER slightly recurved; PER recurved; PME larger than AME, PLE largest, ALE smallest; eye diameters, AME 0.20, ALE 0.10, PME 0.28, PLE 0.35; interdistances AME-PME 0.08, PME-ALE 0.15, ALE-PLE 0.38. PME-PME 1.18. ALE-ALE 1.80; ocular quadrangle AME-AME 0.48, PLE-PLE 1.95; clypeus 0.10 high. Mouthparts:chelicerae with a few stout setae medially and anteriorly; maxillae longer than broad, with tuft of conspicuous setae distally; labium distally rounded. Sternum:1.25 times longer than broad, posteriorly indented. Legs:leg formula unknown (at least one leg missing); scopulae present on distal end of all 4 tarsi; tarsi I-IV with strong claw tufts; pr claw toothed, rl claw with fewer teeth; spination: II, Fm pr 1–1–0, d 1–1–1, rl 1–1–1. Abdomen:without terminal setal tufts. Pedipalp:Fm, spination d 0–1–4;cymbium oval in ventral view, angled bottom, right, very pointed distally; scopulae scattered, denser toward tip; basal cymbial process absent; conductor arising medially and laterally, pointed distally; embolus short and blade like with an oval base, tapering abruptly, directed anteriorly instead of curving around the lateral edge, originating at 6 o'clock terminating at 11 o'clock; MA with a stout quadrangular base, with stout terminal hook, located at 2 o'clock, directed laterally; RTA barely reaching cymbium in ventral view, with two processes, the lateral one on a small stalk, widening distally into a slightly quadrate structure with sinuate lateral margins, truncated distally; ventral branch small and triangular (Figs 33–34). *Dimensions:* Total length 7.85. Carapace length 4.10, width 4.68. Sternum length 2.50, width 2.00. Abdomen length 3.75, width 2.88. Pedipalp: Fm 1.50, Pt 0.25, Ti 0.90, Ta 1.45, total 4.10.

*Paralectotype female:* Color:carapace orange-brown, some dusky markings medially and laterally; sternum light orange-brown; chelicerae orange-brown, with some dusky markings; maxillae brown, lightening distally; labium light brown lightening distally; abdomen dorsally yellow-grey with remnants of lanceolate foliate pattern, chevrons 1/3 and 2/3 down the length of the abdomen, horizontal stripes terminally, dark laterally, festoon present; ventrally dusky grey with no markings; legs orange-brown with annulations, also dark patches on femora of legs I and II and patella, tibia and metatarsus of legs III and IV; Carapace: 0.88 times longer than broad; fovea longitudinal, broad, very shallow. Eyes:AER slightly recurved; PER recurved; PME larger than AME, PLE largest, ALE smallest; eye diameters, AME 0.20, ALE 0.08, PME 0.28, PLE 0.30; interdistances AME-PME 0.03, PME-ALE 0.18, ALE-PLE 0.48. PME-PME 2.18. ALE-ALE 1.90; ocular quadrangle AME-AME 0.50, PLE-PLE 2.10; clypeus 0.06 high. Mouthparts:chelicerae with a few stout setae medially and anteriorly; maxillae longer than broad, with tuft of conspicuous setae distally; labium distally rounded. Sternum:1.20 times longer than broad, posteriorly indented. Legs:leg I much shorter than legs II, III and IV; leg formula 2341; scopulae present on tarsi of all legs and metatarsi of legs I and II; tarsi I-IV with strong claw tufts; pr claw toothed, rl claw with fewer teeth; spination: leg I, Fm pr 1–1–0, d 1–1–1, rl 1–0–1; Ti v 2–2–2–1; Mt v 2–2–2; leg II, Fm pl 1–0–1, d 1–1–1, rl 1–0–1; Ti v 2–2–2; Mt v 2–2; leg III, Fm pl 1–0–0, d 1–1–1, rl 1–0–1; Ti v 1–1–0; Mt v 2–0; leg IV, Fm pr 1–0–0, d 1–1–1, rl 0–0–1; Ti v 1–1; Mt v 1-0;. Abdomen: without terminal setal tufts. Pedipalp:claw with 13 teeth. Epigyne:median field quadrangular, genital openings located anterolaterally to median quadrangular area; internally, cylindrical ducts directed anteriorly, spherical somewhat coiled ducts located laterally, posterodorsal fold present, rounded, covering about half of the internal ducts (Figs 35–36). *Dimensions:* Total length 10.00. Carapace length 4.30, width 4.90. Sternum length 2.40, width 2.00. Abdomen length 5.70, width 4.30. Pedipalp: Fm 1.00, Pt 0.50, Ti 0.75, ta 1.00, total 3.25. Leg I: Fm 4.00, Pt 1.75, Ti 3.75, Mt 2.75, Ta 1.5, total 13.75. Leg II: Fm 5.00, Pt 2.00, Ti 4.00, Mt 3.75, Ta 1.75, total 16.50. Leg III: Fm 5.00, Pt 1.80, Ti 4.00, Mt 3.75, Ta 1.60, total 16.15. Leg IV: Fm 5.00, Pt 1.50, Ti 4.00, Mt 3.60, Ta 1.75, total 15.85.

#### Natural history.

No data.

#### Distribution.

Known only from the type locality (Map 5).

### Selenops minutus

F. O. Pickard-Cambridge, 1900

http://species-id.net/wiki/Selenops_minutus

Figs 37–40 Map 5

Selenops minutus F.O. Pickard-Cambridge 1900: 118, pl. 8, Figs 19–20 (♂, ♀, examined).
Selenops minutes Muma 1953: 38: Figs 64–65 (♂, ♀).

#### Type material.

Holotype male: Guatemala, Godman and Salvin (BMNH, examined). Paratypes: Female, same data as holotype (BMNH).

#### Diagnosis.

**Figures 37–44.**
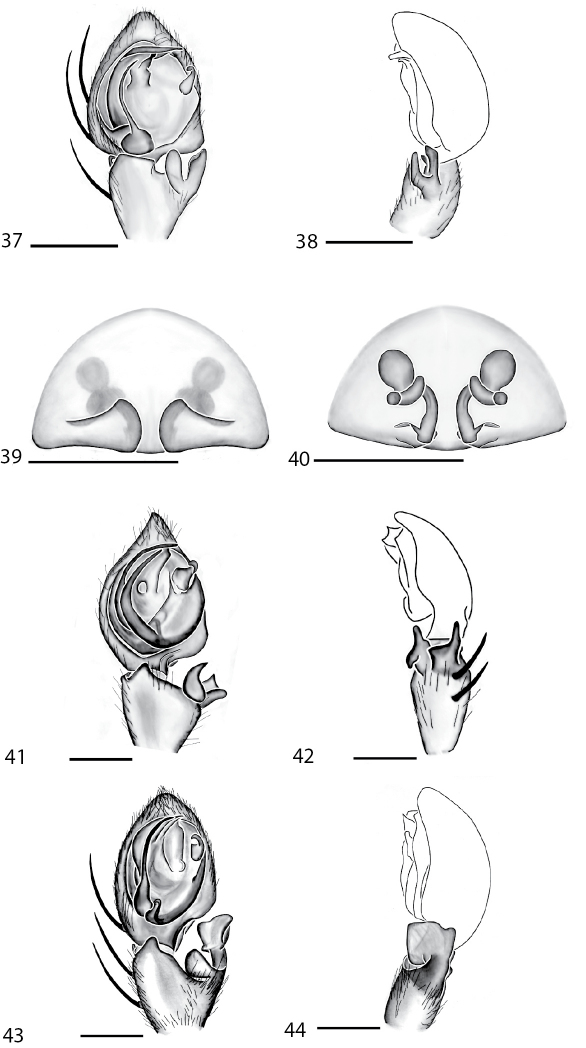
Copulatory organs of *Selenops minutus* F.O.Pickard-Cambridge holotype male from Guatemala (BMNH) **37–38** paratype female from Guatemala (BMNH) **39–40** *Selenops morosus* Banks, lectotype male from Tepic, México (MCZ) **41–42** *Selenops nigromaculatus* Keyserling holotype male from México (BMNH) **43–44, 37, 41, 43** left pedipalp, ventral view **38, 42, 44** left pedipalp, retrolateral view **39** epigyne, ventral view **40** spermathecae, dorsal view. Scale bar = 0.50 mm.

This species can be distinguished from others by a combination of characters, including the overall small size, elaborately patterned abdomen, and genitalic characteristics. In males, these characters are the embolus, which has a round base that tapers abruptly and is very narrow throughout its length, and the RTA, which is claw like laterally (Figs 37–38). In the females, the epigynum has posterolateral lobes that nearly come into contact medially, and oval spermathecae atop long ducts (Figs 39–40).

#### Remarks.

Muma (1953) placed this species with *Selenops micropalpus* and *Selenops banksi*, stating that the embolus and long palpal tibia from the illustrations of F. O. Pickard-Cambridge (1900) were of the same type. Muma (1953) was unable to examine the actual specimens. The illustrations of F. O. P-Cambridge (1900), however, do not show a long palpal tibia, or an embolus similar to that of *Selenops banksi* or *Selenops micropalpus*. F. O. Pickard-Cambridge (1900) also mentioned that none of the females were adults, yet he illustrated an adult. After close examination of the specimens, I do not believe this species to be aligned with *Selenops micropalpus* or *Selenops banksi*, but rather with other species found in México and Guatemala. Unfortunately, this species has not been collected for over 150 years and its precise range is unknown, as the label says only ‘Guatemala.'

This species is listed by Nentwig et al. (1993) as being found in Panamá. This is because Banks (1929) included this in his list of spiders from Panamá, rather than based on a collection made by the former authors. Banks (1929) included no figure of this spider in his publication, and the spider has not been found in any collection examined. I regard Panamá as an erroneous locality for this species.

#### Description.

*Holotype male:*Color:carapace orange-brown; sternum orange-brown; chelicerae orange-brown; maxillae orange-brown; labium orange-brown, lightening toward the distal edge; abdomen dorsally yellowish, remnants of lanceolate, foliate pattern, chevrons extending to lateral margins, occurring about halfway down the length of the abdomen, lots of white; type is old and faded, cannot confirm or deny presence of festoon, however, looks as though it is present in illustrations (F.O. Pickard-Cambridge 1900); ventrally cream-colored, dark laterally and posteriorly; legs orangeish, annulations barely visible, markings on anterolateral faces of femora and tibiae. Carapace 0.92 times longer than broad; fovea longitudinal, broad, very shallow. Eyes:AER slightly recurved; PER recurved; PME same size as AME, PLE largest, ALE smallest; eye diameters, AME 0.10, ALE 0.03, PME 0.10, PLE 0.18; interdistances AME-PME 0.02, PME-ALE 0.80, ALE-PLE 0.19, PME-PME 0.55, ALE-ALE 0.88; ocular quadrangle AME-AME 0.22, PLE-PLE 0.98; clypeus 0.03 high. Mouthparts:chelicerae with a few stout setae medially and anteriorly; maxillae longer than broad, with tuft of conspicuous setae distally; labium distally rounded. Sternum:as long as wide, posteriorly indented. Legs:leg formula unknown (at least one leg missing); scopulae present on distal end of all 4 tarsi; tarsi I-IV with strong claw tufts; pr claw per foot slightly toothed; spination: leg I, Fm pr 1–0–0, d 1–1–1, rl 0; Ti pr 0–0–1, v 1–2–2–2; Mt rl 1–0–0, ventral 2–2; leg II, Fm pr 1–0–0, d 1–1–1, rl 0; Ti v 2–2–2; Mt v 2–2; leg III, Fm pr 0, d 1–1–1, rl 0; Ti 0; Mt 0. Abdomen:without terminal setal tufts. Pedipalp:Fm, spination d 0–1–4; cymbium oval in ventral view, angled posterolaterally margin; basal cymbial process absent; scopulae scattered, denser toward tip; conductor curved laterally and medially, medially a stalk arising from center of bulb, directed laterally at a right angle, extending past the margin of bulb; embolus short, large, round base, tapering abruptly to very narrow, extending straight anteriorly, instead of curving around the perimeter of the cymbium, beginning at 6 o'clock ending at 11; MA small, triangular, with a long base, slightly curved distally forming a very small hook, arising at 3 o'clock, directed distally; RTA barely reaching cymbium in ventral view, with 2 branches, lateral branch narrow, claw-like, ventral branch oblong, rounded distally (Figs 37–38). *Dimensions:* Total length 4.47. Carapace length 2.08, width 2.30. Sternum length 1.50, width 1.50. Abdomen length 2.40, width 2.08. Pedipalp: Fm 0.50, Pt 0.25, Ti 0.25, Ta 0.50, total 1.50.

*Paratype female:*Color:carapace light orange; sternum light yellowish to orange brown, darker around border; chelicerae dusky yellow; maxillae pale yellow-brown; labium pale yellow-brown; abdomen dorsally yellowish, remnants of lanceolate, foliate pattern, chevrons extending laterally about halfway down length of abdomen, lots of white, type is old and faded, cannot confirm or deny presence of festoon, but it looks as though it is present in illustrations (F. O. Pickard-Cambridge, 1900); ventrally cream-colored; legs orangeish, annulations barely still visible, markings on anterolateral faces of femora and tibiae. Carapace: 0.91 times longer than broad; fovea longitudinal, broad, very shallow. Eyes:AER slightly recurved; PER recurved; PME larger than AME, PLE largest, ALE smallest; eye diameters, AME 0.08, ALE 0.03, PME 0.17, PLE 0.19; interdistances AME-PME 0.07, PME-ALE 0.95, ALE-PLE 0.25. PME-PME 0.68, ALE-ALE 1.18; ocular quadrangle AME-AME 0.30, PLE-PLE 1.35; clypeus 0.03 high. Mouthparts:chelicerae with a few stout setae medially and anteriorly; maxillae longer than broad, with tuft of conspicuous setae distally; labium distally rounded. Sternum:as long as broad, posteriorly indented. Legs:scopulae present on distal end of all 4 tarsi; tarsi I-IV with strong claw tufts; pr claw per foot slightly toothed; leg II, Fm pr 0, d 1–1–1, rl 0; Ti v 2–2–2; Mt v 2–2; leg III, Fm pr 0, d 1–1–1, rl 0; Ti 0; Mt 0; leg IV, Fm pr 1–1–1, d 1–1–1, rl 1–1–1. Abdomen: without terminal setal tufts. Pedipalp:claw with 8 teeth. Epigyne:posterolateral lobes, widening medially, not touching medially, genital openings located along anterior margins of posterolateral lobes; internally, ducts twist once, extending to oval spermathecae anteriorly, and lengthening posteriorly to laterally directed fertilization ducts, posterodorsal fold absent (Figs 39–40). *Dimensions:* Total length 6.28. Carapace length 3.00, width 3.29. Sternum length 1.50, width 1.50. Abdomen length 3.28, width 4.00. Pedipalp: Fm 0.60, Pt 0.25, Ti 0.50, Ta 0.75, total 2.10. Leg I: Fm 4.00, Pt 1.00, Ti 3.75, Mt 2.75, Ta 1.00, total 12.50 Leg II: Missing. Leg III: Missing. Leg IV: Missing.

#### Natural history.

No data.

#### Distribution.

All that is known is that the species is found in Guatemala, which may have had different borders at the time the collection was made (Map 5).

### Selenops morosus

Banks, 1898

http://species-id.net/wiki/Selenops_morosus

Figs 41–42 Map 5

Selenops morosus Banks 1898: 267, pl. 16, Fig. 16 (♂, ♀, examined).
Selenops morosus F.O. Pickard-Cambridge 1900: 118 (no Figs).
Selenops morosus Muma 1953: 41, Figs 69–70 (♂, ♀).

#### Type material.

Lectotype male (designated here): Tepic, México, X-XI, Eisen and Vaslit (MCZ, examined). Female syntype has been lost or destroyed, and thus the female is unknown.

#### Other material examined.

MÉXICO: Sonora: 2 miles south of Imuris on Mex 15, 10.IV.1965, W. Shear, 1♂ (AMNH).

#### Diagnosis.

Males can be distinguished by the RTA with two small pointed, angular projections, and the MA, which has a large triangular base and is directed somewhat ventrally (Figs 41–42).

#### Remarks.

Banks (1898) illustrated a male and a female specimen and noted that ‘several specimens' had been collected. Muma (1953) reported that the female type was lost or destroyed, but that females of *Selenops abyssus* may go with males of *Selenops morosus*, as the illustration of the epigynum by Banks (1898) is similar to that of *Selenops abyssus*. Muma also mentions two males and one young female from Tepic at the MCZ, but only a single male was located. An additional specimen from much further north (Sonora) was found in the AMNH. The epigynum illustrated by Banks (1898) looks nothing like that of *Selenops abyssus*, and I conclude that *Selenops abyssus* and *Selenops morosus* are distinct species. Banks (1898) did indeed have the female of *Selenops morosus*. The geographic range of *Selenops abyssus* encompasses that of several species known from only one sex, as does *Selenops morosus*. More collecting from this region and independent data, such as that of molecular or behavioral data, will be needed to determine which males and females belong together. The male specimen is also in poor condition and the description may not reflect well the coloration and markings of this species.

#### Description.

*Lectotype male*:Color: carapace orange-brown; chelicerae orange-brown; maxillae orange-brown, somewhat darker than dusky yellow; labium orange-brown; abdomen dorsally cream, lighter medially, lanceolate stripe now just an outline, some small flecks, festoon barely distinct, ventrally dusky grey-orange, no markings,; legs dusky yellow, no annulations visible. Carapace:1.10 times longer than broad. Eyes:AER slightly recurved; PER recurved; PME larger than AME, PLE largest, ALE smallest; eye diameters, AME 0.30, ALE 0.18, PME 0.45, PLE 0.50; interdistances AME-PME 0.03, PME-ALE 0.15, ALE-PLE 0.38. PME-PME 1.20. ALE-ALE 2.95; ocular quadrangle AME-AME 0.50, PLE-PLE 2.20; clypeus 0.15 high. Mouthparts:chelicerae with a few stout setae medially and anteriorly; maxillae longer than broad, with tuft of conspicuous setae distally; labium distally rounded. Sternum:1.50 times longer than broad, posteriorly indented. Legs:leg I only slightly shorter than II and III; scopulae present on distal end of all 4 tarsi; tarsi I-IV with strong claw tufts; pr claw toothed, rl claw with fewer teeth; spination: leg I, Fm pr 1–1–1, d 1–1–1, rl 1–1–1; Ti pr 1–0–1, rl 1–0–1, d 1–1–0, v 2–2–2; Mt pr 1–1–0, rl 1–1–0, v 2–2, or pr 1–1–0, rl 1–1–0, v 2–2; leg II, Fm pr 1–1–1, d 1–1–1, rl 1–1–1; Ti pr 1–0–1, d 1–1–0, rl 1–0–1, v 2–2–2; Mt pr 1–1–0, v 2–2, rl 1–1–0; leg III, Fm pr 1–1–1, d 1–1–1, rl 1–1–1; Ti pr 1–0–1, rl 1–0–1, d 1–1–0, v 2–2; Mt pr 1–1–0, rl 1–1–0, v 2–2; leg IV, Fm pr 1–1–1, d 1–1–1, rl 1–1–1; Ti pr 1–0–1, rl 1–0–1, d 1–1–0, v 2–2; Mt pr 1–1–0, rl 1–1–0, v 2–2. Abdomen:without terminal setal tufts. Pedipalp:Fm, spination d 0–1–4;cymbium oval in ventral view, pointed distally, slightly angled posterolaterally; conductor large, attached to bulb on short straight stalk anteromedially, directed laterally, but not extending beyond the edge of cymbium, left side connecting to bulb and forming a circular area between the two conductor connections; embolus long, slender, curved, tapering midway, beginning at 6 o'clock, terminating at 12 o'clock; MA arising at 2 o'clock, directed lateroventrally, with stout triangular base, tapered to a distally hooked process; RTA small, barely reaching cymbium in ventral view, with two processes, in lateral view, the ventral process with a distally pointed process and a process that points downward, the lateral process with a distally pointed structure arising from a quadrangular process (Figs 41–42). *Dimensions:* Total length 11.90. Carapace length 5.95, width 5.56. Sternum length 3.00, width 2.00. Abdomen length 5.95, width 4.35. Pedipalp: Fm 1.75, Pt 0.75, Ti 0.90, Ta 2.00, total 5.40. Leg I: Fm 6.00, Pt 2.00, Ti 5.75, Mt 5.00, Ta 2.50, total 21.25. Leg II: Fm 7.00, Pt 2.00, Ti 6.00, Mt 6.00, Ta 2.75, total 23.75. Leg III: Fm 7.00, Pt 2.00, Ti 6.00, Mt 5.75, Ta 2.75, total 23.50. Leg IV: Fm 7.00, Pt 2.00, Ti 5.75, Mt 5.75, Ta 2.00, total 22.50.

#### Natural history.

Nothing is known of this species' natural history, but based on collection locality data, it seems to span aridland and tropical thornscrub habitats.

#### Distribution.

Known from northwestern México and has an apparently broad distribution from northern Sonora to Nayarit (Map 5).

### Selenops nigromaculatus

Keyserling, 1880

http://species-id.net/wiki/Selenops_nigromaculatus

Figs 43–44 Map 5

Selenops nigromaculatus Keyserling 1880: 230, pl. 6, Fig. 126 (♂, ♀, examined).
Selenops nigromaculatus :F. O. P-Cambridge 1900: 117, pl. 8, Fig. 14 (♂ not ♀).
Selenops nigromaculatus : Mello-Leitão 1918: 30, Figs 6–7 (♂, ♀).
Selenops nigromaculatus :Muma 1953: 44, Figs 76–77 (♂ not ♀).
Selenops santibanezi Valdez-Mondragón 2010: 50, Figs 5–11 (♂, ♀; not examined) syn. n.

#### Type material.

Holotype male: México, E. Simon (1595 BMNH, examined).

#### Notes.

Based on the original illustrations by Valdez-Mondragón (2010: figs 5–11), the holotype male of *Selenops santibanezi* Valdez-Mondragón, 2010, is in every way identical to the holotype male of *Selenops nigromaculatus* Keyserling, 1880. The former species was recorded from the following locality: Santa Catarina Ixtepeji, 17.28°, -96.54496667°, 2021 m, Municipio Santa Catarina Ixtepeji, Distrito Ixtlán, Oaxaca, México, 19.IX.2009, ♀, A. Valdez-Mondragón, R. Paredes, C. Santibáñez (CNAN-T0415), not examined.

#### Diagnosis.

Males can be distinguished from other species by the dorsal branch of the RTA which is a small stalk that abruptly widens to a small quadrangular structure distally (Figs 43–44). Females can be separated from other species by the median lobes that are close together and come together medially, before separating posteriorly (Valdez-Mondragón 2010: figs 10–11).

#### Remarks.

Until very recently (Valdez-Mondragón 2010) there was much confusion regarding this species, in particular the female. The male and female were described by Keyserling (1880). The illustrations are of poor quality and it is difficult to distinguish details from them. F. O. Pickard-Cambridge (1900) produced several beautiful illustrations of the male of *Selenops nigromaculatus*. For unknown reasons Simon didn't show him the female, and unfortunately it was not illustrated. In 1918, Mello-Leitão copied Keyserling's (1880) description and illustration, claiming he had collected a similar female, based on Keyserling's description, in Pará, Brazil. Finally, Muma (1953) was not able to examine the specimen himself, but he had M. Vachon describe and illustrate the male. Apparently, Vachon also illustrated the female, but this was disregarded by Muma because “There is no doubt that Keyserling's female type is the same as *gracilis*, new species," which added to the confusion. Although Keyserling's (1880) illustrations are lacking in certain details, his illustration of the female of *Selenops nigromaculatus* looks nothing like *Selenops gracilis*. Other illustrations of female *Selenops* copulatory organs (e.g. *Selenops mexicanus*) do resemble what the species actually looks like. I have come to two conclusions, both having the same outcome. First, Keyserling (1880) may have illustrated a juvenile. In the vials of *Selenops nigromaculatus* types at the BMNH, there is no adult female. There is a single adult male, which is the type, and, in addition, there is a juvenile spider in another vial labelled as *Selenops nigromaculatus*, from Panamá, Koch collection, with a blank label, numbered 15.3.5.6416. There is no female, which looks like *Selenops gracilis* or otherwise. The second involves the actual female of *Selenops nigromaculatus* being lost. Luckily, a male of *Selenops nigromaculatus* was collected with a female, described and beautifully illustrated by Valdez-Mondragón (2010). The specimens were described as *Selenops santibanezi*. The male type and illustration by Valdez-Mondragón (2010) clearly reveal these are the same species, and thus, *Selenops santibanezi* is a junior synonym of *Selenops nigromaculatus*.

There also appear to be some erroneous locality records. Mello-Leitão's (1918) collection of a female in Pará, Brazil is dubious, given that the actual specimen was not illustrated, and that it resembled Keyserling's description. In addition, the thorough work on South American selenopids by (Corronca (1996, 1998) has turned up no specimens of *Selenops nigromaculatus*. Finally, it is mentioned by Simon (1880) and Mello-Leitão (1918) that this spider was found in Antigua by Grenadier. This particular specimen has not been located. This could refer to the vicinity of Antigua, Guatemala, rather than the Caribbean island nation of Antigua and Barbuda. The type locality is México and a different species has been found in Antigua and Barbuda which is not closely allied with *Selenops nigromaculatus*.

#### Description.

*Holotype male:* Color:carapace uniformly brownish-red; sternum orange-brown; chelicerae orange-brown; maxillae brown, lightening distally; labium light brown lightening distally; abdomen dorsally orange-yellow, no markings present any longer, remnants of festoon visible; ventrally orange-yellowish. Carapace: 0.86 times longer than broad; fovea longitudinal, broad, very shallow. Eyes:AER nearly straight; PER slightly recurved; PME larger than AME, PLE largest, ALE smallest; eye diameters, AME 0.30, ALE 0.08, PME 0.40, PLE 0.50; interdistances AME-PME 0.03, PME-ALE 0.10, ALE-PLE 0.68. PME-PME 1.65. ALE-ALE 2.50; ocular quadrangle AME-AME 0.60, PLE-PLE 2.70; clypeus 0.15 high. Mouthparts:chelicerae with stout setae, maxillae longer than broad, with tuft of conspicuous setae distally; labium distally rounded. Sternum:1.09 times longer than broad, posteriorly indented. Legs:leg I only slightly shorter than legs II, III and IV; leg formula 3241 (Muma, 1953); scopulae present on tarsi of all legs and on metatarsi of legs I and II; tarsi I-IV with strong claw tufts on all legs; both claws with around same number of teeth; spination: leg I, Fm pr 1–1–1, d 1–1–1, rl 1–1–1; Ti pr 1–0–1, d 0, rl 1–0–1, v 2–2–2; Mt v 2–2; leg II, Fm pr 1–1–1, d 1–1–1, rl 1–1–1; Ti pr 1–0–1, d 0, rl 1–0–1, v 2–2–2, Mt v 2–2; leg III, Fm pr 1–1–1, d 1–1–1, rl 1–1–1; Ti pr 1–0–1, rl 1–0–1, v 2–2; Mt v 2–2; leg IV, Fm pr 1–1–1, d 1–1–1, rl 1–1–1; Ti pr 1–1–0, v 2–2–0, rl 1–1–0; Mt v 2–2. Abdomen:without terminal tufts of setae. Pedipalp:Fm, spination dorsal 0–1–4; cymbium oval to round in ventral view, angled posterolaterally; scopulae scattered, denser toward tip; conductor arising from side and center of bulb, quadrangular; embolus long, blade like, narrow, begins at 7 o'clock, ends at 12 o'clock, does not curve around edge of cymbial margin, but instead directed anteriorly; MA short with stout base and longer hooked finger-like process, arising at 2 o'clock, directed laterally; RTA extending at least 1/4 the length of cymbium in ventral view; RTA with 2 apophyses, the lateral process is a stout stalk with a distal quadrangular process, the ventral process is smaller, blunt and curved; basal cymbial process absent (Figs 43–44). *Dimensions:* Total length 13.20. Carapace length 5.90, width 6.90. Sternum length 3.00, width 2.75. Abdomen length 7.30, width 5.25. Pedipalp: Fm 1.75, Pt 0.75, Ti 1.00, Ta 1.50, total 5.00. Leg I: Fm 7.00, Pt 3.00, Ti 6.50, Mt 6.00, Ta 2.50, total 25.00. Leg II: Fm 8.00, Pt 3.00, Ti 7.00, Mt 6.65, Ta 2.50, total 27.15. Leg III: Fm 8.00, Pt 2.50, Ti 7.00, Mt 6.50, Ta 2.25, total 26.75. Leg IV: Fm 7.75, Pt 2.50, Ti 6.75, Mt 6.75.

*Female*: See Valdez-Mondragón (2010: under *Selenops santibanezi*).

#### Natural history.

This species has been found in bromeliads growing on oak trees at 2000 m elevation (Valdez-Mondragón 2010).

#### Distribution.

Unfortunately I only know the type to be from México, but it is also possible this species is found near Antigua, Guatemala (see Remarks). Other specimens, including the female, are from Oaxaca (Map 5).

### Selenops makimaki sp. n.

urn:lsid:zoobank.org:act:C9D8E820-9627-414F-8BC3-E9737AF19542

http://species-id.net/wiki/Selenops_makimaki

Figs 45–46 Map 5

#### Type material.

Holotype female: 21 km west of Rizo de Oro, along ridge, southeast of Cerro El Bejucal, near Chiapas border, Oaxaca, México, 8.I.1973, 1615 m, within epiphytic *Vriesia* sp., K.E. Lucas (CAS).

#### Other material examined.

MÉXICO: Oaxaca: same data as holotype, 1♀, 1 imm.

#### Etymology.

This species is named after the word for spider, makimaki, in the indigenous Chimalapas Zoque language from the region of the type locality. The name is to be treated as a noun in apposition.

#### Diagnosis.

**Figures 45–52.**
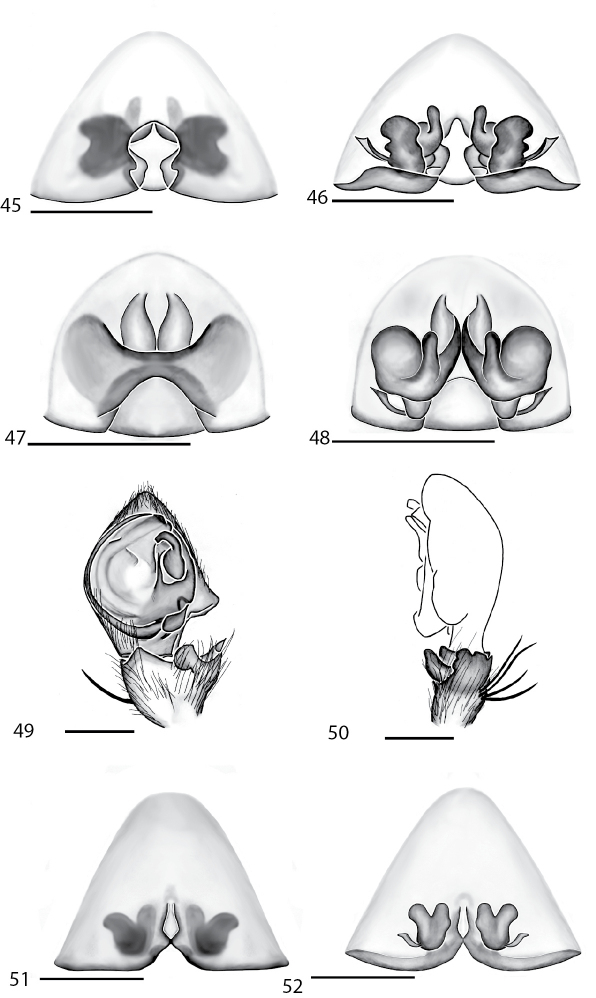
Copulatory organs of *Selenops makimaki* sp. n. holotype female from 21 km west of Rizo de Oro, along ridge, southeast of Cerro El Bejucal, near Chiapas border, Oaxaca, México (CAS) **45–46** *Selenops scitus* Muma holotype female from Mexcala, Guerrero, México (AMNH) **47–48** male from 22 miles west of Puerto Escondido, Oaxaca, México (CAS) **49–50**, *Selenops ixchel* sp. n. holotype female from Cozumel, Quintana Roo, México (CAS) **51–52,** **45, 47, 51** epigyne, ventral view **46, 48, 52** spermathecae, dorsal view **49** left pedipalp, ventral view **50** left pedipalp, retrolateral view. Scale bar = 0.50 mm.

This species can be distinguished from all other species by the presence of a thin median septum, while the region surrounding the median septum is more heavily sclerotized, giving the appearance of a keyhole-shaped opening (Fig. 45). Males unknown.

#### Description.

*Holotype female:* Color:carapace yellow, slightly dusky laterally; sternum yellow, darker around border; chelicerae yellow, dusky u-shaped markings occurring midway down; maxillae pale yellow; labium pale yellow; abdomen dorsally dusky yellow, indications of lanceolate stripe, about 3/4 of the way down the length of the abdomen, and two w-shaped chevrons expending to lateral margins, also a dark spot anteromedially, and some white areas present, festoon present; ventrally dusky yellow-grey, no markings; legs yellowish, very faint annulations present, most prominent on tibiae just below patellae of all legs, especially noticeable in legs I and II, anterolateral faces of legs with dark markings, horizontal stripe, prominent on femora I and II, less prominent on legs III and IV, though still visible. Carapace: as long as broad; fovea longitudinal, broad, very shallow. Eyes:AER nearly straight; PER slightly recurved; PME larger than AME, PLE largest, ALE smallest; eye diameters, AME 0.15, ALE 0.08, PME 0.18, PLE 0.23; interdistances AME-PME 0.06, PME-ALE 0.10, ALE-PLE 0.30. PME-PME 1.03. ALE-ALE 1.63; ocular quadrangle AME-AME 0.40, PLE-PLE 1.83; clypeus 0.08 high. Mouthparts:chelicerae with a few stout setae medially and anteriorly; maxillae longer than broad, with tuft of conspicuous setae distally; labium distally rounded. Sternum:0.92 times longer than broad, posteriorly indented. Legs:leg I only slightly shorter than II and III; leg formula 2314; scopulae present on all 4 tarsi and metatarsi and tibiae I and II; tarsi I-IV with strong claw tufts; pr claw toothed, rl claw with fewer teeth; spination: leg I, Fm pr 1–1–0, d 1–1–1, rl 1–0–0; Ti d 0, ventral 2–2–2; Mt v 2–2; leg II, Fm pr 1–0–0, d 1–1–1, rl 0; Ti v 2–2–2; Mt v 2–2; leg III, Fm pr 1–0–0, d 1–1–1, rl, 0–0–1; Ti v 1–1–0; Mt v 2–0; leg IV, Fm pr 0, d 1–1–1, rl 0–0–1; Ti v 1–1; Mt v 2–0. Abdomen:with terminal setal tufts. Pedipalp:claw present, with ca. 6 teeth. Epigyne:median septum only slightly sclerotized, coupled with more sclerotized lateral lobes, giving the appearance of a keyhole-shaped opening medially on the epigynal plate, genital openings located laterally at edges of septum; internally, ducts are located laterally, with anteriorly directed finger-like extensions, as well as wider sinuous structures, fertilization ducts located laterally, directed anteriorly, posterodorsal fold present, but barely covers posterior edge of ducts (Figs 45–46). *Dimensions:* Total length 7.65. Carapace length 3.88, width 3.85. Sternum length 1.90, width 1.75. Abdomen length 3.78, width 2.70. Pedipalp: Fm 1.00, Pt 0.50, Ti 0.75, Ta 0.90, total 3.15. Leg I: Fm 4.00, Pt 1.65, Ti 3.00, Mt 2.60, Ta 1.25, total 12.50. Leg II: Fm 4.60, Pt 1.60, Ti 3.50, Mt 3.00, Ta 1.00, total 13.70. Leg III: Fm 4.50, Pt 1.50, Ti 3.50, Mt 3.00, Ta 1.00, total 13.50. Leg IV: Fm 4.00 Pt 1.25, Ti 3.00, Mt 2.50, Ta 1.00, total 11.75.

#### Natural history.

This species has been found within epiphytic *Vriesia* sp.

#### Distribution.

Known only from the type locality (Map 5).

### Selenops scitus

Muma, 1953

http://species-id.net/wiki/Selenops_scitus

Figs 47–50 Map 5

Selenops scitus Muma 1953: 19, Fig. 32 (♀, examined).
Selenops scitus :Valdez-Mondragón 2010: 53, Figs 12–18 (♂).

#### Type material.

Holotype female: Mexcala, Guerrero, México, VIII.1946, C.J. Goodnight (AMNH, examined).

#### Other material examined.

MÉXICO: Guerrero: Mexcala, 2.VII.1941, L.I. Davis, 1♀ (AMNH); **Oaxaca:** 22 miles west of Puerto Escondido, palm forest, on low shrubs and fallen fronds, 1♂ (CAS).

#### Diagnosis.

Females can be separated from others by the lateral lobes which have the appearance of being fused and are pinched medially, and internally, the spermathecae are large and round, with two long, finger-like anteriorly directed processes (Figs 47–48). Males can be separated from other species by the large MA, located at 3 o'clock, the inconspicuous conductor, and the laterally angled cymbium (Figs 49–50).

#### Description.

*Male*: Color:carapace orange-brown, some dusky markings medially and laterally; sternum pale yellow; chelicerae orange-brown, with some dusky markings; maxillae dusky yellow-brown, lightening distally; labium grey-brown, lightening distally; abdomen dorsally yellow-brown with dark specks, faded; ventrally pale yellow, no markings; legs light orange-brown, annulations present, mottling on anterolateral faces of femora. Carapace: 0.99 times longer than broad; fovea longitudinal, broad, very shallow. Eyes:AER slightly recurved; PER recurved; PME larger than AME, PLE largest, ALE smallest; eye diameters, AME 0.13, ALE 0.05, PME 0.3, PLE 0.38; interdistances AME-PME 0.05, PME-ALE 0.14, ALE-PLE 0.33. PME-PME 0.88, ALE-ALE 1.58; ocular quadrangle AME-AME 0.28, PLE-PLE 1.58; clypeus 0.13 high. Mouthparts:chelicerae with stout setae medially and anteriorly; maxillae longer than broad, with tuft of conspicuous setae distally; labium distally rounded. Sternum:as long as broad, posteriorly indented. Legs: leg formula 42,3=1; scopulae present on distal end of all 4 tarsi; tarsi I-IV with strong claw tufts; pr claw toothed, rl claw with fewer teeth; spination: leg I, Fm pr 1–1–1, d 1–1–1, rl 1–1–1; Ti d 1–1–0, pr 1–0–1, rl 1–0–1, v 2–2–2; Mt pr 1–1–0, v 2–2–0, rl 1–0–0; leg II, Fm pr 1–1–1, d 1–1–1, rl 1–1–1; Ti pr 1–0–1, rl 1–0–1, v 2–2–2; Mt pr 1–0–0, v 2–2, rl 1–1–0; leg III, Fm pr 1–1–1, d 1–1–1, rl 1–1–1; Ti pr 1–0–1, rl 1–0–1, v 2–2; Mt pr 1–0–0, rl 1–0–0, v 2–1; leg IV, Fm pr 1–1–1, d 1–1–1, rl 1–1–1; Ti pr 1–0–1, rl 1–1–1, v 2–2–2; Mt pr 1–0–0, rl 1–0–0, v 2–2. Abdomen:without terminal setal tufts. Pedipalp:Fm spination d 0–1–4; Cymbium oval in ventral view, angled posterolaterally; basal cymbial process absent; scopulae scattered, denser distally; conductor arises laterally and distally, angular distally; embolus very long, curved around the margin of the cymbium, originating at 5 o'clock, ending at 1 o' clock; MA with a short stout, slightly rounded base with long, hooked, finger-like process, truncate distally, MA arising at 3 o'clock, directed distally; RTA with 2 processes, both about the same size, lateral apophysis indented on distal margin and quadrangular, ventral process rounded distally; RTA barely reaching cymbium in ventral view (Figs 49–50). *Dimensions:* Total length 7.43. Carapace length 3.35, width 3.40. Sternum length 1.65, width 1.65. Abdomen length 4.08, width 2.23. Pedipalp: Fm 1.00, Pt 0.25, Ti 0.40, Ta 1.00, total 2.65. Leg I: Fm 3.75, Pt 1.35, Ti 3.65, Mt 3.65, Ta 1.75, total 14.15. Leg II: Fm 4.00, Pt 1.00, Ti 3.75, Mt 3.75, Ta 1.75, total 14.25. Leg III: Fm 4.00, Pt 1.00, Ti 3.65, Mt 3.75, Ta 1.75, total 14.15. Leg IV: Fm 5.00, Pt 5.00, Ti 1.00, Mt 4.00, Ta 1.90, total 16.9.

*Holotype female*:Color:carapace brown, darker laterally with white setae; sternum yellow-brown; chelicerae brown, darker laterally; maxillae brown, lightening distally; labium brown, lightening distally; abdomen damaged, grey-brown with some darker flecks, damage precludes visualization of patterns; legs light brown with darker annulations. Carapace:0.90 times longer than broad; fovea longitudinal, broad, shallow. Eyes: AER nearly straight; PER recurved; PME larger than AME, PLE largest, ALE smallest; eye diameters, AME 0.10, ALE 0.08, PME 0.20, PLE 0.28; interdistances AME-PME 0.03, PME-ALE 0.08, ALE-PLE 0.40. PME-PME 0.80. ALE-ALE 1.45; ocular quadrangle AME-AME 0.30, PLE-PLE 1.48; clypeus 0.05 high. Mouthparts:chelicerae with a few stout setae medially and anteriorly; maxillae longer than broad, with tuft of conspicuous setae distally; labium distally rounded. Sternum:as long as broad, posteriorly indented. Legs:leg formula 4321; scopulae present on distal end of all 4 tarsi; tarsus I-IV with strong claw tufts; pr claw toothed, rl claws with fewer teeth; spination: leg I, Fm pr 1–1–0, d 1–1–1, rl 1–1–1; Ti v 2–2–2; Mt v 2–2; II, Fm pr 1–0–0, d 1–1–1, rl 1–1–0; Ti v 2–2–2; Mt v 2–2; III, Fm pr 1–1–0, d 1–1–1, rl 1–1–1; IV, Fm pr 1–0–0, d 1–1–1, rl 1–1–1; Ti v 1–1; Mt v 2–2 Abdomen:without terminal setal tufts. Pedipalp:claw with ca.10 teeth. Epigyne:lateral lobes appearing fused medially, pinched forming an anterior u-shape and a posterior arch, genital openings located anteriorly; internally, ducts located medially, nearly touching, extending laterally, spermathecae large and round, with long anteriorly directed finger-like processes located medially, fertilization ducts located posterolaterally, directed anteriorly, posterodorsal fold absent (Figs 47–48). *Dimensions:* Total length 5.65. Carapace length 2.70, width 3.00. Sternum length 1.40, width 1.00. Abdomen length 2.95, width 2.60. Pedipalp: Fm 0.65, Pt 0.25, Ti 0.20, Ta 0.75, total 1.85. Leg I: Fm 2.75, Pt 0.90, Ti 2.00, Mt 1.75, Ta 0.70, total 8.10. Leg II: Fm 3.80, Pt 1.00, Ti 2.30, Mt 2.00, Ta 1.00, total 10.10. Leg III: Fm 3.00, Pt 0.80, Ti 2.50, Mt 2.50, Ta 0.90, total 9.70. Leg IV: Fm 3.00, Pt 0.80, Ti 2.50, Mt and Ta missing.

#### Natural history.

Collected in deciduous thorn scrub (Valdez-Mondragón 2010), as well as in a coastal palm forest on the ground in fallen fronds.

#### Distribution.

Southern México from Guerrero to Oaxaca (Map 5).

### Selenops ixchel sp. n.

urn:lsid:zoobank.org:act:4402E23A-5D8E-410A-AEB5-050D6B7BE313

http://species-id.net/wiki/Selenops_ixchel

Figs 51–52 Map 5

#### Type material.

Holotype female: Cozumel, Quintana Roo, México, 25-27.I.1984, V. Roth (CAS).

#### Etymology.

The specific epithet comes from the name of the Mayan goddess Ix Chel for whom the type locality of Cozumel is sacred. It is to be treated as a noun in apposition.

#### Diagnosis.

Females can be distinguished from other species by the median septum which is very narrow and somewhat diamond shaped, and the v-shaped internal ducts (Figs 51–52). Males unknown.

#### Description.

*Holotype female:* Color:carapace brownish-red with some dusky markings; sternum light brown, darker around border; chelicerae uniformly brownish-red; maxillae light orange-brown, lightening distally; labium light brown lightening distally; abdomen dorsally yellowish-grey, darkening distally, remnants of a faded lanceolate stripe, foliate pattern starting about halfway down, slight festoon; ventrally yellowish, dark on sides, at bottom; legs orange-brown, darkening to brown distally, annulations prominent. Carapace: 1.08 times longer than broad; fovea longitudinal, broad, very shallow. Eyes:AER nearly straight; PER slightly recurved; PME larger than AME, PLE largest, ALE smallest; eye diameters, AME 0.23, ALE 0.08, PME 0.38, PLE 0.45; interdistances AME-PME 0.06, PME-ALE 0.18, ALE-PLE 0.45. PME-PME 1.35. ALE-ALE 2.16; ocular quadrangle AME-AME 0.53, PLE-PLE 2.38; clypeus 0.17 high. Mouthparts:chelicerae with stout setae medially and anteriorly; maxillae longer than broad, with tuft of conspicuous setae distally; labium distally rounded. Sternum:1.38 times longer than broad, posteriorly indented. Legs:leg I only slightly shorter than legs II, III and IV; leg formula 42=31; scopulae present on all 4 tarsi and metatarsi and tibiae I and II; tarsus I-IV with strong claw tufts; pr claw toothed, rl claw with fewer teeth; spination: leg I, Fm pr 1–1–0, d 1–1–1, rl 1–1–1; Ti d 0, v 2–2–2; Mt v 2–2; leg II, Fm pr 1–1–0, d 1–1–1, rl 1–1–1; Ti v 2–2–2; Mt v 2–2; leg III, Fm pr 1–1–0, d 1–1–1, rl 1–1–1; Ti (R) 2–2–1, (L) 2–1–1; Mt v 2–1; leg IV, Fm pr 1–1–0, d 1–1–1, rl 1–1–1; Ti v 2–2–0; Mt v 2–1. Abdomen:without terminal setal tufts. Pedipalp:claw with 13 teeth. Epigyne:very narrow median septum, somewhat diamond shaped, widening posteriorly, genital openings located at lateral margins of median septum; internally, ducts are v-shaped, located laterally, fertilization ducts located posteriorly, directed anterolaterally, small posterodorsal fold present, but does not cover any part of the internal ducts (Figs 51–52). *Dimensions:* Total length 11.48. Carapace length 4.65, width 4.33. Sternum length 2.50, width 1.87. Abdomen length 6.83, width 4.38. Pedipalp: Fm 1.00, Pt 0.50, Ti 0.75, Ta 1.50, total 3.75. Leg I: Fm 3.50, Pt 2.80, Ti 4.00, Mt 3.50, Ta 1.50, total 15.30. Leg II: Fm 5.00, Pt 2.00, Ti 4.00, Mt 3.00, Ta 1.75, total 15.75. Leg III: Fm 5.00, Pt 1.75, Ti 4.00, Mt 3.50, Ta 1.50, total 15.75. Leg IV: Fm 5.00, Pt 1.35, Ti 4.00, Mt 3.75, Ta 1.75, total 15.85.

#### Natural history.

No data.

#### Distribution.

Known only from the type locality (Map 5).

##### Selenops debilis group (sensuMuma 1953)

**Map 7.**
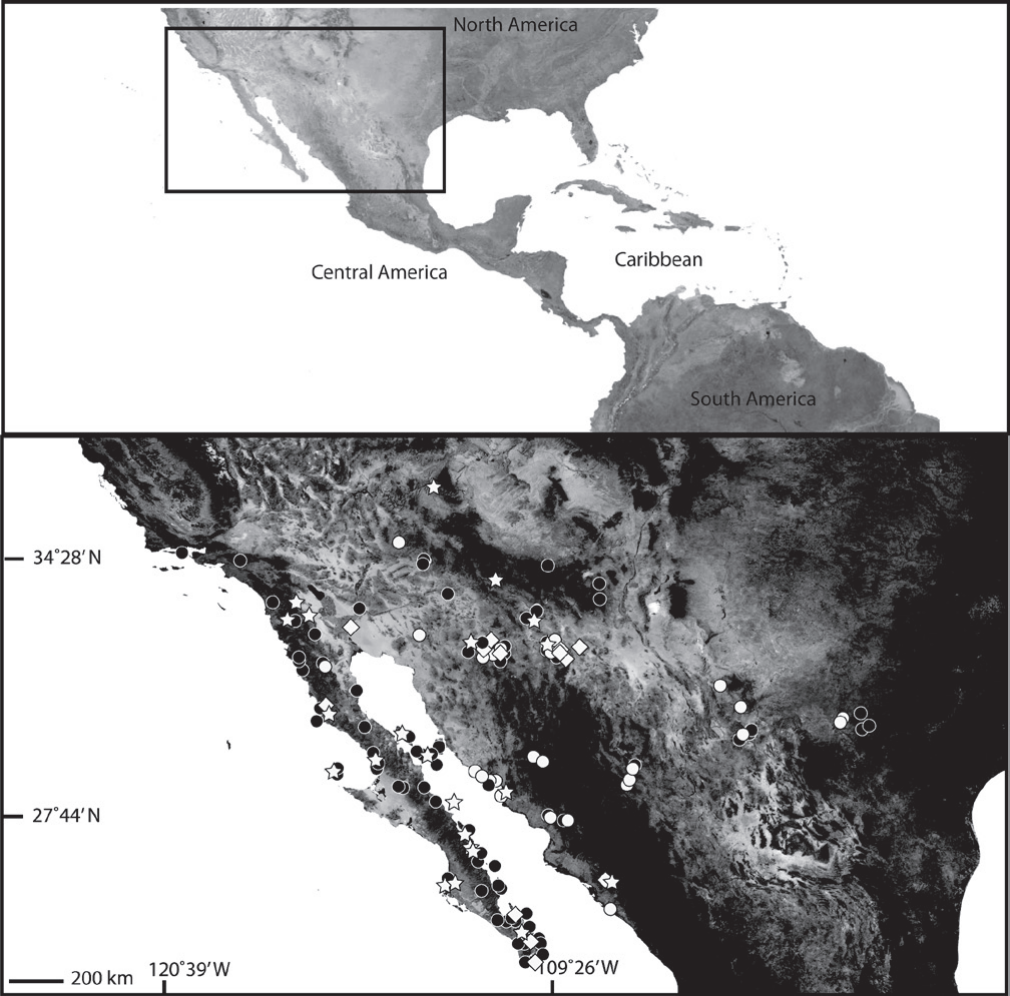
Map of North America, including the United States and México (inset) showing the distribution of some members of the *Selenops debilis* group (*sensu* Muma 1953), including those than cannot be identified to species (black circles) either because they are juveniles or they have diagnostic characteristics of more than one species (see text), *Selenops actophilus* Chamberlin (white circles), *Selenops debilis* Banks (white diamonds), and *Selenops nesophilus* Chamberlin (white stars).

The following three descriptions are of the spiders that belong to what Muma (1953) called the *Selenops debilis* group. These spiders were all described nearly 100 years ago, and the male and female types of *Selenops debilis* and *Selenops actophilus* are not from the same locality (though collecting from the same locality does not guarantee the same species as known in other species). Additionally, the three species have overlapping distributions in the desert southwest of North America and all three may be found in a single locality (Fig. 198, Map 7). Finally, there also appears to be genitalic variation away from the types, and several specimens have been found that share morphological features of more than one species. DNA data indicates at least three species in the region (Crews and Gillespie 2010). Morphologically determining which specimen belongs to which species is a daunting task, and in some instances is not possible at the present time. Unfortunately recent collections did not contain adults. At this time, no taxonomic changes are made. Below, the types are re-described and locality data are included only when a specimen could, with reasonable certainty, be assigned to a particular species (i.e., when they did not differ from the type). Additional locality records are included below for members of the *Selenops debilis* group that cannot be sorted to species. Variations of the internal female copulatory organs are found in Figs 65–68. In order to sort out the variation and uncertainty in this group, to determine the correct number of species, and to learn more about biogeography and possible hybridization, this group is in need of revision. This will include the collection and analysis of molecular, morphometric, and behavioral data.

Unsorted records. MÉXICO: Baja California: 0.5 mi S Las Virgenes, 2000', 9.IV.1969, S.C. Williams, 1 imm. (CAS); 2 mi S El Rosario on Mex Hwy 1 (dirt road) RB-3, 27.XI.1962, P.R. Craig, D. Dailey, 2 imm. (CAS); Isla Cedros, 14.III.1945, B.B. Osorio, 1 imm. (AMNH); Isla Cedros, Gran Cañon, 10.III.1945, B.O. Tafall, 2 imm. (AMNH); 42 miles south of Ensenada, 28.IV.1961, W.J. Gertsch, 1 imm. (AMNH); Isla San Esteban, 1.IV.1953, B. Firstman, 1 imm. (AMNH); Puertocitos, on wall of cabaña, 10.VI.1968, S.C. Williams, M.M. Bentzein, no abdomen (CAS); 1 mile north of Miller's Landing, 28.30 N, 114.00 W, 24.II.1966, V. Roth, 1 imm. (AMNH); Isla San Lorenzo, northern island, 16.III.1971, V.F. Lee, 3 imm. (CAS); Isla Cedros, Punta Norte, 15 m, 3.VII.1983, V.F. Lee, 1♀ (CAS); 44.1 mi N by Mex Hwy 1, of junction with road to Bahia de Los Angeles, 19.VII.1977, R. Seib, 1 imm. (CAS); Sierra Juarez, Tajo-Cantil Canyon system, 14-18.IV.1973, S.C. Williams, 1 imm. (CAS); Isla Raza, 31.III.1981, S.C. Williams, 1 imm. (CAS); Isla Cedros, trail from El Pueblo de Cerre de Cetros, 180 m, 26.IX.1984, D.B. Weissman, V.F. Lee, 1 exuvium (CAS); 11 mi SW Punta Prieta, 200', 15.IV.1969, S.C. Williams, 1 imm. (CAS); Isla Raza, 15.III.1971, V.F. Lee, 3 imm. (CAS); 10 mi N Domingo Landing, 27.VI.1938, A.E. Michelbacher, E.S. Ross, 1 imm. (CAS); 13.2 mi S of El Rosario on Mex Hwy 1, dirt road RB-3, 29.XI.1962, P.R. Craig, D.L. Dailey, 1 imm. (CAS); Isla Sal Si Puedes, 15.III1971, V.F. Lee 4 imm. (CAS); Isla San Jéronimo, 1.VI.1944, B. Osorio, several (AMNH). **Baja California Sur:** Bahia de Los Muertos, 20.XII.1958, H.B. Leech, in rotting *Pachycereus calva*, 1 imm. (CAS); Boca de la Sierra, near Miraflores, 23.20 N, 109.45 W, 10.II.1966, V. Roth, 1♂, 1 imm. (AMNH); Isla Carmen, 21.V.1921, J.C. Chamberlin, 1 imm. (CAS); La Paz, 25', 30.IV.1969, S.C. Williams, 1 imm. (CAS); 44 km W La Paz at 0.2 km S km 44 on Mex Hwy 1, 31.XII.1978, D. Weissman, R. Love, V. Lee, C. Mullinex, 1 imm. (CAS); 1 km W La Burrera on road to La Burrera, 2.I.1979, D. Weissman, R. Love, C. Mullinex, V.F. Lee, 2 imm. (CAS); Isla San Ildefonso, 30.III.1953, J.P. Figg-Hoblyn, 2 imm. (AMNH); Isla San Diego, west side, 25.III.1971, V.F. Lee, 1 imm. (CAS); Isla Angel de la Guarda, North of Punta Rocosa, 14.III.1971, V.F. Lee, 2 imm. (CAS); Isla San Jose, near boy's prison, 25.III.1953, B. Firstman, 2 imm. (AMNH); Isla Ceralvo, El Mostrador, river bed, 22.III.1953, B. Firstman, under rocks, 2 imm. (AMNH); Isla Santa Catalina, SW end at navigation light, 21.V.1970, S.C. Williams, V.F. Lee, 1 imm., 1p♂ (CAS); 3 miles west El Triunfo, 23.45 N, 110.00 W, 11.II.1966, V. Roth, 1 imm. (AMNH); La Paz, 1-3.II.1965, V. Roth, 1 imm. (AMNH); 20 miles southeast of El Arco, 19.I.1965, V. Roth, 1 imm. (AMNH); 3.8 mi S of the Santa Maria Sky Ranch on Mex Hwy 1, dirt road RB-3, 25.XI.1963, P.R. Craig, D. Dailey, 1 imm. (CAS); Las Barracas, 30 km ESE Santiago, IV.1982, P. DeBach, 2♀ (CAS); Rancho Centenario, west of La Paz on road to Los Aripes, 10.IX.1963, P.R, D.L. Craig, R.A. Gates, 1♂, 1♀, 1 imm. (CAS); 1.3 miles NW El Triunfo, 20.I.1959, 3 imm. (CAS); 3 mi NW San Antonio, 13-18.XII.1977, C.E. Griswold, L. Vincent, 1 imm. (CAS); Isla San Esteban, 19.IV.1921, J.C. Chamberlin, 1 imm. (CAS); La Ribera, 23.30°N, 109.30°W, 10.II.1966, V. Roth, 2 imm. (AMNH); San Jose del Cabo, 23.III.1945, M. Correa, 1 imm. (AMNH); Isla Magdalena, Puerto Magdalena, 17.III.1957, R. Zweifel, 1 imm. (AMNH); Isla Danzante, 24.V.1921, J.C. Chamberllin, 1p♂ (MCZ); Bahia Agua Verde, 26.V.1921, J.C. Chamberlin, 2 imm. (MCZ); Isla San Esteban, 19.IV.1921, J.C. Chamberlin, 2 imm. (MCZ); Isla Sal Si Puedes, 9.V.1921, J.C. Chamberlin, 2 imm. (MCZ); Isla Sal Si Puedes, 21.V.1962, R.E, A.E. Ryckman, C.P. Christianson, 1 imm. (AMNH); Misión San Luis Gonzaga, 25 N, 111.15 S, 14.II.1966, V. Roth, 1p♂ (AMNH); Bahia de Los Angeles, 25.VI.1921, J.C. Chamberlin, 2 imm. (MCZ); Isla San Pedro Martir, 18.IV.1921, J.C. Chamberlin, 2 imm. (CAS); Cabo San Lucas, 22.VII.1969, M. Cazier, S.C. Williams, 1 imm. (CAS); Isla Cerralvo, Bahia Limoña, 17.V.1970, S.C. Williams, V.F. Lee, 2 imm. (CAS); La Paz, 50', 12.X.1968, E.L. Sleeper, F.J. Moore, 1 imm. (CAS); Punta Trinidad, cobblestone beach, 20.III.1971, V.F. Lee, 1 imm. (CAS); 44 km S Guerrero Negro, turnoff at km 171 on Mex Hwy 1, 29.XII.1978, D. Weissman, R. Love, V. Lee, C. Mullinex, 1 imm. (CAS); La Paz, Arroyo de Palo, off airport road, 7.IX.1963, P.R. Craig, E. Warington, 1 imm. (CAS); Sierra La Laguna, 17 air mi ENE Todos Santos, 6000', 12-18.XII.1979, C.E. Griswold, 1♂, 7 imm. (CAS); Sierra La Laguna, 1♀ (USNM); 2.8 mi SSE of Todos Santos, 25.XII.1958, H.B. Leech, beating dead leaves of living *Yucca valida*, 3 imm. (CAS); 5.9 mi N of Todos Santos, 24.VII.1968, Williams, Fox, Bentzion, under dead vegetation, 1♀ (CAS); Cabo San Lucas, 5.III.1928, T. Craig, 1 imm. (CAS); Isla Carmen, Puerto Balandra, 23.III.1971, V.F. Lee, 1 imm. (CAS); Santiago, in house, 15-18.VIII.1964, H.W. Campbell, 1 imm. (CAS). **Chihuahua:** Tejaban, 11-15.V.1991, R.E. Stecker, 1♂ (CAS). **Sonora:** Álamos, Guaymas, 9.IV.1921, E.P. Van Duzee, 1 imm. (AMNH); Guaymas, 14.IV.1921, J.C. Chamberlin, 1 imm. (AMNH); 25 km southwest Navojoa, 22.VII.1954, R.E. Ryckman, C. P. Christianson, 1 imm. (AMNH); Guaymas, 14.IV.1921, J.C. Chamberlin, 1 imm. (MCZ); Guaymas, II.1953, A. Ebeling, 1 imm. (AMNH); East Side of Sierra Alamos, 12.XI.1972, V. Roth, 3 imm. (AMNH); Isla Tiburon, 3.VII.1921, J.C. Chamberlin, 1 p♂ (MCZ); Isla San Pedro Martir, south end, 17-18.III.1971, V.F. Lee, 1 imm. (CAS). **UNITED STATES: Arizona:** Apache Co., Southfork, 6-13.V.1956, M. Statham, several (AMNH); Cochise Co., Portal, 8.VI.1964, V. Roth, 1♂, 1♀ (AMNH); Cochise Co., Portal, 21.V.1985, W.J. Gertsch, in house, 1♂ (AMNH); Cochise Co., Portal, 1.VI.1993, V. Roth, in building, 1♂ (AMNH); Cochise Co. SWRS, 5 mi West of Portal, 20-23.V.1972, D.C. Rentz, 1♀ (CAS); Cochise Co., Portal, 12.VI.1923, W.J. Gertsch, 1♂ (AMNH); Cochise Co., Portal, 28.V.1973, W.J. Gertsch, 1♂ (AMNH); Cochise Co., Portal, 16-30.V.1964, 1♂, 1♀ (AMNH); Cochise Co., South fork Cave Creek, Chiricahua Mountains, 11.IX.1950, W.J. Gertsch, 3 imm. (AMNH); Cochise Co., 1 mile west of Portal, 10.IX.1950, W.J. Gertsch, 3 imm. , 2p♂ (AMNH); Cochise Co., Portal, in house, 14.VI.1974, 1♂ (AMNH); Cochise Co., Portal, 13.V.1972, W.J. Gertsch, in house, 1♂ (AMNH); Cochise Co., west of Portal, Painted Canyon Ranch, 20.VI.1954, M.A. Cazier, several (AMNH); Cochise Co., 5 miles southeast San Simone, 21.V.1956, E. Ordway, 1♂ (AMNH); Cochise Co., Portal, V.1969, W. J. Gertsch, several (AMNH); Cochise Co., SWRS, 5 miles west of Portal, V.1962, W.J. Gertsch, 1♂ (AMNH); Cochise Co., Portal, VI.1965, W.J. Gertsch, 1♂ (AMNH); Cochise Co., SWRS, 5 miles west of Portal, 26.VI.1955, M. Statham, 2♂ (AMNH); Cochise Co., SWRS, 5 miles west of Portal, 15.VI.1955, M. Statham, 2♀ (AMNH); Cochise Co., Portal, Chiricahua Mountains, 1.VI.1952, several (AMNH); Cochise Co., Portal, matured V.1965, V. Roth, 1♂ (AMNH); Cochise Co., SWRS, 5 miles west of Portal, 6-20.VII.1955, W.J. Gertsch, several (AMNH); Cochise Co., 2 miles west of Chiricahua National Monument, 6000-6400', 27.VIII.1951, T. Cohn, 3 imm. (AMNH); Cochise Co., Fort Bowie, V.1973, V. Roth, 1♂, 1 imm. (AMNH); Cochise Co., Guadelupe Canyon, 28 miles east of Douglas, 9.VI.1972, G. Dingerkus, 1♂ (AMNH); Cochise Co., Portal, 14.VI.1972, W.J. Gertsch, 1♀ (AMNH); Cochise Co., Portal, Herb Martyr Dam, 7.VI.1972, G. Dingerkus, 1♀ (AMNH); Cochise Co., Portal, 12.X.1953, M.A. Cazier (AMNH); Cochise Co., Portal, 12.XII.1967, W. J. Gertsch, 1♂ (AMNH); Cochise Co., 3 miles west of Portal, VII.1963, J. Woods, 1♀; Cochise Co., Portal, 10.VI.1971, in house, 1♂ (AMNH); Cochise Co., West of Portal, Painted Canyon, Ranch, 4.VII.1954, W.J. Gertsch, 1♂ (AMNH); Maricopa Co., South Mountain Park, pit trap 61-80, 24.X.1965, S.C. Willilams, 2 imm. (CAS); Maricopa Co., South Mountain Park, pit trap 81-100, 6.XII.1964, S.C. Williams, 1 imm. (CAS); Pima Co., near Tucson, Upper Sabmino Canyon, 24.III.1960, W.J. Gertsch, W. Ivie, R. Schrammel, under rocks, dry hillside, 1 imm. (AMNH); Pima Co., Tucson, 20.III.1940, R.H. Crandall, 3 imm. (AMNH); Pima Co., Baboquivari Mountains, Rancho El Mirador, 4.IX.1950, W. J. Gertsch, 2♀, 3 imm. (AMNH); Pima Co., Baboquivari Canyon, west side of Baboquivari Mountains, 25-27.V.1952, H.B. Leech, J.W. Green, 1 imm. (CAS); Pima Co., Baboquivari Mountains, Brown Canyon, 19.VII.1959, V. Roth, 3 imm. (AMNH); Pima Co., Baboquivari Mountains, 22.IV.1961, W.J. Gertsch, 1 imm. (AMNH); Pima Co., Wet Canyon, Mount Graham, 14.VII.1950, M.A. Cazier, 1 p♂ (AMNH); Pima Co., Colossal Cave Camp, 8.IX.1941, W. Ivie, 3 imm. (AMNH); Santa Cruz Co., Patagonia, turn off Hwy 80, 23.V.1972, D.C. Rentz, 1♂ (CAS); Santa Cruz Co., 5 miles southwest of Patagonia, 25.VIII.1950, M.A. Cazier, 1p♂, 2 imm. (AMNH); Santa Cruz Co., Madera Canyon, 5000', II-IV.1931, H. Exline, 1♀ (MCZ); Santa Cruz Co., Roundup Camp, Madera Canyon, 10.IX.1941, W. Ivie, 2p♂, 3 imm. (AMNH); Santa Cruz Co., Roundup Camp, Madera Canyon, 23.III.1960, W.J. Gertsch, W. Ivie, R. Schrammel, 5 imms, 1p♂ (AMNH); Santa Cruz Co., Santa Rita Mountains, Box Canyon, 19.VI.1957, 1♀ (AMNH); Santa Cruz Co., Santa Rita Mountains, 1 imm. (AMNH); Santa Cruz Co., Santa Rita Mountains, White House Canyon, 15.X.1936, O. Bryant, 1p♂, 1 imm. (AMNH); Santa Cruz Co., Santa Rita Mountains, mouth of Madera Canyon, 29.VII.1949, W.J., J.W. Gertsch, 4 imm. (AMNH); Santa Cruz County, Santa Rita Mountains, Madera Canyon, O. Bryant, 1p♂ (AMNH); Santa Cruz Co., Santa Rita Mountains, Madera Canyon, 4600', 2.V.1941, H. Ellsworth, 3 imm. (AMNH); Santa Cruz Co., Madera Canyon, 25.VIII.1950, M.A. Cazier, 1 imm. (AMNH); Santa Cruz Co., Santa Rita Mountains, Madera Canyon, VI.1898, GAS, 1♀ (USNM); Santa Cruz Co., 4 mi southeast of Ruby, 5.IX.1950, W.J. Gertsch, 1 imm, (AMNH); Yuma Co., Palm Canyon, 7.V.1960, W.J. Gertsch, V. Roth, 1 imm. (AMNH); Palm Canyon, Kofa Mountains, 25.IV.1960, W.J. Gertsch, 3 imm. (AMNH); Yavapai Co., Yarnell, 5.VI.1937, R. Komarek, 1♀ (AMNH); Yavapai Co., Congress Junction, VII.1938, J.P. Klein, 1♀, ♂ (CAS) **California:** Los Angeles Co., Mulholland Drive, Santa Monica Mountains, 1♀ (AMNH); Ventura Co., Santa Barbara, 1♀ (USNM); San Diego Co., Sutherland Dam, Santa Ysabel Creek, 14.V.1948, W.M. Pearce, 1 imm. (AMNH); San Diego Co., Jacumba, 30.IX.1961, W. Ivie, W.J. Gertsch, 3 imm. (AMNH). **Texas:** Presidio Co., 1 mile west of Lajitas, 23-30.IV.1963, J.E. Gillaspy, 1♀ (MCZ); Brewster Co., Big Bend National Park, KA Bar ranch house, 24-25.V.1967, E. Sabath, on rock porch, 1 imm. (AMNH); Brewster Co., Big Bend National Park, Hot Springs, 12.XII.1954, K.W. Haller, 1p♂ (AMNH); Edwards Co., Punkin Cave, 8 miles southwest of Carta Valley, 4.IX.1965, J. Redell, 1 imm. (AMNH); Val Verde Co., Marshall Bat Cave, bottom of dug entrance shaft, 800' from natural entrance, 1.I.1963, Reddell, Reddell, 1p♂ (AMNH); Val Verde Co., H. Fawcett Ranch, 40 miles north of del Rio, 29.XII.1958, F.E. Potter Jr., several (AMNH); Val Verde Co., 10 mi west of Del Rio at jnct of Devil's and US 90 P33, 7.IV.1963, C.E. Parker, 6 imm. (CAS).

Distribution.
*Selenops debilis*, *Selenops actophilus* and *Selenops nesophilus* are all found throughout the southwest of North America (Map 7).

### Selenops actophilus

Chamberlin, 1924

http://species-id.net/wiki/Selenops_actophilus

**Figures 53–60.**
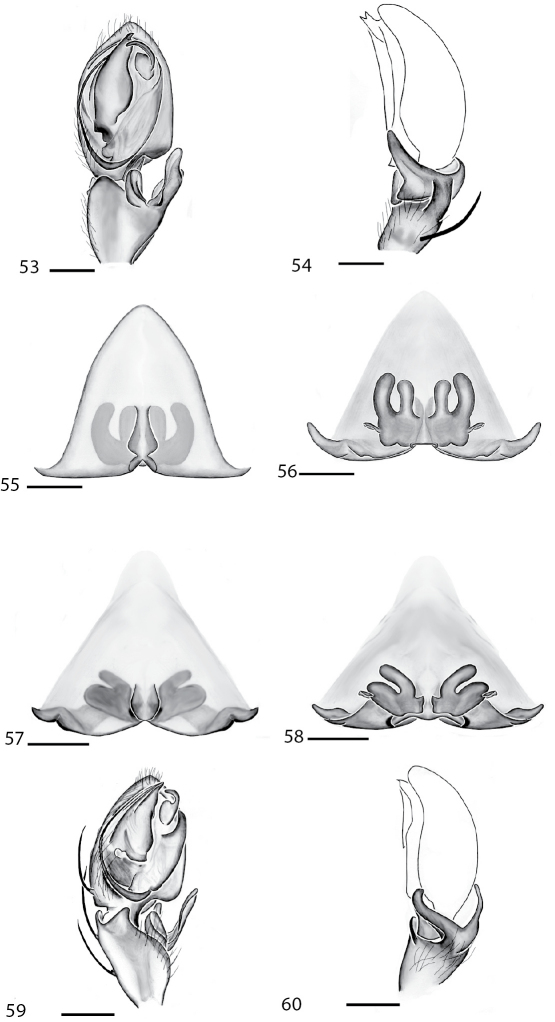
Copulatory organs of *Selenops actophilus* Chamberlin holotype male from San Carlos Bay, Sonora, México (CAS) **53–54** female CAS 1436 from San Pedro Bay, Sonora, México (CAS) **55–56** *Selenops debilis* Banks neotype female from San Jose del Cabo, México (MCZ) **57–58** male from Oro Blanco Mountains,12 miles from Nogales, Arizona (AMNH) **59–60, 53, 59** left pedipalp, ventral view **54, 60** left pedipalp, retrolateral view, **55, 57** epigyne, ventral view **56, 58** spermathecae, dorsal view. Scale bar = 0.50 mm.

Figs 53–56 198 Map 7

Selenops actophilus Chamberlin 1924: 655, Figs 95–98 (♂, ♀, examined).
Selenops actophilus :Muma 1953: 14, Figs 19–22 (♂, ♀).

#### Type material.

Holotype male: San Carlos Bay, Sonora, México, 7.VII.1921, J.C. Chamberlin (CAS1435, examined).

#### Other material examined.

MÉXICO: Baja California: Meling Ranch, 26.V.1981, V. Roth, 1♂ (AMNH). **Chihuahua:** 19 mi SW Tejaban along Urique River, 19.V.1991, R.E. Stecker 1♂ (CAS); 10 mi SW Tejaban along Urique River, 11-15.V.1991, R.E. Stecker, 2♀, 1♂, 3 imm. (CAS); 25 mi SW Tejaban along Urique River, 20-23.V.1991, R.E. Stecker, 1♂ (CAS). **Sinaloa:** 40 miles south of Culiacán, 6.VIII.1956, V. Roth, W.J. Gertsch, 1♀ (AMNH). **Sonora:** Guaymas, under a rock on hill above Jamison's Dock, 3.IV.1964, W. Shear, 1♀ (AMNH); Guaymas, 24.VI.1943, F.J. Pough, 1♀ (AMNH); Guaymas, 14.IV.1921, J. C. Chamberlin, 1♀ (AMNH); Guaymas, 9.IV.1921, E.P. Van Duzee, 1♀ (AMNH); San Pedro Bay, 7.VII. 1921, J.C. Chamberlin (CAS1436); 15 miles west of Yecora, 4000', 7.VIII.1986, V. Roth, 1♀ (CAS); Navajoa, 1.VIII.1952, P., C. Vaurie, 1♂ (AMNH); Alamos, on building, 27.IV.1986, V. Roth, several (AMNH); Sonoran Highway on way to Yecora, 28°34'08.7"N, 109°39'28.1"W, 1512', 14.VII.2007, M. VanDam, 1♀ (EME sel_851). **UNITED STATES: Arizona:** Cochise Co., Chiricahua Mtns.,1.VI.1952, M. Cazier, W.J. Gertsch, R. Schrammel, 1♀ (AMNH); Cochise Co., 4 mi west of Portal, South fork of Cave Creek, 10.VI.1958, W.J, J.W. Gertsch, 1♀ (AMNH); Cochise Co., Chiricahua Mtns, SWRS, 5400', 5.VI.1965, D. Ligon, 1♀ (AMNH); Cochise Co., Chiricahua Mountains, South Fork of Cave Creek, 31.51 N, 109.12 W, 24.V.1963, W.J. Gertsch, W. Ivie, 1♂, 1♀ (AMNH); Mohave Co., South of Wickieup, 2.7 miles from Hwy 93, Burro Creek Crossing Road, 34°36'449"N, 113°28'921"W, S. Crews, L. Bell, 1♂ (CAS sel_001); Pima Co., Organ Pipe Cactus National Monument, 10.VI.1952, M. Cazier, W. J. Gertsch, R. Schrammel, 1♂ (AMNH); Santa Cruz Co., Pena Blanca Lake, northwest of Nogales, 31.24 N, 111.05 W, 19.V.1963, W. J. Gertsch, W. Ivie, several (AMNH); Santa Cruz Co., 10 miles east of Nogales, 9.V.1961, W.J. Gertsch, 1♀, 1 imm. (AMNH); Santa Cruz Co., Santa Rita Mountains, Madera Canyon, 7.VI.1952, Cazier, Gertsch, Schrammel, 1♀ (AMNH - with a male, however, male cannot be confirmed as this species). **Texas:** Alpine Co, 30.VI.1965, running on ground, M.H. Muma, 1♂ (FSU); Brewster Co., Big Bend National Park, The Basin, 5400', 24.V.1965, K.W. Haller, 1♂ (AMNH); Presidio Co., 11 miles west of Valentine, VI.1948, Reagan, Flury, 2♀ (AMNH); Val Verde Co., 19 miles north of Comstock, 14.IV.1973, C. Soileau, 2♀, 1♂, 3 imm. (AMNH); Val Verde Co., Seminole Canyon, Hwy 90, under bridge, 29°42.523"N, 101°18.138"W, 18.XI.2004, along road, under rock, P. Paquin, PP-8804, 1 imm. (EME sel_208).

#### Diagnosis.

**Figures 61–68.**
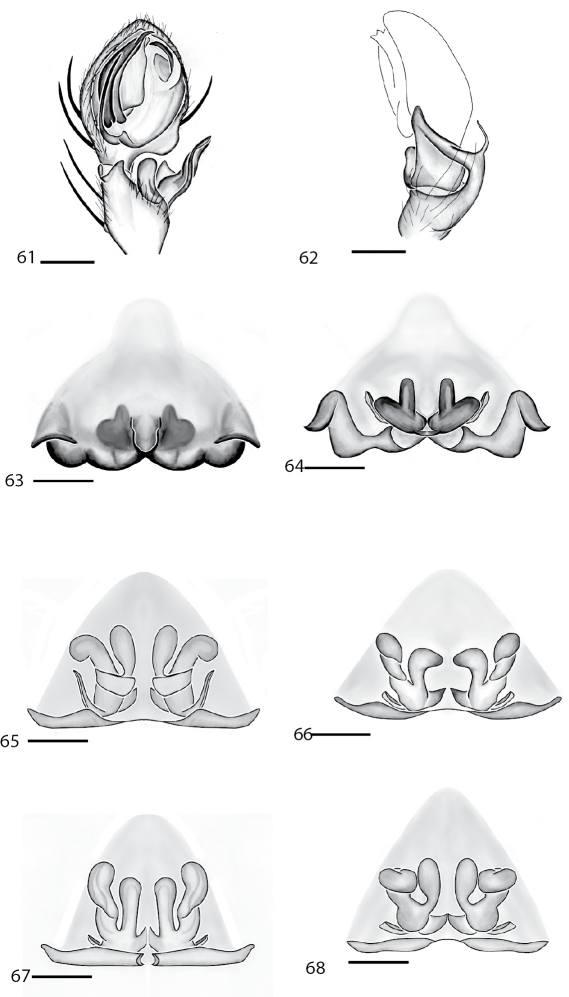
Copulatory organs of *Selenops nesophilus* Chamberlin holotype male from Isla Tortuga, Baja California Sur, México (CAS) **61–62** female from Isla Tortuga, Baja California Sur, México (CAS) **63–64** *Selenops debilis* group showing some of the internal variation found in various specimens. Female from Portal, Cochise Co., Arizona (AMNH) **65** females from Guadelupe Canyon, Cochise Co., Arizona (AMNH) **66–67** female from 1 mile west of Lajitas, Brewster Co., Texas (AMNH) **68, 61** left pedipalp, ventral view **62** left pedipalp, retrolateral view **63** epigyne, ventral view **64–67** spermathecae, dorsal view. Scale bar = 0.50 mm.

Males can be distinguished from *Selenops debilis* and *Selenops nesophilus* by a combination of the embolus beginning at the 5 o'clock position and a MA that is broad, but tapers suddenly to a finger-like extension. Additionally, the conductor and RTA are shaped differently than those of *Selenops debilis* and *Selenops nesophilus* (Figs 59–62). Females can be distinguished by a more triangular epigynal plate, with a distally narrower septum. Internally, there is a much smaller invagination of the posterodorsal fold than in the other two species, and the spermathecae consist of two nearly parallel branches extending anteriorly (Figs 55–56).

#### Description.

*Holotype male:* Color:carapace brownish-red; sternum red-brown; chelicerae reddish-brown; maxillae light yellow-brown, lightening to white distally; labium grey-brown, lightening distally; abdomen dorsally grey-brown, damaged; ventrally dusky grey with no markings; legs orange-brown, annulations no longer apparent on this specimen. Carapace:0.96 times longer than broad; Eyes:AER slightly recurved; PER recurved; PME larger than AME, PLE largest, ALE smallest; eye diameters, AME 0.15, ALE 0.05, PME 0.20, PLE 0.33; interdistances AME-PME 0.05, PME-ALE 0.18, ALE-PLE 0.43. PME-PME 0.95 mm. ALE-ALE 1.58; ocular quadrangle AME-AME 0.40, PLE-PLE 1.95; clypeus 0.08 high. Mouthparts: chelicerae with a few stout setae medially and anteriorly; maxillae longer than broad, with tuft of conspicuous setae distally; labium distally rounded. Sternum:1.11 times longer than broad, posteriorly indented. Legs:Leg formula 4321 (Muma, 1953); scopulae present on distal end of all 4 tarsi; tarsi I-IV with strong claw tufts; pr claw toothed, rl claw with fewer teeth; Leg I, missing; Leg II, Fm pr 1–1–1, d 1–1–1, rl 1–1–1; Ti pr 1–1–0, d 1–2–0, rl 1–1–0, v 2–2–2; Mt pr 1–1–0, v 2–2, rl 1–1–0; Leg III, Fm pr 1–1–1, d 1–1–1, rl 1–1–1; Ti pr 1–1–0, d 1–1–0, rl 1–1–0, v 2–1–2; Mt v 2–2; Leg IV, Fm pr 1–1–1, d 1–1–1, rl 1–1–1; Ti pr 1–1–0, d 1–2–0, rl 1–1–0, v 2–2–2; Mt pr 1–1–0, v 2–2, rl 1–1–0. Abdomen:without terminal setal tufts. Pedipalp:Fm, spination dorsal 0–1–4; cymbium ovoid in ventral view; conductor large, subangular structure arising mediolaterally, curved on one side and sinuous on the other, angular at bottom, tapering upwards with small hook distally, pointed toward 12 o'clock; embolus long, slender, curving around edge of bulb, beginning at 4 o'clock, terminating at 12 o'clock; MA located at 2 o'clock position, directed laterally, with short, very wide base and a long finger-like process; RTA with two branches, ventral process smaller of the two and blunt and curved, the lateral process a stout stalk with large nearly quadrangular process; tibial apophyses extend at least ¼ the length the cymbium in ventral view (Figs 53–54). *Dimensions:* Total length 8.60. Carapace length 4.25, width 4.45. Sternum length 2.00, width 1.80. Abdomen length 3.35, width 2.55. Pedipalp: Fm 1.50, Pt 0.30, Ti 0.75, Ta 1.00, total 3.55. Leg I: Missing. Leg II: Fm 5.50, Pt 2.00, Ti 5.00, Mt 4.75, Ta 2.00, total 18.75. Leg III: Fm 5.90, Pt 1.75, Ti 5.00, Mt and Ta missing. Leg IV: Missing.

*Female (CAS 1436):* Color:carapace (CAS 1436) brown-red (recent) dusky yellow with dark patches laterally; sternum (CAS 1436) red-brown (recent) light orange-brown darker at border; chelicerae (CAS 1436) reddish-brown (recent) dusky yellow with broad lateral brown stripes; maxillae (CAS 1436) red-brown lightening distally (recent) light brown lightening distally; labium (CAS 1436) red-brown, lightening distally (recent) orange-brown, dusky on sides, lightening distally; abdomen dorsally (CAS 1436) grey-brown, damaged (recent) grey to cream-colored, slightly damaged, markings not visible; ventrally (CAS 1436) dark grey (recent) cream-colored; legs (CAS 1436) orange-brown, markings not visible (recent) dusky cream with stripes, not present on retrolateral side of femur. Carapace:0.98 times longer than broad. Eyes:AER slightly recurved; PER recurved; PME larger than AME, PLE largest, ALE smallest; eye diameters, AME 0.23, ALE 0.10, PME 0.35, PLE 0.53; interdistances AME-PME 0.05, PME-ALE 0.23, ALE-PLE 0.53. PME-PME 1.23. ALE-ALE 2.08; ocular quadrangle AME-AME 0.45, PLE-PLE 2.30; clypeus 0.23 high. Mouthparts:Chelicerae with stout setae medially and anteriorly; labium distally rounded. Sternum:1.13 times longer than broad, posteriorly indented. Legs:Leg I only slightly shorter than legs II, III and IV; leg formula 4321 (Muma, 1953); scopulae present on tarsi of all legs and metatarsi of legs I and II; tarsi I-IV with strong claw tufts; pr claw per foot slightly toothed; spination: leg I, Fm pr 1–1–0, d 1–1–1, rl 1–1–1; Ti v 2–2–2; Mt v 2–2; leg II, Fm pr 1–1–0, d 1–1–1, rl 1–1–1; Ti v 2–2–2; Mt v 2–2; leg III, Fm pr 1–1–0, d 1–1–1, rl 1–1–1; Ti v 2–2–0; Mt v 2–2; leg IV, Fm pr 1–1–0, d 1–1–1, rl 1–1–1; Ti pr 1–0–0, v 2–2–0, rl 1–1–0; Mt v 2–2. Abdomen:without terminal setal tufts. Pedipalp:Claw with 11 teeth. Epigyne:epigynal plate triangular, narrow septum that widens and connects to what appear to be epigynal pockets, genital openings located where the septum begins to widen; internally, spermathecae simple, two branched cylindrical structures that extend anteriorly, with each branch nearly parallel to the other, though the outermost branches curve outward a bit; small posterodorsal fold present that doesn't cover any of the internal structures, and is just a very slight invagination of the bottom of the epigynal plate (Figs 55–56). *Dimensions:* Total length 8.68. Carapace: length 5.00 width 5.10. Sternum: length 2.25, width 2.00. Pedipalp: Fm 1.35, Pt 0.60, Ti 0.80, Ta 1.50, total 4.25. Leg I: Fm 5.00, Pt 1.80, Ti 4.00, Mt 3.50, Ta 1.75, total 16.05. Leg II: Missing. Leg III: Fm 5.80, Pt 2.00, Ti 4.65, Mt 4.00, Ta 1.75, total 18.20. Leg IV: Fm 5.75, Pt 2.00, Ti 4.75, Mt and Ta missing.

#### Natural History.

Collected under rocks in the arid southwest (Fig. 198).

#### Distribution.

Definitely found from Sonora, México, east to west Texas (Map 7). It may also be found in other areas of desert southwest of North America.

### Selenops debilis

Banks, 1898

http://species-id.net/wiki/Selenops_debilis

Figs 57–60 198 Map 7

Selenops debilis Banks 1898: 267, pl. 16, Fig. 14 (♀, not examined).
Selenops debilis Muma 1953: 13, figs 15–18 (♀, ♂; ♀ neotype examined).

#### Type material.

Neotype female designated by Muma (1953: 14): San Jose del Cabo, México, G. Eisen, F. Vaslit (MCZ, examined). The holotype, from the same location, was lost or destroyed.

#### Note.

Muma (1953: 14) also designated a male specimen from Oro Blanco Mountains, 12 miles from Nogales, Arizona, VII.1937, P. Steckler (AMNH), as an ‘allotype'. The neotype and Muma's ‘allotype' are in terrible shape and the descriptions differ from Muma (1953) in coloration and markings due to aging, drying and fading.

#### Other material examined.

MÉXICO: Baja California: El Rosario, under reeds along lagoon, hillsides, 5.V.1961, W.J. Gertsch, V. Roth, 1♀ (AMNH). **Baja California Sur:** 23 km north of La Paz, *Neotoma* 8460, 10.VII.1957, Ryckman, Spencer, 1♀ (AMNH); Sierra La Laguna, Cañon de la Zorra, 240 m, 22.VIII.1986, M. Jiminez, 1♂ (USNM). **Sinaloa:** 6 mi S of Culiacán, 22.VII.1954, W.J. Gertsch, 1♀ (AMNH). **Sonora:** San Miguel Hercostos, 2♀ (USNM). **UNITED STATES: Arizona:** Cochise Co., Chiricahua Mtns., 6000', 31.V.1952, M. Cazier, W.J. Gertsch, R. Schrammel, 1♀ (AMNH); Cochise Co., Portal, 12.VI.1923, W.J. Gertsch, 1♂ (AMNH); Pima Co.,Tucson, O. Bryant, 1♀ (AMNH); Pima Co., Santa Catalina Mountains, 17.V.1941, R.H. Crandall, 1p♀, 1♀ (AMNH); Santa Cruz Co., Paradise, 10.V.1989, S. Roth, 1♂ (CAS); Santa Cruz Co., Madera Canyon, Mt. Wrightson Trail, 1.VIII.2005, J. Starrett, S. Thomas, under rocks, logs in forest, 4 imm. (EME sel_264, 270, 271, 272). **New Mexico:** Hidalgo Co., Antelope Pass, 2.VI.1988, Tomberlin, pan trap, 1♂ (AMNH).

#### Diagnosis.

Females can be distinguished from others by the combination of a wider median septum with parallel lateral margins, and long, cylindrical spermathecae directed anterolaterally (Figs 57–58). Males can be distinguished from other species by the RTA, which is a very small stalk, distally bifurcated into a v-shape, with a small angular lateral projection (Figs 59–60).

#### Remarks.

The male was matched to the female neotype by Muma (1953) based on the general appearance, including coloration, pattern and structure. They are from distant localities, and there are other species in this group that are very similar, and species boundaries are unclear. Thus, it is entirely possible that this male and this female are not the same species.

#### Description.

*Male* (fromOro Blanco Mountains, Muma's ‘allotype'):carapace orange-brown; chelicerae orange-brown; maxillae brown-yellow, lighter distally; labium brown, lightening distally; abdomen dorsally orange-brown, no marks visible; legs orange-brown, markings indistinct. Carapace: 0.94 times longer than broad. Eyes:AER nearly straight; PER recurved; PME larger than AME, PLE largest, ALE smallest; eye diameters, AME 0.13, ALE 0.10, PME 0.30, PLE 0.35; interdistances AME-PME 0.03, PME-ALE 0.10, ALE-PLE 0.50. PME-PME 1.00. ALE-ALE 1.60; ocular quadrangle AME-AME 0.35, PLE-PLE 1.88; clypeus 0.09 high. Mouthparts:chelicerae with a few stout setae medially and anteriorly; maxillae longer than broad, with tuft of conspicuous setae distally; labium distally rounded. Sternum:as long as broad, posteriorly indented. Legs:leg formula 4321 (Muma, 1953); scopulae present on distal end of all 4 tarsi; tarsi I-IV with strong claw tufts; both claws with same number of teeth; spination: leg I, Fm pr 1–1–1, d 1–1–1, rl 1–1–1; Ti pr 1–0–1, rl 1–0–1, d 1–2–0, v 2–2–2; Mt pr 1–0–0, v 2–2–0, rl 1–1–0; II, F pr 1–1–1, d 1–1–1, rl 1–1–1; Ti pr 1–1–0, d 1–2–0, rl 1–1–0, v 2–2–2; Mt pr 1–0–0, v 2–2, rl 1–0–0; III, Fm pr 1–1–1, d 1–1–1, rl 1–1–1; Ti pr 1–0–1, rl 1–0–1, d 1–1–1, v 2–2–2; Mt pr 1–0–0, rl 1–0–0, v 2–2. Abdomen:without terminal setal tufts. Pedipalp:Fm, spination d 0–1–4; cymbium oval in ventral view, slightly angled posterolaterally; conductor large angular structure arising laterally, curved on one side, sinuate on other, tapering upwards, pointed distally; embolus long, slender, curved, beginning at 5 o'clock, terminating at 12 o'clock; MA with short round base, tapering abruptly to longer finger-like process, originating at 2 o'clock, directed laterally; RTA with two processes, dorsal process bifurcated, on short stalk, distally v-shaped, with angular process; ventral apophysis small, rounded,directed ventrally; apophyses extend at least ¼ the length of the cymbium in ventral view (Figs 59–60). *Dimensions:* Total length 8.70. Carapace length 4.15, width 4.40. Sternum length 1.70, width 1.70. Abdomen length 4.55, width 3.00. Pedipalp: Fm 1.50, Pt 0.35, Ti 0.75, Ta 1.40, total 4.00. Leg I: Fm 4.75, Pt 2.00, Ti 4.90, Mt 4.50, Ta 2.40, total 18.55. Leg II: Fm 5.30, Pt 2.40, Ti 5.00, Mt 5.00, Ta 2.00, total 19.70. Leg III: Fm 5.00, Pt 1.85, Ti 4.80, Mt 5.00, Ta 2.00, total 18.65. Leg IV: Absent, Fm 4.99, Pt 1.53, Ti 4.58, Mt 4.16, Ta 1.87, total 17.13.

*Neotype female:* Color:carapace orange-brown, no markings visible; chelicerae orange-brown; maxillae orange-brown, lightening distally; labium orange-brown, lightening distally; abdomen dorsally yellow-tan with mediolateral dark spots and laterocaudal festoon; ventrally dusky yellow-grey, no markings; legs pale-yellow. Carapace: 0.94 times longer than broad. Eyes:AER slightly recurved; PER recurved; PME larger than AME, PLE largest, ALE smallest; eye diameters, AME 0.15, ALE 0.10, PME 0.30, PLE 0.45; interdistances AME-PME 0.05, PME-ALE 0.08, ALE-PLE 0.45. PME-PME 1.00. ALE-ALE 1.60; ocular quadrangle AME-AME 0.35, PLE-PLE 1.80; clypeus 0.15 high. Mouthparts:chelicerae with a few stout setae medially and anteriorly; maxillae longer than broad, with tuft of conspicuous setae distally; labium distally rounded. Sternum:1.14 times longer than broad, posteriorly indented. Legs:leg formula 4321 (Muma, 1953); spination: unavailable, legs disarticulated. Abdomen:without terminal setal tufts. Pedipalp:claw with 13 teeth. Epigyne: Median septum conspicuous, sides of septum mostly parallel, somewhat truncate posteriorly, genital openings located at lateral margins of septum, posterior margin of plate sinuous; internally, ducts elongated, directed anterolaterally, fertilization ducts located laterally; small posterodorsal fold present, sinuous, not covering internal ducts (Figs 55–56). *Dimensions:* Total length 8.85. Carapace length 3.85, width 4.10. Sternum length 2.00, width 1.75. Abdomen length 5.00, width 4.00.

#### Natural History.

Found under rocks and logs in both arid regions (Fig. 198) and oak woodlands.

#### Distribution.

Known from the southwest of North America, from southwestern New Mexico to Baja California, México (Map 7).

### Selenops nesophilus

Chamberlin, 1924

http://species-id.net/wiki/Selenops_nesophilus

Figs 61–64 198–199 Map 7

Selenops nesophilus Chamberlin 1924: 656, Figs 97–98 (♂, ♀, examined).
Selenops nesophilus Muma 1953: 15, Figs 23–26 (♂, ♀).

#### Type material.

Holotype male: Isla Tortuga, Baja California Sur, México, 11.V.1921, J.C. Chamberlin (CAS, examined). Paratypes: Female, same data as holotype (CAS, examined).

#### Other material examined.

MÉXICO: Baja California: 2 miles south El Rosario on Mex Hwy 1 (dirt road) RB-3, 26.XI.1968, P.R. Craig, D. Dailey, 1♂ (CAS); Isla Angel de la Guarda, Palm Cañon, under bark of Palo Verde, 3.V.1921, J.C. Chamberlin, 1♀ (CAS); Isla Raza, 21.IV.1921, J.C. Chamberlin, 1♀, 2 imm. (CAS); 11 mi SW Punta Prieta, 200' 15.IV.1969, S.C. Williams, 1♀ (CAS); Isla Cedros, 2nd Canyon, South of Punta Norte on east side of island, 2.VII.1983, V.F. Lee, 1♂ (CAS). **Baja California Sur:** between San Juanico and San Ignacio, ~2km East of Ballena, 26°24'00.4"N, 112°35'64.0"W, ~190', 20.III.2004, M.C. Hedin, under rocks, 1♀, 1♂ (EME sel_210, 213); Cuevas Pintas, 25.58.644 N, 111.27.924 W, 31.III.2003, D. Palmer, D. Wood 1 imm. (EME sel_009); Isla Magdalena, Laguna Santa Maria, 9.VII.1983, D.K. Faulkner, D. C. Lightfoot, 1♀ (CAS); West of San Miguel Comondu, road to Villa Insurgentes, ~3 km West of town, 26.01'47.3"N, 111.50'84.8"W, ~590', 18-19.III.2004, M.C. Hedin, under rocks along road, 1p♂ (EME sel_212); Puerto Escondido, 14.VI.1921, E.P. Van Duzee, 1♂ (CAS); 6.8 mi S of San Antonio, 24.VII.1980, Williams, Bentzion, 1♀ (CAS). **Sinaloa:** 6 miles south of Culiacan, 22.VII.1954, W.J. Gertsch, 1♀ (AMNH). **Sonora:** Los Algodones, 6 miles west San Carlos Bay, 22-23.III.1980, C.E. Griswold, 1♂ (CAS). **UNITED STATES: Arizona:** Cochise Co., 2.5 miles southeast of Portal, 26.III.1965, B., C. Durden, several (AMNH); Coconino Co., Grand Canyon National Park, Tapeats Creek Campground, Tapeats Canyon, Tapeats Creek Trail 36°22.826'N, 112°27.744'W, ~2230', 15.VIII. 2004, S. Crews, under rocks, 1 imm. (EME sel_211); Pima Co., Espero Canyon, 1♀ (AMNH); Pima Co., Baboquivari Mountains, Brown Canyon, 9.VI.1952, M. Cazier, W.J. Gertsch, R. Schrammel, several (AMNH); Pima Co., Esperero Co., several (AMNH); Pinal Co., Superior Thompson Southwestern Arboretum, 11.V.1940, 2300', D.C. Lowrie, 1♂ (AMNH). **California:** San Diego Co., Upper Otay River Valley, 19.V.2003, D. Palmer, 1♀ (EME sel_002); San Diego Co., Jamul, Lyon's Valley, N of Lyon's Pk, 32.71415°N, 116.7677°W, ~674 m, D. Palmer, running on ground, oak woodland, 1♂ (EME sel_837); San Diego Co., Anza Borrego Desert State Park, Carrizo Palm Grove, below railroad tracks, 32.46°N, 116.11°W, ~1800', 23.II.2003, M.C. Hedin, D. Palmer, D. Wood, 2 imm. (EME sel_021, 214).

#### Diagnosis.

Males can be differentiated from all other species by the embolus, which originates at 6 o'clock, a long process on the MA, and the RTA, which is on a very long stalk and has a very large quadrangular process distally (Figs 61-62). Females can be separated from other species by the posterior margin of the epigynum which is sinuous and heavily sclerotized, with a median septum that is wider anteriorly, and a posterodorsal fold that is sinuous (Figs 63–64).

#### Description.

*Holotype male:* Color:carapace orange-brown (recent) pale yellow; sternum (holotype) red-brown (recent) yellow; chelicerae (holotype) brown (recent) yellowish, like carapace, darker toward inner margins; maxillae (holotype) yellow-brown, lightening distally (recent) light yellow, lightening distally; labium (holotype) orange-brown (recent) light yellow-orange; abdomen dorsally (holotype) dark grey-brown (recent) pale yellow to cream with some darker grey flecks, festoon present; ventrally (holotype) grey (recent) light yellow; legs (holotype) orange-brown, no markings evident (recent) pale yellow with darker grey annulations. Cephalothorax:0.91 times longer than broad. Eyes:AER slightly recurved; PER recurved; PME larger than AME, PLE largest, ALE smallest; eye diameters, AME 0.18, ALE 0.10, PME 0.35, PLE 0.55; interdistances AME-PME 0.03, PME-ALE 0.18, ALE-PLE 0.75. PME-PME 1.18. ALE-ALE 2.00; ocular quadrangle AME-AME 0.45, PLE-PLE 2.38; clypeus 0.23 high. Mouthparts:chelicerae with a few stout setae medially and anteriorly; maxillae longer than broad, with tuft of conspicuous setae distally; labium distally rounded. Sternum:1.25 times longer than broad, posteriorly indented. Legs:leg I only slightly shorter than legs II, III and IV; leg formula 2341; scopulae present on all 4 tarsi, as well as tibia and metatarsus of leg I and distally on metatarsi of legs II, III, IV; tarsi I-IV with strong claw tufts; pr claw toothed, rl claw with fewer teeth; spination: leg I, Fm pr 1–1–1, d 1–1–1, rl 1–1–1; Ti pr 1–0–1, d 1–2–0, rl 1–0–1, v 2–2–2; Mt pr 1–1–0, rl 1–1–0, v 2–2; leg II, Fm pr 1–1–1, d 1–1–1, rl 1–1–1; Ti pr 1–0–1, rl 1–0–1, d 1–2–0, v 2–2–2–1; Mt pr 1–1–0, v 2–2, rl 1–1–0; leg III, Fm pr 1–1–1, d 1–1–1, rl 1–1–1; Ti pr 1–0–1, d 1–0–0, rl 1–0–0, v 2–2–1–1; Mt pr 1–1–0, rl 1–1–0, v 2–2; leg IV, Fm pr 1–1–1, d 1–1–1, rl 1–1–1; Ti pr 1–0–1, rl 1–1–1, d 1–1–0, v 2–2–2; Mt pr 1–1–0, rl 1–1–0, v 2–2. Abdomen:without terminal setal tufts. Pedipalp:Fm, spination dorsal 0–1–4; cymbium oval in ventral view, slightly angled posterolaterally; conductor large, angular, arising laterally, curved on one side, sinuate on other, tapering distally; embolus long, slender, curved, beginning at 6 o'clock, terminating at 12 o'clock; MA arising at 2 o'clock, directed laterally, with short stout base and long finger-like process; two tibial apophyses, ventral process smaller of the two, blunt and curved, lateral process a stout stalk curving laterally away from cymbium, with large angular blunt process, similar to type of *Selenops actophilus*, but stalk longer and more curved (Figs 61–62). *Dimensions:* Total length 8.75. Carapace length 5.13, width 5.65. Sternum length 2.50, width 2.00. Abdomen length 3.53, width 2.78. Pedipalp: Fm 1.75, Pt 0.65, Ti 0.75, Ta 1.75, total 4.90. Leg I: Fm 6.75, Pt 2.25, Ti 5.50, Mt 5.25, Ta 2.75, total 22.50. Leg II: Fm 6.00, Pt 2.75, Ti 6.25, Mt 5.60, Ta 2.60, total 23.20. Leg III: Fm 6.75, Pt 2.50, Ti 5.80, Mt 5.75, Ta 2.20, total 23.00. Leg IV: Fm 6.00, Pt 2.00, Ti 6.00, Mt 6.00, Ta 2.75, total 22.75.

*Paratype female:* Color:carapace (paratype) red-brown (recent) yellow-tan, brown line extending from center of cephalic area medially thorough entire cephalothorax, dark spots laterally; sternum (paratype) brown, darker around border (recent) yellow, darker around border; chelicerae (paratype) red-brown (recent) light brown medially, darker laterally; maxillae (paratype) brown (recent) yellow-orange, both lightening distally; labium (paratype) brown (recent) orange-brown, lightening distally; abdomen dorsally (paratype) grey-brown (recent) grey-brown, lighter medially, dark festoon present; ventrally cream colored; legs (type) orange-brown, annulations barely visible (recent) pale yellow-brown with dark annulations. Carapace:0.92 times longer than broad. Eyes:AER slightly recurved; PER recurved; PME larger than AME, PLE largest, ALE smallest; eye diameters, AME 0.15, ALE 0.08, PME 0.45, PLE 0.65; interdistances AME-PME 0.03, PME-ALE 0.15, ALE-PLE 0.70. PME-PME 1.35. ALE-ALE 2.35; ocular quadrangle AME-AME 0.53, PLE-PLE 2.68; clypeus 0.15 high. Mouthparts:chelicerae with a few stout setae medially and anteriorly; maxillae longer than broad, with tuft of conspicuous setae distally. Sternum:1.20 times longer than broad, posteriorly indented. Legs:leg formula 4321; scopulae present on all 4 tarsi and metatarsi and distally on tibiae on legs I and II; tarsi I-IV with strong claw tufts; pr claw per foot slightly toothed; spination: leg I, Fm pr 1–1–0, d 1–1–1, rl 1–1–1; Ti v 2–2–2; Mt v 2–2; II, Fm pr 1–0–0, d 1–1–1, rl 1–1–1; Ti v 2–2–2; Mt v 2–2; III, Fm pr 1–1–0, d 1–1–1, rl 1–1–1; Ti v 2–2–0; Mt v 2–2. Abdomen:without terminal setal tufts. Pedipalp:claw with 9 teeth. Epigyne:median septum present, wider anteriorly, rounded to pointed terminally, posterior margin of plate sinuous, with lateral protrusions, genital openings located at lateral margins of septum; internally, cylindrical ducts directed anteriorly and anterolaterally, fertilization ducts located laterally, directed anterolaterally, posterodorsal fold present, not connected medially, heavily sclerotized, not covering internal ducts (Figs 63–64). *Dimensions:* Total length 11.50. Carapace length 5.68, width 6.20. Sternum length 3.00, width 2.50. Abdomen length 5.83, width 3.73. Pedipalp: Fm 1.75, Pt 0.75, Ti 1.00, Ta 2.00, total 5.50. Leg I: Fm 6.00, Pt 2.50, Ti 5.00, Mt 4.00, Ta 1.75, total 13.25. Leg II: Missing. Leg III: Fm 6.50, Pt 2.50, Ti 5.50, Mt 5.00, Ta 2.00, total 21.50. Leg IV: Missing.

#### Natural History.

This species has been found under rocks (Figs 198–199) as well as running on the ground in oak woodlands.

#### Distribution.

Known from southwestern North America, from Baja California Sur and Sinaloa to Arizona (Map 7).

### Selenops lepidus

Muma, 1953

http://species-id.net/wiki/Selenops_lepidus

Figs 69–72 Map 5

Selenops lepidus Muma 1953: 17. Figs 27–29 (♂, ♀, examined).

#### Type material.

Holotype male: Manzanillo, Colima, México, 17.VI.1941 L.I. Davis (AMNH, examined). Paratypes: Female from San Blas, Nayarit, México, 6.VIII.1947, C. M. Goodnight, B. Malkin (AMNH, examined).

#### Other material examined.

MÉXICO: Nayarit: San Juan Peyotan, 1-3.VIII.1955, B. Malkin, 1♀ (AMNH).

#### Diagnosis.

**Figures 69–76.**
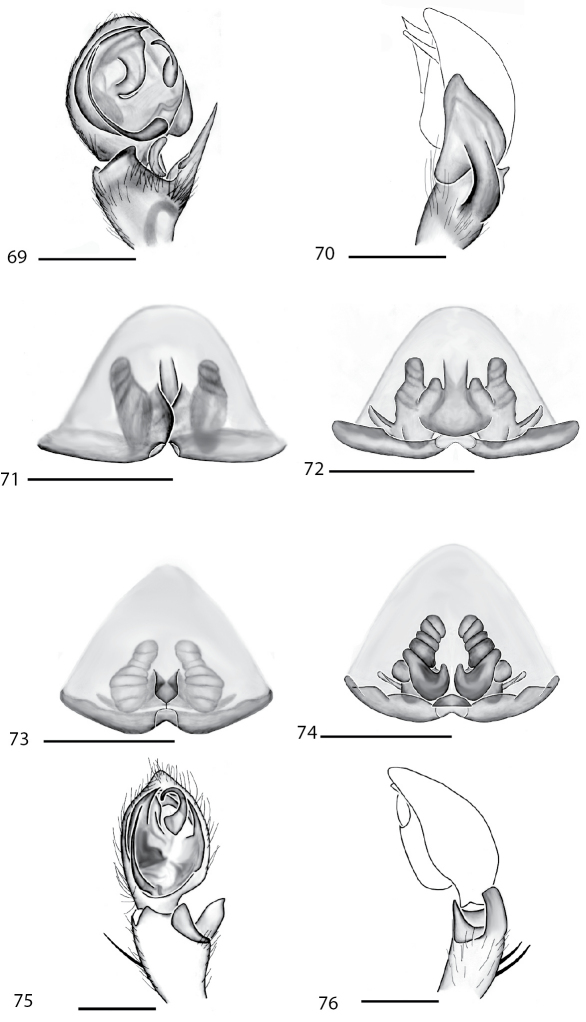
Copulatory organs of *Selenops lepidus* Muma holotype male from Manzanillo, Colima, México (AMNH) **69–70** female from San Blas, Nayarit, México (AMNH) **71–72** *Selenops chamela* sp. n. holotype female from Chamela, Jalisco, México (AMNH) **73–74** *Selenops petenajtoy* sp. n. holotype male from Cueva Aktun Kan, Colonia del Bosque, Santa Elena de la Cruz, Petén, Guatemala (EME sel_867) **75–76, 69, 75** left pedipalp, ventral view **70, 76** left pedipalp, retrolateral view **71, 73** epigyne, ventral view **72, 74** spermathecae, dorsal view. Scale bar = 0.50 mm.

Males can be differentiated from all other species by having a very narrow base of the MA, and the lateral branch of the RTA is a large stalk with an immense triangular distal process (Figs 69–70). Females can be separated from all other species by having the median septum covered distally by the lateral lobes with one lobe partially covering the other (Figs 71–72).

#### Remarks.

This male and female were not collected together and it is presumed Muma (1953) placed them together based on their size. However, a second small species (*Selenops chamela* sp. n., see below) known from a single female has been found in this region, and nearer to the type locality of the male of *Selenops lepidus*. Thus, it is possible that what is currently described as the female *Selenops lepidus* is actually a different species. In this region of México, there are 3 females known and 2 males known, and it is unclear if any of them go with the other, or if they are all separate species. At this time I make no changes.

#### Description.

*Holotype male:* Color:carapace orange-brown with white setae, markings indistinguishable; chelicerae dark red-brown; labium pale brown; abdomen dorsally grey-tan, darker mediolaterally, laterocaudal festoon present; abdomen ventrally grey-tan; legs orange-brown, some annulations still visible. Carapace: as long as broad. Eyes:AER slightly recurved; PER recurved; PME larger than AME, PME same as PLE, ALE smallest; eye diameters, AME 0.10, ALE 0.05, PME 0.18, PLE 0.18; interdistances AME-PME 0.05, PME-ALE 0.08, ALE-PLE 0.13. PME-PME 0.70. ALE-ALE 1.28; ocular quadrangle AME-AME 0.20, PLE-PLE 1.25. Mouthparts:chelicerae with a few stout setae medially and anteriorly; maxillae longer than broad, with tuft of conspicuous setae distally; labium distally rounded. Sternum:posteriorly indented. Legs:disarticulated; leg formula 4321 (Muma 1953); tarsi I-IV with strong claw tufts.Abdomen:without terminal setal tufts. Pedipalp:Ti: 0.60, Ta 0.80;cymbium oval in ventral view, slightly angled posterolaterally; conductor large, angular structure arising from the center of bulb on short, curved stalk connected to the side, tapering anterolaterally, pointed distally; embolus long, slender, curved, beginning between 4 and 5 o'clock, ending at 12 o'clock; MA arising at 3 o'clock, directed anterolaterally, with a long ovoid base and a long, narrow curving, finger-like process arising from base; two tibial apophyses, ventral structure curves toward cymbium, below angled portion; lateral apophysis a large angular structure on long stalk which curves away from, then towards, cymbium; RTA extends at least 1/3 length of the cymbium (Figs 69–70). *Dimensions:* Total length 5.05. Carapace length 2.63, width 2.63. Abdomen length 2.43, width 1.93.

*Paratype female:*Color:carapace orange-brown with white setae, markings indistinguishable; sternum light brown; chelicerae brown; maxillae light orange-brown lightening distally; labium brown lightening distally; abdomen dorsally grey-tan, broad cream-colored w-shaped mark posteriorly, laterocaudal festoon present; ventrally dusky grey with no markings; legs orange-brown with annulations. Carapace: 1.01 times longer than broad. Eyes:AER slightly recurved; PER recurved; PME larger than AME, PLE largest, ALE smallest; eye diameters, AME 0.12, ALE 0.10, PME 0.20, PLE 0.28; interdistances AME-PME 0.03, PME-ALE 0.10, ALE-PLE 0.30. PME-PME 0.85. ALE-ALE 1.40; ocular quadrangle AME-AME 0.40, PLE-PLE 1.57; clypeus 0.13 high. Mouthparts:chelicerae with a few stout setae medially and anteriorly; maxillae longer than broad, with tuft of conspicuous setae distally; labium distally rounded. Sternum:1.25 times longer than broad, posteriorly indented. Legs:leg formula 4321 (Muma, 1953); tarsi I-IV with strong claw tufts; prolateral claw per foot slightly toothed. Abdomen:without terminal setal tufts. Epigyne:median septum located in center of epigynal plate, narrow and parallel sided, lateral lobes covering terminus of septum, one lobe atop the other, making the epigynum appear slightly asymmetrical, genital openings located posterolaterally to septum, epigynal pockets present; internally, openings connect to a large, somewhat triangular atrium that connects to lateral anteriorly directed ducts, one small, the other larger with spiralled appearance, fertilization ducts located and directed posterolaterally, posterodorsal fold present, but barely covers part of internal ducts (Figs 71–72). *Dimensions:* Total length 7.23. Carapace length 2.98, width 2.95. Sternum length 1.25, width 1.00. Abdomen length 4.25, width 2.90. Leg measurements unavailable.

#### Natural History.

No data.

#### Distribution.

Southwestern México, from Nayarit to Colima (Map 5).

### Selenops chamela sp. n.

urn:lsid:zoobank.org:act:E7E54D3B-0940-45AD-84DC-ACA7A7A039B6

http://species-id.net/wiki/Selenops_chamela

Figs 73–74 Map 5

#### Type material.

Holotype female: Chamela, Jalisco, México, 1.X.1989, sea level, W. Eberhard (AMNH).

#### Etymology.

This species is named after the type locality, Chamela, and is to be treated as a noun in apposition.

#### Diagnosis.

Females can be separated from all other species by the epigynum which has a median septum and epigynal pockets, and internally, the ducts are coiled at least 5 times (Figs 73–74). Males unknown.

#### Remarks.

It is unclear whether this is a new species or is the female of *Selenops lepidus*. See abover under ‘Remarks' of the latter species.

#### Description.

*Holotype female*: color: carapace yellow-brown with dusky marks laterally, with white setae; sternum brown; chelicerae orange-brown; maxillae brown; labium yellow-brown; abdomen dorsally cream to grey brown, with a darker lanceolate stripe and several w-shaped darker markings, festoon absent; ventrally dusky with no marks; legs yellow-brown with darker annulations. Carapace: 0.92 times as long as broad; fovea longitudinal, broad, shallow. Eyes: AER slightly recurved; PER recurved; PME larger than AME, PLE largest, ALE smallest; eye diameters, AME 0.09, ALE 0.02, PME 0.20, PLE 0.25; interdistances AME-PME 0.09, PME-ALE 0.10, ALE-PLE 0.30, PME-PME 0.50, ALE-ALE 1.20; ocular quadrangle AME-AME 0.10, PLE-PLE 1.20; clypeus 0.08 high; chillum absent. Mouthparts: chelicerae with a few stout setae medially and anteriorly; lateral boss present smooth; maxillae longer than broad, with tuft of conspicuous setae distally; labium distally rounded. Sternum: longer than broad, posteriorly indented. Legs: leg I shorter than 2; leg formula unknown, some parts missing; scopulae present on all 4 tarsi; tarsi I-IV with strong claw tufts; spination: leg I, Fm pr 1-1-0, d 1-1-1; Ti d 0, v 2-2-2; Mt v 2-2; leg II, Fm pr 1-0-0, d 1-1-1, rl 1-0-1; Ti v 2-2-2; Mt v 2-2; leg III, Fm pr 1-0-0, d 1-1-1, rl 1-0-0, Ti 0, Mt missing; leg IV, Fm pr 0, d 1-1-1, rl 0-0-1; Ti v 1-1; Mt v 2-0. Abdomen: with terminal setal tufts. Epigyne: median septum located medially on plate, narrowing slightly posteriorly, slightly pointed, epigynal pockets present, genital openings located at anterolateral margins of median septum; internally, large lateral ducts connect to anteriorly directed laterally coiling ducts, coiled at least 5 times, fertilization ducts located posteriorly, directed posterolaterally, posterodorsal fold present, sinuous, barely covering any portion of internal ducts (Figs 73-74). *Dimensions:* Total length 5.50. Carapace length 2.30, width 2.50. Abdomen length 3.20, width 2.60. Pedipalp: Fm 0.50, Pt 0.30, Ta, 0.40, total 1.20. Leg I: Fm 2.00, Pt 0.70, Ti 1.40, Mt 1.00, Ta 0.90, total 5.00. Leg II: Fm 1.70, Pt 0.70, Ti 1.30, Mt 1.10, Ta 1.00, total 5.80. Leg III: Fm 1.40, Pt 0.65, Ti 2.30, Mt missing, Ta missing. Leg IV: Fm 1.60, Pt 0.90, Ti 2.00, Mt missing, Ta absent.

#### Natural history.

No data.

#### Distribution.

Known only from the type locality (Map 5).

### Selenops petenajtoy sp. n.

urn:lsid:zoobank.org:act:D51546F7-C481-49C6-8508-C5B6A3347803

http://species-id.net/wiki/Selenops_petenajtoy

Figs 75–76 Map 5

#### Type material.

Holotype male: outside of Cueva Aktun Kan, Colonia del Bosque, Santa Elena de la Cruz, Petén, Guatemala, 16°54'10.9"N, 89°53'44.3"W, ~164 m, 1.I.2008, S. Crews, under bark of large tree, SCC08_001 (EME sel_867).

#### Other material examined.

GUATEMALA:
**Petén**, Santa Elena de la Cruz, Colonia del Bosque, other entrance of Cueva Aktun Kan, 16°54'15.7" N, 89°53'54.6" W, ~142 m, 31.XII.2007, S. Crews, under bark, SCC07_060, 2 imm. (CAS sel_865, 866).

#### Etymology.

The name of this species comes from a combination of words, Petén, referring to the type locality, and the word for spider, ajtoy, in Mopan Mayan, the language indigenous to the people of the region of the type locality. It is to be treated as a noun in apposition.

#### Diagnosis.

**Figures 77–84.**
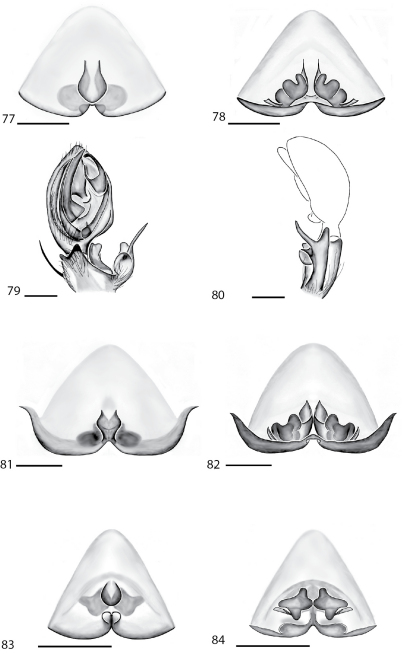
Copulatory organs of *Selenops huetocatl* sp. n. holotype female from Las Golondrinas, Huixtla, Chiapas, México (CNAN sel_045) **77–78** *Selenops bifurcatus* Banks male from Oricuajo, Costa Rica (MCZ) **79–80** female (holotype of *Selenops salvadoranus*) from San Salvador, El Salvador (MCZ) **81–82** *Selenops buscki* Muma of holotype female from Taboga Island, Panamá (USNM) **83–84,** **77, 81, 83** epigyne, ventral view **78, 82, 84** spermathecae, dorsal view **79** left pedipalp, ventral view **80** left pedipalp, retrolateral view. Scale bar = 0.50 mm.

Males can be separated from all other species by the large, hooked MA, and the small simple RTA (Figs 77–78). Females unknown.

#### Description.

*Holotype male:* color:carapace light yellow, a few dusky marks laterally, dark seam around edge of cephalothorax, clypeus with dark stripes beneath ALE; sternum yellow, darker around border; chelicerae dusky yellow, black stripe anteriorly; maxillae dusky yellow brown, lightening distally; labium dusky yellow, lightening distally; abdomen dorsally light yellowish, lighter distally, darkening caudally, with 2 darker horizontal stripes caudally and laterocaudal festoon; ventrally dusky yellow grey, no markings; legs light yellow with dusky annulations, specks on anterolateral faces of femora, forming long line. Carapace: 0.95 times longer than broad; fovea longitudinal, broad, very shallow. Eyes:AER slightly recurved; PER recurved; PME larger than AME, PLE largest, ALE smallest; eye diameters, AME 0.20, ALE 0.10, PME 0.30, PLE 0.40; interdistances AME-PME 0.03, PME-ALE 0.18, ALE-PLE 0.40. PME-PME 1.00. ALE-ALE 1.72; ocular quadrangle AME-AME 0.40, PLE-PLE 1.80; clypeus 0.15 high. Mouthparts:chelicerae with a few scattered setae medially and anteriorly; maxillae longer than broad, with tuft of conspicuous setae distally; labium distally rounded. Sternum:1.12 times longer than broad, posteriorly indented. Legs:leg I much shorter than legs II, III and IV; leg formula 2341; scopulae present on distal end of all 4 tarsi; tarsi I-IV with strong claw tufts; pr claw toothed, rl claw with fewer teeth; spination: leg I, Fm pr 1–1–1, d 1–1–1, rl 1–1–1; Ti d 0–1–0, pr 1–0–1, rl 1–0–1, v 2–2–2; Mt f 2–2; leg II, Fm pr 1–1–1, d 1–1–1, rl 1–1–1; Ti pr 1–0–1, d 0–1–0, rl 1–0–1, v 2–2–2; Mt v 2–2; leg III, Fm pr 1–1–1, d 1–1–1, rl 1–1–1; Ti pr 1–0–1, rl 1–0–1, v 2–2–1; Mt v 2–2; leg IV, Fm pr 1–1–1, d 1–1–1, rl 1–1–1; Ti pr 1–0–1, v 2–2–0, rl 1–0–1; Mt pr 1–1–0, rl 1–1–0, v 2–2. Abdomen:without terminal setal tufts. Pedipalp:Fm, spination d 0–1–4; cymbium oval in ventral view, angled posterolaterally; scopulae scattered, denser distally; conductor large angular structure arising from side of bulb, curved on one side, sinuate on other, tapering anteriorly; embolus long, slender, straight, arising at 7 o'clock, terminating at 11 o'clock; MA large with short triangular base with long hooked finger-like process, MA arising at 2 o'clock, directed anterolaterally; RTA barely reaching cymbium in ventral view, with two processes, lateral process long and rectangular, slightly curved, distally truncate, ventral process widening and rounded distally, slightly twisted (Figs 75–76). *Dimensions:* Total length 6.80. Carapace length 3.55, width 3.73. Sternum length 1.90, width 1.70. Abdomen length 3.25, width 2.35. Pedipalp: Fm 1.00, Pt 0.30, Ti 0.60, Ta 1.00, total 2.90. Leg I: Fm 3.75, Pt 1.00, Ti 3.80 Mt 4.00, Ta 1.75, total 14.30. Leg II: Fm 4.75, Pt 2.50, Ti 4.20, Mt 4.00, Ta 1.75, total 17.20. Leg III: Fm 4.80, Pt 1.75, Ti 4.00, Mt 4.00, Ta 1.60, total 16.15. Leg IV: Fm 4.75, Pt 1.50, Ti 4.00, Mt 4.00, Ta 1.75, total 16.00.

#### Natural history.

This species has been found beneath bark on a large tree with at least one other species of selenopid present (*Selenops mexicanus*).

#### Distribution.

Known only from the type locality and immediate vicinity (Map 5).

### Selenops huetocatl sp. n.

urn:lsid:zoobank.org:act:48506E93-9F05-4062-B609-541D9A87E6A9

http://species-id.net/wiki/Selenops_huetocatl

Figs 77–78 200 Map 5

#### Type material.

Holotype female: under concrete blocks, Las Golondrinas, Huixtla, Chiapas, México, 15°25.747'N, 92°39.270'W, ~4667', 22.IX.2004, S. Crews, U.O.G. Vázquez, A. Mendoza, SCC04_020 (CNAN sel_045).

#### Other material examined.

MÉXICO: Chiapas: Pueblo Nuevo Solistahuacan, 17.IX.2004, 17°11.550'N, 92°54.875'W, ~3284', A. Mendoza, under bark on cut down pine tree, en cultivos, dermaptera also found, molted to adult 20.IX.204, SCC04_018, 1♀ (EME sel_043); Motozintla de Mendoza, Chevolcán, 15°20'52.4"N, 92°19'25.4"W , 21.IX.2004, 1752 m, U.O.G. Vázquez, under rocks on outcrop along road cut, SCC04_019, 1♀, 2 imm. (CNAN sel_029,030, 038).

#### Etymology.

The specific epithet comes from a combination of words, huei=big and tocatl=spider, in the Nahau language, indigenous to the region where the spider is found, and refers to the large size of this species. The name is to be treated as a noun in apposition.

#### Diagnosis.

Females can be distinguished from others by the large and posteriorly diamond-shaped median septum, the presence of epigynal pockets, and internally, the ducts are branched (Figs 77–78). Males unknown.

#### Description.

*Holotype female:* Color:carapace dark brown, hint of red, darker laterally; sternum brown; chelicerae uniformly dark reddish-brown; maxillae brown, lightening distally; labium brown, lightening distally; ventrally dusky grey with no markings; legs brown, darkening distally, annulations visible. Carapace: 0.87 times longer than broad; fovea longitudinal, broad, very shallow. Eyes:AER nearly straight; PER slightly recurved; PME larger than AME, PLE largest, ALE smallest; eye diameters, AME 0.33, ALE 0.13, PME 0.38, PLE 0.45; interdistances AME-PME 0.10, PME-ALE 0.33, ALE-PLE 0.70. PME-PME 2.0. ALE-ALE 3.49; ocular quadrangle AME-AME 0.80, PLE-PLE 3.50; clypeus 0.10 high. Mouthparts:chelicerae with stout setae medially and anteriorly; maxillae longer than broad, with tuft of conspicuous setae distally; labium distally rounded. Sternum:1.28 times longer than broad, posteriorly indented. Legs:leg I=lV, but only slightly shorter than II; leg formula 234=1; scopulae present on tarsi of all legs and metatarsi of legs I and II; tarsi I-IV with strong claw tufts; pr claw per foot slightly toothed; spination: leg I, Fm pr 1–1–0, d 1–1–1, rl 1–1–1; Ti d 0, v 2–2–2; Mt v 2–2; leg II, Fm pr 1–0–0, d 1–1–1, rl 1–1–1; Ti v 2–2–2; Mt v 2–2; leg III, Fm pr 1–0–0, d 1–1–1, rl 1–1–1; Ti v 2–2–0; Mt v 2–1; leg IV, Fm pr 1–0–0, d 1–1–1, rl 0–0–1; Ti v 2–2–0; Mt v 2–2. Abdomen:without terminal setal tufts. Pedipalp:claw with 11 teeth. Epigyne:conspicuous median septum, posteriorly diamond-shaped, genital openings located at lateral margins of septum, epigynal pockets present; internally, ducts branched multiple times, fertilization ducts located posteriorly, directed anterolaterally, posterodorsal fold present, doesn't cover internal ducts (Figs 77–78). *Dimensions:* Total length 13.13. Carapace length 6.55, width 7.55. Sternum length 3.80, width 3.00. Abdomen length 6.63, width 5.90. Pedipalp: Fm 2.00, Pt 0.80, Ti 1.00, Ta 2.50, total 6.30. Leg I: Fm 6.75, Pt 3.50, Ti 7.00, Mt 5.00, Ta 2.00, total 24.25. Leg II: Fm 8.00, Pt 3.50, Ti 7.00, Mt 6.00, ta 2.50, total 27.00. Leg III: Fm 8.00, Pt 3.40, Ti 7.00, Mt 6.00, Ta 2.00, total 26.40. Leg IV: Fm 6.75, Pt 2.75, Ti 6.75, Mt 6.00, Ta 2.00, total 24.25.

#### Natural history.

Collected under bark, rocks and concrete blocks in cloud forests (Fig. 200). It has been found with *Selenops mexicanus*.

#### Distribution.

Southern México in the state of Chiapas (Map 5).

### Selenops bifurcatus

Banks, 1909

http://species-id.net/wiki/Selenops_bifurcatus

**Map 8.**
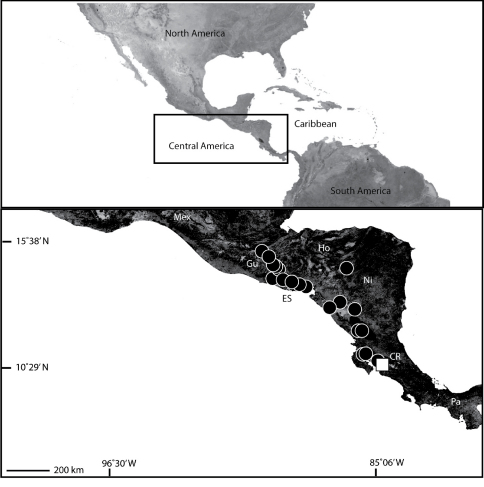
Map of Central America (inset) showing the distribution of two species of *Selenops* in the region. *Selenops bifurcatus* Banks (black circles) and *Selenops oricuajo* sp. n. (white square). (Mex= México, Gu=Guatemala, ES=El Salvador, Ho=Honduras, Ni=Nicaragua, CR=Costa Rica, Pa=Panamá).

Figs 79–82 182 184 201 Map 8

Selenops bifurcatus Banks 1909: 214, pl. 5, Fig. 3 (♂, examined; misidentification of female, not *Selenops bifurcatus*).
Selenops salvadoranus Chamberlin 1925: 218 (♀, examined), syn. n.
Selenops salvadoranus :Muma 1953: 18, Fig. 30 (♀).
Selenops salvadoranus :Kraus 1955: 53, Figs. 139–141 (♀, ♂).

#### Type material.

Holotype male: Oricuajo, Costa Rica, Biolley and Tristan (MCZ, examined).

#### Notes.

The holotype female of *Selenops salvadoranus* Chamberlin, 1925 from San Salvador, El Salvador, I.1920 (MCZ, examined) is in every way identical to females collected with males of S. *bifurcatus* Banks, 1909, the name is to be synonymized.

#### Other material examined.

COSTA RICA: Guanacaste: Palo Verde Field Station, vic. OTES office, hill behind office, 10°20'42.5"N, 85°20'19.1"W, 17.I.2008, ~31 m, S. Crews, R. Duncan, SCC08_021, 1 imm. (EME sel_987); Palo Verde National Park, Cueva Las Tigres, 10°21'58.9"N, 85°21'14.2"W, 17.I.2007, ~31 m, dry limestone forest, S. Crews, under bark, 1 imm. (CAS sel_991). **EL SALVADOR: Chalatenango:** Mun: Chalatenango, La Cueva del Corridor, 6.I.2008, ~1147 m, under rocks in and around cave, S. Crews, R. Duncan, P. Berea, J. Carver, SCC08_010, 4♀, 1 imm. (EME sel_925-929) Mun. Tejutto, Canton Apsectos, Rest. Eucalyptos, 14°12'20.5"N, 89♂06'43.9"W, ~447 m, S. Crews, R. Duncan, P. Berea, J. Carver, under rocks, SCC08_008, 1♂ (CAS sel_921). **Conchagua:** Volcán Conchagua, near La Uníon, 13°18'14.1"N, 87°51'19.6"W, 4.I.2008, ~281 m, S. Crews, R. Duncan, J. Carver, P. Berea, under rocks, SCC08_006, 1p♂, 2 imm. (EME sel_912, 914-915). **La Uníon:** Mun. El Carmen, Lotificacion Amaya, 13°21'44.9" N, 87°59'58.2" W, 5.I.2008, ~297 m, S. Crews, R. Duncan, P. Berea, J. Carver, SCC08_007, 3♀, 1♂, 1 imm. (CAS sel_916, 918-920). **San Salvador:** 1928, S. Calderon, 1♀ (USNM); I.1920, several (USNM); 700 m, 5.V.1905, Schuster, 1♀ (AMNH); Museo Nacional de Historia Natural, 13°40'23.4"N, 89°11'53.6"W, ~397 m, 3.I.2008, S. Crews, R. Duncan, P. Berea, J. Carver, SCC08_003, 2♀, 4♂, 2 imm. (EME sel_881-887, 889). **San Vicente:** Mun. Tepetitán, vic. Finca El Carmen 13°37'53.0"N, 88°50'19.5"W, ~732 m, 4.I.2008, S. Crews, R. Duncan, SCC08_005, 2♀, 2♂, 1p♂, 1 imm. (CAS sel_899, 901, 903-905, 910); vic. San Vicente, off of road to Zacatecolouca near gentlemen's club ‘Dreamed Girl', 13°37'43.4"N, 88°46'49.6"W, ~397 m, 3.I.20, S. Crews, R. Duncan, P. Berea, J. Carver, under rocks at night, SCC08_004, 1♀, 1♂, 5 imm. (EME sel_890-896). **GUATEMALA: Chiquimula:** 1250', 23-23.VII.1947, C. and P. Vaurie, 1♂ (AMNH). **Zacapa:** Zacatán, Las Guacamayas, Carretera Santa Rosalia Marmo, Hídroelectrica Pasabíen, 15°01'39.7"N, 89°41'41.2"W, 1-2.I.2008, S. Crews, R. Duncan, P. Berea, J. Carver, dry thornscrub, under rocks and bark, SCC08_002, 1♀, 2♂, 2p♂, 6 imm. (CAS sel_869-877, 879-880). **NICARAGUA: Altagracia:** Lago Nicaragua, Isla Ometeme, Charco Verde, Volcán Concepción, around Hotel Finca Vincenzia, 11°28'42.6"N, 35°38'20.6"W, ~67 m, 12.I.2008, S. Crews, R. Duncan, P. Berea, under bark of fence posts and trees, under roof shingles on ground, SCC08_014, 1p♂, 3 imm. (EME sel_943, 945-946, 948); Lago Nicaragua, Isla Ometeme, across the street and up the hill from Hotel Vinca Vincenzia, ~84 m, 11°29'31.2"N, 85°38'14.1"W, S. Crews, R. Duncan, P. Berea, under bark of *Bursera*, SCC08_015, 1p♂, 1 imm. (CAS sel_951, 953). **Boaco:** Aguascalientes, Alc. Teustepe, Camino La Cuesta, 12°22'57.8"N, 85°47'30.7"W, ~195 m, 15.I.2008, S. Crews, R. Duncan, P. Berea, SCC08_020, 1♀, 4 imm. (EME sel_963, 976, 979-981). **Leon:** Alc. El Jicaral, Camino Santa Rosa, Puente la Guayabita, 12°44'31.2"N, 86°22'44.6"W, ~122 m, 14.I.2008, under rocks, S. Crews, R. Duncan, P. Berea, J. Carver, SCC08_017, 3♀, 3 imm. (CAS sel_961-962, 964, 967, 969-970); Alc. San Jacinto, Mina El Límon, Rancho Las Brisas, off road from Telica to San Isidro, 12°37'03.8"N, 86°44'34.3"W, ~87 m, 14.I.2008, S. Crews, R. Duncan, P. Berea, J. Carver, SCC08_016, 1p♂, 5 imm. (EME sel_954-955, 957-960). **Madríz:** Alc. Ocotal, Totogalpa, 11.I.2008, 13°33'49.5"N, 86°29'64.6"W, ~672 m, S. Crews, under rocks, SCC08_013, 2♀, 1♂, 4 imm. (CAS sel_931-933, 936-939).

#### Diagnosis.

Males can be distinguished from other species by the large, bifurcated MA that somewhat resembles an articulating claw (Fig. 79). In many specimens the second branch is obscured by the conductor. The RTA is also distally bifurcated (Fig. 80). Females can be distinguished from others by having the posterior margin of the epigynal plate extend beyond the epigastric furrow, and a septum that appears to befused to the epigynal plate posteriorly (Figs 81–82).

#### Remarks.

Banks (1909) first described a male and female collected from Oricuajo, Costa Rica by Biolley and Tristan. Presumably they were collected together. The female was not illustrated. Muma (1953) redescribed these specimens, illustrating both the male and the female. Chamberlin (1925) described the female of *Selenops salvadoranus*, but made no illustration. Muma (1953) also redescribed and illustrated this type. Kraus (1955) described and illustrated both the male and female of *Selenops salvadoranus*. The male matched the male of *Selenops bifurcatus* from both Banks (1909) and Muma (1953). The illustration of the female was clearly that of *Selenops salvadoranus* as illustrated by Muma (1953). It seems as though Kraus (1955) was aware of Muma's (1953) work, but perhaps was unable to look at the actual specimens. In many instances, the innermost branch of the MA is obscured by the conductor, in which case, the MA appears to be single branched, rather than double branched. It would appear that this was the case when Muma (1953) illustrated the type, which upon closer examination does have a two branched MA. Several species of selenopids have overlapping distributions. Some species have widespread distributions, while others have small distributions. Thus, even collecting a male and female from the same locality does not guarantee that they are same species. Unless a male and female are collected mating, or collected simultaneously from multiple localities, or their identities can be cross-checked with DNA data, it is recommended that they not be described as the same species, as this case illustrates. I have collected the same male and female from multiple localities across Central America, they have mated and produced offspring in the lab, and they have matching DNA sequences for multiple genes. In conclusion, the name *Selenops salvadoranus* has been synonymized with *Selenops bifurcatus*, and what was originally designated as the female of *Selenops bifurcatus* is here described as *Selenops oricuajo* sp. n. (see below).

#### Description.

*Holotype male*: Color. carapace (holotype) dusky orange with white setae (recent) dusky yellow, duskier laterally; sternum light yellow; chelicerae (holotype) same color as carapace, with dusky longitudinal lateral stripes and one dusky medial area (recent) same color as carapace, with duskier marks; maxillae (holotype) yellow brown (recent) light yellow; labium (holotype) dusky yellow brown, darker laterally (recent) pale yellow, lightening distally; abdomen dorsally (holotype) greyish-tan (recent) greyish-tan, mottled with darker spots; ventrally light yellow; legs (holotype), same color as carapace, annulations not visible, but ventral surface of femur I dusky (recent) legs light yellow, annulations on femur not visible, but dusky area ventrally, patella and tibia with rings. Carapace:0.90 times longer than broad. Eyes:AER slightly recurved; PER recurved; PME larger than AME, PLE largest, ALE smallest; eye diameters, AME 0.23, ALE 0.10, PME 0.33, PLE 0.50; interdistances AME-PME 0.08, PME-ALE 0.28, ALE-PLE 0.30. PME-PME 1.28. ALE-ALE 2.30; ocular quadrangle AME-AME 0.45, PLE-PLE 2.25; clypeus 0.20 high. Mouthparts:chelicerae with few scattered setae medially and anteriorly; maxillae longer than broad, with tuft of conspicuous setae distally; labium distally rounded. Sternum:as long as broad, posteriorly indented. Legs:leg I only slightly shorter than II and III; leg formula 4321; scopulae present on distal end of all 4 tarsi; tarsi I-IV with strong claw tufts; pr claw toothed, rl claw with fewer teeth; spination: leg I, Fm pr 1–1–1, d 1–1–1, rl 1–1–1; Ti pr 1–0–1, rl 1–0–1, d 1–1–0, v 2–2–2; Mt pr 1–1–0, rl 1–1–0, v 2–2; leg II, Fm pr 1–1–1, d 1–1–1, rl 1–1–1; Ti pr 1–0–1, d 1–1–0, rl 1–0–1, v 2–2–2; Mt pr 1–1–0, v 2–2, rl 1–1–0; leg III, Fm pr 1–1–1, d 1–1–1, rl 1–1–1; Ti pr 1–0–1, rl 1–0–1, d 1–1–0, v 2–2; Mt pr 1–1–0, rl 1–1–0, v 2–1–1; leg IV, Fm pr 1–1–1, d 1–1–1, rl 1–1–1; Ti pr 1–0–1, rl 1–0–1, d 1–1–0, v 2–2; Mt pr 1–1–0, rl 1–1–0, v 2–1–1. Abdomen:without terminal setal tufts. Pedipalp:Fm, spination d 0–1–4; cymbium oval in ventral view, slightly angled posterolaterally; conductor large angular structure arising from the side and medially,curved laterally, tapering upwards, pointed at tip; embolus long, slender, curved, beginning at 6 o'clock, terminating at 12 o'clock, not quite extending to lateral edge, but more medial, tapering distally; MA arising at 2 o'clock, with two long, finger-like branches, directed laterally, base wide; RTA with 2 apophyses, one curving in toward cymbium, small, quadrangular, one curving away from cymbium, with 3 distal projections, one rounded and curved, the other two forming a y-shaped distal bifurcation, RTA extending at least one third the length of cymbium in ventral view (Figs 79–80). *Dimensions:* Total length 9.75. Carapace length 4.75, width 5.30. Sternum length 2.00, width 2.00. Abdomen length 5.00, width 3.53. Pedipalp: Fm 1.75, Pt 0.40, Ti 0.70, Ta 1.00, total 3.85. Leg I: Fm 4.75, Pt 1.75, Ti 4.40, Mt 4.00, Ta 2.90, total 17.80. Leg II: Fm 5.50, Pt 1.90, Ti 5.00, Mt 4.50, Ta 1.90, total 18.80. Leg III: Fm 5.75, Pt 1.75, Ti 4.65, Mt 4.75, Ta 1.75, total 17.65. Leg IV: Fm 5.75, Pt 1.75, Ti 5.00, Mt 5.00, Ta 1.75, total 19.25.

*Female (holotype of* *Selenops salvadoranus)*: Color: carapace (holotype of *Selenops salvadoranus*) red-brown with white setae (recent) dusky yellow-brown overall, more brown in cephalic region and medially, more yellow laterally, with light setae in the cephalic region and on laterally; sternum orange-brown, darker around border; chelicerae (holotype of *Selenops salvadoranus*) brown (recent) orange-brown, darker laterally; maxillae orange-brown, lightening distally; labium dark brown, lightening distally; abdomen dorsally (holotype of *Selenops salvadoranus*) light orange-brown, wrinkled, no distinct marks (recent) light grey brown, speckled with darker spots, dark caudal festoon present; ventrally dusky yellow grey, no markings; legs (holotype of *Selenops salvadoranus*) legs orange-tan, no markings apparent; (recent) legs yellowish, annulations present on all segments. Carapace:0.96 times longer than broad. Eyes:AER slightly recurved; PER recurved; PME larger than AME, PLE largest, ALE smallest; eye diameters, AME 0.23, ALE 0.15, PME 0.43, PLE 0.53; interdistances AME-PME 0.10, PME-ALE 0.20, ALE-PLE 0.53. PME-PME 1.53. ALE-ALE 2.50; ocular quadrangle AME-AME 0.55, PLE-PLE 2.80; clypeus 0.17 high. Mouthparts:chelicerae with stout setae medially and anteriorly; maxillae longer than broad, with tuft of conspicuous setae distally; labium distally rounded. Sternum:1.20 times longer than broad, posteriorly indented. Legs:leg formula 4321 (Muma 1953); scopulae present on tarsi of all legs and metatarsi of legs I and II; tarsus I-IV with strong claw tufts; both claws with similar number of teeth; spination: leg I, Fm pr 1–1–0, d 1–1–1, rl 1–1–1; Ti d 0, v 2–2–2; Mt v 2–2; II, Fm pr 1–1–0, d 1–1–1, rl 1–1–1; Ti v 2–2–2; Mt v 2–2; III, Fm pr 1–1–0, d 1–1–1, rl 1–1–1; Ti v 2–2–0; Mt v 2–1–1. Abdomen:without terminal setal tufts. Pedipalp:Claw with 15 teeth. Epigyne:epigynal plate extending beyond epigastric furrow, truncate posteriorly, median septum present, sinuous laterally, genital openings located anterolaterally to septum, epigynal pockets absent; internally, ducts directed slightly posteriorly, then laterally, fertilization ducts located laterally, small posterodorsal fold present covering posterior margin of internal ducts (Figs 81–82). *Dimensions:* Total length 11.85. Carapace length 5.65, width 5.90. Sternum length 3.00, width 2.50. Abdomen length 6.20, width 4.75. Pedipalp: Fm 1.60, Pt 0.75, Ti 1.00, Ta 2.00, total 5.35. Leg I: Fm 5.25, Pt 2.00, Ti 4.75, Mt 3.80, Ta 1.75, total 17.55. Leg II: Fm 5.00, Pt 2.00, Ti 4.25, Mt 3.75, Ta 2.00, total 17.00. Leg III: Fm 6.50, Pt 2.00, Ti 5.00, Mt 4.50, Ta 2.00, total 20.00. Leg IV: Missing.

#### Natural history.

Found under rocks and other debris on the ground, under the bark of trees and on fence posts and around human dwellings in a variety of habitats (Fig. 200). The egg sac is a white to yellowish flat disc that is either guarded by the female or not, and is either hidden or not (Fig. 182). A female will lay eggs within a few weeks of mating. One female made 3 egg sacs, each containing 40–70 eggs. This species is widely distributed and, thus, is also found with other species of selenopid, in particular *Selenops mexicanus* and *Selenops oricuajo* sp. n.

#### Distribution.

Found from southeastern Guatemala to northwestern Costa Rica (Map 8).

### Selenops buscki

Muma, 1953

http://species-id.net/wiki/Selenops_buscki

Figs 83–84 Map 3

Selenops buscki Muma 1953: 19, Fig. 31 (♀).

#### Type material.

Holotype female from Taboga Island, Panamá, VI.1911, A. Busck (USNM, examined).

#### Diagnosis.

Females can be separated from all other species by the centrally located median lobe, the epigynal pockets, and the shape of internal ducts (Figs 83–84). Males unknown.

#### Description.

*Holotype female*: Color:carapace orange-brown; chelicerae orange-brown, darker laterally; maxillae dark yellow; abdomen damaged, dorsally cream-colored, no noticeable markings; legs dusky orange, no markings visible; Carapace:0.95 times longer than broad. Eyes:AER nearly straight; PER recurved; PME larger than AME, PME largest, ALE smallest; eye diameters, AME 0.18, ALE 0.05, PME 0.25, PLE 0.20; interdistances AME-PME 0.10, PME-ALE 0.18, ALE-PLE 0.45. PME-PME 1.15. ALE-ALE 1.90; ocular quadrangle AME-AME 0.40, PLE-PLE 2.10. Mouthparts:chelicerae with stout setae medially and anteriorly; maxillae longer than broad, with tuft of conspicuous setae distally; labium distally rounded. Sternum:posteriorly indented. Legs: leg formula 4321 (Muma, 1953); scopulae present on tarsi of all legs and metatarsi of legs I and II; tarsi I-IV with strong claw tufts; spination: leg I, Fm pr 1–1–0, d 1–1–1, rl 1–1–1; leg II, Fm pr 1–1–0, d 1–1–1, rl 1–1–1; Ti v 2–2–2; Mt v 2–2; leg III, Fm pr 1–1–0, d 1–1–1, rl 1–1–1; Ti v 2–2–0; Mt v 2–1–1; leg IV, missing. Abdomen:without terminal setal tufts. Epigyne:lateral lobes conspicuous, median septum a small teardrop-shaped lobe, epigynal pockets present; internally, ducts and spermathecae located medially, fertilization ducts located posteriorly, directed laterally, very small posterodorsal fold present, not covering any part of internal ducts (Figs 83–84). *Dimensions:* Total length 8.30. Carapace length 4.00, width 4.20. Abdomen length 4.30, width 2.65. Leg I: Fm 3.40, Pt 1.70, Ti missing, Mt missing, Ta missing, total missing. Leg II: Fm 3.20, Pt 1.20, Ti 2.50, Mt 3.40, Ta 1.10, total 11.40. Leg III: Fm 3.80, Pt 1.50, Ti missing, Mt missing, Ta missing, total missing. Leg IV: Missing.

#### Natural history.

No data.

#### Distribution.

Known only from the island of Taboga in Panamá (Map 3).

### Selenops oricuajo sp. n.

urn:lsid:zoobank.org:act:2E813C62-BEB7-4194-953D-F95D86514F3B

http://species-id.net/wiki/Selenops_oricuajo

Figs 85–86 Map 8

Selenops bifurcatus Muma, 1953: 20, fig. 35 (misidentification: ♀ only).

#### Type material.

Holotype female: Oricuajo, Costa Rica, Biolley and Tristan (MCZ).

#### Etymology.

This species is named after the type locality, Oricuajo, and is to be treated as a noun in apposition.

#### Diagnosis.

**Figures 85–92.**
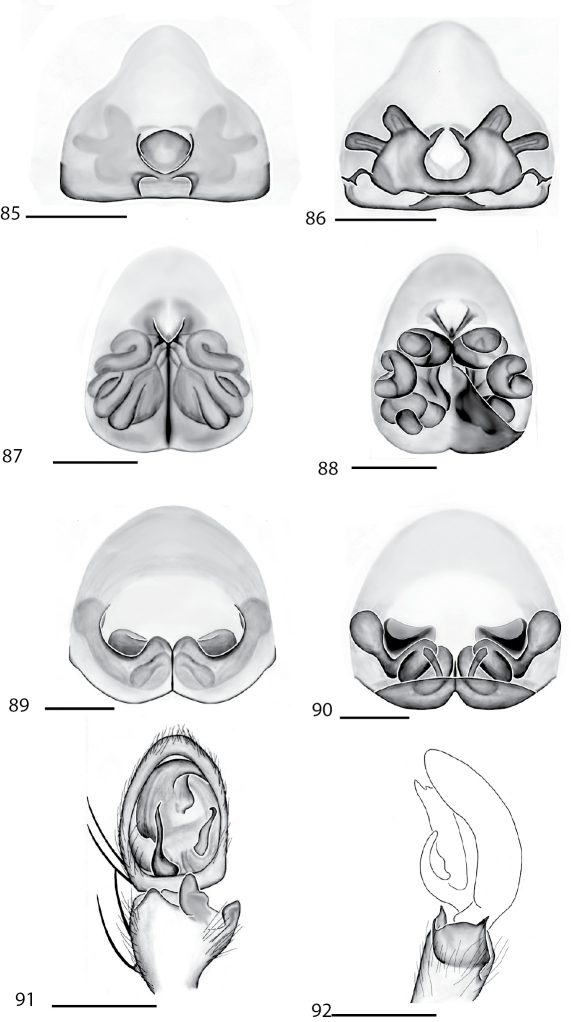
Copulatory organs of *Selenops oricuajo* sp. n. female from Oricuajo, Costa Rica (MCZ) **85–86** *Selenops amona* sp. n. holotype female from Bajura Empalme, Isla Mona (EME sel_847) **87–88** *Selenops lindborgi* Petrunkevitch holotype female from Santa Maria Bay, St. Thomas, United States Virgin Islands (MCZ) **89–90** male from Christianstead, St Croix, United States Virgin Islands (MCZ) **91–92, 85, 87, 89** epigyne, ventral view **86, 88, 90** spermathecae, dorsal view **91** left pedipalp, ventral view **92** left pedipalp, retrolateral view. Scale bar = 0.50 mm (85–86, 89–92), 0.30 mm (87–88).

Females can be separated from other species by the epigynum which has a conspicuous angular median septum located in the center of the epigynal plate, and by the epigynal pockets, and the internal ducts which have two branches (Figs 85–86). Males unknown.

#### Description.

*Holotype female:* Color: carapace dusky orange with white setae; sternum light yellow; chelicerae same color as carapace, with dusky longitudinal lateral stripes and one dusky area in the center, not extending all the way to the top of the chelicerae; maxillae dusky yellow brown, darker on sides; abdomen dorsally grey to cream-colored with dark laterocaudal festoon; legs light yellow, darkening distally, with annulations on anteroventral faces of anterior femora. Carapace:0.95 times longer than broad. Eyes:AER nearly straight; PER recurved; PME larger than AME, PLE largest, ALE smallest; eye diameters, AME 0.15, ALE 0.03, PME 0.18, PLE 0.30; interdistances AME-PME 0.10, PME-ALE 0.28, ALE-PLE 0.30. PME-PME 1.00. ALE-ALE 1.83; ocular quadrangle AME-AME 0.38, PLE-PLE 1.80. Legs:leg formula 4321 (Muma, 1953); spination not available, legs completely disarticulated. Mouthparts:chelicerae with stout setae medially and anteriorly; maxillae longer than broad, with tuft of conspicuous setae distally; labium distally rounded. Abdomen:without terminal setal tufts. Epigyne:angular to quadrate median lobe, truncate posteriorly, epigynal pockets present, genital openings located at anterolateral margins of median lobe; internally, ducts large, branching into two heavily sclerotized lateral branches, fertilization ducts located posterolaterally and directed posteriorly, small posterodorsal fold present, does not cover ducts (Figs 85–86). *Dimensions:* Total length 10.45. Carapace length 3.60, width 3.80. Abdomen length 6.85, width 4.65.

#### Natural history.

All that is known of this species' natural history is that it has been collected with *Selenops bifurcatus*.

#### Distribution.

Known only from the type locality (Map 8).

### Selenops amona sp. n.

urn:lsid:zoobank.org:act:3F63D138-00A6-4D0E-8C06-75791F496D53

http://species-id.net/wiki/Selenops_amona

Figs 87–88 Map 9

#### Type material.

Holotype female: Bajura Empalme, Isla Mona, I.2007, A. Puente-Rolón (EME sel_847).

#### Other material examined.

PUERTO RICO: Isla Mona: same data as holotype, 1 imm. (EME sel_846); Camino de los Cobros, VI.2006, A. Puente-Rolón, SCC06_059, under bark of *Bursera*, 1 imm. (CAS sel_502); Maricao: Bosque Estatl de Maricao, 18°08'51.2"N, 66°59'35.0"W, 10.VI.2006, 700–800 m, S. Crews, A. Puente- Rolón, serpentine forest, 1 imm. (CAS sel_440).

#### Etymology.

The specific name comes from the Taino word ‘amona', the indigenous name for the type locality. It is to be treated as a noun in apposition.

#### Diagnosis.

Females can be separated from other species by the extensively coiled internal ducts (Figs 87–88). Males unknown.

#### Description.

*Holotype female:* Color: carapace light tan-yellow, slightly darker in cephalic region, dusky around edges; sternum pale yellow; chelicerae light yellowish-tan, dusky markings anteromedially, progressively becoming darker to black at the distal end of the chelicerae; maxillae pale yellow; labium pale yellow; abdomen dorsally yellowish-orange, dark on sides, with laterocaudal festoon; lanceolate stripe located medially extending about half the length of the abdomen, faded spot anteriorly, 2 more spots just below, 2 more below that, and 3 spots at posterior margin of abdomen; ventrally cream-colored laterocaudally; legs pale yellow, darkening distally, annulations present. Carapace: 0.89 times longer than broad; fovea longitudinal, broad, very shallow. Eyes:AER nearly straight; PER slightly recurved; PME larger than AME, PME same as PLE, ALE smallest; eye diameters, AME 0.10, ALE 0.08, PME 0.25, PLE 0.25; interdistances AME-PME 0.03, PME-ALE 0.15, ALE-PLE 0.23. PME-PME 0.98. ALE-ALE 1.68; ocular quadrangle AME-AME 0.30, PLE-PLE 1.70; clypeus 0.08 high. Mouthparts:chelicerae with stout setae medially and anteriorly; maxillae longer than broad, with tuft of conspicuous setae distally; labium distally rounded. Sternum: 1.14 times longer than broad, posteriorly indented. Legs:leg I only slightly shorter than legs II, III and IV; leg formula unknown (at least one leg missing); legs II and III equal in length, with I just slightly shorter; scopulae present on all 4 tarsi and metatarsi and tibia I and II; tarsi I-IV with strong claw tufts; pr claw per foot slightly toothed; spination: leg I, Fm pr 1–1–1, d 1–1–1, rl 1–1–1; Ti d 0, v 2–2–2; Mt v 2–2; leg II, Fm pr 1–1–0, d 1–1–1, rl 1–0–1; Ti v 2–2–2; Mt v 2–2; leg III, Fm pr 1–1–1, d 1–1–1, rl 1–1–1; Ti v 2–2–0; Mt v 2–1. Abdomen:with terminal setal tufts. Pedipalp:claw with 5 teeth. Epigyne:lateral lobes fused medially for nearly 2/3 the length of the plate, small v-shaped medial area located anteriorly, genital openings located at lateral margins of v-shape, epigynal pockets absent; internally, wide ducts coiled multiple times, mostly symmetrical, though not exactly, large posterodorsal fold, covering a large part of the internal ducts (Figs 87–88). *Dimensions:* Total length 6.33. Carapace length 3.00, width 3.38. Sternum length 1.60, width 1.40. Abdomen length 3.33, width 2.48. Pedipalp: Fm 1.00, Pt 0.25, Ti 0.50, Ta 0.80, total 2.55. Leg I: Fm 3.50, Pt 1.00, Ti 3.50, Mt 2.25, Ta 1.00, total 11.25. Leg II: Fm 3.50, Pt 1.00, Ti 3.50, Mt 2.50, Ta 1.00, total 11.50. Leg III: Fm 3.75, Pt 1.00, Ti 3.00, Mt 2.75, Ta 1.00, total 11.50. Leg IV: Missing.

#### Natural history.

Found under bark and rocks.

#### Distribution.

**Map 9.**
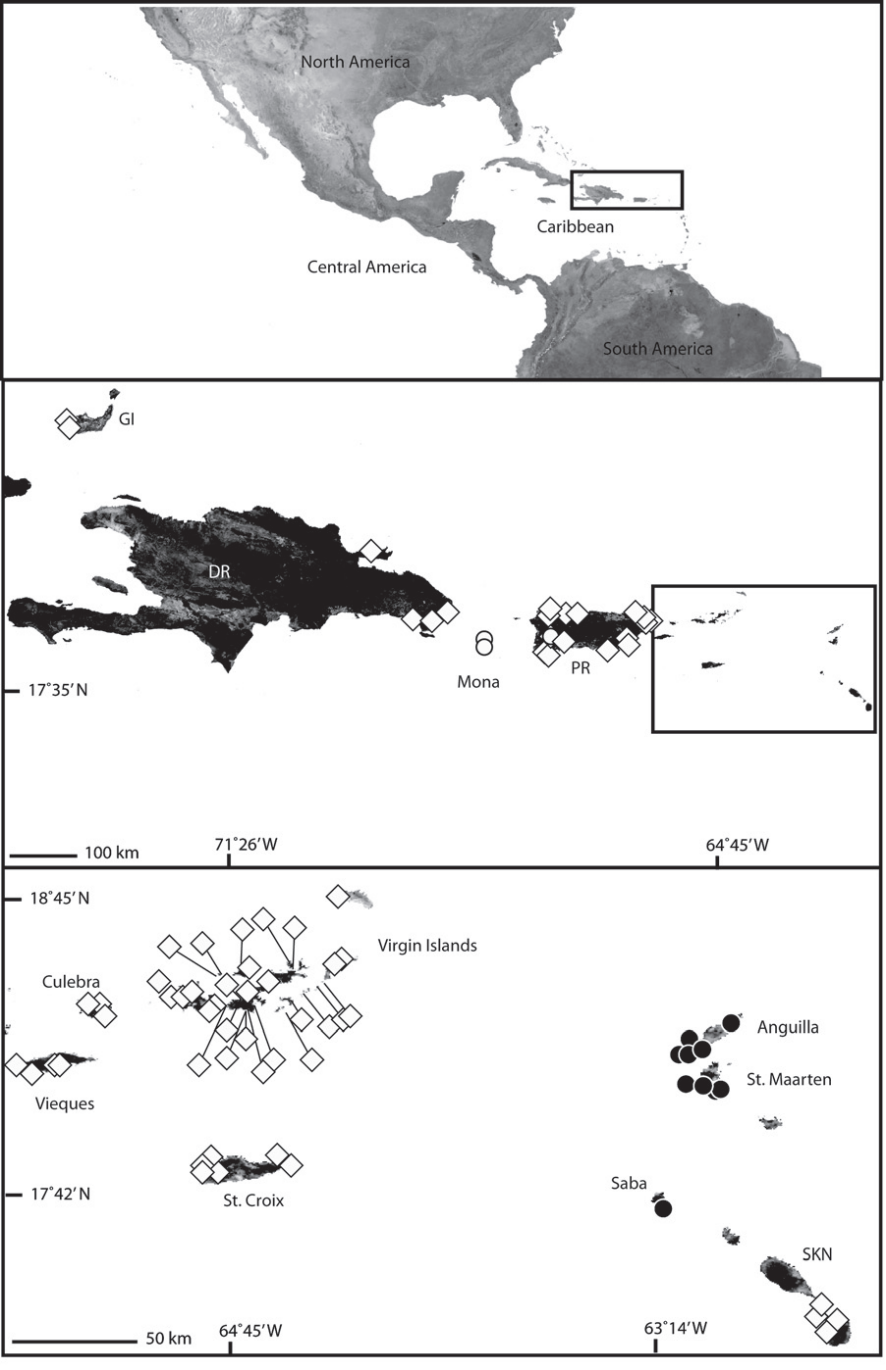
Map of the northern Caribbean (insets) showing the distribution of three species of *Selenops* in the region, *Selenops lindborgi* Petrunkevitch (white diamonds), *Selenops souliga* sp. n. (black circles) , and Selenops amona (ital.) sp. n. (white circles). Due to the large difference in scale of the different islands, there are two insets. (Inset 1: GI=Great Inagua, DR=Dominican Republic, PR=Puerto Rico (Inset 2: SKN=St. Kitts and Nevis).

Known from Isla Mona and a single locality in western Puerto Rico (Map 9).

### Selenops lindborgi

Petrunkevitch, 1926

http://species-id.net/wiki/Selenops_lindborgi

Figs 89–92 185–186 Map 8

Selenops lindborgi Petrunkevitch 1926: 55, fig. 16 (♀, examined).
Selenops lindborgi :Petrunkevitch 1930: 38, figs 28–29 (♀).
Selenops longipes Petrunkevitch 1930: 36, figs 26–27 (♂).
Selenops lindborgi :Bryant 1942: 349, figs 29, 37 (♂, ♀).
Selenops lindborgi :Muma 1953: 34, figs 57–59 (♂, ♀).

#### Type material.

Holotype female: Santa Maria Bay, St. Thomas, Virgin Islands, under bark, 28.VII.1925 (MCZ).

#### Note.

Muma (1953: 35) designated a male specimen from Christiansted, St. Croix, United States Virgin Islands (MCZ) as an ‘allotype'.

#### Other material examined.

BAHAMAS: Great Inagua: Man O' War Bay, 21°04'30.2"N, 73°38'36.7"W, ~4 m, 16.V.2006, dry forest with limestone rocks, S. Crews, under bark, SCC06_007, 1♂ (CAS sel_316); Old Aerostat Base, 21°06'06.7"N, 73°39'01.9"W, ~2 m, 16.V.2006, S. Crews, under bark of *Casuarina*, SCC06_008, 2 imm. (CAS sel_317-318). **BRITISH VIRGIN ISLANDS: Anegada:** west end, 4.VI.1966, Island Project Staff, University of Puerto Rico, 1♂ (AMNH). **Dead Man's Chest:** 26.V.1966, Island Project Staff, University of Puerto Rico, 1♂, 2 imm. (AMNH). **Guana Island:** north side of island near beach house, 18°28.793'N, 64°34.473'W, 18.X.2004, S. Crews, D. Hamilton, on outside of house, during day, SCC04_047, 2♀, 2 imm. (EME sel_071, 081, 085, 122); south side of island near club house, trees near salt pond, 18°28.619'N, 64°34.475'W, 18.X.2004, S. Crews, D. Hamilton, SCC04_046; 4 imm. (CAS sel_079, 084, 086, 087). **Island #9:** Spaghetti Key, Island Project Staff, University of Puerto Rico, 1♂. **Island #16:** Julie Key, 12-16.I.1966, Island Project Staff, University of Puerto Rico, 1♂ (AMNH). **Cooper Island,** 23.V.1966, Island Project Staff, University of Puerto Rico, 1♀, 2 imm. (AMNH). **Ginger Island:** 25.V.1966, Island Project Staff, University of Puerto Rico, 1♀ (AMNH). **Great Tobago Island:** 2.IV.1966, Island Project Staff, University of Puerto Rico, 1♀, 3 imm. (AMNH). **Little Jost Van Dyke:** 27.VII.1965, Island Project Staff, University of Puerto Rico, 1♀, 1 imm. (AMNH). **Little Tobago:** 4.IV.1966, Island Project Staff, University of Puerto Rico, 1♀ (AMNH). **Norman Island:** 26.V.1966, Island Project Staff, University of Puerto Rico, 1♀, 1 imm. (AMNH). **Peter Island:** 9.VII.1965, Island Project Staff, University of Puerto Rico, 1♀ (AMNH); 12.V.1966, P. Chubb, 1♂ (AMNH). **Sandy Key:** near Tortola, 31.VIII.1965, H., A. Heatwole, 1♀, 1♂ (AMNH). **Tortola:** vic. Sage Mountain, down road from park entrance, out of park, 20.X.2004, S. Crews, D. Hamilton, under concrete block on porch, SCC04_049, 1p♂ (MUS sel_078). **Virgin Gorda:** Virgin Gorda Mountain, 26.VI.1966, Island Project Staff, University of Puerto Rico, 1♂, 1♀; lower trail up to Gorda Peak, along trail, 18°28.774'N, 64°24.210'W, ~300 m, 19.X.2004, S. Crews, SCC04_048, 1 p♂, 6 imm. (EME sel_073-075, 083, 089, 096, 109). **DOMINICAN REPUBLIC: La Altagracia:** Parque del Este, Boca de Yuma, 18°21.875' N, 68°37.080' W, 29-30.XI.2004, S. Crews, under bark along trail, on ranger's station at night, SCC04_089, 3 imm. (EME sel_195-196, 216); Parque del Este, Guaraguao, 18°19.968'N, 68°48.709'W, 30.XI.2004, sea level, S. Crews, under bark, dry limestone forest, SCC04_090., 2♀, 2♂, 4 imm. (EME sel_200-207); Punta Cana Resort, 5-9.VII.2006, S. Crews, L. Mahler, on trees at night, SCC06_066, 1♂ (MNHNSD sel_529). **Samaná:** Las Terrenas, 28.VII.2006, sea level, 19.32364°N, 69.53108°W, L. Mahler, under bark, 2♀ (MNHNSD sel_590, 592). **San Cristóbal:** Borbon Cuevas Pomier, 200 m, 28.VII-5.VIII 1995, S.,J. Peck, tropical deciduous forest, 1♂ (AMNH). **PUERTO RICO: Arecibo:** Arenalejos, Carretera 657, km 1.9, 18°25'15.9"N, 66°40'35.2"W, 141 m, saw several egg sacs, 7.VI.2006, S. Crews, A. Puente-Rolón, M. Nevaraez, under bark of *Krugiodendrum ferreum*, and *Buersera simaruba*, SCC06_035, 3♂, 1♀ (CAS sel_392-393, 397-398); between Barceloneta and Arecibo, Bosque Cambalacheo, 18°27'07.0"N, 66°35'49.9"W, 9.VI.2006, S. Crews, O. Monzon, under bark of *Bursera* and surrounding trees, dry forest, SCC06_041; 2 imms. (EME sel_421-422). **Blanquilla:** 29.X.1964, H. Heatwole, R. Levins, F. McKenzie, 1♀, 3 imm. (AMNH). **Cayo Caracoles:** 6.I.1966, Island Project Staff, University of Puerto Rico, 1♀ (AMNH). **Ceiva:** Los Corchos, 18°12'13.8"N, 65°40'06.5"W, sea level-3 m, 8.VI.2006, S. Crews, O. Monzon, SCC06_040, 2 imm. (CAS sel_416, 418). **Culebra:** 19.VII.1965, F. McKenzie, 1♀ (AMNH); Brava Beach Trail, 18°19'38.9"N, 65°16'54.1"W, ~9 m, 12.VI.2006, S. Crews, G. Olivieri, dry forest, under bark, SCC06_053, 2♀, 2 imm. (EME sel_486-489); Monte Resaca, 18°19'20.7"N, 65°18'10.5"W, ~146 m, 12.VI.2006, under bark, S. Crews, G. Olivieri, SCC06_052, 1♂, 5 imm. (CAS sel_480-484). **Fajardo:** Seven Seas Public Beach, 18°22'03.7"N, 65°38'04.9"W, sea level–2 m, 8.VI.2006, S. Crews, O. Monzon, dry coastal forest, under bark, SCC06_039, 2♀, 1♂, 1 imm. (EME sel_406, 408, 410, 412-414). **Fundillo Espinoso:** 1-10.I.1966, Island Project Staff, University of Puerto Rico, 1♀, 1 imm. (AMNH). **Guanica:** Punta Ballenas, 17°57'13.6"N, 66°50'57.8"W, ~14 m, 10.VI.2006, dry coastal forest, thorn scrub, S. Crews, A. Puente-Rolón, SCC06_047, 2♂, 5 imm. (CAS sel_454-460). **Humacao:** Barrio Collores, 11.VI.2006, S. Vega, under bark of mahogany, SCC06_051, 3 imm. (EME sel_469, 472, 476). **Isábela:** Bosque de Guajataca, 18°25'16.7"N, 66°57'58.0"W, ~47 m, 9.VI.2006, S. Crews, O. Monzon, under bark of dead tree, SCC06_044, 1 imm. (CAS sel_433). **Isla Cabeza de Perro:** 16.I.1965, H. Heatwole, F. McKenzie, 1♂, 3 imm. (AMNH). **Isla Icacos:** north part of island, 31.V.1964, H. Heatwole, Torres, 2♀ (MCZ). **Isla Romero:** 12-15.I.1966, Island Project Staff, University of Puerto Rico, 1♀ (AMNH); **Isleta Marina:** 17.III.1965, H. Heatwole, R. Levins, F. McKenzie, 1♂ (AMNH). **Isla Mayaguez:** 6-9.I.1966, Island Project Staff, University of Puerto Rico, 1♂, 1♀, 2 imm. (AMNH). **Isla Palominitos:** 17.II.1966, H. Heatwole, F. McKenzie, 1♀, 1 imm. (AMNH); **Loiza:** Punta Vacia, Talega, 18°27'03.8"N, 65°54'16.7"W, ~3 m, 8.VI.2006, S.Crews, O. Monzon, under bark of *Bursera* and sea grape, SCC06_038, 2♂, 4♀ (EME sel_399-404). **Manuabo:** Mariani Creek, 18°00'29.7"N, 65°52'17.0"W, ~34 m, 11.VI.2006, under bark, S. Crews, A. Puente-Rolón, SCC06_050, 1♀, 2 imm. (CAS sel_465-466, 468). **Maricao:** Bosque Estatl de Maricao, 18°08'51.2"N, 66°59'35.0"W, ~700-800 m, 10.VI.2006, S. Crews, A. Puente-Rolón, under rocks, SCC06_045, 2♀, 3 imm. (EME sel_427, 434, 436-437, 439). **Quebradillas:** Merendero de Guajataca, 18°29'23.7" N, 66°56'59.4" W, ~ 46 m, 9.VI.2006, S. Crews, O. Monzon, under bark of *Bursera*, *Casuarina*, sea grape, SCC06_043, 1♂ (EME sel_427). **Sabana Grande:** Susúa State Forest, 18°04'15.0"N, 66°54'31.6"W, ~207 m, 10.VI.2006, S. Crews, A. Puente- Rolón, dry serpentine forest, under rocks, SCC06_046, 1p♂, 4 imm. (CAS sel_444, 446-447, 450). **Salinas:** Reserva Jobos, Parque Jagüeys, 17°57'13.9"N, 66°15'03.5"W, ~28 m, 11.VI.2006, S. Crews, A. Puente-Rolón, under bark of burned *Acacia*, SCC06_049, 1♀ (EME sel_464). **Vieques:** Entrada Caño Hondo NR, Puerta Mosquito, 18°06'11.0"N, 65°27'05.5"W, sea level, 19.VI.2006, S. Crews, E. Bermudez, *Acacia* wetland, SCC06_061, 4 imm. (CAS sel 504-506, 508); entrance to Caño Hondo NR, off road 997, km 5, 18°06'47.6"N, 65°27'13.0"W, ~6 m, 19.VI.2006, S. Crews, E. Bermudez, under bark, SCC06_062, 1 imm. (EME sel_512); Playa Caracas-Laguna Puerto Ferro, Refugio Nacional de Vida Silvestre de Vieques, 18°06'24.5"N, 65°25'25.8"W, ~6 m, 19.VI.2006, S. Crews, E. Bermudez, under bark, SCC06_063, 1♂, 2 imm. (CAS sel_513-515); Ruinas Central Playa Grande, 18°05'43.2"N, 65°31'13.2"W, ~26 m, 19.VI.2006, S. Crews, E. Bermudez, under bark, SCC06_064, 1♀, 3 imm. (EME sel_516-518, 521). **ST. KITTS AND NEVIS: St. Kitts:** Major's Bay, 17°13'37.9"N, 62°38'49.3"W, sea level, 24.II.2007, S. Crews, under bark, SCC07_032, 1♀, 2 imm. (CAS, sel_750-752); SE Peninsula off Dr. Kennedy Simmons Hwy @ Sand Bank Bay, 17°14'59.1"N, 62°38'40.8"W, 24.II.2007, S. Crews, under bark in forest, debris on beach, SCC07_031, 2♀, 4 imm. (EME sel_743, 745-749). **Nevis:** Round Hill entrance to Mt. Nevis, main road toward Cottle Church, dry forest, some palms, 23.II.2006, S. Crews, under bark, lots of egg sacs everywhere, SCC07_029, 1♀, 4 imm. (CAS sel_735-739); Tamarind Bay, Galliput Restaurant, 23.II.2006, S. Crews, SCC07_030, under rock and under bark of tamarind tree, 1♀, 2 imm. (EME sel_740-742). **UNITED STATES VIRGIN ISLANDS: Buck Island:** 14-16.XII.1966, Island Project Staff, University of Puerto Rico, 1♂ (AMNH). **St. Croix:** Butler Bay, west shore road, 17°45'49.7"N, 64°52'58.8"W, sea level, 14.VI.2006, S. Crews, C. Niebuhr, under bark of sea grape, SCC06_054, 1♀, 2 imm. (CAS sel_490-493); Christianstead, H.A. Beatty (MCZ); Radio Telescope Station, east island, 17°45.398'N, 64°35.045'W, 18.XI.2004, S. Crews, under bark of tamarind tree, several egg sacs, geckos, centipedes, tenebrionids, SCC04_072, 1 imm. (EME sel_527); road from Creque Dam to Mahogany Road, intersecting Mt. Victory Camp, 17°44'27.1"N, 64°51'25.4"W, ~21 m, 14.VI.2006, S. Crews, C. Niebuhr, under bark of *Bursera*,SCC06_057, 1♀ (CAS sel_501); Sprat Hall Beach, Rte 63, Fredericksted, 17°44'09.8"N, 64°53'24.0"W, sea level, 14.VI.2006, S. Crews, C. Niebuhr, under bark of sea grape on beach, SCC06_056, 4 imm. (EME sel_497-500); Sprat Hall Hill, west shore road, 1st right after old sub tracking station, 17°44'38.4"N, 64°53'22.3"W, ~54 m, 14.VI.2006, S. Crews, C. Niebuhr, on landscape plants around house, SCC06_055, 3 imm. (CAS sel_494-496); summer 1941, H.A. Beatty, 1♂ (MCZ); summer 1941, H.A. Beatty, 1♀ (AMNH). **St. John:** Lind Point Trail, sunny to very dry area, 22.III.1970, H.L., F., Levi, 1♀ (AMNH); John's Folly, 20.III.1970, H.L, F. Levi, 1♂, 1♀ (AMNH); 18-29.III.1970, H.L, F. Levi, among coral and debris under sea grape, 1♀, 1 imm. (AMNH); USVINP, Bordeaux Ridge Road, 18°20.125'N, 64°43.672'W, ~377 m, 17.XI.2004, S. Crews, under bark, SCC04_071, 1 ♂, 3 imm. (CAS sel_140-142, 157); Bordeaux Ridge Road, 27.III.1970, L., H.L. Levi, vegetation in forest, 1♀ (MCZ); USVINP, Cinnamon Bay Loop Trail, 18°21.226'N, 64°45.259'W, ~50 m, 16-17.XI.2004, S. Crews, under bark of bay trees, SCC04_070, 1♂, 1 imm. (EME sel_126-127); USVINP, Leinster Bay Trail, 18°21.825'N, 64°43.743'W, ~9 m, 16.XI.2004, S. Crews, under bark and rocks, SCC04_069, 3♂, 1 imm. (CAS sel_136-139); Leinster Bay, 13.VI.1986, W.B. Muchmore, under rock, 1♀ (FSU SJ80-1); Reef Bay, 12.VI.1979, W.B. Muchmore, under bark, 1♀ (FSU WM5450); Lameshur Bay, 26.V.1979, W.B. Muchmore, under bark, 1♂ (FSU WM5443). **St. Thomas:** Benner Hill, above armory, 18°19.533'N, 64°51.703'W, ~82 m, 19.XI.2004, S. Crews, R. Platenberg, under bark and rocks, SCC04_074, 4 imm. (EME sel_123, 132-134); Estate Nazareth, East End, Dolphin House, 18°19.128'N, 64°51.567'W, ~82 m, 19.XI.2004, S. Crews, R. Platenberg, SCC04_076, 5 imm. (CAS sel_143-147); Perseverance, Perseverance Bay Trail, 18°21.463'N, 64°59.753'W, 22.X.2004, S. Crews. R. Platenberg, under bark of *Meliococcus bijugatus*, SCC04_050, 1 imm. (EME sel_076); Magen's Bay Trail, 18°21.332'N, 64°55.220'W, ~150 m, 19.XI.2004, S. Crews, R. Platenberg, on palms and under bark, one eating termite, SCC04_073, 1♂, 5 imms. (CAS sel_124-125, 128-130, 164) Magen's Bay, top of trail, 18°21.350'N, 64°55.231'W, 22.X.2004, S. Crews, R. Platenberg, under rock, SCC04_052, 1 imm. (EME sel_077); St. Peter House, 24.X.2004, under plastic tub, R. Platenberg, SCC04_075, 1♀ (CAS sel_131).

#### Diagnosis.

Females can be separated from other species by the oval spermathecae arising from coiled ducts (Figs 89–90). Males can be separated from other species by the very short embolus located posteromedially, and the MA which is almost as long as the embolus (Figs 91–92).

#### Remarks.

Despite the large distribution this species occupies, little to no variation was noticed in size or in genitalic morphology.

#### Description.

*Male from Christiansted, St. Croix, Muma's ‘allotype'*: Color: carapace (‘allotype') orange-brown, no markings (recent) yellow-brown, with dark markings medially, laterally and mediolaterally; sternum (“allotype") light orange-yellow, darker around border, (recent) dusky yellow, darker around border; chelicerae (“allotype") brown, darker laterally (recent) light medial part same color as carapace, surrounded by brown area; maxillae (“allotype") light orange-brown, lightening distally (recent) dusky yellow; labium (“allotype") orange-brown (recent) brown lightening distally; abdomen dorsally (“allotype") grey to cream with some dark flecks, dark laterocaudal festoon present (recent) grey-yellow with darker grey median lanceolate stripe which becomes 2 slightly curved lines ¾ length to the caudal margin, one thicker lateral line below these, entire abdomen with lots of dark flecks, five light tufts of setae caudally that stand erect in life over dark background, forming a more pointed festoon area; abdomen ventrally (“allotype") light yellow (recent) dusky yellow-grey; legs (“allotype") orange-brown with annulations distinct (recent) legs dusky yellow with dark brown-black annulations, that are sometimes not distinct and the ‘stripe' on anteroventral face, looks as though it is made up of multiple spots rather than a solid stripe. Carapace:0.93 times longer than broad. Eyes:AER slightly recurved; PER recurved; PME larger than AME, PLE largest, ALE smallest; eye diameters, AME 0.15, ALE 0.05, PME 0.23, PLE 0.33; interdistances AME-PME 0.03, PME-ALE 0.23, ALE-PLE 0.23. PME-PME 1.00. ALE-ALE 1.73; ocular quadrangle AME-AME 0.30, PLE-PLE 1.63; clypeus 0.05 high. Mouthparts:chelicerae with a few stout setae medially and anteriorly; promargin with 3 teeth, retromargin with 2 teeth. Sternum:as long as broad, posteriorly indented. Legs:leg I only slightly shorter than II and III; leg formula 23,1=4; scopulae present on distal end of all 4 tarsi; tarsi I-IV with strong claw tufts; pr claw per foot slightly toothed; spination: leg I, Fm pr 1–1–1, d 1–1–1, rl 1–1–1; Ti pr 1–0–1, rl 1–0–1, d 1–1–0, v 2–2–2; Mt pr 1–1–0, rl 1–1–0, v 2–2; leg II, Fm pr 1–1–1, d 1–1–1, rl 1–1–1; Ti pr 1–0–1, d 1–1–0, rl 1–0–1, v 2–2–2; Mt pr 1–1–0, rl 1–0–0, v 2–2; leg III, Fm pr 1–1–1, d 1–1–1, rl 1–1–1; Ti pr 1–0–1, rl 1–0–1, d 1–0–0, v 2–2; Mt pr 1–1–0, rl 1–0–0, v 2–2; leg IV, Fm pr 1–1–1, d 1–1–1, rl 1–1–1; Ti pr 1–0–1, rl 1–0–1, d 1–0–0, v 2–2; Mt pr 1–1–0, rl 1–1–0, v 2–1–1. Abdomen: with terminal setal tufts. Pedipalp:Fm, d 0–1–4; cymbium oval in ventral view, slightly angled posterolaterally; conductor large angulate structure arising mediolaterally, curving around into large hook, pointed distally; embolus short, slightly sinuate, tapering from base, directed anteriorly, starting at 7 o'clock, terminating at 9 o'clock; MA arising at 5 o'clock, directed distally, narrow, long base curving to small single hook; RTA with one apophysis, small, wide, quadrate with thornlike point along the anterior margin; tibial apophyses barely reaching cymbium in ventral view (Figs 91–92). *Dimensions:* Total length 6.50. Carapace length 3.28, width 3.53. Abdomen length 3.23, width 2.25. Pedipalp: Fm 0.85, Pt 0.25, Ti 0.30, Ta 0.75, total 2.15. Leg I: Fm 5.00, Pt 1.60, Ti 4.75, Mt 4.75, Ta 1.90, total 18.00. Leg II: Fm 5.50, Pt 1.75, Ti 5.00, Mt 5.00, Ta 2.00, total 19.25. Leg III: Fm 5.50, Pt 1.50, Ti 4.60, Mt 4.95, Ta 2.00, total 18.55. Leg IV: Fm 5.50, Pt 1.50, Ti 4.50, Mt 4.75, Ta 1.75, total 18.00.

*Holotype female:* color:carapace (holotype) orange-brown, no markings (recent) yellow-brown, with dark markings medially, laterally and mediolaterally; sternum (holotype) orange-brown, darker around border (recent) yellow, darker around border; chelicerae (holotype) brown, darker laterally (recent) light medial part same color as carapace, surrounded by darker brown area; maxillae (holotype) light orange-brown, lightening distally (recent) dusky yellow; labium (holotype) orange-brown (recent) brown, lightening distally; abdomen dorsally (holotype) grey to cream with some dark flecks, dark laterocaudal festoon present (recent) grey-yellow with darker grey median lanceolate stripe that becomes 2 slightly curved lines ¾ length toward the posterior end of the abdomen, one thicker lateral line below these, entire abdomen with lots of dark flecks, five light tufts of setae caudally that stand erect in life over dark background, forming a more pointed festoon area; ventrally (type) light yellow (recent) dusky yellow-grey; legs (type) orange-brown with annulations distinct (recent) dusky yellow with dark brown to black annulations. Carapace: 0.91 times longer than broad. Eyes:AER nearly straight; PER recurved; PME larger than AME, PLE largest, ALE smallest; eye diameters, AME 0.15, ALE 0.10, PME 0.26, PLE 0.33; interdistances AME-PME 0.03, PME-ALE 0.30, ALE-PLE 0.30. PME-PME 1.00. ALE-ALE 1.95; ocular quadrangle AME-AME 0.30, PLE-PLE 1.88; clypeus 0.05 high. Mouthparts:chelicerae with a few stout setae medially and anteriorly; maxillae longer than broad, with tuft of conspicuous setae distally; labium distally rounded. Sternum:1.17 times longer than broad, posteriorly indented. Legs:leg I only slightly shorter than legs II, III and IV; leg formula 2134; scopulae present on tarsi of all legs and metatarsi of legs I and II; tarsi I-IV with strong claw tufts; pr claw per foot slightly toothed; spination: leg I, Fm pr 1–1–0, d 1–1–1, rl 0–0–1; Ti d 0, v 2–2–2; Mt v 2–2; leg II, Fm pr 1–1–0, d 1–1–1, rl 0–0–1; Ti v 2–2–2; Mt v 2–2; leg III, Fm pr 1–1–0, d 1–1–1, rl 0–0–1; Ti v 2–2–0; Mt v 2–1; leg IV, Fm pr 1–0–0 (L), 1–1–0 (R), d 1–1–1, rl 0–0–1; Ti v 2–1; Mt v 1–1. Abdomen:with terminal setal tufts. Pedipalp:claw with ca. 6 teeth. Epigyne:median field depressed, lateral lobes meet at posterior margin, genital openings located just anterior to lobes, epigynal pockets absent; internally, openings lead to coiled ducts and oval spermathecae located laterally, fertilization ducts located medially, directed anteriorly, posterodorsal fold very small, barely covering any part of internal ducts (Figs 89–90). *Dimensions:* Total length 7.75. Carapace length 3.15, width 3.45. Sternum length 1.75, width 1.50. Abdomen length 4.60, width 3.35. Pedipalp: Fm 0.80, Pt 0.25, Ti 0.50, Ta 0.75, total 2.30. Leg I: Fm 3.70, Pt 1.45, Ti 3.50, Mt 2.75, Ta 1.00, total 12.40. Leg II: Fm 4.00, Pt 1.40, Ti 3.75, Mt 2.75, Ta 1.00, total 12.90. Leg III: Fm 4.00, Pt 1.00, Ti 3.45, Mt 2.85, ta 1.00, total 12.30. Leg IV: Fm 4.20, Pt 0.90, Ti 3.00, Mt 2.60, Ta 1.00, total 11.70.

#### Natural history.

Found under bark (Figs 185–186), rocks and debris, and in between the leaves of tropical plants, from sea level to higher elevations, in dry to very wet areas. In the Greater Antilles it is often found in the same locations as *Selenops insularis*. The female is unusual in that she does not guard the egg sac, but rather makes several at once, and often leaves them anywhere, in a haphazard fashion, rather than hiding them beneath something (Fig. 186). This species has been seen feeding on termites and has been observed being fed upon by *Anolis*. More details of this species' natural history can be found in Crews et al. (2008).

#### Distribution.

Known from Eastern Hispaniola, Isla Mona, Puerto Rico, Culebra, Vieques, all of the Virgin Islands including many small cays, St. Kitts and Nevis, as well Great Inagua in the Bahamas (Map 9).

### Selenops aissus

Walckenaer, 1837

http://species-id.net/wiki/Selenops_aissus

**Map 10.**
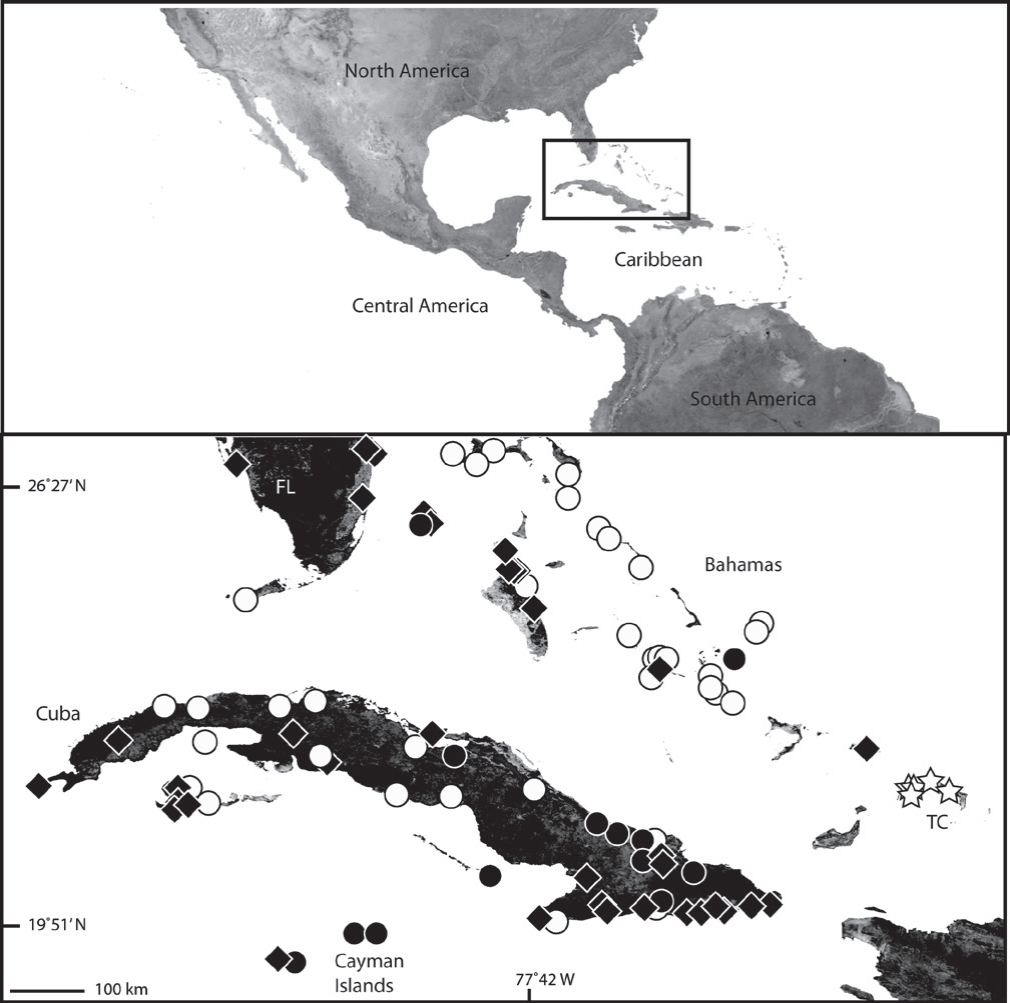
Map of southern Florida, the Bahamas, Cuba, Cayman Islands and Turks and Caicos Islands (inset) showing the distribution of four species of *Selenops*. *Selenops aissus* Walckenaer (white circles), *Selenops simius* Muma (black circles), *Selenops submaculosus* Bryant (black diamonds), *Selenops baweka* sp. n. (white stars). (FL=Florida, TC=Turks and Caicos Islands).

**Figures 93–100.**
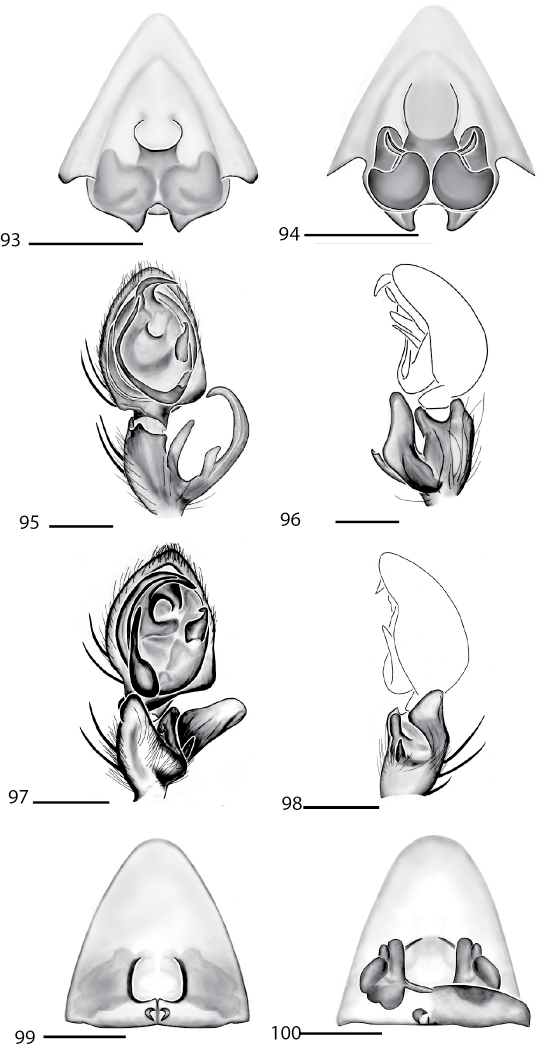
Copulatory organs of *Selenops aissus* Walckenaer female from Ensenada de Cochina, Cuba (MCZ) **93–94** male from Spanish Wells, Eleuthera, Bahamas (USNM) **95–96** *Selenops candidus* Muma male from Jamaica (MCZ) **97–98** female from Jamaica (holotype of *Selenops lunatus)* (MCZ) **99–100,** **93, 99** epigyne, ventral view **94, 100** spermathecae, dorsal view **95, 97** left pedipalp, ventral view **96, 98** left pedipalp, retrolateral view. Scale bar = 0.50 mm.

Figs 93–96 202 Map 10

Selenops aissus Walckenaer 1837: 547 (♀ lost, not examined).
Selenops aissus :Keyserling 1884: 683, pl. 21, Fig. 30 (♀).
Selenops confusus Petrunkevitch 1925: 134 (♀) (believed Keyserling's ♀ misidentified).
Selenops aissus :Petrunkevitch 1925: 134, Figs 50–52 (♀) (misidentified).
Selenops timidus Bryant 1940: 407, pl. 13, Fig. 183 (♀).
Selenops aissus :Muma 1953: 31, Fig. 55 (♀).
Selenops vexillarius Muma 1953: 30, Figs 53–54 (♂). Synonymized with *Selenops aissus* by Alayón-García (2005).
Selenops aissus :Alayón-García 2005: 26, Figs 21–24 (♀, ♂).

#### Type material.

Female holotype: Trinidad, Walckenaer (MNHN – likely an erroneous locality, and type is lost; see ‘Remarks' below), not examined. Neotype female (designated here) from Bahamas, Abaco Island, Ralph's Chimney, off Queen's (Abaco) Highway, 26°14'58.2"N, 77°11'25.4"W, ~6 m, 14.V.2006, S. Crews, under bark of dead tree, SCC06_006, 1♀ (EME sel_315).

#### Other material examined.

BAHAMAS: Abaco: Abaco National Park, 26°03'44.0"N, 77°12'46.2"W, ~13 m, 14.V.2006, S. Crews, under bark, limestone rocks, SCC06_005, 1 imm. (EME sel_313). **Andros:** Fresh Creek, 23.IV.1953, L. Giovannolli, 1♂, 2 imm. (AMNH). **Eleuthera:** Spanish Wells (holotype male and allotype female of *Selenops vexillarius* USNM); 1965, A. Spielman, 1♂ (AMNH); New Portsmouth, 28.III.1953, G. Rabb, several (AMNH). **Grand Bahama:** 29.VI.1951, A.F. Carr, Jr., 1♀ (FSU); West End Settlement, 5.VI.1949, 2♀ (FSU); Freeport, V.1965, A. Spielman, 1♀ (MCZ). **Great Exuma:** George Town, Regatta Point, 23°30'24.7"N, 75°45'58.0"W, sea level, 18.V.2006, S. Crews, under fallen coconuts, and under bark, SCC06_009, 1♀, 5 imm. (EME sel_319-324); cays west of Green Turtle cut, 1973, R. Wetzler, 1♂ (MCZ); near Great Exuma, Wetzler, 1♂ (AMNH); Stanyard Cay, 13-I.1953, E. Hayden, 1♀ (AMNH). **Long Island:** Clarence Town, 14.III.1953, Hayden, Rabb, Giovannoli, 1♀ (AMNH); Deadman's Cay, 11.III.1953, E. Hayden, 1♀ (AMNH); Simons, 19.VII.1936, 1♀ (MCZ). **San Salvador:** Cockburn Town, 18.III.1953, Rabb, Giovannoli, 1♂ (AMNH); Gerace Field Station, trails behind field station, vic. 24°06.9'N, 74°27.8'W, ~3–25 m, 19.V.2006, S. Crews, under rocks and bark, SCC06_012, 4♀, 7 imm. (CAS sel_333-343). **Stocking Island:** near Great Exuma, 23°32'08.9"N, 75°46'29.6'W, sea level-~16 m, 18.V.2006, under bark of *Casuarina* and *Bursera*, S. Crews, SCC06_010, 2♀, 5 imm. (EME sel_325-331). **CUBA: Cabanas:** p dR, 5-8.IX.1913, 1♀ (AMNH). **Cienfuegos Bay:** Cays Ocampo, agave stump, 11.VII.1947, W.L. Nutting, 1♀ (MCZ); Ensenada de Cochina, 2.III.1917, Barbour, Brooks and Warner (holotype of *Selenops timidus* MCZ). **UNITED STATES: Alabama:** Montgomery Co., Montgomery, winter, 1946, A.F. Archer, 1♀; **Florida:** Key West, 1♀ (USNM).

#### Diagnosis.

Females can be separated from other species by the posterior margin of the epigynal plate which extends below the epigastric furrow and forms two points (Figs 93–94). Males can be separated from other species by the very large RTA which extends laterally, and curves back toward the bulb. The longest apophysis also has a small process (Figs 95–96).

#### Remarks.

Walckenaer (1837) described this species from Trinidad. The type was lost. This species is not found in Trinidad. Either Walckenaer (1837) described another species, or this type was from another locality where *Selenops aissus* actually occurs. The drawing of *Selenops aissus* from Muma (1953) is clearly the same as that provided by Keyserling (1884). Petrunkevitch (1925) doubted that Keyserling's (1884) *Selenops aissus* was the same as the type described by Walckenaer (1837), and described *Selenops confusus*. Petrunkevitch (1925) based this on leg lengths, which have problems associated with them, including intraspecific variation. Petrunkevitch (1925) also illustrated two species of ‘*Selenops aissus*' from Panamá, but the illustrations are clearly those of *Selenops mexicanus*. Alayón-García (2005) synonymized the male of *Selenops vexillarius* with *Selenops aissus*. Although the type was not figured by Walckenaer (1837), Muma (1953) believed this specimen to be the same as the one he was describing as *Selenops aissus*. This leads me to two conclusions, both with the same outcome: (1) Walckenaer (1837) had incorrect locality data for the specimen he described as *Selenops aissus* from Trinidad, or (2) the specimen Walckenaer (1837) described was introduced on materials shipped from somewhere within the range of the species. In either case, the name is valid, but the locality data is not. Thus, no changes have been made to the name. Yet, in order to stabilize the species name, the female neotype collected from the Bahamas has been designated.

The specimen from Montgomery, Alabama is regarded as a likely importation, and it is unlikely this species is established there, especially in the winter.

There is some variation in size. In particular, the specimens from Stocking Island are larger than specimens from elsewhere. For example, one specimen from Stocking Island (sel_325) has a body length of 15.20, while one from Great Exuma (sel_324) has a length of 8.20.There is variation in the morphology of the epigynum. In some species it is wider and shorter vs. others where it is narrower and longer. There is also variation in the markings on the abdomen. In some specimens there is only a festoon present. Other specimens have a festoon and faint pairs of spots anteromedially, some with festoon, spots and chevrons, caudally. There were not enough male specimens to assess variation.

#### Description.

*Male (holotype of S. vexillarius)*:Color:carapace dark green-grey-brown; sternum grey-green; chelicerae red-brown, darker laterally; abdomen dorsally grey-brown, no markings visible; legs orange-brown, no visible markings. Carapace:0.90 times longer than broad. Eyes:AER slightly recurved; PER recurved; PME larger than AME, PLE largest, ALE smallest; eye diameters, AME 0.20, ALE 0.06, PME 0.25, PLE 0.38; interdistances AME-PME 0.03, PME-ALE 0.05, ALE-PLE 0.33. PME-PME 1.10. ALE-ALE 1.78; ocular quadrangle AME-AME 0.40, PLE-PLE 2.25. Legs:leg formula 2314 (Muma, 1953); leg II longest. Mouthparts:chelicerae with stout setae medially and anteriorly; maxillae longer than broad, with tuft of conspicuous setae distally; labium distally rounded. Abdomen:without terminal setal tufts. Pedipalp: Fm,spination, d 1-1-2; cymbium oval in ventral view, slightly angled posterolaterally; conductor large, located anterolaterally, pointed laterally, not extending beyond cymbium edge, curving around from lateral margin, tapering to hook, small circular space where conductor attached to bulb; embolus long, slender, curved, tapering, beginning at 4 o'clock, terminating at 11 o'clock; MA originating at 4 o'clock, directed ventrally, on narrow, long base, tapering distally, and curved into a small single hook; two tibial apophyses, ventral process smaller, curves outward ventrally and back toward bulb, uniform in shape,slightly pointed at tip; lateral process very long, nearly as long as the cymbium, but arises rather low on the palpal tibia it barely reaches the cymbium, curves outward, then back toward bulb, with second small process located near base; tibial apophyses barely reaching cymbium in ventral view (Figs 95–96). *Dimensions:* Total length 7.25. Carapace length 3.68, width 4.10. Abdomen length 3.58, width 2.60. Pedipalp: Fm 1.50, Pt 0.90, Ti 1.00, Ta 1.40, total 4.80. Leg I: Fm 4.50, Pt 1.20, Ti absent, Mt missing, Ta missing, total missing. Leg II: Fm 4.50, Pt 2.50, Ti 5.00, Mt 3.20, Ta missing, total missing. Leg III: Fm 4.50, Pt 2.40, Ti 4.90, Mt 3.20, Ta 2.00, total 17.00. Leg IV: Fm 3.90, Pt 1.60, Ti missing, Mt missing, Ta missing, total missing.

*Female (holotype of S. timidus)*: Color:carapace (holotype) orange-brown, white setae present (neotype) red-brown to yellow-brown, tan, sometimes with dusky markings distally and darker laterally, white setae present; sternum light yellow to light brown; chelicerae (holotype) red-brown (neotype) red-brown darker laterally; maxillae light yellow to light brown, lightening distally; labium light yellow to light brown; abdomen dorsally (holotype) yellowish anteriorly, brown elsewhere, festoon no longer visible, dark spots barely visible (neotype) light grey; ventrally grey, no markings visible; legs (holotype) orange-brown, no visible markings (neotype) light yellow with faint annulations, legs darkening from the tibiae to tarsi. Carapace: 0.85 times longer than broad; fovea longitudinal, broad, very shallow. Eyes:AER nearly straight; PER recurved; PME larger than AME, PLE largest, ALE smallest; eye diameters, AME 0.28, ALE 0.08, PME 0.38, PLE 0.48; interdistances AME-PME 0.10, PME-ALE 0.10, ALE-PLE 0.55. PME-PME 1.50. ALE-ALE 2.43; ocular quadrangle AME-AME 0.55, PLE-PLE 2.60; clypeus 0.14 high. Mouthparts:chelicerae with a few scattered setae medially and anteriorly; maxillae longer than broad, with tuft of conspicuous setae distally; labium distally rounded. Sternum:1.08 times longer than broad, posteriorly indented. Legs:leg I much shorter than legs II, III and IV; leg formula 2341; scopulae present on all four tarsi, on metatarsi and distally on tibiae of legs I and II; tarsi I-IV with strong claw tufts; pr claw per foot slightly toothed; spination: leg I, Fm pr 2–2–0, d 1–1–1, rl 1–0–1; Ti v 2–2–2; Mt v 2–2; leg II, Fm pr 0–2–0, d 1–1–1, rl 1–0–1; Ti v 2–2–2; Mt v 2–2; leg III, Fm pr 1–1–0, d 1–1–1, rl 1–1–1; Ti v 2–2–0; Mt v 2–2; leg IV, Fm pr 1–1–0, d 1–1–1, rl 0–0–1; Ti v 2–2–0; Mt v 2–2. Abdomen:without terminal setal tufts. Pedipalp:claw with 8 teeth. Epigyne: epigynal plate triangular, extending posteriorly past epigastric furrow, into two points, median area with u-shape, genital openings located along posterior margin of this, epigynal pockets absent; internally, short ducts extend to large roundish spermathecae, fertilization ducts located anteriorly and directed anterolaterally, posterodorsal fold absent (Figs 93–94). *Dimensions:* Total length 10.38. Carapace length 4.58, width 5.40. Sternum length 2.60, width 2.40. Abdomen length 5.80, width 4.50. Pedipalp: Fm 1.00, Pt 0.50, Ti 1.00, Ta 1.75, total 4.25. Leg I: Fm 4.50, Pt 1.50, Ti 3.50, Mt 3.00, Ta 1.60, total 14.10. Leg II: Fm 6.00, Pt 2.00, Ti 5.00, Mt 3.75, Ta 1.60, total 18.35. Leg III: Fm 5.40, Pt 1.75, Ti 4.65, Mt 4.00, Ta 1.75, total 17.55. Leg IV: Fm 5.00, Pt 1.4,0 Ti 4.00, Mt 3.50, Ta 1.60, total 15.50.

#### Natural history.

Found under the bark of several types of trees, in agave, and under rocks (Fig. 202).

#### Distribution.

Found in the Florida Keys, the Bahamian islands of Abaco, Andros, Eleuthera, Grand Bahama, Great Exuma, Long Island, Staniel Cay, Stocking Island and San Salvador, as well as on the Greater Antillean island of Cuba (Map 10).

### Selenops candidus

Muma, 1953

http://species-id.net/wiki/Selenops_candidus

Figs 97–100 Map 11

Selenops aissus Petrunkevitch 1925: 134, Figs 53–54 (♀, misidentified).
Selenops candidus Muma 1953: 29, Figs 51–52 (♂, examined).
Selenops lunatus Muma 1953: 31, Fig. 56 (♀, examined) syn. n.*Selenops candidus*: Alayón-García 2003: 118, Figs 3–6 (♂, ♀).
Selenops lunatus : Alayón-García 2003: 118, Figs 1–2 (♀).

#### Type material.

Male holotype: Jamaica, 1935, L. Perkins (MCZ, examined).

#### Notes.

The female holotype of *Selenops lunatus* Muma, 1953 retained in the MCZ (examined) is in every way identical to the females collected with the males of *Selenops candidus* Muma 1953, and thus both species names are to be synonymised.

#### Other material examined.

JAMAICA: 1934, L. Perkins (holotype of *Selenops lunatus*) (MCZ);**Clarendon Parish:** on gravel road off road to Lluidasvale, 18°07'50.8"N, 77°10'05.0"W, ~454 m, 31.V.2006, S. Crews, E. Morrison, L. Wright, under bark of *Bursera*, 1♂, 1 p♀ (IJNHM. sel_363-364); **St. Andrew Parish:** Castleton Botanic Garden, 18°10'20.3"N, 76°49'27.6"W, ~157 m, 27.V.2006, S. Crews, I. Wilmot, under bark in garden, 1 p♂, 1 imm. (EME. sel_350-351); Hermitage Dam Road, 2.6 km from junction with Stony Hill, 18°04'25.4"N, 76°47'01.3"W ~368 m, 5.VI.2006, S. Crews, I. Wilmot, under bark of *Bursera*, 1 ♀ (IJNHM sel_385). **St. Ann Parish:** North Coast Highway between Discovery Bay and Rio Bueno, 18°28'31.3", N 77°25'49.0" W, ~25 m, 28.V.2006, S. Crews, I. Wilmot, under bark on limestone ridge, 2 ♂, 2 imm. (IJNHM. sel_357-360). **St. Mary Parish:** near Mango Valley at football field, 1.6 km off of North Coast Hwy., 18°24'23.4"N, 77°02'37.5"W, ~156 m, 28.V.2006, S. Crews, I. Wilmot, under bark, 5 imm. (EME sel_352-356); Strawberry Fields near Robin's Bay and Green Castle, campground, 22.III.1972, H. & F. Levi, 1 ♀ (MCZ). **Westmoreland Parish:** near New Hope, on road to Savanna-la-Mar, 18°14'55.4"N, 78°14'41.0"W, ~40 m, 29.V.2006, S. Crews & I. Wilmot, under fence post (no voucher, spiderling, used in molecular analysis, sel_362). **Manchester Parish:** Mandeville, 14.II.1946, B. Heineman, 1 ♀ (AMNH).

#### Diagnosis.

Males can be separated from all other species by the palpus, which is similar to that of other Jamaican species in having a two-pronged embolus and a tibial apophysis with 3 branches instead of two, however, the dorsal branch of the RTA is wider distally, and the base of the MA is more quadrangular (Figs 97–98). Females can be distinguished from other species by the quadrangular to round median field (Figs 99–100).

#### Remarks.

The female of this species was described by Petrunkevitch (1925) as *Selenops aissus* based on a description only, and without viewing Walckenaer's (1837) specimen. Muma (1953) described the female of *Selenops lunatus*, designating a new type. Muma also described the male as *Selenops candidus* and noted that, based on their overall appearance, *Selenops candidus* and *Selenops lunatus* might be the same species (these specimens were apparently not collected together, and the precise collection localities are unknown). Alayón-García (2003) re-described Petrunkevitch's (1925) specimens of *Selenops aissus* from the Peabody Museum as two different species, one as *Selenops lunatus* and one as the female of *Selenops candidus*. Muma (1953) noted that the two specimens designated as *Selenops aissus* by Petrunkevitch (1925) may demonstrate genitalic variation. In my extensive collecting, several males identified as *Selenops candidus* were collected, and one female from a second locality that was identified as *Selenops lunatus*. However, molecular analyses (Crews and Gillespie 2010) indicate that the female specimen described as *Selenops lunatus* is the same species as those specimens described as *Selenops candidus*. Therefore, it would seem the female previously described as *Selenops lunatus* by Alayón-García (2003) is a variant of *Selenops candidus*. Therefore, this species' name has been synonymised. Despite that Petrunkevitch's specimens were viewed recently (Alayón-García 2003), and should be in the PM, they cannot be located now. Female specimens show genitalic variation in the shape of the median field. It can be very square or roundish, and wide or narrow.

#### Description.

*Holotype male:* Color: Carapace (holotype) orange and transparent (recent) brown-yellow with darker marks laterally and lateromedially, white setae prominent around the lateral margins of the carpace and the eyes; sternum pale yellow, darker around border; chelicerae (holotype) dark red-brown (recent) light reddish-brown with indistinct dusky marks on the anterior faces; maxillae yellow, lightening distally; labium dusky yellow, lightening distally; abdomen (holotype) orange-tan with the two pairs of dark median spots and festoon visible (recent) tan-grey, lighter anteromedially, darkening laterally and caudally, with 2 median dark spots, and festoon pattern visible; ventrally light yellow; legs (holotype) orange-yellow with leg bands very indistinct (recent) brown-yellow darkening distally, especially in legs III and IV, with leg bands indistinct. Carapace: 0.83 times longer than broad. Eyes: AER nearly straight; PER recurved; AME slightly larger than PME, PLE largest, ALE smallest; eye diameters, AME 0.35, ALE 0.10, PME 0.25, PLE 0.38; interdistances AME-PME 0.10, PME-ALE 0.13, ALE-PLE 0.38. PME-PME 1.36. ALE-ALE 2.15; ocular quadrangle AME-AME 0.55, PLE-PLE 2.20; clypeus 0.09 high. Mouthparts: Chelicerae with stout setae medially and anteriorly; maxillae longer than broad, with tuft of conspicuous setae distally; labium distally rounded. Sternum: 1.38 times longer than broad, posteriorly indented. Legs: Leg I only slightly shorter than II and III; leg formula 2314; scopulae present on distal end of all 4 tarsi; tarsi I-IV with strong claw tufts; pr claw per foot slightly toothed; spination: leg I, Fm pr 1–1–1, d 1–1–1, rl 1–1–1; Ti d 1–1–0, pr 1–1–0, rl 1–1–0, v 2–2–2–2; Mt pr 1–0–0, v 2–2–0, rl 1–1–0; leg II, Fm pr 1–1–1, d 1–1–1, rl 1–1–1; Ti pr 1–1–0, d 1–1–0, rl 1–1–0, v 2–2–2–2; Mt pr 1–1–0, v 2–2, rl 1–0–0; leg III, Fm pr 1–1–1, d 1–1–1, rl 1–1–1; Ti pr 1–1–0, d 0, rl 1–1–0, v 2–2–2; Mt pr 1–0–0, rl 1–0–0, v 2–2; leg IV, Fm pr 1–1–0, d 1–1–1, rl 1–1–1; Ti pr 1–0–1, v 2–2–0, rl 1–0–1; Mt pr 1–1–0, rl 1–0–0, v 2–2–0. Abdomen: Without terminal setal tufts. Pedipalp: Femur, spination d 0–1–3; cymbium oval in ventral view, truncate posterolaterally; conductor large, arising from short, slightly curved stalk near distal portion of bulb, pointed laterally toward 2 o'clock position, and not extending beyond the edge of cymbium, curving around retrolateral side and reconnecting to bulb near center, forming circular space between the connections; embolus two-pronged, one prong long, slender, curving around edge of cymbium ending at 1 o'clock, more ventral prong arises from large rounded base, tapering abruptly, beginning at 6 o'clock and terminating at 9 o'clock; MA located between 3 and 4 o'clock, directed distally, with stout, squarish base that tapers abruptly to slender, curved single hook; tibial apophyses extend at least ¼ way up length of cymbium in ventral view; three tibial apophyses; ventral apophysis slender, widening, flattened at tip; MA is very small, pointed, conical structure; dorsal tibial apophysis longest, curving dorsally, then ventrally, widening toward apex, truncate (Figs 97–98). *Dimensions:* Total length 8.73. Carapace length 4.50, width 5.40. Sternum length 2.75, width 2.00. Abdomen length 4.23, width 3.60. Pedipalp: Fm 1.50, Pt 0.50, Ti 0.75, Ta 1.25, total 3.90. Leg I: Fm 5.80, Pt 2.00, Ti 5.75, Mt 5.75, Ta 2.20, total 21.5. Leg II: Fm 7.25, Pt 2.00, Ti 6.50, Mt 6.00, Ta 2.40, total 24.15. Leg III: Fm 6.75, Pt 1.90, Ti 6.00, Mt 5.90, Ta 2, total 22.55. Leg IV: Fm 6.00, Pt 1.80, Ti 5.00, Mt 5.00, Ta 1.90, total 19.70.

*Female (holotype of S. lunatus):* Color:Carapace dark red-brown with white setae; sternum (holotype) rusty yellow (recent) orange-brown, darker around border; chelicerae (holotype) dark brown (recent) dark brown with white setae; maxillae (holotype) rusty yellow (recent) orange-brown, lightening distally; labium (holotype) rusty yellow (recent) orange-brown, lightening distally; abdomen dorsally (holotype) cream-colored, with a very slight indication of a medial stripe, festoon barely visible (recent) yellow-grey, lighter medially, greyer on sides, festoon prominent, 4 pairs of spots medially, caudal-most pair most prominent; ventrally (holotype) light yellow (recent) dusky yellow; legs (holotype) orange-brown (recent) yellow-brown, annulations visible. Carapace:0.93 times longer than broad. Eyes:AER nearly straight; PER slightly recurved; PME larger than AME, PME same as PLE, ALE smallest; eye diameters, AME 0.30, ALE 0.13, PME 0.35, PLE 0.35; interdistances AME-PME 0.08, PME-ALE 0.23, ALE-PLE 0.20. PME-PME 1.50. ALE-ALE 2.75; ocular quadrangle AME-AME 0.55, PLE-PLE 2.75; clypeus 0.08 high. Mouthparts:chelicerae with stout setae medially and anteriorly; maxillae longer than broad, with tuft of conspicuous setae distally; labium distally rounded. Sternum:1.09 times longer than broad, posteriorly indented. Legs:leg I only slightly shorter than legs II and III; leg formula 2314; scopulae present on all 4 tarsi, metatarsi and distally on tibiae I and II; tarsi I-IV with strong claw tufts; pr claw per foot slightly toothed; spination: leg I, Fm pr 1–1–1, d 1–1–1, rl 1–1–0; Ti v 2–2–2; Mt v 2–2; leg II, Fm pr 1–1–1, d 1–1–1, rl 1–1–1; Ti v 2–2–2; Mt v 2–2; leg III, Fm pr 1–1–1, d 1–1–1, rl 1–1–0; Ti v 2–2–0; Mt v 2–2; leg IV, Fm pr 1–1–0, d 1–1–1, rl 0–0–1; Ti v 2–2–0; Mt v 2–1. Abdomen:Without terminal setal tufts. Pedipalp:claw with 8 teeth. Epigyne:externally,lateral lobes widely separated medially, meeting caudally, forming quadrangular to rounded median field, genital openings lateral, with epigynal pockets present; left and right halves of internal copulatory organs distantly positioned on either side of the median field, internal copulatory organs large and rather amorphous, posterodorsal fold present, covers ¼ of internal copulatory organs (Figs 99–100). *Dimensions:* Total length 14.58. Carapace length 5.25, width 5.65. Sternum length 3.00, width 2.75. Abdomen length 9.33, width 7.35. Pedipalp: Fm 1.75, Pt 0.90, Ti 1.00, Ta 1.90, total 5.55. Leg I: Fm 6.00, Pt 2.50, Ti 5.00, Mt 4.75, Ta 1.80, total 20.05. Leg II: Fm 6.75, Pt 2.75, Ti 5.50, Mt 4.75, Ta 1.75, total 21.50. Leg III: Fm 6.75, Pt 2.75, Ti 5.65, Mt 4.75, Ta 1.75, total 21.65. Leg IV: Fm 6.00, Pt 1.75, Ti 4.75, Mt 4.75, Ta 1.60, total 18.85.

#### Natural history.

This species has been collected in dry coastal limestone forests, as well as dry inland forests, from sea level to 500 m. It has been taken on *Pandanus*, under the loose bark of several trees including Pimento, *Bursera*, and *Eucalyptus*, on fence posts, and on bananas. Egg sacs are single, flat, white discs attached under bark and guarded by the female.

#### Distribution.

**Map 11.**
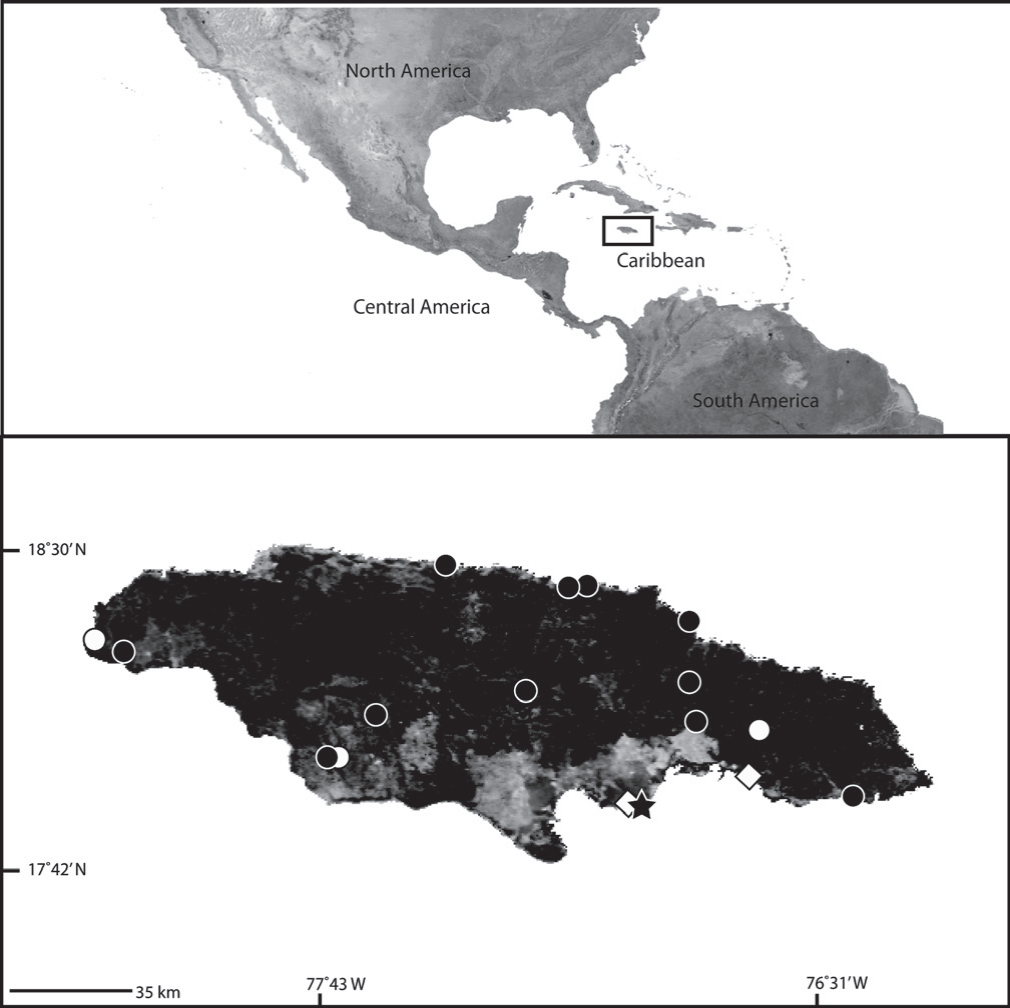
Map of Jamaica (inset) showing the distribution of endemic Selenopidae found on the island. *Selenops candidus* Muma (black circles), *Selenops petrunkevitchi* Alayón-García (white circles), *Selenops wilmotorum* sp. n. (white diamonds), *Selenops wilsoni* sp. n. (black star).

*Selenops candidus* is endemic to the island of Jamaica, though historically has been occasionally shipped to the United States on bananas (Map 11).

### Selenops petrunkevitchi

Alayón-García, 2003

http://species-id.net/wiki/Selenops_petrunkevitchi

**Figures 101–108.**
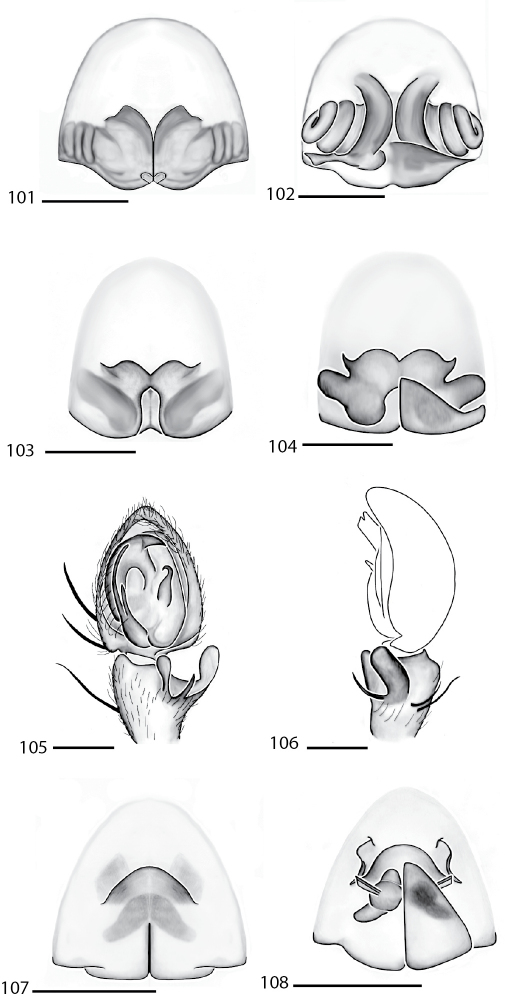
Copulatory organs of *Selenops petrunkevitchi* Alayón-García female from Whitfield Hall, Blue Mountains, Jamaica (IJNHM sel_368) **101–102** *Selenops wilmotorum* sp. n. holotype female from Hellshire Hills, St. Catherine Parish, Jamaica (EME sel_383) **103–104** male from Hellshire Hills, St. Catherine Parish, Jamaica (EME sel_384) (**105–106**), *Selenops wilsoni* sp. n. holotype female from South of Spanish Town, Hellshire Hills, St. Catherine Parish, Jamaica (CAS) **107–108,** **101, 103, 107** epigyne, ventral view **102, 104, 108** spermathecae, dorsal view **105** left pedipalp, ventral view **106** left pedipalp, retrolateral view. Scale bar = 0.25 mm (101–102), 0.50 mm (103–108).

Figs 101–102 187 Map 11

Selenops petrunkevitchi Alayón-García 2003: 120, Figs 7–8 (♀, not examined).

#### Note.

The female holotype of *Selenops petrunkevitchi* Alayón-Garcíais stated to be deposited in the PM (Alayón-García 2003), but the specimen cannot be located at PM. Nevertheless, the description and illustrations are adequate to recognize the specimens described and listed below as this species.

#### Type material.

Female holotype: Malvern, Santa Cruz Mountains, St. Elizabeth Parish, Jamaica (PM), not examined.

#### Material examined.

JAMAICA: St. Thomas Parish: Blue Mountains National Park, Whitfield Hall, 18°02'54.8"N, 76°37'03.7"W, 1.VI.2006, ~1250 m, S. Chai, I. Wilmot, S. Crews, near cloud forest, under bark of eucalypts, SCC06_029 4♀, 7 imm. (IJNHM sel_365-368, EME sel_370-373, CAS sel_369, 374-375).

#### Diagnosis.

While similar to *Selenops wilmotorum* sp. n., the shape of the anterior margin of the lateral lobes is more angular, and less sinuous, and the internal ducts are distinctly coiled (Figs 101–102). Males unknown.

#### Description.

*Female (sel_368 IJNHM):* Color:carapace red-brown, dusky medially and laterally; sternum orange-brown, darker around border; chelicerae red-brown with black y-shaped marks beginning medially; maxillae light orange-brown lightening distally; labium brown, lightening distally; abdomen dorsally cream-colored with lanceolate stripe, pointed festoon, other dusky mottling around stripe; ventrally cream-colored, dark laterally and caudally; legs light orange-brown, annulations present, mottling on anterolateral faces of femora. Carapace: 0.82 times longer than broad; fovea longitudinal, broad, very shallow. Eyes:AER nearly straight; PER slightly recurved; PME larger than AME, PLE largest, ALE smallest; eye diameters, AME 0.23, ALE 0.08, PME 0.25, PLE 0.33; interdistances AME-PME 0.08, PME-ALE 0.18, ALE-PLE 0.28. PME-PME 1.28. ALE-ALE 2.15; ocular quadrangle AME-AME 0.50, PLE-PLE 2.18; clypeus 0.05 high. Mouthparts:chelicerae with a few stout setae medially and anteriorly; maxillae longer than broad, with tuft of conspicuous setae distally; labium distally rounded. Sternum: 1.11 times longer than broad, posteriorly indented. Legs:leg I only slightly shorter than II and III; leg formula 3214 or 2413 (Alayón-García 2003); scopulae present on all 4 tarsi, metatarsi and tibiae I and II; tarsI I-IV with strong claw tufts; prolateral claw per foot slightly toothed; spination: leg I, Fm pr 0–0–1, d 1–1–1, rl 1–0–0; Ti d 0, v 2–2–2; Mt v 2–2; leg II, Fm pr 1–0–0, d 1–1–1, rl 0–0–1; Ti v 2–2–2; Mt v 2–2; leg III, Fm pr 1–0–0, d 1–1–1, rl, 0–0–1; Ti v 2–2–0; Mt v 2–2; leg IV, Fm pr 1–0–0, d 1–1–1, rl 0–0–1; Ti v 1–1; Mt v 2–1. Abdomen:with terminal setal tufts. Pedipalp:claw with 3 teeth, though looks like one or 2 teeth may have broken off. Epigyne:lateral lobes meet medially, extend anteriorly about half of the length of epigynal plate, angular along anterior margins, genital openings located just behind, epigynal pockets present; internally ducts coiled anteriorly to posteriorly, directed laterally, fertilization ducts located posteriorly,directed laterally, posterodorsal fold present, covering nearly ¼ of internal ducts (Figs 101–102). *Dimensions:* Total length 11.35. Carapace length 3.95, width 4.80. Sternum length 2.50, width 2.25. Abdomen length 7.40, width 5.10. Pedipalp: Fm 1.25, Pt 0.60, Ti 0.75, Ta 1.40, total 4.00. Leg I: Fm 5.00, Pt 1.90, Ti 4.60, Mt 3.75, Ta 1.60, total 16.85. Leg II: Fm 6.25, Pt 1.50, Ti 4.00, Mt 4.00, Ta 1.50, total 17.25. Leg III: Fm 6.70, Pt 1.90, Ti 5.00, Mt 4.00, Ta 1.50, total 19.10. Leg IV: Fm 5.60, Pt 1.65, Ti 4.00, Mt 3.75, Ta 1.50, total 16.50.

#### Natural history.

Collected under bark, including eucalypts, at both low and high elevations (Fig. 187).

#### Distribution.

Endemic to Jamaica, and appears to be widespread there (Map 11).

### Selenops wilmotorum sp. n.

urn:lsid:zoobank.org:act:CECEB994-F974-41B3-8F4B-C839DE3930A7

http://species-id.net/wiki/Selenops_wilmotorum

Figs 103–106 188 Map 11

#### Type material.

Holotype female: Hellshire Hills, St. Catherine Parish, Jamaica, ‘A2 Depression', (iguana site), 17°51'59.3"N, 76°57'54.0"W, ~275 m, 3.VI.2006, S. Crews, under bark, SCC06_031 (EME sel_383). Paratypes: Male, same data as holotype (EME sel_384).

#### Other material examined.

JAMAICA: St. Catherine Parish: same data as types, 2♀, 4 imm. (IJNHM sel_376-380, 382). **St. Thomas Parish:** near 12 mile Bull Bay on left side of road, heading east, 17°55'32.5" N, 76°38'31.0" W, ~118 m, 5.VI.2006, S. Crews, I. Wilmot, under bark, SCC06_034, 1♀, 2♂, 2 imm. (CAS sel_387-391).

#### Etymology.

This species is named in honor of the Wilmot family for their hospitality and preservation and expansion of Jamaican culture. It is to be treated as a noun in apposition.

#### Diagnosis. 

Females can be differentiated from other species by the sinuous margin and epigynal pockets (Figs 103–104) and males can be separated from other species by the conductor and thornlike medial branch of the RTA (Figs 105–106).

#### Description.

*Male:* Color:carapace light tan-yellow, Slightly darker in cephalic region, dusky around edge; sternum pale yellow; chelicerae light brown with some dusky markings; maxillae dusky yellow-brown, lightening distally; labium pale yellow-brown; abdomen dorsally grey with lanceolate stripe with chevrons anteromedially, connecting to duskier lateral margins, one dot near the end of the abdomen, festoon present; ventrally pale yellow, no markings; legs light yellowish-tan, darkening distally, annulations hardly visible. Carapace: 0.89 times longer than broad; fovea longitudinal, broad, very shallow. Eyes:AER nearly straight; PER recurved; PME larger than AME, PLE largest, ALE smallest; eye diameters, AME 0.25, ALE 0.08, PME 0.28, PLE 0.35; interdistances AME-PME 0.08, PME-ALE 0.10, ALE-PLE 0.35. PME-PME 1.25. ALE-ALE 1.95; ocular quadrangle AME-AME 0.45, PLE-PLE 2.15; clypeus 0.05 high. Mouthparts:chelicerae with a few stout setae medially and anteriorly; maxillae longer than broad, with tuft of conspicuous setae distally; labium distally rounded. Sternum:as long as broad, posteriorly indented. Legs:leg I only slightly shorter than II and III; leg formula 2314; scopulae present on distal end of all 4 tarsi; tarsi I-IV with strong claw tufts; pr claw per foot slightly toothed; spination: leg I, Fm pr 1–1–1, d 1–1–1, rl 1–1–1; Ti pr 1–0–1, d 1–1–0, rl (R) 1–1–1 (L) 1–0–1, v 2–2–2–2; Mt pr 1–0–0, v 2–2, rl 1–0–0; leg II, Fm pr 1–1–1, d 1–1–1, rl 1–1–1; Ti pr 1–0–1, d 1–1–0, rl 1–0–1, v 2–2–2–2; Mt pr 1–1–0, rl 1–0–0, v 2–2; leg III, Fm pr 1–1–1, d 1–1–1, rl 1–1–1; Ti pr 1–0–1, d 0–1–0, rl 1–0–1, v 2–2–2; Mt pr 1–1–0, rl 1–0–0, v 2–2; leg IV, Fm pr 1–1–1, d 1–1–1, rl 0–1–1; Ti pr 1–0–1, v 2–2–0, rl 1–0–1; Mt pr 1–1–0, rl 1–0–0, v 2–2–0. Abdomen:without terminal setal tufts. Pedipalp:femur, spination d 0–1–4; cymbium triangular in ventral view, angled posterolaterally; basal cymbial process absent, scopulae scattered, denser toward tip; conductor somewhat hammer shaped to T-shaped, on short straight stalk originating anteromedially, pointed laterally, not extending beyond cymbium edge, left side connecting to bulb, forming circular space between two conductor connections; embolus short, beginning at 6 o'clock, ending at 11 o'clock, basally stout and 2 branched, narrowing distally, slender and slightly curved; MA wider at base, slightly sinuous, distally curved into single hook, MA originating at 3 o'clock, directed distally; RTA extending 1/5th the length of cymbium in ventral view, with 3 processes, lateral process large, curves away from cymbium, terminally rounded, middle apophysis very small, pointed, ventral apophysis curves ventrally then dorsally, widening,rounded distally (Figs 105–106). *Dimensions:* Total length 8.55. Carapace length 4.00, width 4.50. Sternum length 1.65, width 1.65. Abdomen length 4.55, width 3.53. Pedipalp: Fm 0.90, Pt 0.30, Ti 0.40, Ta 0.75, total 2.35. Leg I: Fm 4.40, Pt 1.70, Ti 4.00, Mt 4.00, Ta 1.75, total 15.85. Leg II: Fm 5.00, Pt 1.70, Ti 4.60, Mt 4.50, Ta 1.65, total 17.45. Leg III: Fm 5.00, Pt 1.50, Ti 4.40, Mt 4.40, Ta 1.50, total 16.8.0 Leg IV: Fm 4.50, Pt 1.00, Ti 3.70, Mt 3.75, Ta 1.30, total 14.25.

*Holotype female:* Color:carapace red-brown, dusky medially and laterally; sternum orange-brown, darker around border; chelicerae red-brown, black medially to caudally; maxillae light orange-brown, lightening distally; labium orange-brown, lightening toward the distal edge; abdomen dorsally grey with lanceolate stripe with chevrons coming off near the top and center, connecting to duskier lateral edges, one dot near the end of the abdomen, festoon present; ventrally cream-colored, dark on laterally, caudally; legs orange-brown, annulations present, legs darkening distally, brown at metatarsi, tarsi. Carapace: 1.10 times longer than broad; fovea longitudinal, broad, very shallow. Eyes:AER nearly straight; PER slightly recurved; PME larger than AME, PLE largest, ALE smallest; eye diameters, AME 0.23, ALE 0.18, PME 0.28, PLE 0.35; interdistances AME-PME 0.08, PME-ALE 0.18, ALE-PLE 0.43. PME-PME 1.30. ALE-ALE 1.88; ocular quadrangle AME-AME 0.45, PLE-PLE 2.15; clypeus 0.07 high. Mouthparts:chelicerae with stout setae medially and anteriorly; maxillae longer than broad, with tuft of conspicuous setae distally; labium distally rounded. Sternum:1.11 times longer than broad, posteriorly indented Legs:leg I only slightly shorter than legs II, III and IV; leg formula 3241; scopulaepresent on all 4 tarsi and metatarsi and tibiae I and II; tarsi I-IV with strong claw tufts; prolateral claw per foot slightly toothed; spination: leg I, Fm pr 1–1–0, d 1–1–1, rl 0–1–1; Ti d 0, v 2–2–2; mt v 2–2; leg II, Fm pr 1–1–0, d 1–1–1, rl 0–1–1; Ti v 2–2–2; Mt v 2–2; leg III, Fm pr 1–0–0, d 1–1–1, rl 0; Ti v 2–2–0; Mt v 2–0; leg IV, Fm pr 1–0–0, d 1–1–1, rl 0–0–1; Ti v 1–1; Mt v 2–1. Abdomen:with terminal setal tufts. Pedipalp:claw with 5 teeth. Epigyne:sinuous opening located medially on epigynal plate, epigynal pockets present, genital openings located behind sinuous margin; internally, ducts expand posteriorly and laterally, cylindrical, though not coiled, posterodorsal fold present, covering internal ducts medially (Figs 103–104). *Dimensions:* Total length 8.85. Carapace length 4.05, width 4.40. Sternum length 2.00, width 1.80. Abdomen length 4.80, width 3.68 Pedipalp: Fm 1.00, Pt 0.50, Ti 0.50, Ta 1.00, total 3.00. Leg I: Fm 4.00, Pt 1.75, Ti 3.00, Mt 3.00, Ta 1.00, total 12.75. Leg II: Fm 4.00, Pt 2.00, Ti 3.50, Mt 3.00, Ta 1.00, total 13.50. Leg III: Fm 5.00, Pt 1.75, Ti 4.00, Mt 3.00, Ta 1.00, total 14.75. Leg IV: Fm 4.60, Pt 1.00, Ti 3.50, Mt 3.00, Ta 1.00, total 13.10.

#### Natural History.

This species has been found in dry forests under bark of *Bursera*, sea grape and on tree trunks at night. The female guards the white, disc-shaped egg sac. One female made an egg sac before 5.VI.2006, and spiderlings hatched, but stayed in the egg sac until 17.VI.2006. One was observed attacking a termite and then rejecting it. A pseudoscorpion was also found inside of an abandoned egg sac (Fig. 188)

#### Distribution.

Endemic to Jamaica and known from the southeast coast (Map 11).

### Selenops wilsoni sp. n.

urn:lsid:zoobank.org:act:7C51022E-AAC1-4ECC-80D0-FBA9703E1539

http://species-id.net/wiki/Selenops_wilsoni

Figs 107 110 Map 11

#### Type material.

Female holotype from South of Spanish Town, Hellshire Hills, St. Catherine Parish, Jamaica, 300–500 m, 17°52'N, 76°58'W, II-III.1997; B. Wilson, dry forest in limestone karst (CAS). Paratypes: Male, same data as holotype.

#### Other material examined.

JAMAICA: St. Catherine Parish: Hellshire Hills, ‘A2 Depression', (iguana site), 17°51'59.3"N, 76°57'54.0"W, ~275 m, 3.VI.2006, S. Crews, under bark, SCC06_031, 1 imm. (EME sel_381).

#### Etymology.

The specific epithet is in honor of the spiders' collector, Professor Byron Wilson of the University of the West Indies, Mona, in acknowledgement of his work regarding Jamaican biodiversity and conservation. It is to be treated as a noun in appostion.

#### Diagnosis.

**Figures 109–115.**
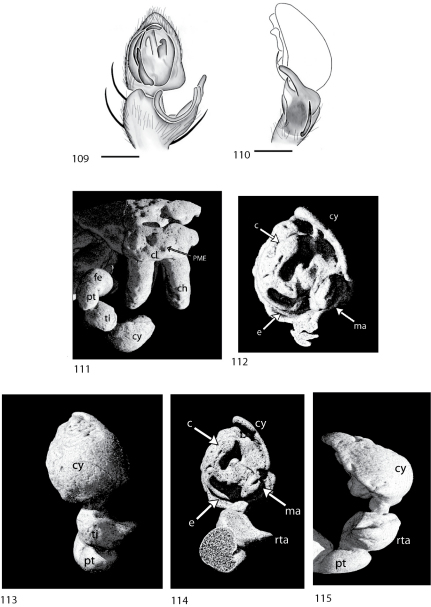
Copulatory organs of *Selenops wilsoni* sp. n. male from South of Spanish Town, Hellshire Hills, St. Catherine Parish, Jamaica (CAS) **109–110** Scans of *Selenops beynai* Schawaller holotype male from Dominican amber (SMNHS) **111–115, 109, 114** left pedipalp, ventral view **110, 115** left pedipalp, retrolateral view **111** features of the carapace, mouthparts and right pedipalp **112** right palpal bulb, rotated horizontally for consistency, ventral view. Although not perfectly preserved, several features are still apparent **113** right pedipalp, rotated for consistency, dorsal view. Scale bar = 0.50 mm. Abbreviations: **Cl** = clypeus, **Ch** = chelicerae, **PME** = posterior median eyes, **fe** = femur, **pt** = patella, **ti** = tibia, **cy** = cymbium, **c** = conductor, **ma** = median apophysis, **rta** = retrolateral tibial apophysis, **e** = embolus.

**Figures 116–120.**
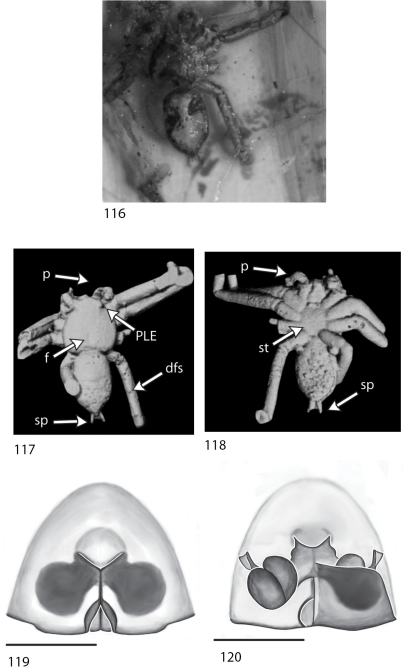
*Selenops beynai* Schawaller holotype male from Dominican amber (SMNHS) **116–118** copulatory organs of *Selenops insularis* Keyserling holotype female from Puerto Rico (MCZ) **119–120,** **116** specimen in amber **117** scan of habitus, dorsal view **118** scan of habitus, ventral view **119** epigyne, ventral view **120** spermathecae, dorsal view. Scale bar = 0.50 mm. Abbreviations: **p** = pedipalp, **PLE** = posterior retrolateral eyes, **f** = fovea, **sp** = spinnerets, **dfs** = dorsal femoral sockets, **st** = sternum.

The epigynym of *Selenops wilsoni* sp. n. somewhat resembles that of *Selenops duan* **sp. n.**, in that they both have the lateral lobes fused to form an anteriorly projecting lobe (Figs 107, 133). In *Selenops wilsoni* sp. n., the lobes are completely fused anteriorly, the epigynal plate is not ovoid, and the posterodorsal fold is very large, over half the length of the plate, completely covering the spermathecae (Figs 107–108). Also, *Selenops wilsoni* sp. n. is only known from the island of Jamaica while *Selenops duan* sp. n. is known only from the island of Hispaniola. Males can be separated from other Jamaican selenopids by their smaller size and copulatory organs, including the shape of the large, robust RTA and the overall stoutness of the median apophysis, as opposed to only a stout base as in *Selenops candidus* (Figs 109–110).

#### Remarks.

The copulatory organs of this species differ substantially from other Jamaican selenopids. The male palpal organ has a huge, bifid dorsal RTA and large median apophysis, though the branched embolus is the same as other Jamaican endemics. In the female, the anteriorly extending lobe of epigynum differs from that of all other Jamaican selenopids. Although a considerable amount of time was spent collecting in the vicinity of the type locality, only one juvenile of this species has been recovered, and many more individuals of *Selenops wilmotorum* sp. n.

#### Description.

*Paratype male:* Color: Carapace uniformly light brown; sternum pale yellow, darker around border; chelicerae uniformly light brown; maxillae dusky light orange-yellow, lightening distally; labium orange-brown, lightening toward the distal edge; abdomen dorsally yellow-brown with red-brown markings; ventrally dusky yellow-grey, no markings; legs uniform yellowish. Carapace:0.89 times longer than broad. Eyes:AER slightly recurved; PER recurved; PME larger than AME, PLE largest, ALE smallest; eye diameters, AME 0.18, ALE 0.15, PME 0.30, PLE 0.43; interdistances AME-PME 0.05, PME-ALE 0.13, ALE-PLE 0.30. PME-PME 1.13 mm. ALE-ALE 1.95; ocular quadrangle AME-AME 0.40, PLE-PLE 2.15; clypeus 0.09 high. Mouthparts:chelicerae with a few stout setae medially and anteriorly; maxillae longer than broad, with tuft of conspicuous setae distally; labium distally rounded. Sternum:as long as broad, posteriorly indented. Legs:Leg I only slightly shorter than legs II, III and IV; leg formula 2341; scopulae present on distal end of all 4 tarsi; tarsi I-IV with strong claw tufts; pr claw per foot slightly toothed; spination: leg I, Fm pr 1–1–1, d 1–1–1, rl 1–1–1; Ti d 1–1–0, pr 1–0–1, rl 1–0–1, v 2–2–2–2; Mt pr 1–1–0, v 2–2–0, rl 1–0–0; leg II, Fm pr 1–1–1, d 1–1–1, rl 1–1–1; Ti pr 1–1–0, d 1–1–0, rl 1–1–0, v 2–2–2–2; Mt pr 1–1–0, v 2–2, rl 1–0–0; leg III, Fm pr 1–1–1, d 1–1–1, rl 1–1–1; Ti pr 1–1–0, d 0–1–0, rl 1–1–0, v 2–2–2; Mt pr 1–1–0, rl 1–1–0, v 2–2–2; leg IV, Fm pr 1–1–1, d 1–1–1, rl 1–1–1; Ti pr 1–1–0, v 2–2–0, rl 1–1–0; Mt pr 1–1–0, rl 1–1–0, v 2–2–1. Abdomen:Without terminal setal tufts. Pedipalp:Femur spination pr 0–0–1, d 0–1–2, rl 0–0–1; cymbium triangular in ventral view; conductor large, attached to center of bulb by straight, narrow stalk, pointed laterally, extending to 1 o'clock position, curving around retrolateral side; embolus bifid, one branch long, slender, sightly sinuous, other wider at base, tapering slightly toward tip, beginning at 6 o'clock, terminating at 11 o'clock; MA originating at 3 o'clock, directed distally, stout, with single stout rounded hook; RTA extends anteriorly nearly half the length of cymbium; ventral tibial apophysis is large, wide u-shaped structure in ventral view, curving inwards toward cymbium; dorsal tibial apophysis wide, curving outward quite far from the cymbium, bifid, with one extension finger-like, directed distally, the other pointed, curving dorsally (Figs 109–110). *Dimensions:* Total length 7.55. Carapace length 4.10, width 4.60. Sternum length 2.00, width 2.00 Abdomen length 3.45, width 2.25. Pedipalp: Fm 1.25, Pt 0.25, Ti 0.70, Ta 0.75, total 2.95. Leg I: Fm 4.25, Pt 1.30, Ti 4.25, Mt 4.40, Ta 2.25, total 16.50. Leg II: Fm 5.00, Pt 2.00, Ti 5.10, Mt 5.10, Ta 2.25, total 19.50. Leg III: Fm 5.10, Pt 2.00, Ti 3.90, Mt 5.00, Ta 2.25, total 18.30. Leg IV: Fm 4.50, Pt 1.25, Ti 4.50, Mt 4.60, Ta 2.10, total 16.95.

*Holotype female:* Color:carapace uniformly brown-red; sternum light yellow-brown with darker border; chelicerae uniformly brown-red; maxillae light yellow-brown, lightening to white distally; labium light brown, lightening distally; abdomen dorsally grey-tan, dark festoon pattern caudally; ventrally dusky grey with no markings; legs yellowish with annulations on femora and tibiae, darkening toward tarsus, though annulations still visible. Carapace:0.91 times longer than broad. Eyes: AER nearly straight; PER slightly recurved; PME larger than AME, PLE largest, ALE smallest; eye diameters, AME 0.35, ALE 0.20, PME 0.40, PLE 0.50; interdistances AME-PME 0.10, PME-ALE 0.40, ALE-PLE 0.35. PME-PME 1.30. ALE-ALE 2.20; ocular quadrangle AME-AME 0.50, PLE-PLE 2.02; clypeus 0.03 high. Mouthparts:Chelicerae with stout setae medially and anteriorly; maxillae longer than broad, with tuft of conspicuous setae distally; labium distally rounded. Sternum:as long as broad, posteriorly indented. Legs:Leg I only slightly shorter than legs II, III and IV; leg formula 4321; scopulae present on all 4 tarsi and metatarsi and tibiae I and II; tarsi I-IV with strong claw tufts; pr claw per foot slightly toothed; spination: leg I, Fm pr 1–1–0, d 1–1–1, rl 1–0–1; Ti d 0, v 2–2–2; Mt v 2–2; leg II, Fm pr 1–0–0, d 1–1–1, rl 1–1–1; Ti v 2–2–2; Mt v 2–2; leg III, Fm pr 1–0–0, d 1–1–1, rl 1–1–1; Ti v 2–2–0; Mt v 2–2–1; leg IV, Fm pr 1–0–0, d 1–1–1, rl 1–1–1; Ti v 2–2–0; Mt v 2–2–1. Abdomen:Without terminal setal tufts. Pedipalp:Claw with 11 teeth. Epigyne:lateral lobes fused anteromedially, forming anteriorly-directed lobe; genital openings located on extreme lateral edges of lobe; separation of lateral lobes visible medially, extends anteriorly approximately 1/3rd of the length of epigynal plate; internally, ducts located medially, extend to rounded, cylindrical spermathecae, fertilization ducts located medially, extend laterally; posterodorsal fold very large,heavily sclerotized, covering internal copulatory organs except ends of fertilization ducts (Figs 107–108). *Dimensions:* Total length 8.50. Carapce length 3.80, width 4.20. Sternum length 1.90, width 1.90. Abdomen length 4.25, width 3.30. Pedipalp: Fm 0.75, Pt 0.40, Ti 0.60, Ta 1.00, total 2.75. Leg I: Fm 3.60, Pt 1.50, Ti 3.00, Mt 2.40, Ta 1.25, total 11.75. Leg II: Fm 3.75, Pt 1.50, Ti 3.65, Mt 2.75, Ta 1.30, total 12.95. Leg III: Fm 4.00, Pt 1.50, Ti 3.30, Mt 3.00, Ta 1.30, total 13.10. Leg IV: Fm 4.25, Pt 1.40, Ti 3.50, Mt 3.00, Ta 1.25, total 13.40.

#### Natural history.

Collected from a dry forest in limestone karst during the months of February-March at 500 m elevation.

#### Distribution.

Endemic to Jamaica and is known only from the type locality (Map 11).

### † Selenops beynai

Schawaller, 1984

http://species-id.net/wiki/Selenops_beynai

Figs 111–118

Selenops beynai Schawaller 1984:3, Figs 1–5 (♂, examined).

#### Type material.

Holotype male from northern Dominican Republic, in amber (SMNHS).

#### Diagnosis.

Males can be distinguished from other species by the shape of the RTA and the location of the MA and embolus (Figs 112–115).

#### Remarks.

This species was first described by Schawaller (1984). Despite HR-CT scanning (Penney et al. 2007), details of this spider, in particular the copulatory organs, are still lacking. One palp is poorly preserved and the other is damaged. Other juvenile specimens from the SMNHS are beautifully preserved, however, it is unknown whether these are the same species. Dominican amber has been dated as being 16 myo (Iturralde-Vinent 2001).

Description.
*Holotype male:* Color:overall greyish, though somewhat difficult to see due to poor preservation and color of the amber, no markings visible on the carapace or abdomen; legs yellowish with annulations on femora and tibiae. Carapace:1.08 times longer than broad. Eyes: AER nearly straight; PER slightly recurved; AME larger than PME, PLE largest, ALE smallest; eye diameters, AME 0.33, ALE 0.19, PME 0.28, PLE 0.36. Mouthparts:Chelicerae with stout setae medially and anteriorly; maxillae longer than broad, with tuft of conspicuous setae distally; labium distally rounded. Sternum:longer than wide, posteriorly indented. Legs:Leg I only slightly shorter than legs II, III and IV; leg formula 2431; spination: leg I, Fm pr 1–1–1, d 1–1–0, rl 1–1–1; leg II, Fm pr 1–1–0, d 0–1–1, rl 1–1–1; leg III, missing; leg IV, Fm pr 0–1–1, d 0–1–1, rl 1–1–0. Pedipalm:cymbium oval in ventral view; conductor large, attached to center of bulb curving around lateral margin; embolus beginning at 6 o'clock, curving around the lateral margin of cymbium; MA originating at 4 o'clock; RTA doesn't reach cymbium in ventral view; both apophyses small. *Dimensions:* Total length 8.60. Carapace length 4.00, width 3.70. Abdomen length 4.60, width 3.00. Pedipalp: Fm 1.10, Pt 0.25, Ti 0.50, Ta 1.00, total 2.85. Leg I: Fm 4.00. Leg II: Fm 5.35. Leg III: Fm 4.55. Leg IV: Fm 4.85.

#### Natural history.

This species is only known from a single fossil specimen, and thus, the natural history is unknown. However, as it was found in amber and many species of Caribbean *Selenops* are found under bark, it is likely that this species was also found under the bark and on the trunks of trees.

#### Distribution.

Known only from a single specimen in Dominican amber.

### Selenops insularis

Keyserling, 1881

http://species-id.net/wiki/Selenops_insularis

**Map 12.**
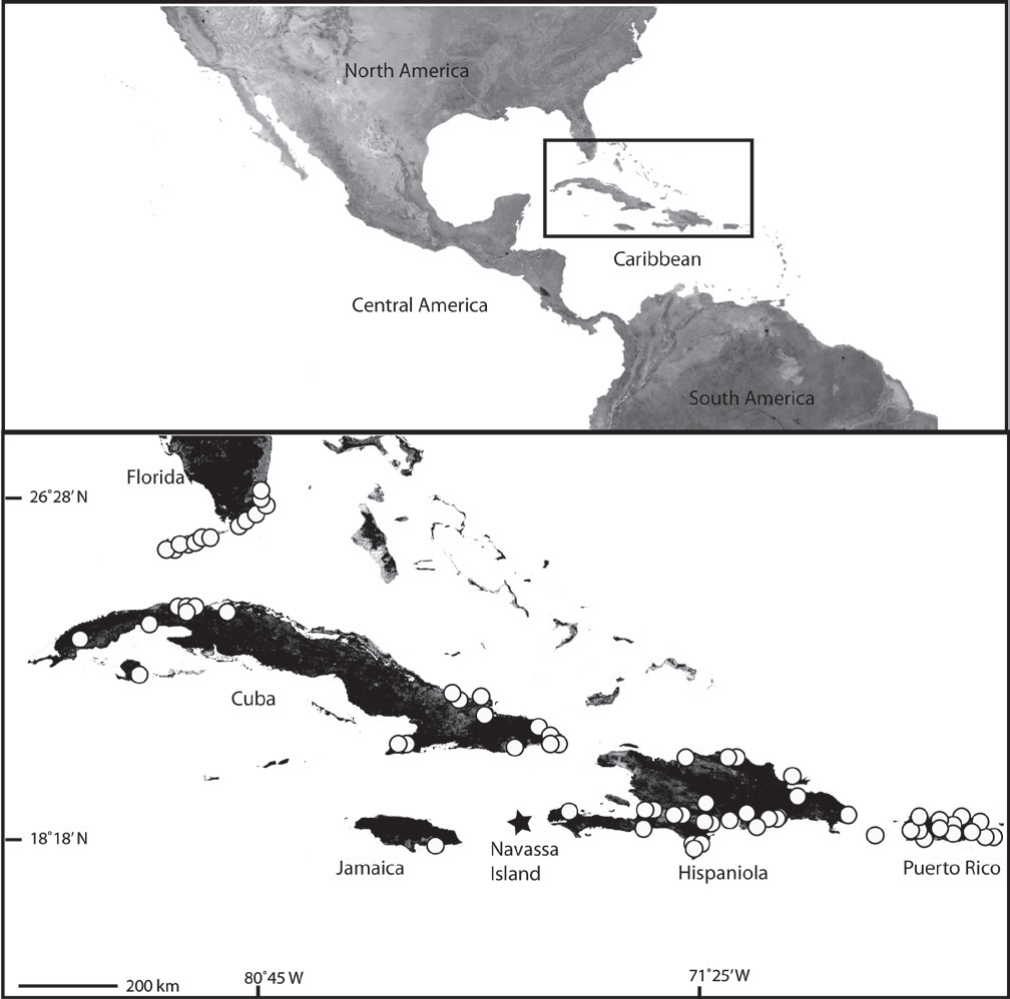
Map of southern Florida, the Bahamas, and the Greater Antilles (inset) showing the distribution of two *Selenops* species, *Selenops insularis* Keyserling (white circles) and *Selenops trifidus* Bryant (black star).

Figs 119 122 189 206 Map 12

Selenops insularis Keyserling 1881: 311, pl. 11, Fig. 28 (♀, examined).
Selenops insularis :Petrunkevitch 1920: 31, Figs 21–25 (♀, ♂).
Selenops insularis :Muma 1953: 24, Figs 37–40 (♂, ♀).
Selenops insularis :Alayón-García 2005: 13, Figs 2–5 (♂, ♀).

#### Type material.

Female holotype: Puerto Rico (MCZ, examined).

#### Other material examined.

CUBA: taken on dead twig, excellent protective resemblance, N. Banks, 2♀ (MCZ). **Havana:** N. Banks, 1♀ (MCZ); 1♂ (MCZ). **Banes:** Oriente, 103.VIII.1955, A.F. Archer, 1♀, 1 imm. (AMNH). **Santa Marta:** San Francisco, 1.III.1944, C. Vascos (AMNH). **Santiago de Las Vegas:** N. Banks, 2♀ (MCZ); 1♀ (MCZ). **DOMINICAN REPUBLIC: Azua:** Padre las Casas, 24.XII.2002, D. Veloz, 1♀, 1p♂, 1♂ (MNHNSD). **Provincia Barahona:** 6 km from Cabral, 22.IV.1979, JAD, DGR, on *Coccothrinax*, 1♀ (MNHNSD, A-104);CoralSol Resort, 18°03.512'N, 71°06.718'W, 29.VI.2006, sea level, L. Mahler, concrete fencepost by walkway near light, 1♀, 8 imm. (EME sel_537-545); CoralSol Resort, 18°03.512'N, 71106.718'W, 11.VII.2006, sea level, L. Mahler, under rook at forest edge near beach, 1p♂ (EME sel_567); CoralSol Resort, 18°03.512'N, 71°06.718'W, 12.VII.2006, sea level, L. Mahler, in bathroom, 1♂, (CAS sel_564); CoralSol Resort, 18°03.512'N, 71°06.718'W, 13.VII.2006, sea level, L. Mahler, under baseboard near roof, under bark of stump, guarding eggs, 2♀ (CAS sel_561, 563); CoralSol Resort, 18°03.512'N, 71°06.718'W, 14.VII.2006, sea level, L. Mahler, bromeliad, 2♀ (EME sel_575-576); CoralSol Resort, 18°03.512'N, 71°06.718'W, 15.VII.2006, sea level, L. Mahler, on wall, 1♂, 1 imm. (EME sel_573-574); CoralSol Resort, 18°03.512'N, 71°06.718'W, 2.VIII.2006, sea level, L. Mahler, in bed, 1 imm. (EME sel_588); CoralSol Resort, 18°03.512'N, 71°06.718'W, 3.VIII.2006, sea level, L. Mahler, on egg sac, 1 imm. (EME sel_587); San Rafael Beach 18.01.761 N, 71.08.201 W, 14.VII.2006, sea level, L. Mahler, under bark, 2 imm. (EME sel_559-560); Carretera El Higuero-Polo, 18°00.251'N, 71°18.838'W, ~466 m, 26.XI.2004, S. Crews, under bark, SCC04_083, 2 imm. (EME sel171, 173). **Hato Major:** near Los Haiteses, 1 km S of El Valle, 10.VII.2006, E. Fernandez, 1 imm. (MNHNSD sel_555). **Independencia:** Isla Cabritos, La Descubierta, Lago Enriquillo, 1.VIII.1981, E. Marcano, 1♀ (MNHNSD); Lago Enriquillo, 18°33.685'N, 71°41.835'W, 16.VII.2006, -11 m, L. Mahler, in bathroom, near trail under rock, 4 imm. (EME sel_569-572, 599); La Descubierta, El Azufrade, north margin of Lago Enriquillo, 18°33.75'N, 71°41.853'W, -15 m, 26.XI.2004, S. Crews, under rocks along trail to lake, SCC04_084, 1♀, 1 imm. (CAS sel_184,187). **La Altagracia:** Punta Cana, Punta Cana Resort, 5-8.VII.2006, sea level, L. Mahler, S. Crews, on trees at night, 1♀, 1♂, 5 imm. (CAS sel_530-536); Parque del Este, Boca de Yuma, 18°21.875'N, 68°37.080'W, ~23 m, 29-30.XI.2004, S. Crews, under bark, SCC04_089, 6 imm. (EME sel_191-194,197, 199). **Monti Cristi:** El Morro, 19°89233'N, 71°65688'W, 22.VII.2006, 20-40 m, L. Mahler, under rock beside road, 1 imm. (EME sel_581); El Morro, 19.89233°N, 71.65688°W, 8.X.2006, 20-40 m, S. Crews, under rocks, bark, SCC06_069, 1♀, 2 imm. (EME sel_612-613, 617). **Provincia Pedernales:** Oviedo, 22.V.1999, K.P.M. Chapa, 1♂ (MNHNSD, A-65); Oviedo, house of Y. Arias, 25.X.2003, S. Crews, 1 imm. (EME sel_020); Oviedo, Fondo de Mamá Cocoño, 17°48.817'N, 71°26.514'W, 342', 25.X.2003, S. Crews, S. Kanwischer, dry forest under bark, on dry, dead trees, SCC03_021, 1♀, 1 imm. (CAS sel_018-019); Parque Nacional de Jaragua, 17.81205°N, 71.44276°W, 3.VIII.2006, L. Mahler, under bark, 1♀ (EME sel_586). **Provincia Peravia:** Baní, Río Nizao, 18°16.915'N, 70°12.101'W, ~52 m, 27.XI.2004, S. Crews, under bark, SCC04_087, 1♂, 3 imm. (MNHNSD sel_176-179); road from Baní to Manaclar, past La Laguna, 18°21.343'N, 70°21.077'W, ~251 m, 27.XI.2004, S. Crews, under bark of *Bursera*, SCC04_086, 1 imm. (EME sel_175). **Puerto Plata:** Puerto Plata, entrance to Loma de Isabel, ranger's station, 19°46'41.9" N, 70°42'01.1" W, 7.X.2006, 96 m, S. Crews, under bark, on trees at night, SCC06_068, 6 imm. (EME sel_597, 602, 605, 607-609). **Sosúa:** 19.78041°N, 70.50755°W, 25.VII.2006, sea level, L. Mahler, under bark, 1 imm. (EME sel_578). **Samaná:** Las Terrenas, 19.32364°N, 69.53108°W, 27.VII.2006, sea level, L. Mahler, under bark near beach, 1♀, 3 imm. (EME sel_577, 591, 593-594). **San Cristóbal:** Engombe, farm of Autonomous University, 18°27.360'N, 70°00.306'W, down dirt road near cocoa field, S. Crews, L. Lopez, D. Veloz, under bark of *Acacia* along road, SCC03_023, 1♀, 1♂, 6 imm. (CAS sel_010-017); Santo Domingo: 15.I.1985, C. Mateo, 1♀ (MNHNSD, A-80); Jardin Botanic, 18-22.III.1984, H., L. Levi, 1♀ (MCZ). **HAITI: Grand'Anse:** Whler. 1♂, 1♀ (MCZ). **Jacmel:** St. Cyr, 18°14'16.6"N, 72°31'41.2"W, 23.X.2006, sea level, S. Crews, on dry forest trees, landscape around a house, at night, SCC06_078, 1♀, 1♂, 1p♂, 5 imm. (CAS sel_657-660, 662-665). **Petionville:** 26.VII.1958, Slazell, 1♀ (AMNH). **Port Au Prince:** 1♀ (MCZ). **JAMAICA: Kingston:** VI.1912, C.J. Brues, 1♀ (MCZ); 1.III.1935, Blackweller, Chapin, 1♀ (USNM). **PUERTO RICO:** 30.IV.1905, L. Warneke, 3♂ (AMNH); Santuvee, 30.VIII.1968, R. Gil, in house, 1♀ (FSU). **Arecibo:** Arenalejos, Carretera 657, km 1.9, 18°25'15.9"N, 66°40'35.2"W, 141 m, saw several egg sacs, 7.VI.2006, S. Crews, A. Puente-Rolón, M. Nevaraez, under bark of *Krugiodendrum ferreum*, and *Bursera simaruba*, SCC06_035, 1♀, 2 imm. (EME sel_394-396); between Barceloneta and Arecibo, Bosque Cambalacheo, 18°27'07.0"N, 66°35'49.9"W, 9.VI.2006, S. Crews, O. Monzon, under bark of *Bursera* and surrounding trees, dry forest, SCC06_041; 1♂, 1 imm. (EME sel_419-420). **Ceiva:** Los Corchos, 18°12'13.8"N, 65°40'06.5"W, sea level-3 m, 8.VI.2006, S. Crews, O. Monzon, SCC06_040, 1 imm. (EME sel_417). Ciales: Bosque Fronton, Carretera 146, km 16.3 Interior Camino Maximo Nuñez, Sector Los Gonzalez, 18°18'33.8" N, 66°32'42.8" W, ~21 m, 15.VI.2006, A. Puente-Rolón, in house, SCC06_060, 1♂ (CAS sel_503). **Coamo:** Baños de Coamo, night collecting, wire fence and bark, 2.IV.1990, H.L. Levi, 1♀, 1♂, 2 imm. (MCZ); Baños de Coamo, 18°02'19.4"N, 66°22'27.0"W, ~78 m, 11.VI.2006, S. Crews, A. Puente-Rolón, under bark of fence posts, SCC06_048, 2♀ (CAS sel_462-463); Coamo Springs, 5-7.VI.1915, 1♀ (AMNH); Fajardo: Seven Seas Public Beach, 18°22'03.7"N, 65°38'04.9"W, sea level–2 m, 8.VI.2006, dry coastal forest, under bark, S. Crews, O. Monzon, SCC06_039, 2♀, 3 imm. (EME sel_405, 407, 409, 411, 415). **Hormigueros:** 9.III.1962, A. Velez, 1♀, imms. (AMNH). **Humacao:** Barrio Collores, 11.VI.2006, S. Vega, under bark of mahogany, SCC06_051, 3♀, 1♂, 1p♂, 3 imm. (CAS sel_470-471, 473-475, 477-479); **Isla Mona:** Sardinera, IX.2006, A. Puente-Rolón, 3♀, 1p♂, 3 imm. (EME sel_838-844); VIII.1944, H. Beatty, 3♀, 1♂, 1 imm. (MCZ). **Jayuya:** Gripiñas, Rt. 527, coffee, bananas, 30.III.1990, H.L. Levi, 1♂; 5.IV.1944, Semalles, 1♀ (MCZ). **Manuabo:** Mariani Creek, 18°00'29.7" N, 65°52'17.0" W, ~34 m, 11.VI.2006, S. Crews, A. Puente-Rolón, under bark, SCC06_050, 1 imm. (CAS sel_467). **Maricao:** Bosque Estatl de Maricao, 18°08'51.2" N, 66°59'35.0" W, ~700-800 m, 10.VI.2006, S. Crews, A. Puente-Rolón, under rocks, SCC06_045, 2♀, 2 imm. (EME sel_435, 438, 442); Hacienda Juanita, old coffee plantation, night collecting, 600 m, 1-2.IV.1989, H.L. Levi, 1♀, 1♂ (MCZ). **Mayaguez:** VIII.1935, K.H. Bartlett, 1♀ (MCZ); IV.1963, J.L. Colón, 1♀ (AMNH); 15.II.1962, D. Pabon, 1♀ (AMNH). **Quebradillas:** Merendero de Guajataca, 18°29'23.7"N, 66°56'59.4"W, ~ 46 m, 9.VI.2006, S. Crews, O. Monzon, under bark of *Bursera*, *Casuarina*, sea grape, SCC06_043, 2♀, 2♂, 5 imm. (CAS sel_423-426, 428-432). **Sabana Grande:** Susúa State Forest, 18°04'15.0"N, 66°54'31.6"W, ~207 m, 10.VI.2006, S. Crews, A. Puente-Rolón, dry serpentine forest, under rocks, SCC06_046, 2♀, 1♂, 4 imm. (EME sel_443, 445, 448-449, 451-453). **San German:** II.1963, E. Lopez, 1♂, imms. (AMNH). **San Jaun:** 26-27.V.1915, 1♀. **Toa Baja:** Bosque Media Luna, PR-2, km 21-6, 14.VI.2006, M. Nevaraez, under bark of *Bursera*, SCC06_058, 1 imm. (EME sel_461). **Vieques:** Entrada Caño Hondo NR, Puerta Mosquito, 18°06'11.0" N, 65°27'05.5" W, sea level, 19.VI.2006, S. Crews, E. Bermudez, *Acacia* wetland, SCC06_061, 1 imm. (EME sel 507); entrance to Caño Hondo NR, off road 997, km 5, 18°06'47.6"N, 65°27'13.0"W, ~6 m, 19.VI.2006, S. Crews, E. Bermudez, under bark, SCC06_062, 2♀, 1 imm. (CAS sel_509-511); Ruinas Central Playa Grande, 18°05'43.2"N, 65°31'13.2"W, ~26 m, 19.VI.2006, S. Crews, E. Bermudez, under bark, SCC06_064, 2 imm. (EME sel_519-520); Laguna Kiani, 18°07'02.2"N, 65°33'41.4"W, ~24 m, 19.VI.2006, S. Crews, E. Bermudez, under bark, on boardwalk, SCC06_065, 1♀, 1♂, 3 imm. (CAS sel_522-526). **UNITED STATES: Florida:** Dade Co., Elliot Key, 27.XII.1982, H.V. Weems, 3♀ (FSU); Dade Co., Matheson Hammock, 20.VI.1964, T.J. Walker, on tree trunk in dense mesic hammock, 1♀, 1♂ (FSU); Monroe Co., Tavernier, 7.III.1964, H.L. Levi, 1♂, 1 imm. (MCZ); 15 miles NE of Key Largo, 15.VI.1974, J.B. Heppner, under bark, 1♀ (FSU); Key Largo, under bark, 26.III.1957, H.V. Weems, Jr., 1♂, 3♀ (AMNH); Monroe Co., Key Largo, 26.III.1957, H.V. Weems, under bark, 1♀, 2♂ (FSU); Stock Island, 12.V.1961, H.V. Weems, under dead *Eugenia* species in hammock, 1♀ (FSU); Plantation Key, 29.II.1936, 1♂ (AMNH); Key West, 24.VI.1934, E.L. Pierce, Jr., 1♀ (AMNH); Key West, 1.VII.1935, 1♂ (AMNH); **Washington DC:** on bananas, 1♀ (MCZ).

#### Diagnosis.

**Figures 121–128.**
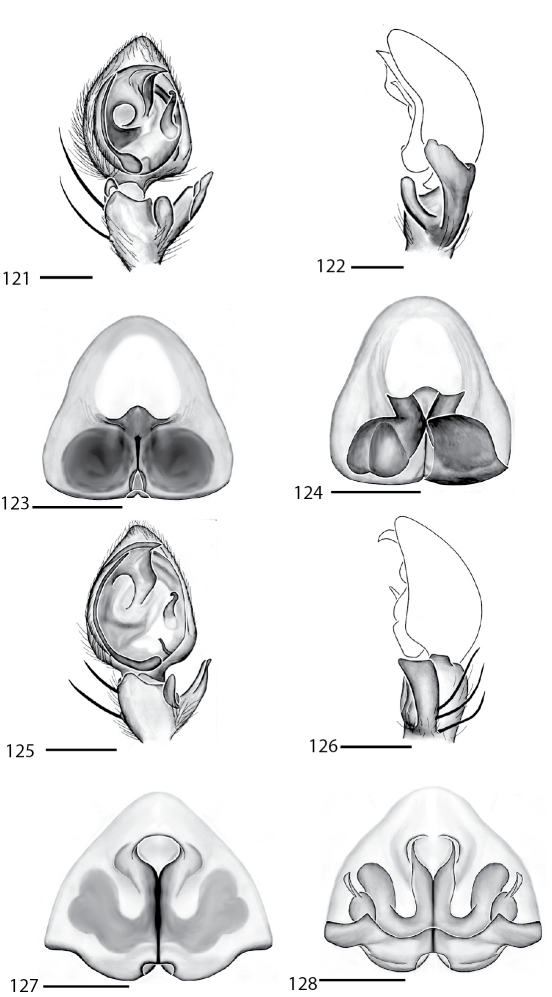
Copulatory organs of *Selenops insularis* Keyserling male from St. Cyr, Jacmel, Haiti (CAS sel_657) **121–122** *Selenops marcanoi* Alayón-García female from El Banano, Rio Mulito, Pedernales, Dominican Republic (EME sel_151) **123–124** male from El Banano, Rio Mulito, Pedernales, Dominican Republic (EME sel_152) **125–126** *Selenops bocacanadensis* sp. n. holotype female from kilometer 13 along Carretera ALCOA, Pedernales, Dominican Republic (EME sel_166) **127–128, 121, 125** left pedipalp, ventral view **122, 126** left pedipalp, retrolateral view **123, 127** epigyne, ventral view **124, 128** spermathecae, dorsal view. Scale bar = 0.50 mm.

This species is most similar in habitus to *Selenops bocacanadensis* sp. n., but the females can be easily distinguished by the copulatory organs in that in *Selenops insularis* the genital openings occur along a small v-shaped opening (Figs 119–120). The male is most similar to *Selenops trifidus*, but can be distinguished by the copulatory organs in that the dorsal branch of the RTA of *Selenops insularis* widens distally, and is longer anteriorly, and the MA is slender throughout (Figs 121–122).

#### Remarks.

It is likely this species isn't established in Jamaica, as both records were from Kingston, a major shipping port, and are quite old. Recent searching in Jamaica turned up no specimens of this species. Muma (1953) mentioned that the females of this species are variable, but he did not elaborate. There is some variation in size in both males and females, but none was noticed in the copulatory organs of the many specimens examined. Muma (1953) suggested that some variants may be females of *Selenops trifidus*. Given the isolation of that species on Navassa Island, I believe this to be unlikely.

#### Description.

*Male (sel_657):* Color:carapace uniform dusky yellow to brown; sternum dusky yellow; chelicerae with a tan central area enclosed by brown; maxillae dusky yellow; labium brown, lightening distally; abdomen dorsally light grey to tan with a pair of small dark median spots and black laterocaudal festoon; ventrally dusky with light longitudinal stripes; legs dusky yellow, darkening to brown-grey distally, annulations not visible. Carapace: 0.88 times longer than broad. Eyes:AER nearly straight; PER slightly recurved; PME larger than AME, PLE largest, ALE smallest; eye diameters, AME 0.30, ALE 0.10, PME 0.33, PLE 0.40; interdistances AME-PME 0.15, PME-ALE 0.20, ALE-PLE 0.40. PME-PME 1.45. ALE-ALE 2.40; ocular quadrangle AME-AME 0.50, PLE-PLE 2.48; clypeus 0.08 high. Mouthparts:chelicerae with stout setae medially and anteriorly; maxillae longer than broad, with tuft of conspicuous setae distally; labium distally rounded. Sternum:as long as broad, posteriorly indented. Legs:leg I much shorter than legs II, III and IV; leg formula 2314; scopulae present on tarsi I-IV and metatarsi of legs I and II; tarsi I-IV with strong claw tufts; pr claw per foot slightly toothed; spination: leg I, Fm pr 1–1–1, d 1–1–1, rl 1–1–1; Ti pr 1–0–1, d 1–2–0, rl 1–0–1, v 2–2–2–2; Mt pr 1–1–0, rl 1–1–0, v 2–2; leg II, Fm pr 1–1–1, d 1–1–1, rl 1–1–1; Ti pr 1–1–0, rl 1–1–0, d 1–1–0, v 2–2–2–2; Mt pr 1–1–0, rl 1–0–0, v 2–2; leg III, Fm pr 1–1–1, d 1–1–1, rl 1–1–1; Ti pr 1–0–1, rl 1–0–1, v 2–2; Mt pr 1–1–0, rl 1–1–0, v 2–2; leg IV, Fm pr 1–1–0, d 1–1–1, rl 0–1–1; Ti pr 1–1–0, v 2–2–0, rl 1–1–0; Mt pr 1–1–0, rl 1–0–0, v 2–0–1. Abdomen:without terminal setal tufts. Pedipalp:Fm, spination d 0–1–3; cymbium oval to angular in ventral view, slightly angled posterolaterally; conductor arising from short, straight stalk anteromedially, pointing laterally, curving retrolaterally around cymbium, reconnecting to bulb forming circular space between two conductor connections; embolus long, slender, curved, beginning at 6 o'clock, terminating at 12 o'clock; MA arising at 3 o'clock, directed anteriorly, narrow, sinuous, tip rounded, curved to form small single hook; two tibial apophyses, ventral apophysis curves ventrally, slightly smaller than lateral apophysis, rounded terminally; lateral apophysis curves away from then back toward cymbium, widening distally, longer anteriorly; tibial apophyses extend at least 1/6th length of cymbium in ventral view (Figs 121–122). *Dimensions:* Total length 9.45. Carapace length 4.50, width 5.13. Sternum length 2.75, width 2.75. Abdomen length 4.95, width 3.10. Pedipalp: Fm 1.50, Pt 0.30, Ti 0.55, Ta 1.20, total 3.55. Leg I: Fm 5.75, Pt 1.85, Ti 5.75, Mt 5.50, Ta 2.20, total 21.05. Leg II: Fm 6.75, Pt 2.00, Ti 6.75, Mt 6.00, Ta 2.25, total 23.75. Leg III: Fm 6.25, Pt 1.75, Ti 6.60, Mt 5.75, Ta 2.00, total 22.35. Leg IV: Fm 5.90, Pt 1.75, Ti 5.00, Mt 5.00, Ta 1.85, total 19.50.

*Holotype female:* Color:carapace (holotype) dark red-brown, some white setae (recent) dark red-brown to yellow-brown to brown, typically darker in cephalic area, with white setae; sternum (holotype) transparent brown (recent) light orange-brown, darker around border; chelicerae (holotype) dark brown (recent) orange-red-brown; maxillae (holotype) dark brown, lightening distally (recent) light orange-brown, lightening distally; labium (holotype) dark brown, lightening distally (recent) light brown lightening distally; abdomen dorsally (holotype) uniform brown (recent) pale cream to grey, two dark small dots in center, sometimes smaller dark flecks, dark laterocaudal festoon prominent; ventrally (holotype) grey-brown (recent) yellow-grey with two yellow rectangular areas medially, lots of yellow flecks on grey background; legs (holotype) orange-brown (recent) slightly lighter than carapace, annulations prominent on femora, legs darkening from patella distally. Carapace:0.91 times longer than broad; fovea longitudinal, broad, very shallow. Eyes:AER nearly straight; PER slightly recurved; PME larger than AME, PLE largest, ALE smallest; eye diameters, AME 0.28, ALE 0.10, PME 0.33, PLE 0.50; interdistances AME-PME 0.15, PME-ALE 0.10, ALE-PLE 0.50. PME-PME 1.68. ALE-ALE 2.60; ocular quadrangle AME-AME 0.60, PLE-PLE 2.75; clypeus 0.07 high. Mouthparts:chelicerae with a few stout setae medially and anteriorly; maxillae longer than broad, with tuft of conspicuous setae distally; labium distally rounded. Sternum:as long as broad, posteriorly indented. Legs:leg I only slightly shorter than II and III; leg formula 2314; scopulae present on all 4 tarsi and on metatarsi I and II, and distally on tibia I and II; tarsi I-IV with strong claw tufts; pr claw per foot slightly toothed; spination: leg I, Fm pr 1–1–1, d 1–1–1, rl 1–1–0; Ti d 0, v 2–2–2; Mt v 2–2; leg II, Fm pr 1–0–0, d 1–1–1, rl 1–1–0; Ti v 2–2–2; Mt v 2–2; leg III, Fm pr 1–0–0, d 1–1–1, rl 1–0–1; Ti v 2–2–0; Mt v 2–2; leg IV, Fm pr 1–0–0, d 1–1–1, rl 0–0–1; Ti v 1–1; Mt v 2–1. Abdomen:without terminal setal tufts. Pedipalp:claw with 9 teeth. Epigyne:lateral lobes come into contact medially, genital openings located at margins of medial v-shaped opening, epigynal pockets present; internally, ducts extend posterolaterally, terminally coiled, rounded, fertilization ducts located laterally, directed anteriorly, posterodorsal fold large, heavily sclerotized, quadrangular, covering most of internal ducts (Figs 119–120). *Dimensions:* Total length 11.30. Carapace length 5.55, width 6.10. Sternum length 2.75, width 2.75. Abdomen length 3.73, width 3.24. Pedipalp: Fm 1.00, Pt 0.75, Ti 0.75, Ta 1.00, total 3.50. Leg I: Fm 5.50, Pt 2.40, Ti 5.00, Mt 3.75, Ta 1.75, total 18.40. Leg II: Fm 6.00, Pt 2.00, Ti 5.75, Mt 3.75, Ta 1.75, total 19.25. Leg III: Fm 6.30, Pt 2.00, Ti 5.00, Mt 4.00, Ta 1.75, total 19.05. Leg IV: Fm 5.50, Pt 1.75, Ti 4.00, Mt 4.00, Ta 1.80, total 17.05.

#### Natural History.

This species is very common and widespread. From historical records, it seems to travel easily on bananas. It is found under bark and rocks, in houses, on plants, often occurring in areas with other species of *Selenops* (Figs 189, 206). The female guards the white, disc-shaped egg sac. One egg sac had 87 eggs and never hatched. Another egg sac had 42 offspring inside.

#### Distribution.

Found in the Greater Antillean islands of Cuba, Hispaniola, Mona and Puerto Rico. It has historically been collected in Jamaica (see Remarks). It is also found in southern Florida (Map 12).

### Selenops insularis

Keyserling, 1881

http://species-id.net/wiki/Selenops_insularis

Figs 119 122 189 206 Map 12

Selenops insularis Keyserling 1881: 311, pl. 11, Fig. 28 (♀, examined).
Selenops insularis :Petrunkevitch 1920: 31, Figs 21–25 (♀, ♂).
Selenops insularis :Muma 1953: 24, Figs 37–40 (♂, ♀).
Selenops insularis :Alayón-García 2005: 13, Figs 2–5 (♂, ♀).

#### Type material.

Female holotype: Puerto Rico (MCZ, examined).

#### Other material examined.

CUBA: taken on dead twig, excellent protective resemblance, N. Banks, 2♀ (MCZ). **Havana:** N. Banks, 1♀ (MCZ); 1♂ (MCZ). **Banes:** Oriente, 103.VIII.1955, A.F. Archer, 1♀, 1 imm. (AMNH). **Santa Marta:** San Francisco, 1.III.1944, C. Vascos (AMNH). **Santiago de Las Vegas:** N. Banks, 2♀ (MCZ); 1♀ (MCZ). **DOMINICAN REPUBLIC: Azua:** Padre las Casas, 24.XII.2002, D. Veloz, 1♀, 1p♂, 1♂ (MNHNSD). **Provincia Barahona:** 6 km from Cabral, 22.IV.1979, JAD, DGR, on *Coccothrinax*, 1♀ (MNHNSD, A-104);CoralSol Resort, 18°03.512'N, 71°06.718'W, 29.VI.2006, sea level, L. Mahler, concrete fencepost by walkway near light, 1♀, 8 imm. (EME sel_537-545); CoralSol Resort, 18°03.512'N, 71106.718'W, 11.VII.2006, sea level, L. Mahler, under rook at forest edge near beach, 1p♂ (EME sel_567); CoralSol Resort, 18°03.512'N, 71°06.718'W, 12.VII.2006, sea level, L. Mahler, in bathroom, 1♂, (CAS sel_564); CoralSol Resort, 18°03.512'N, 71°06.718'W, 13.VII.2006, sea level, L. Mahler, under baseboard near roof, under bark of stump, guarding eggs, 2♀ (CAS sel_561, 563); CoralSol Resort, 18°03.512'N, 71°06.718'W, 14.VII.2006, sea level, L. Mahler, bromeliad, 2♀ (EME sel_575-576); CoralSol Resort, 18°03.512'N, 71°06.718'W, 15.VII.2006, sea level, L. Mahler, on wall, 1♂, 1 imm. (EME sel_573-574); CoralSol Resort, 18°03.512'N, 71°06.718'W, 2.VIII.2006, sea level, L. Mahler, in bed, 1 imm. (EME sel_588); CoralSol Resort, 18°03.512'N, 71°06.718'W, 3.VIII.2006, sea level, L. Mahler, on egg sac, 1 imm. (EME sel_587); San Rafael Beach 18.01.761 N, 71.08.201 W, 14.VII.2006, sea level, L. Mahler, under bark, 2 imm. (EME sel_559-560); Carretera El Higuero-Polo, 18°00.251'N, 71°18.838'W, ~466 m, 26.XI.2004, S. Crews, under bark, SCC04_083, 2 imm. (EME sel171, 173). **Hato Major:** near Los Haiteses, 1 km S of El Valle, 10.VII.2006, E. Fernandez, 1 imm. (MNHNSD sel_555). **Independencia:** Isla Cabritos, La Descubierta, Lago Enriquillo, 1.VIII.1981, E. Marcano, 1♀ (MNHNSD); Lago Enriquillo, 18°33.685'N, 71°41.835'W, 16.VII.2006, -11 m, L. Mahler, in bathroom, near trail under rock, 4 imm. (EME sel_569-572, 599); La Descubierta, El Azufrade, north margin of Lago Enriquillo, 18°33.75'N, 71°41.853'W, -15 m, 26.XI.2004, S. Crews, under rocks along trail to lake, SCC04_084, 1♀, 1 imm. (CAS sel_184,187). **La Altagracia:** Punta Cana, Punta Cana Resort, 5-8.VII.2006, sea level, L. Mahler, S. Crews, on trees at night, 1♀, 1♂, 5 imm. (CAS sel_530-536); Parque del Este, Boca de Yuma, 18°21.875'N, 68°37.080'W, ~23 m, 29-30.XI.2004, S. Crews, under bark, SCC04_089, 6 imm. (EME sel_191-194,197, 199). **Monti Cristi:** El Morro, 19°89233'N, 71°65688'W, 22.VII.2006, 20-40 m, L. Mahler, under rock beside road, 1 imm. (EME sel_581); El Morro, 19.89233°N, 71.65688°W, 8.X.2006, 20-40 m, S. Crews, under rocks, bark, SCC06_069, 1♀, 2 imm. (EME sel_612-613, 617). **Provincia Pedernales:** Oviedo, 22.V.1999, K.P.M. Chapa, 1♂ (MNHNSD, A-65); Oviedo, house of Y. Arias, 25.X.2003, S. Crews, 1 imm. (EME sel_020); Oviedo, Fondo de Mamá Cocoño, 17°48.817'N, 71°26.514'W, 342', 25.X.2003, S. Crews, S. Kanwischer, dry forest under bark, on dry, dead trees, SCC03_021, 1♀, 1 imm. (CAS sel_018-019); Parque Nacional de Jaragua, 17.81205°N, 71.44276°W, 3.VIII.2006, L. Mahler, under bark, 1♀ (EME sel_586). **Provincia Peravia:** Baní, Río Nizao, 18°16.915'N, 70°12.101'W, ~52 m, 27.XI.2004, S. Crews, under bark, SCC04_087, 1♂, 3 imm. (MNHNSD sel_176-179); road from Baní to Manaclar, past La Laguna, 18°21.343'N, 70°21.077'W, ~251 m, 27.XI.2004, S. Crews, under bark of *Bursera*, SCC04_086, 1 imm. (EME sel_175). **Puerto Plata:** Puerto Plata, entrance to Loma de Isabel, ranger's station, 19°46'41.9" N, 70°42'01.1" W, 7.X.2006, 96 m, S. Crews, under bark, on trees at night, SCC06_068, 6 imm. (EME sel_597, 602, 605, 607-609). **Sosúa:** 19.78041°N, 70.50755°W, 25.VII.2006, sea level, L. Mahler, under bark, 1 imm. (EME sel_578). **Samaná:** Las Terrenas, 19.32364°N, 69.53108°W, 27.VII.2006, sea level, L. Mahler, under bark near beach, 1♀, 3 imm. (EME sel_577, 591, 593-594). **San Cristóbal:** Engombe, farm of Autonomous University, 18°27.360'N, 70°00.306'W, down dirt road near cocoa field, S. Crews, L. Lopez, D. Veloz, under bark of *Acacia* along road, SCC03_023, 1♀, 1♂, 6 imm. (CAS sel_010-017); Santo Domingo: 15.I.1985, C. Mateo, 1♀ (MNHNSD, A-80); Jardin Botanic, 18-22.III.1984, H., L. Levi, 1♀ (MCZ). **HAITI: Grand'Anse:** Whler. 1♂, 1♀ (MCZ). **Jacmel:** St. Cyr, 18°14'16.6"N, 72°31'41.2"W, 23.X.2006, sea level, S. Crews, on dry forest trees, landscape around a house, at night, SCC06_078, 1♀, 1♂, 1p♂, 5 imm. (CAS sel_657-660, 662-665). **Petionville:** 26.VII.1958, Slazell, 1♀ (AMNH). **Port Au Prince:** 1♀ (MCZ). **JAMAICA: Kingston:** VI.1912, C.J. Brues, 1♀ (MCZ); 1.III.1935, Blackweller, Chapin, 1♀ (USNM). **PUERTO RICO:** 30.IV.1905, L. Warneke, 3♂ (AMNH); Santuvee, 30.VIII.1968, R. Gil, in house, 1♀ (FSU). **Arecibo:** Arenalejos, Carretera 657, km 1.9, 18°25'15.9"N, 66°40'35.2"W, 141 m, saw several egg sacs, 7.VI.2006, S. Crews, A. Puente-Rolón, M. Nevaraez, under bark of *Krugiodendrum ferreum*, and *Bursera simaruba*, SCC06_035, 1♀, 2 imm. (EME sel_394-396); between Barceloneta and Arecibo, Bosque Cambalacheo, 18°27'07.0"N, 66°35'49.9"W, 9.VI.2006, S. Crews, O. Monzon, under bark of *Bursera* and surrounding trees, dry forest, SCC06_041; 1♂, 1 imm. (EME sel_419-420). **Ceiva:** Los Corchos, 18°12'13.8"N, 65°40'06.5"W, sea level-3 m, 8.VI.2006, S. Crews, O. Monzon, SCC06_040, 1 imm. (EME sel_417). Ciales: Bosque Fronton, Carretera 146, km 16.3 Interior Camino Maximo Nuñez, Sector Los Gonzalez, 18°18'33.8" N, 66°32'42.8" W, ~21 m, 15.VI.2006, A. Puente-Rolón, in house, SCC06_060, 1♂ (CAS sel_503). **Coamo:** Baños de Coamo, night collecting, wire fence and bark, 2.IV.1990, H.L. Levi, 1♀, 1♂, 2 imm. (MCZ); Baños de Coamo, 18°02'19.4"N, 66°22'27.0"W, ~78 m, 11.VI.2006, S. Crews, A. Puente-Rolón, under bark of fence posts, SCC06_048, 2♀ (CAS sel_462-463); Coamo Springs, 5-7.VI.1915, 1♀ (AMNH); Fajardo: Seven Seas Public Beach, 18°22'03.7"N, 65°38'04.9"W, sea level–2 m, 8.VI.2006, dry coastal forest, under bark, S. Crews, O. Monzon, SCC06_039, 2♀, 3 imm. (EME sel_405, 407, 409, 411, 415). **Hormigueros:** 9.III.1962, A. Velez, 1♀, imms. (AMNH). **Humacao:** Barrio Collores, 11.VI.2006, S. Vega, under bark of mahogany, SCC06_051, 3♀, 1♂, 1p♂, 3 imm. (CAS sel_470-471, 473-475, 477-479); **Isla Mona:** Sardinera, IX.2006, A. Puente-Rolón, 3♀, 1p♂, 3 imm. (EME sel_838-844); VIII.1944, H. Beatty, 3♀, 1♂, 1 imm. (MCZ). **Jayuya:** Gripiñas, Rt. 527, coffee, bananas, 30.III.1990, H.L. Levi, 1♂; 5.IV.1944, Semalles, 1♀ (MCZ). **Manuabo:** Mariani Creek, 18°00'29.7" N, 65°52'17.0" W, ~34 m, 11.VI.2006, S. Crews, A. Puente-Rolón, under bark, SCC06_050, 1 imm. (CAS sel_467). **Maricao:** Bosque Estatl de Maricao, 18°08'51.2" N, 66°59'35.0" W, ~700-800 m, 10.VI.2006, S. Crews, A. Puente-Rolón, under rocks, SCC06_045, 2♀, 2 imm. (EME sel_435, 438, 442); Hacienda Juanita, old coffee plantation, night collecting, 600 m, 1-2.IV.1989, H.L. Levi, 1♀, 1♂ (MCZ). **Mayaguez:** VIII.1935, K.H. Bartlett, 1♀ (MCZ); IV.1963, J.L. Colón, 1♀ (AMNH); 15.II.1962, D. Pabon, 1♀ (AMNH). **Quebradillas:** Merendero de Guajataca, 18°29'23.7"N, 66°56'59.4"W, ~ 46 m, 9.VI.2006, S. Crews, O. Monzon, under bark of *Bursera*, *Casuarina*, sea grape, SCC06_043, 2♀, 2♂, 5 imm. (CAS sel_423-426, 428-432). **Sabana Grande:** Susúa State Forest, 18°04'15.0"N, 66°54'31.6"W, ~207 m, 10.VI.2006, S. Crews, A. Puente-Rolón, dry serpentine forest, under rocks, SCC06_046, 2♀, 1♂, 4 imm. (EME sel_443, 445, 448-449, 451-453). **San German:** II.1963, E. Lopez, 1♂, imms. (AMNH). **San Jaun:** 26-27.V.1915, 1♀. **Toa Baja:** Bosque Media Luna, PR-2, km 21-6, 14.VI.2006, M. Nevaraez, under bark of *Bursera*, SCC06_058, 1 imm. (EME sel_461). **Vieques:** Entrada Caño Hondo NR, Puerta Mosquito, 18°06'11.0" N, 65°27'05.5" W, sea level, 19.VI.2006, S. Crews, E. Bermudez, *Acacia* wetland, SCC06_061, 1 imm. (EME sel 507); entrance to Caño Hondo NR, off road 997, km 5, 18°06'47.6"N, 65°27'13.0"W, ~6 m, 19.VI.2006, S. Crews, E. Bermudez, under bark, SCC06_062, 2♀, 1 imm. (CAS sel_509-511); Ruinas Central Playa Grande, 18°05'43.2"N, 65°31'13.2"W, ~26 m, 19.VI.2006, S. Crews, E. Bermudez, under bark, SCC06_064, 2 imm. (EME sel_519-520); Laguna Kiani, 18°07'02.2"N, 65°33'41.4"W, ~24 m, 19.VI.2006, S. Crews, E. Bermudez, under bark, on boardwalk, SCC06_065, 1♀, 1♂, 3 imm. (CAS sel_522-526). **UNITED STATES: Florida:** Dade Co., Elliot Key, 27.XII.1982, H.V. Weems, 3♀ (FSU); Dade Co., Matheson Hammock, 20.VI.1964, T.J. Walker, on tree trunk in dense mesic hammock, 1♀, 1♂ (FSU); Monroe Co., Tavernier, 7.III.1964, H.L. Levi, 1♂, 1 imm. (MCZ); 15 miles NE of Key Largo, 15.VI.1974, J.B. Heppner, under bark, 1♀ (FSU); Key Largo, under bark, 26.III.1957, H.V. Weems, Jr., 1♂, 3♀ (AMNH); Monroe Co., Key Largo, 26.III.1957, H.V. Weems, under bark, 1♀, 2♂ (FSU); Stock Island, 12.V.1961, H.V. Weems, under dead *Eugenia* species in hammock, 1♀ (FSU); Plantation Key, 29.II.1936, 1♂ (AMNH); Key West, 24.VI.1934, E.L. Pierce, Jr., 1♀ (AMNH); Key West, 1.VII.1935, 1♂ (AMNH); **Washington DC:** on bananas, 1♀ (MCZ).

#### Diagnosis.

This species is most similar in habitus to *Selenops bocacanadensis* sp. n., but the females can be easily distinguished by the copulatory organs in that in *Selenops insularis* the genital openings occur along a small v-shaped opening (Figs 119–120). The male is most similar to *Selenops trifidus*, but can be distinguished by the copulatory organs in that the dorsal branch of the RTA of *Selenops insularis* widens distally, and is longer anteriorly, and the MA is slender throughout (Figs 121–122).

#### Remarks.

It is likely this species isn't established in Jamaica, as both records were from Kingston, a major shipping port, and are quite old. Recent searching in Jamaica turned up no specimens of this species. Muma (1953) mentioned that the females of this species are variable, but he did not elaborate. There is some variation in size in both males and females, but none was noticed in the copulatory organs of the many specimens examined. Muma (1953) suggested that some variants may be females of *Selenops trifidus*. Given the isolation of that species on Navassa Island, I believe this to be unlikely.

#### Description.

*Male (sel_657):* Color:carapace uniform dusky yellow to brown; sternum dusky yellow; chelicerae with a tan central area enclosed by brown; maxillae dusky yellow; labium brown, lightening distally; abdomen dorsally light grey to tan with a pair of small dark median spots and black laterocaudal festoon; ventrally dusky with light longitudinal stripes; legs dusky yellow, darkening to brown-grey distally, annulations not visible. Carapace: 0.88 times longer than broad. Eyes:AER nearly straight; PER slightly recurved; PME larger than AME, PLE largest, ALE smallest; eye diameters, AME 0.30, ALE 0.10, PME 0.33, PLE 0.40; interdistances AME-PME 0.15, PME-ALE 0.20, ALE-PLE 0.40. PME-PME 1.45. ALE-ALE 2.40; ocular quadrangle AME-AME 0.50, PLE-PLE 2.48; clypeus 0.08 high. Mouthparts:chelicerae with stout setae medially and anteriorly; maxillae longer than broad, with tuft of conspicuous setae distally; labium distally rounded. Sternum:as long as broad, posteriorly indented. Legs:leg I much shorter than legs II, III and IV; leg formula 2314; scopulae present on tarsi I-IV and metatarsi of legs I and II; tarsi I-IV with strong claw tufts; pr claw per foot slightly toothed; spination: leg I, Fm pr 1–1–1, d 1–1–1, rl 1–1–1; Ti pr 1–0–1, d 1–2–0, rl 1–0–1, v 2–2–2–2; Mt pr 1–1–0, rl 1–1–0, v 2–2; leg II, Fm pr 1–1–1, d 1–1–1, rl 1–1–1; Ti pr 1–1–0, rl 1–1–0, d 1–1–0, v 2–2–2–2; Mt pr 1–1–0, rl 1–0–0, v 2–2; leg III, Fm pr 1–1–1, d 1–1–1, rl 1–1–1; Ti pr 1–0–1, rl 1–0–1, v 2–2; Mt pr 1–1–0, rl 1–1–0, v 2–2; leg IV, Fm pr 1–1–0, d 1–1–1, rl 0–1–1; Ti pr 1–1–0, v 2–2–0, rl 1–1–0; Mt pr 1–1–0, rl 1–0–0, v 2–0–1. Abdomen:without terminal setal tufts. Pedipalp:Fm, spination d 0–1–3; cymbium oval to angular in ventral view, slightly angled posterolaterally; conductor arising from short, straight stalk anteromedially, pointing laterally, curving retrolaterally around cymbium, reconnecting to bulb forming circular space between two conductor connections; embolus long, slender, curved, beginning at 6 o'clock, terminating at 12 o'clock; MA arising at 3 o'clock, directed anteriorly, narrow, sinuous, tip rounded, curved to form small single hook; two tibial apophyses, ventral apophysis curves ventrally, slightly smaller than lateral apophysis, rounded terminally; lateral apophysis curves away from then back toward cymbium, widening distally, longer anteriorly; tibial apophyses extend at least 1/6th length of cymbium in ventral view (Figs 121–122). *Dimensions:* Total length 9.45. Carapace length 4.50, width 5.13. Sternum length 2.75, width 2.75. Abdomen length 4.95, width 3.10. Pedipalp: Fm 1.50, Pt 0.30, Ti 0.55, Ta 1.20, total 3.55. Leg I: Fm 5.75, Pt 1.85, Ti 5.75, Mt 5.50, Ta 2.20, total 21.05. Leg II: Fm 6.75, Pt 2.00, Ti 6.75, Mt 6.00, Ta 2.25, total 23.75. Leg III: Fm 6.25, Pt 1.75, Ti 6.60, Mt 5.75, Ta 2.00, total 22.35. Leg IV: Fm 5.90, Pt 1.75, Ti 5.00, Mt 5.00, Ta 1.85, total 19.50.

*Holotype female:* Color:carapace (holotype) dark red-brown, some white setae (recent) dark red-brown to yellow-brown to brown, typically darker in cephalic area, with white setae; sternum (holotype) transparent brown (recent) light orange-brown, darker around border; chelicerae (holotype) dark brown (recent) orange-red-brown; maxillae (holotype) dark brown, lightening distally (recent) light orange-brown, lightening distally; labium (holotype) dark brown, lightening distally (recent) light brown lightening distally; abdomen dorsally (holotype) uniform brown (recent) pale cream to grey, two dark small dots in center, sometimes smaller dark flecks, dark laterocaudal festoon prominent; ventrally (holotype) grey-brown (recent) yellow-grey with two yellow rectangular areas medially, lots of yellow flecks on grey background; legs (holotype) orange-brown (recent) slightly lighter than carapace, annulations prominent on femora, legs darkening from patella distally. Carapace:0.91 times longer than broad; fovea longitudinal, broad, very shallow. Eyes:AER nearly straight; PER slightly recurved; PME larger than AME, PLE largest, ALE smallest; eye diameters, AME 0.28, ALE 0.10, PME 0.33, PLE 0.50; interdistances AME-PME 0.15, PME-ALE 0.10, ALE-PLE 0.50. PME-PME 1.68. ALE-ALE 2.60; ocular quadrangle AME-AME 0.60, PLE-PLE 2.75; clypeus 0.07 high. Mouthparts:chelicerae with a few stout setae medially and anteriorly; maxillae longer than broad, with tuft of conspicuous setae distally; labium distally rounded. Sternum:as long as broad, posteriorly indented. Legs:leg I only slightly shorter than II and III; leg formula 2314; scopulae present on all 4 tarsi and on metatarsi I and II, and distally on tibia I and II; tarsi I-IV with strong claw tufts; pr claw per foot slightly toothed; spination: leg I, Fm pr 1–1–1, d 1–1–1, rl 1–1–0; Ti d 0, v 2–2–2; Mt v 2–2; leg II, Fm pr 1–0–0, d 1–1–1, rl 1–1–0; Ti v 2–2–2; Mt v 2–2; leg III, Fm pr 1–0–0, d 1–1–1, rl 1–0–1; Ti v 2–2–0; Mt v 2–2; leg IV, Fm pr 1–0–0, d 1–1–1, rl 0–0–1; Ti v 1–1; Mt v 2–1. Abdomen:without terminal setal tufts. Pedipalp:claw with 9 teeth. Epigyne:lateral lobes come into contact medially, genital openings located at margins of medial v-shaped opening, epigynal pockets present; internally, ducts extend posterolaterally, terminally coiled, rounded, fertilization ducts located laterally, directed anteriorly, posterodorsal fold large, heavily sclerotized, quadrangular, covering most of internal ducts (Figs 119–120). *Dimensions:* Total length 11.30. Carapace length 5.55, width 6.10. Sternum length 2.75, width 2.75. Abdomen length 3.73, width 3.24. Pedipalp: Fm 1.00, Pt 0.75, Ti 0.75, Ta 1.00, total 3.50. Leg I: Fm 5.50, Pt 2.40, Ti 5.00, Mt 3.75, Ta 1.75, total 18.40. Leg II: Fm 6.00, Pt 2.00, Ti 5.75, Mt 3.75, Ta 1.75, total 19.25. Leg III: Fm 6.30, Pt 2.00, Ti 5.00, Mt 4.00, Ta 1.75, total 19.05. Leg IV: Fm 5.50, Pt 1.75, Ti 4.00, Mt 4.00, Ta 1.80, total 17.05.

#### Natural History.

This species is very common and widespread. From historical records, it seems to travel easily on bananas. It is found under bark and rocks, in houses, on plants, often occurring in areas with other species of *Selenops* (Figs 189, 206). The female guards the white, disc-shaped egg sac. One egg sac had 87 eggs and never hatched. Another egg sac had 42 offspring inside.

#### Distribution.

Found in the Greater Antillean islands of Cuba, Hispaniola, Mona and Puerto Rico. It has historically been collected in Jamaica (see Remarks). It is also found in southern Florida (Map 12).

### Selenops marcanoi

Alayón-García, 1992

http://species-id.net/wiki/Selenops_marcanoi

Figs 123–126 Map 13

Selenops marcanoi Alayón-García 1992: 3, Figs 3a-3b (♀, not examined).

#### Type material.

Holotype female: El Aguacate, Neyba, Baoruco, Dominican Republic (IES), not examined.

#### Other material examined.

DOMINICAN REPUBLIC: Pedernales: El Banano, Rio Mulito, 18°09.165'N, 71°45.388'W, ~288 m, 25 XI.2004, S. Crews, under bark, SCC04_081, 1♀, 4♂, 1 imm. (EME sel_150-152, MNHN sel_153-154, CAS sel_155). **Barahona:** Carretera El Higuero-Polo, 18°00.251'N, 71°18.838'W, ~466 m, 26.XI.2004, S. Crews, under bark, SCC04_083, 3 imm. (CAS sel_169-170, 172).

#### Diagnosis.

Females can be separated from others by having only a lightly sclerotized median area, with a sinuous opening and large, round spermathecae (Figs 123–124). The male palpal organ is most similar to that of *Selenops insularis*, though the circular space formed by the conductor connections is more semi-circular, and the RTA is not as wide distally and is truncate (Figs 125–126).

#### Description.

*Male (sel_152):* Color:carapace yellow, slightly dusky laterally; sternum pale yellow; chelicerae dusky yellow; maxillae pale yellow; labium pale yellow; abdomen dorsally cream-colored background with many small spots, particularly medially, a median lanceolate stripe, with the chevrons below, with a single dot below this, festoon prominent; ventrally cream-colored, dark laterally, caudally; legs light yellow, darkening slightly distally, with faint annulations. Carapace: 0.89 times longer than broad; fovea longitudinal, broad, very shallow. Eyes:AER nearly straight; PER slightly recurved; PME larger than AME, PLE largest, ALE smallest; eye diameters, AME 0.10, ALE 0.09, PME 0.16, PLE 0.25; interdistances AME-PME 0.06, PME-ALE 0.14, ALE-PLE 0.33. PME-PME 1.00. ALE-ALE 1.60; ocular quadrangle AME-AME 0.35, PLE-PLE 1.60; clypeus 0.05 high. Mouthparts:chelicerae with stout setae medially and anteriorly; maxillae longer than broad, with tuft of conspicuous setae distally; labium distally rounded. Sternum:as long as broad, posteriorly indented. Legs:leg I only slightly shorter than II and III; leg formula 2314; scopulae present on all 4 tarsi and metatarsi and tibia I and II; tarsi I-IV with strong claw tufts; pr claw per foot slightly toothed; spination: leg I, Fm pr 1–1–1, d 1–1–1, rl 1–1–1; Ti pr 1–0–1, d 1–0–1, rl 1–0–1, v 2–2–2; Mt pr 1–1–0, v 2–2–0, rl 1–0–0; leg II, Fm pr 1–1–1, d 1–1–1, rl 1–1–1; Ti pr 1–0–1, d 1–0–1, rl 1–0–1, v 2–2–2; Mt pr 1–1–0, v 2–2, rl 1–0–0; leg III, Fm pr 1–1–1, d 1–1–1, rl 1–1–1; Ti pr 1–0–1, rl 1–0–1, v 2–2; Mt pr 1–1–0, rl 1–0–0, v 2–1; leg IV, Fm pr 1–1–1, d 1–1–1, rl 1–1–1; Ti pr 1–0–1, rl 1–0–0, v 1–1; Mt pr 1–1–0, rl 1–0–0, v 2–1. Abdomen:without terminal setal tufts. Pedipalp:Fm, spination d 0–1–4; cymbium oval in ventral view, slightly angled posterolaterally; scopulae scattered, denser toward tip; conductor hammer shaped to T-shaped, arising anteromedially on slightly curved stalk, reconnecting laterally, pointed laterally; embolus long and slender, beginning at 4 o'clock, terminating at 11 o'clock; MA narrow, sinuous, distally curved into small single hook, originating at 3–4 o'clock, directed distally; RTA extending 1/5 the length of cymbium in ventral view; with two apophyses, dorsal apophysis rectangular, only slightly curved, wider, truncate distally, ventral apophysis narrower, shorter (Figs 125–126). *Dimensions:* Total length 6.75. Carapace length 3.30, width 3.70. Sternum length 1.75, width 1.75. Abdomen length 3.45, width 2.85. Pedipalp: Fm 0.80, Pt 0.25, Ti 0.45, Ta 0.85, total 2.35. Leg I: Fm 4.00, Pt 1.75, Ti 4.00, Mt 3.75, Ta 1.60, total 15.10. Leg II: Fm 5.45, Pt 1.95, Ti 4.75, Mt 4.00, Ta 1.75, total 17.90. Leg III: Fm 5.00, Pt 1.50, Ti 4.75, Mt 4.00, Ta 1.60, total 16.85. Leg IV: Fm 4.50, Pt 1.00, Ti 3.75, Mt 3.50, Ta 1.25, total 14.00.

*Female (sel_151)*: Color:carapace orange-brown, duskier laterally, white setae on lateral margins and in cephalic area; sternum light yellow to orange-brown, darker around border; chelicerae brown; maxillae brown, lightening distally; labium brown, lightening distally; abdomen dorsally cream-colored background with many small spots, particularly medially, with the splotches below this forming a fanned chevron pattern, with one dot below this, dark laterally, festoon prominent; ventrally cream-colored, dark laterally and caudally; legs light yellow to brown darkening distally, annulations faint. Carapace: 0.81 times longer than broad; fovea longitudinal, broad, very shallow. Eyes:AER nearly straight; PER slightly recurved; PME larger than AME, PLE largest, ALE smallest; eye diameters, AME 0.26, ALE 0.08, PME 0.28, PLE 0.40; interdistances AME-PME 0.04, PME-ALE 0.15, ALE-PLE 0.26. PME-PME 1.13 mm. ALE-ALE 2.98; ocular quadrangle AME-AME 0.40, PLE-PLE 3.05; clypeus 0.05 high. Mouthparts:chelicerae with stout setae medially and anteriorly; maxillae longer than broad, with tuft of conspicuous setae distally; labium distally rounded. Sternum:as long as broad, posteriorly indented. Legs:leg I only slightly shorter than II and III; leg formula unknown (at least one leg missing), 2341 (Alayón-García 1992); scopulae present on all 4 tarsi and metatarsi of leg II and metatarsi and tibiae of leg I; tarsi I-IV with strong claw tufts; pr claw per foot slightly toothed; spination: leg I, Fm pr 1–1–0, d 1–1–1, rl 0–0–1; Ti d 0, v 2–2–2; Mt v 2–2; leg II, Fm pr 1–0–0, d 1–1–1, rl 0–0–1; Ti v 2–2–2; Mt v 2–2; leg III, Fm pr 1–0–0, d 1–1–1, rl 0; Ti v 1–1–0; Mt v 2–0. Abdomen:without terminal setal tufts. Pedipalp: claw with 7 teeth. Epigyne:anteromedian area less sclerotized than rest of epigynum, lateral lobes fused medially, sinuous projection medially, genital openings occur behind this, epigynal pockets present; internally, ducts lead to large roundish spermathecae, posterodorsal fold present, quadrangular, covering majority of internal ducts (Figs 123–124). *Dimensions:* Total length 8.00. Carapace length 3.45, width 4.25. Sternum length 1.88, width 1.88. Abdomen length 4.55, width 3.90. Pedipalp: Fm 1.00, Pt 0.30, Ti 1.50, Ta 1.00, total 3.80. Leg I: Fm 4.00, Pt 1.60, Ti 3.30, Mt 3.00, Ta 1.25, total 13.15. Leg II: Fm 5.00, Pt 1.75, Ti 4.40, Mt 3.00, Ta 1.00, total 15.15. Leg III: Fm 5.00, Pt 1.75, Ti 4.00, Mt 3.00, Ta 1.25, total 15.00. Leg IV: Missing.

#### Natural history.

This species has been collected under bark, in riparian areas and dry forests. Alayón-García (1992) also reported this species occurring under rocks in humid forests.

#### Distribution.

**Map 13.**
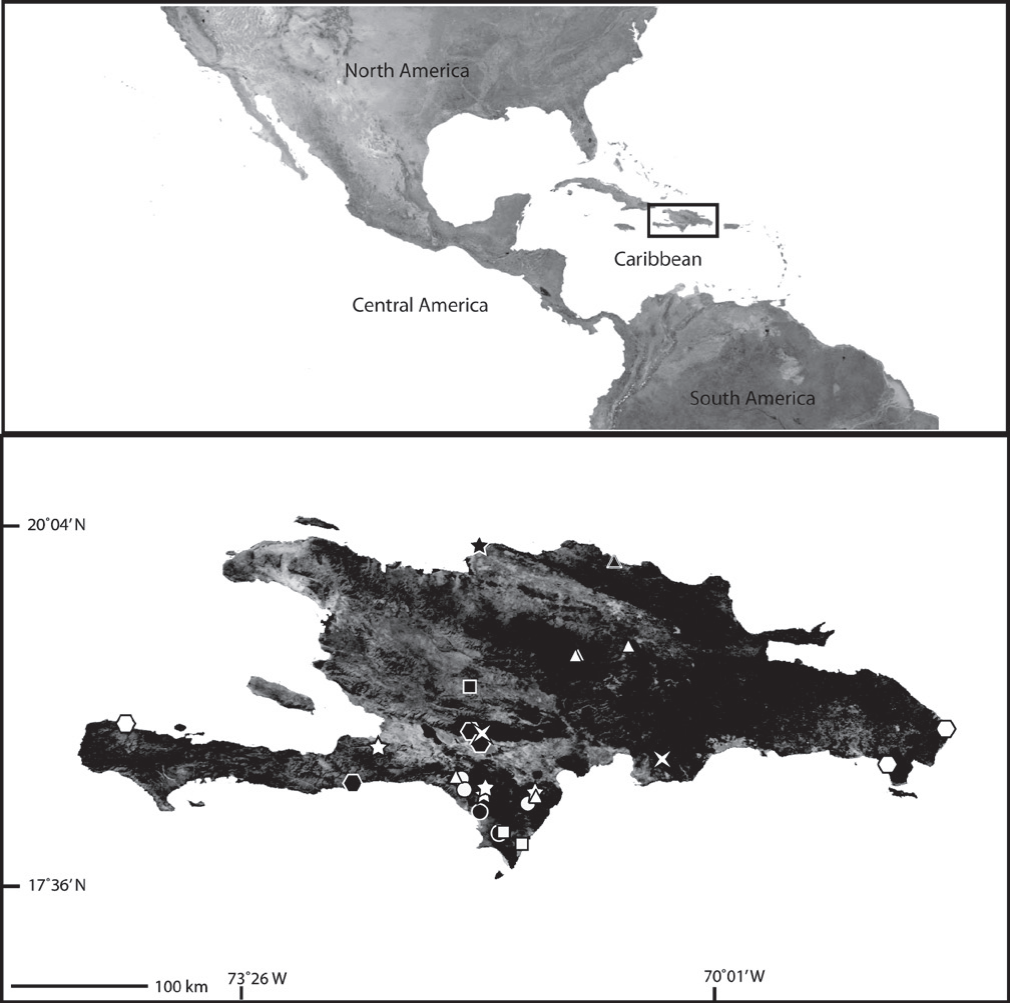
Map of Hispaniola (inset) showing the distribution of endemic *Selenops* species, *Selenops marcanoi* Alayón-García (white circles), *Selenops bocacanadensis* sp. n. (black circles), *Selenops oviedo* sp. n. (white squares), *Selenops duan* sp. n. (black square), *Selenops denia* sp. n. (white triangles), *Selenops guerrero* sp. n. (black triangle), *Selenops bani* Alayón-García (white Xs), *Selenops pensilis* Muma (white hexagons), *Selenops enriquillo* sp. n. (black hexagons), *Selenops phaselus* Muma (white stars), and *Selenops morro* sp. n. (black star).

Endemic to the Dominican Republic and seems to have a range restricted to the south of the country (Map 13).

### Selenops bocacanadensis sp. n.

urn:lsid:zoobank.org:act:B4BBF900-A850-45C5-BDE1-DF53B6D426EA

http://species-id.net/wiki/Selenops_bocacanadensis

Figs 127–128 Map 13

#### Type material.

Holotype female: from a rocky outcrop at night at kilometer 13 along Carretera ALCOA, Pedernales, Dominican Republic, 18°01.962' N, 71°38.748' W, 24XI.2004, S. Crews, SCC04_079 (EME sel_166).

#### Other material examined.

DOMINICAN REPUBLIC: same data as holotype, 6 imm. (EME sel_148-149, 161-163, 165, 167); Boca de la Cañada off Hwy. Pedernales-Oviedo, 17°55'00.5"N, 71°30'03.4"W, 9.X.2006, 64 m, S. Crews, on rocks at night, SCC06_071, 5 imm. (CAS sel_629-631, MNHNSD 635-636).

#### Etymology.

The specific epithet refers to a locality where the species is found, La Boca de la Cañada. It is to be treated as a noun in appostion.

#### Diagnosis.

The females of *Selenops bocacanadensis* sp. n. are similar to S. insularis in size and coloration, but females can be distinguished by the epigynal plate, as it is not as straight along the posterior margin, the genital opening is more rounded, rather than v-shaped, the posterodorsal fold is smaller, and the internal ducts are shaped differently (Figs 127–128). Males unknown.

#### Description.

*Holotype female:* Color: Carapace uniformly brownish-red with white setae; sternum light orange-brown; chelicerae uniformly dark reddish-brown; maxillae light orange-brown, darker on outer distal edge, white on inner distal edge; labium orange-brown, lightening distally; abdomen dorsally grey-tan, dark festoon pattern caudally; ventrally dusky grey with no markings; legs: femora red-brown with darker annulations, hardly visible, patella to tibia very dark (black in live specimens). Carapace:0.91 times longer than broad. Eyes:AER nearly straight; PER recurved; PME larger than AME, PLE largest, ALE smallest; eye diameters, AME 0.38, ALE 0.13, PME 0.48, PLE 0.55; interdistances AME-PME 0.10, PME-ALE 0.13, ALE-PLE 0.60. PME-PME 1.68 mm. ALE-ALE 2.85; ocular quadrangle AME-AME 0.60, PLE-PLE 2.98; clypeus 0.09 high. Mouthparts:chelicerae with stout setae medially and anteriorly; maxillae longer than broad, with tuft of conspicuous setae distally; labium distally rounded. Sternum:as long as broad, posteriorly indented. Legs:Leg I only slightly shorter than legs II, III and IV; leg formula 2341; legs I and II with tarsal and metatarsal scopulae; tarsi I-IV with strong claw tufts; claws without teeth; spination: leg I, Fm pr 2–1–0, d 1–1–1, rl 1–1–1; v 2–2–2; Mt v 2–2; leg II, Fm pr 1–0–0, d 1–1–1, rl 1–1–1; Ti v 2–2–2; Mt v 2–2; leg III, Fm pr 1–0–0, d 1–1–1, rl 1–1–1; Ti v 2–2–0; Mt v 2–2; leg IV, Fm pr 1–0–0, d 1–1–1, rl 0–0–1; Ti v 2–2–0; Mt v 2–2. Abdomen:without terminal setal tufts. Pedipalp:claw with 11 teeth. Epigyne:lateral lobes abut one another,¾ the length of plate, extending from caudal margin to bottom of genital opening; opening small, slightly ovoid, epigynal pockets present; caudal margin sinuous, lobes extend posteromedially; internally, sperm ducts extend posteriorly, then separate laterally to oblong, cylindrical spermathecae, the fertilization ducts located laterally, directed anterolaterally, posterodorsal fold small, sinuous, does not completely cover spermathecae (Figs 127–128). *Dimensions:* The abdomen was damaged during collection, however, the specimen falls on the larger end of the spectrum for this genus (>10.00). Carapace: length 5.25, width 5.75. Pedipalp: Fm 2.00, Pt 0.30, Ti 0.75, Ta 1.75, total 4.80. Leg I: Fm 6.00, Pt 2.00, Ti 6.00, Mt 4.25, Ta 2.00, total 20.25. Leg II: Fm 8.00, Pt 2.25, Ti 6.50, Mt 5.00, Ta 2.00, total 23.75. Leg III: Fm 7.75, Pt 2.00, Ti 6.00, Mt 5.00, Ta 2.00, total 22.75. Leg IV: Fm 6.50, Pt 2.00, Ti 5.75, Mt 5.75, Ta 2.00, total 22.00.

#### Natural History.

Collected at night on rock outcrops and on and under rocks in the dryer part of the island from 64 -140 m.

#### Distribution.

Endemic to Hispaniola; known only from the Pedernales Peninsula in the Dominican Republic (Map 13).

### Selenops oviedo sp. n.

urn:lsid:zoobank.org:act:F8E9BA14-3DFC-4A62-9007-72688028CCC0

http://species-id.net/wiki/Selenops_oviedo

**Figures 129–136.**
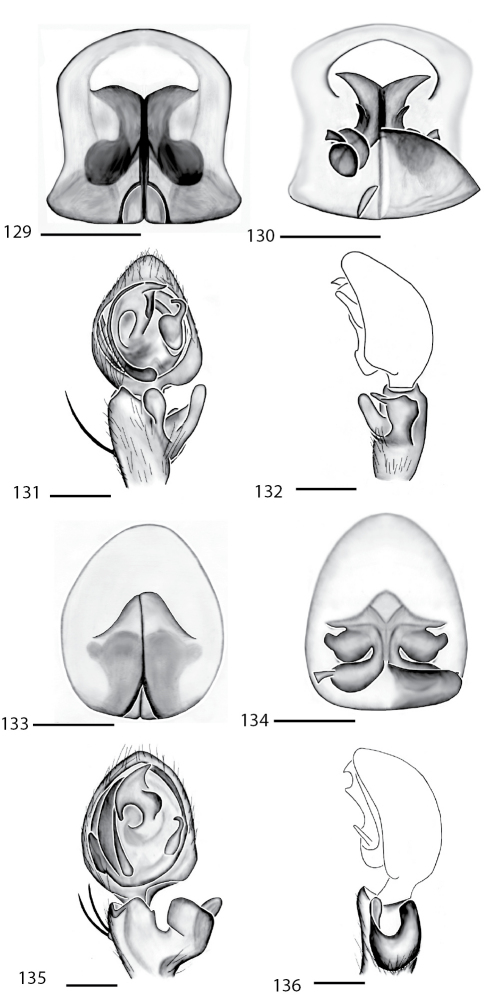
Copulatory organs of *Selenops oviedo* sp. n. holotype female from El Cajuil, Laguna Oviedo, Pedernales, Dominican Republic (EME sel_623) **129–130** *Selenops trifidus* Bryant holotype male from Navassa Island (MCZ-B0045) **131–132** *Selenops duan* sp. n. holotype female from Cañada Miguel, Comendador, Elias Piña, Dominican Republic (MNHNSD, A-515) **133–134** *Selenops denia* sp. n. holotype male from Mata Grande, Santiago, Dominican Republic (EME sel_641) **135–136, 129, 133, 135** epigyne, ventral view **130, 134, 136** spermathecae, dorsal view **131** left pedipalp, ventral view **132** left pedipalp, retrolateral view. Scale bar = 0.50 mm.

Figs 129–130 203 Map 13

#### Type material.

Holotype female: El Cajuil, Laguna Oviedo, Pedernales, Dominican Republic, 17°48'08.1"N, 71°31'45.6"W, ~86 m, 9.X.2006, S. Crews, under and on rocks in forest surrounding lake, SCC06_070 (EME sel_623).

#### Other material examined.

DOMINICAN REPUBLIC: Pedernales: same data as holotype, 1♀, 1p♂, 7 imm. (MNHNSD sel_619, 621-622, 624-629); Boca de la Cañada off Hwy. Pedernales-Oviedo, 17°55'00.5"N, 71°30'03.4"W, 9.X.2006, 64 m, S. Crews, on rocks at night, SCC06_071, 2 imm. (CAS sel_637-638).

#### Etymology.

This specific epithet comes from the name of the type locality and is to be treated as a noun in apposition.

#### Diagnosis.

Females can be separated from all others by the genital openings which are are located anteriorly, the epigynal pockets are large, and the rounded posterodorsal fold extends nearly half the length of the epigynal plate (Figs 129–130). Males unknown.

#### Description.

*Female holotype*: Color:carapace light orange-brown to yellow-brown, duskier laterally, darker in fovea, some white setae present; sternum light yellowish-brown, darker around border; chelicerae brown, lighter anteromedially, and orange caudally; maxillae light brown, lightening distally; labium brown, lightening distally; abdomen dorsally with a grey-yellow background, 4 pair of spots anteriorly to medially, medial spots fused, light grey chevrons at second pair of spots and again after the 4th pair, which are fused, a single dot below that, laterocaudal festoon present, angled; ventrally cream-colored, dark laterally, caudally; legs orange-brown, annulations present, legs darkening distally, brown at metatarsus and tarsus. Carapace: 0.87 times longer than broad; fovea longitudinal, broad, very shallow. Eyes:AER slightly recurved; PER slightly recurved; PME larger than AME, PLE largest, ALE smallest; eye diameters, AME 0.18, ALE 0.08, PME 0.28, PLE 0.40; interdistances AME-PME 0.03, PME-ALE 0.10, ALE-PLE 0.38. PME-PME 1.20. ALE-ALE 1.98; ocular quadrangle AME-AME 0.40, PLE-PLE 2.09; clypeus 0.13 high. Mouthparts:chelicerae with a few stout setae medially and anteriorly; maxillae longer than broad, with tuft of conspicuous setae distally; labium distally rounded. Sternum:1.06 times longer than broad, posteriorly indented. Legs:leg I only slightly shorter than legs II, III and IV; leg formula 2431; scopulae present on all 4 tarsi and metatarsi and tibiae I and II; tarsi I-IV with strong claw tufts; pr claw per foot slightly toothed; spination: leg I, Fm pr 1–1–0, d 1–1–1, rl 1–1–1; Ti d 0, v 2–2–2; Mt v 2–2; leg II, Fm pr 1–0–0, d 1–1–1, rl 1–1–1; Ti v 2–2–2; Mt v 2–2; leg III, Fm pr 1–0–0, d 1–1–1, rl 1–0–1; Ti v 2–2–0; Mt v 2–1; leg IV, Fm pr 1–0–0, d 1–1–1, rl 0–0–1; Ti v 2–2–0; Mt v 2–1. Abdomen:without terminal setal tufts. Pedipalp:claw with 8 teeth. Epigyne:anteromedially, plate less sclerotized anteriorly compared to rest of plate, sinuous margin located anteriorly, genital openings located behind this, lateral lobes fused medially, epigynal pockets present; internally, ducts directed posteriorly, touching anteriorly, then separating medially,directed laterally, rounded terminally, fertilization ducts located and directed laterally, posterodorsal fold semi-circular, extending nearly half length of epigynal plate (Figs 129–130). *Dimensions:* Total length 9.15. Carapace length 3.48, width 4.00. Sternum length 1.85, width 1.75. Abdomen length 5.68, width 3.70. Pedipalp: Fm 1.00, Pt 0.25, Ti 0.75, Ta 1.45, total 3.45. Leg I: Fm 3.75, Pt 1.75, Ti 3.30, Mt 3.00, Ta 1.00, total 12.80. Leg II: Fm 4.75, Pt 1.50 Ti 4.00, Mt 3.00, Ta 1.50, total 14.75. Leg III: Fm 4.50, Pt 1.50, Ti 3.65, Mt 3.00, Ta 1.00, total 13.65. Leg IV: Fm 4.50, Pt 1.00, Ti 4.00, Mt 3.00, Ta 1.35, total 13.85.

#### Natural history.

This species has been found under and on rocks, around the shores of a lake and in the forest (Fig. 203). The female guards the flat, white, disc-shaped egg sac. One female made an egg sac on 17.X.2006 and the eggs hatched 15.XI.2006. The mother died 21.XI.2006.

#### Distribution.

Endemic to the Dominican Republic and has a limited distribution on the southern Pedernales peninsula (Map 13).

### Selenops trifidus

Bryant, 1948

http://species-id.net/wiki/Selenops_trifidus

Figs 131–132 Map 12

Selenops trifidus Bryant 1948: 415, pl. 10, Figs 98, 100 (♂, examined).
Selenops trifidus :Muma 1953: 25, Figs 41–42 (♂).

#### Type material.

Holotype male: Navassa Island, 1–9.I.1930, Clench, Schevill, Rehder (MCZ-B0045).

#### Diagnosis.

Males can be easily distinguished from other species by the large base and distal hook of the MA, and the small process on the distal end of the dorsal branch of the RTA (Figs 131–132). Females unknown.

#### Remarks.

The type was apparently not in great shape when Muma (1953) viewed it 60 years ago. Since Muma's (1953) descriptions, it appears to be even more discolored.

#### Description.

*Male holotype:* Color:carapace orange-brown with no markings; sternum orange-brown; chelicerae orange-brown; maxillae orange-brown, lightening distally; labium orange-brown, lightening distally; abdomen dorsally brown-grey, markings indistinct due to shrivelling, laterocaudal festoon distinct; legs orange-brown. Carapace: 0.86 times longer than broad; fovea longitudinal, broad, very shallow. Eyes:AER nearly straight; PER recurved; AME slightly larger than PME, PLE largest, ALE smallest; eye diameters, AME 0.28, ALE 0.13, PME 0.20, PLE 0.45; interdistances AME-PME 0.03, PME-ALE 0.38, ALE-PLE 0.18. PME-PME 1.23. ALE-ALE 1.90; ocular quadrangle AME-AME 0.40, PLE-PLE 2.23; clypeus 0.91 high. Mouthparts:chelicerae with a few scattered setae medially and anteriorly; maxillae longer than broad, with tuft of conspicuous setae distally; labium distally rounded. Sternum:0.95 times longer than broad, posteriorly indented. Legs:leg I only slightly shorter than II and III; leg formula 2314; scopulae present on tarsi of all legs and metatarsi of legs I and II; tarsi I-IV with strong claw tufts; pr claw per foot slightly toothed; spination: leg I, Fm pr 1–1–1, d 1–1–1, rl 1–1–1; Ti d 1–1–0, pr 1–1–0, rl 1–1–0, v 2–2–2–2; Mt pr 1–1–0, v 2–2–0, rl 1–0–0; leg II, Fm pr 1–1–1, d 1–1–1, rl 1–1–1; Ti pr 1–1–0, rl 1–1–0, d 1–1–0, v 2–2–2–2; Mt pr 1–1–0, v 2–2, rl 1–1–0; leg III, Fm pr 1–1–1, d 1–1–1, rl 1–1–0; Ti pr 1–0–1, d 0–1–0, rl 1–0–1, v 2–2–1–2 (2–2–2 on R side); Mt pr 1–1–1, rl 1–0–0, v 2–2; leg IV, Fm pr 1–1–1, d 1–1–1, rl 1–1–1; Ti pr 1–0–1, rl 1–0–1, d 1–1–0, v 2–2; Mt pr 1–1–0, rl 1–0–0, v 2–2–0. Abdomen:without terminal setal tufts. Pedipalp:Fm, spination d 0–1–3;cymbium triangular in ventral view, angled slightly posterolaterally; conductor large, arising from short straight stalk anteromedially, pointed laterally, reconnecting laterally, forming oblong space between two conductor connections; embolus long, slender, curved, not quite at edge of cymbium, beginning at 6 o'clock, terminating at 12 o'clock; MA with very large oval base, narrowing abruptly into long hook, arising from 2–4 o'clock, directed distally; RTA with two apophyses, ventral process shorter, narrower than lateral process, curves away from cymbium, rounded distally; lateral process large, curves away from cymbium, widens distally, truncate to sinuous terminally, with pointed process directed ventrally; RTA barely reaching cymbium in ventral view (Figs 131–132). *Dimensions:* Total length 6.45. Carapace length 3.90, width 4.53. Sternum length 1.75, width 1.85. Abdomen length 2.58, width 2.40. Pedipalp: Fm 1.45, Pt 0.40, Ti 0.75, Ta 1.25, total 3.85. Leg I: Fm 5.00, Pt 2.00, Ti 5.50, Mt 5.50 Ta 2.50, total 20.50. Leg II: Fm 5.75, Pt 2.00, Ti 6.00, Mt 6.00, Ta 2.50, total 22.25. Leg III: Fm 6.00, Pt 2.00, Ti 5.50, Mt 5.50, Ta 2.00, total 21.00. Leg IV: Fm 5.75, Pt 1.75, Ti 5.00, Mt 5.00, Ta 2.00, total 19.50.

#### Natural history.

No data.

#### Distribution.

Known only from the type locality, and thus appears to be endemic to the mostly uninhabited, politically contested Navassa Island (Map 12).

### Selenops duan sp. n.

urn:lsid:zoobank.org:act:1DF5E3C7-51AE-4D53-883D-85D5670E65E6

http://species-id.net/wiki/Selenops_duan

Figs 133–134 Map 13

#### Type material.

Holotype female: Cañada Miguel, Comendador, Elias Piña, Dominican Republic, 17.XII.1979, F. Marcano, (MNHNSD, A-515).

#### Etymology.

The species name comes from the indigenous Taíno word for the region, Duan, and is to be treated as a noun in apposition.

#### Diagnosis.

Females can be separated from all other species by the lateral lobes which come together medially and form an anterior rounded projection that the genital openings are located behind (Figs 133–134). Males unknown.

#### Description.

*Female holotype*: Color:carapace orange-brown; sternum orange- brown, darker around border; chelicerae uniformly brownish-red; maxillae light orange-brown lightening distally; labium orange-brown, lightening toward the distal edge; abdomen dorsally cream-colored, faded, remnants of lanceolate stripe, including pale spots, particularly in center, festoon visible; ventrally cream-colored; legs orange to orange-brown, annulations faint but present, probably faded. Carapace: 0.89 times longer than broad; fovea longitudinal, broad, very shallow. Eyes:AER nearly straight; PER slightly recurved; PME larger than AME, PLE largest, ALE smallest; eye diameters, AME 0.28, ALE 0.10, PME 0.40, PLE 0.48; interdistances AME-PME 0.10, PME-ALE 0.15, ALE-PLE 0.48. PME-PME 1.63. ALE-ALE 2.70; ocular quadrangle AME-AME 0.58, PLE-PLE 2.68; clypeus 0.09 high. Mouthparts:chelicerae with stout setae medially and anteriorly; maxillae longer than broad, with tuft of conspicuous setae distally; labium distally rounded. Sternum:1.10 times longer than broad, posteriorly indented. Legs:leg I only slightly shorter than leg II; leg formula 2134; scopulae present on all 4 tarsi and metatarsi and tibiae I and II; tarsi I-IV with strong claw tufts; pr claw per foot slightly toothed; spination: leg I, Fm pr 1–1–0, d 1–1–1, rl 1–1–1; Ti d 0, v 2–2–2; Mt v 2–2; leg II, Fm pr 1–1–0, d 1–1–1, rl 1–1–1; Ti v 2–2–2; Mt v 2–2; leg III, Fm pr 1–1–0, d 1–1–1, rl 0–0–1; Ti v 2–2–0; Mt v 2–1; leg IV, Fm pr 1–1–0, d 1–1–1, rl 0–1–1; Ti v 2–2–0; Mt v 2–1. Abdomen:without terminal setal tufts. Pedipalp:claw with 13 teeth. Epigyne:lateral lobes fused asymmetrically, anteriorly forming rounded projection, genital openings located laterally behind projection, epigynal pockets present; internally, ducts connect to oval to round spermathecae, anterior most with lateral, small quadrangular projections, fertilization ducts located posteriorly, directed laterally, posterodorsal fold present, covering posterior spermathecae (Figs 133–134). *Dimensions:* Total length 11.83. Carapace length 5.08, width 5.68. Sternum length 2.75, width 2.50. Abdomen length 6.75, width 4.80. Pedipalp: Fm 1.50, Pt 1.00, Ti 1.00, Ta 1.70, total 5.20. Leg I: Fm 5.75, Pt 2.75, Ti 5.00, Mt 4.00, Ta 1.85, total 19.35. Leg II: Fm 6.75, Pt 2.40, Ti 5.50, Mt 4.35, Ta 2.00, total 21.00. Leg III: Fm 6.00, Pt 1.75, Ti 4.75, Mt 4.60, Ta 1.75, total 18.85. Leg IV: Fm 5.75, Pt 1.90, Ti 4.75, Mt 4.00, Ta 1.75, total 18.15.

#### Natural history.

No data.

#### Distribution.

Known only from the type locality, the center of Hispaniola, and is apparently endemic to the island (Map 13).

### Selenops denia sp. n.

urn:lsid:zoobank.org:act:199DF889-C082-40AD-BFE0-98DF792B2F07

http://species-id.net/wiki/Selenops_denia

Figs 135–136 Map 13

#### Type material.

Holotype male: Mata Grande, Santiago, Dominican Republic, 19°11'43.0"N, 70°59'42.0"W, 14-15.X.2006, 1009 m, S. Crews, on buildings, trees, fence posts at night, SCC06_075 (EME sel_641).

#### Other material examined.

DOMINICAN REPUBLIC: Santiago: same data as holotype, 1♂, 1p♂, 4 imm. (MNHNSD sel_640, 642-646); Parque Nacional Armando Bermudéz, trailhead to Loma del Oro, 19°12'05.2"N, 71°00'04.8"W, 13.X.2006, 1035 m, S. Crews, on ranger station at night, SCC06_074, 1 imm. (EME sel_639); **Barahona:** Polo Coffee Plantation, 18°06.275'N, 71°15.487'W, 17.VI.2006, 970 m, L. Mahler, on banana tree under a dead frond on trunk, 1♂ (CAS sel_568). **La Vega:** 10 km NE Jarabacoa, Hotel Montana, 18.VII-4.VIII.1995, 550 m, S., J. Peck, forest, (AMNH). **Pedernales:** road to Aguacate from Río Mulito, 18°13.895'N, 71°45.190'W, 25.XI.2004, S. Crews, under bark of dead tree stump, dry forest, though most of the trees recently chopped down, lots of egg sacs, SCC04_082, 1 imm. (EME sel_156).

#### Etymology.

This species is named in honor of Denia Veloz for all of her work on arthropod biodiversity in the Dominican Republic. It is to be treated as a noun in apposition.

#### Diagnosis.

Males can be separated from all other species by the conductor which arises anteromedially on a short stalk with a rounded projection. The conductor is directed opposite the RTA and is hammer shaped (Figs 135–136). Females unknown.

#### Description.

*Holotype male*: Color: carapace brownish-orange; sternum, brownish-yellow, darker around border; chelicerae dark red-brown; maxillae light orange-brown, lightening distally; labium orange-brown, lightening toward distally edge; abdomen dorsally brownish-orange, dark at anterior margin, lanceolate stripe extending nearly 3/4th the length of abdomen, line extending across entire abdomen below stripe, laterocaudal festoon present; ventrally yellow-grey, darker laterally; legs yellowish-brown to orange-brown, darkening slightly distally, annulations conspicuous on femora, patellae and tibiae. Carapace: as long as broad; fovea, longitudinal, narrow, shallow. Eye: AER nearly straight; PER slightly recurved; PME the same size as AME, PLE largest, ALE smallest; eye diameters, AME 0.40, ALE 0.23, PME 0.40, PLE 0.55; interdistances AME-PME 0.10, PME-ALE 0.20, ALE-PLE0.40, PME-PME 1.15, ALE-ALE 2.30; ocular quadrangle AME-AME 0.20, PLE-PLE 2.35; clypeus 0.10 high; chillum absent. Mouthparts: chelicerae with stout setae medially and anteriorly; maxillae longer than broad with tuft of conspicuous setae distally; labium distally rounded. Sternum: as long as broad, posteriorly indented. Legs: leg I much shorter than leg II and slightly shorter than leg III; leg formula 2314; scopulae present on all 4 tarsi; tarsi I-IV with strong claw tufts; pr claw per foot slightly toothed; spination: leg I, Fm pr 1-1-0, d 1-1-1, rl 1-1-1; Ti d 0, v 2-2-2; Mt v 2-2; leg II, Fm pr 1-1-1, d 1-1-1, rl 1-1-1; Ti v 2-2-2; Mt v 2-2; leg III, Fm pr 1-1-0, d 1-1-1, rl 0-1-1; Ti v 2-2; Mt v 2-2; leg IV, Fm pr 1-1-0, d 1-1-1, rl 0-0-1; Ti v 1-1; Mt v 2-1. Abdomen: without terminal setal tufts. Pedipalp: Fm spination 0-1-3; cymbium oval in ventral view, slightly angled posterolaterally; basal cymbial process absent, scopulae scattered, denser distall; conductor arising anterolaterally on short stalk with rounded lateral projection, hammer shaped, directed opposite RTA, forming circular space between hammer shape and stalk, embolus shorter, slender, directed anteriorly, straight, arising at 6 o'clock, terminating at 11 o'clock; MA wider at base, narrowing, curving into small hook, originating at 4 o'clock, directed anterolaterally; RTA barely reaching cymbium in ventral view; posterior branch directed anteriorly, quadrangular, truncate terminally; ventral branch quadrangular in ventral view, flattened in lateral view; RTA looks u-shaped in lateral view (Figs 135–136). *Dimensions:* Total length 9.50. Carapace length 5.00, width 5.00. Sternum length 2.00, width 2.00. Abdomen length 4.50, width 4.00. Pedipalp: Fm 1.75, Pt 1.00, Ti 1.00, Ta 1.30, total 5.05. Leg I: Fm 7.00, Pt 2.00, Ti 6.00, Mt 5.00, Ta 2.50, total 22.50. Leg II: Fm 7.00, Pt 2.50, Ti 6.75, Mt 6.00, Ta 2.75, total 25.00. Leg III: Fm 7.00, Pt 2.00, Ti 6.00, Mt 5.50, Ta 2.50, total 23.00. Leg IV: Fm 6.00, Pt 2.00, Ti 5.00, Mt 5.00, Ta 2.00, total 20.00.

#### Natural history.

This species has been collected under bark and on buildings and fence posts at night, primarily at higher elevations (> 550 m).

#### Distribution.

Endemic to the Dominican Republic, though seems to be widespread across the country (Map 13).

### Selenops guerrero sp. n.

urn:lsid:zoobank.org:act:58C40A19-89A8-42DE-B775-A41A67366547

http://species-id.net/wiki/Selenops_guerrero

Figs 137–138 Map 13

#### Type material.

Holotype female: ranger's station at entrance to Loma de Isabel, Puerto Plata, Dominican Republic, 19°46'41.9"N, 70°42'01.1"W, 7.X.2006, ~96 m, on S. Crews, trees at night, under bark, SCC06_068 (EME sel_606).

#### Other material examined.

DOMINICAN REPUBLIC: Puerto Plata: same data as holotype, 2♀, 3. imm. (MNHNSD sel_598, CAS sel_600-601, 603-605).

#### Etymology.

This species is named in honor of Kelvin Guerrero for all of his work on the biodiversity of arthropods of the Dominican Republic, and is to be treated as a noun in apposition.

#### Diagnosis.

**Figures 137–144.**
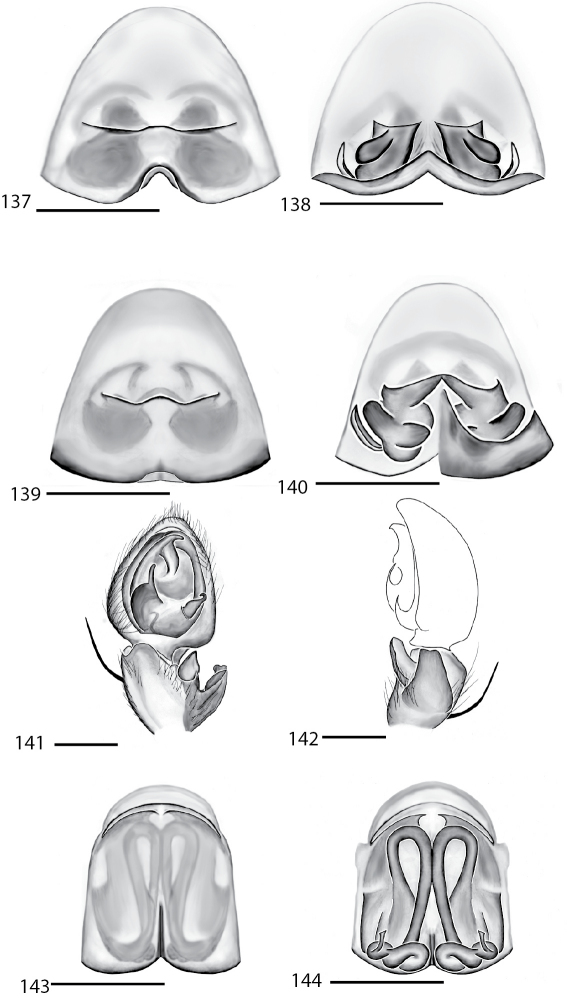
Copulatory organs of *Selenops guerrero* sp. n. holotype female from entrance to Loma de Isabel, Puerto Plata, Dominican Republic (EME sel_606) **137–138** *Selenops baweka* sp. n. holotype female from Garden Pond Field Road, Middle Caicos, Turks and Caicos Islands (EME sel_678) **139–140** male from Wade's Green Plantation, North Caicos, Turks and Caicos Islands (EME sel_675) **141–142** *Selenops kalinago* sp. n. holotype female from East of Veranda Resort, Indian Town, Antigua (EME sel_753) **143–144, 137, 139, 143** epigyne, ventral view **138, 140, 144** spermathecae, dorsal view **141** left pedipalp, ventral view **142** left pedipalp, dorsal view. Scale bar = 0.50 mm.

This species is most similar to *Selenops baweka* sp. n**.**, but can be separated having the genital opening nearly straight, differently shaped spermathecae, and a very small posterodorsal fold (Figs 137–138). Males unknown.

#### Description.

*Holotype female:* Color: carapace reddish-brown, darker laterally, sternum orange-brown, darker around border; chelicerae dark red-brown; maxillae orange brown, lightening distally; labium orange-brown, lightening toward the distal edge; abdomen dorsally greyish-tan with darker splotches and spots, laterocaudal festoon present; ventrally cream-colored, dark laterally; legs yellow-brown, darkening distally, annulations conspicuous on femora, patellae and tibiae. Carapace: 0.90 times longer than broad; fovea longitudinal, narrow, shallow. Eyes: AER nearly straight; PER slightly recurved; PME larger than AME, PLE largest, ALE smallest; eye diameters, AME 0.31, ALE 0.17, PME 0.33, PLE 0.46; interdistances AME-PME 0.08, PME-ALE 0.21, ALE-PLE 0.50, PME-PME 1.13, ALE-ALE 2.04; ocular quadrangle AME-AME 0.25, PLE-PLE 2.29; clypeus 0.08. Mouthparts: chelicerae with stout setae medially and anteriorly; maxillae longer than broad, with tuft of conspicuous setae distally; labium distally rounded. Sternum 1.11 times longer than broad, posteriorly indented. Legs: leg I shorter than II, III, and IV; leg formula 2341; scopulae present on all 4 tarsi and metatarsi and tibiae I and II; tarsi I-IV with strong claw tufts; pr claw per foot slightly toothed; spination: leg I, Fm pr 1–1–0, d 1–1–1, rl 1–0–1; Ti d 0, v 2–2–2; Mt v 2–2; leg II, Fm pr 1–0–0, d 1–1–1, rl 1–0–1; Ti v 2–2–2; Mt v 2–2; leg III, Fm pr 1–0–0, d 1–1–1, rl 1–0–1; Ti v 2–2–0; Mt v 2–2; leg IV, Fm pr 1–1–0, d 1–1–1, rl 0–0–1; Ti v 2–2–0; Mt v 2–2. Abdomen: with terminal setal tufts. Pedipalp: claw with 11 teeth. Epigyne: lateral lobes indistinct, nearly straight line across center of epigynal plate, genital openings located medially behind this slit, epigynal pockets present; internally, ducts wide, spaced widely, with smaller, more oblong projections, fertilization ducts located posteriorly, directed anterolaterally, small posterodorsal fold present, barely covering internal ducts (Figs 137–138).

*Dimensions:* Total length 10.40. Carapace length 5.20, width 5.80. Sternum length 2.00, width 1.80. Abdomen length 5.20, width 3.80. Pedipalp: Fm 1.50, Pt 1.00, Ti 1.00, Ta 1.25, total 4.75. Leg I: Fm 5.00, Pt 2.00, Ti 4.00, Mt 3.00, Ta 1.50, total 15.50. Leg II: Fm 6.00, Pt 2.00, Ti 5.00, Mt 4.00, Ta 1.75, total 18.75. Leg III: Fm 5.50, Pt 2.00, Ti 4.25, Mt 4.00, Ta 2.00, total 17.25. Leg IV: Fm 5.00, Pt 1.50, Ti 4.00, Mt 4.00, Ta 1.75, total 16.25.

#### Natural History.

This species has been collected on tree trunks at night and under bark.

#### Distribution.

Endemic to the Dominican Republic and known only from the type locality (Map 13).

### Selenops baweka sp. n.

urn:lsid:zoobank.org:act:1BACC7EC-E2D2-4BED-B10D-776523AA7EAB

http://species-id.net/wiki/Selenops_baweka

Figs 139–142 190 Map 10

#### Type material.

Holotype female: Garden Pond Field Road, Middle Caicos, Turks and Caicos Islands, 2414000 E, 19221000 N, 3.II.2007, S. Crews, B. Manco, high scrub, SCC07_004 (EME sel_678). Paratypes: Male, Turks and Caicos Islands, North Caicos, Wade's Green Plantation, 18808000 E, 2427000 N, 2.II.2007, S. Crews, B. Manco, tropical dry forest, SCC07_001 (EME sel _675).

#### Other material examined.

TURKS AND CAICOS ISLANDS: Providenciales: The Bight, 21°47'00.6"N, 72°13'06.4"W, ~2 m, 22.V.2006, S. Crews, under bark of sea grape, SCC06_013, 1♂, 1p♂ (CAS sel_344, 345); Turtle Cove, Third Turtle Drive, 21°47'01.1"N, 72°13'45.4"W, 22.V.2006, S. Crews, M. Jones, under bark of *Psidium* and wild tamarind, SCC06_017, 1♂, 3 imm. (EME sel_346-349); Turtle Cove, Third Turtle Drive, 21°47'01.1"N, 72°13'45.4"W, 10.II.2007, S. Crews, M. Jones, on tamarind and *Agave*, SCC07_043, 2 imm. (EME sel_694-695); vic. Northwest Point Pond Nature Reserve, 21°50'32.1"N, 72°19'43.7"W, ~9 m, 8.II.2007, S. Crews, under bark of poisonwood, SCC07_010, 4 imm. (EME sel_689-692); South View Drive off Leeward Hwy., 21°46'45.7"N, 72°13'45.4"W, 10.II.2007, S. Crews, M. Jones, under bark of sea grape, SCC07_012, 1♀ (CAS sel_693). **Middle Caicos:** same data as holotype, 1♂, 2 imm. (CAS sel_676-677, 679). **North Caicos:** same data as paratype, 1♀, 2♂, 1p♂, 4 imm. (EME sel_680-688).

#### Etymology.

The specific epithet comes from the name the indigenous people of the Turks and Caicos Islands used to refer to the Caicos Bank, Baweka, where this species is endemic. It is to be treated as a noun in apposition.

#### Diagnosis.

This species is similar to *Selenops guerrero* sp. n., but females can be differentiated by having the genital openings located behind a slightly sinuous margin, and internally, the ducts are in contact anteriorly, and the posterodorsal fold is large particularly medially (Figs 139–140). Males can be separated by the very short embolus and the MA which arises at 4 o'clock (Figs 141–142).

#### Description.

*Paratype Male:* Color:carapace light orange-brown to yellow-brown, duskier on the edges, darker in fovea, some white setae; sternum pale yellow, darker around border; chelicerae brown-darker medially; maxillae brown-yellow, lighter distally; labium dusky yellow, lightening distally; abdomen dorsally yellow-grey to brown, lanceolate medial stripe, hollow in center, sometimes disconnected forming pairs of dots, 2 lateral horizontal lines, not connecting in center, located caudally, laterocaudal festoon present; ventrally yellowish, dark laterally, caudally; legs yellowish-brown to tan, lighter than cephalothorax, with dark annulations, darker distally, metatarsus and tarsus brown. Carapace: 0.91 times longer than broad; fovea longitudinal, broad, very shallow. Eyes:AER nearly straight; PER recurved; PME larger than AME, PLE largest, ALE smallest; eye diameters, AME 0.23, ALE 0.05, PME 0.30, PLE 0.38; interdistances AME-PME 0.03, PME-ALE 0.20, ALE-PLE 0.38. PME-PME 1.13. ALE-ALE 1.88; ocular quadrangle AME-AME 0.48, PLE-PLE 2.00; clypeus 0.56 high. Mouthparts:chelicerae with a few scattered setae medially and anteriorly; maxillae longer than broad, with tuft of conspicuous setae distally; labium distally rounded. Sternum:1.14 times longer than broad, posteriorly indented. Legs:leg I only slightly shorter than legs II, III and IV; leg formula 2314; scopulae present on distal end of all 4 tarsi; tarsi I-IV with strong claw tufts; pr claw per foot slightly toothed; spination: leg I, Fm pr 1–1–1, d 1–1–1, rl 1–1–1; Ti pr 1–0–1, rl 1–0–1, d 1–1–0, v 2–2–2; Mt rl 1–0–0, ventral 2–2; leg II, Fm pr 1–1–1, d 1–1–1, rl 1–1–1; Ti pr 1–0–1, d 1–1–0, rl 1–0–1, v 2–2–2; Mt pr 1–0–0, v 2–2, rl 1–0–0; leg III, Fm pr 1–1–1, d 1–1–1, rl 1–1–1; Ti pr 1–0–1, d 0–1–0, rl 1–0–1, v 2–2; Mt rl 1–0–0, v 2–2; leg IV, Fm pr 1–1–0, d 1–1–1, rl 0–1–1; Ti pr 1–0–1, rl 1–0–0, v 2–2; Mt pr 0–1–0, rl 1–0–0, v 2–1. Abdomen:without terminal setal tufts. Pedipalp:Fm, spination d 0–1–4; cymbium oval to round in ventral view, angled posterolaterally; scopulae scattered, denser distally; conductor slightly hammer shaped, arising anteromedially on short slightly sinuate stalk, coming to point, other side curving around,reconnected to side of bulb forming semicircular space; embolus very short, arising from lateral margin of rounded projection, slightly curved, arising at 9 o'clock, terminating at 10 o'clock; MA wider basally, narrowing distally, curved into hook distally; MA arising at 4 o'clock, directed anterolaterally; RTA barely reaching cymbium in ventral view, with 2 processes, lateral process basally wide, narrowing distally, truncate at tip; ventral process curving away from tibia, slightly bent, rounded at tip in ventral view (Figs 141–142). *Dimensions:* Total length 7.65. Carapace length 4.00, width 4.40. Sternum length 2.00, width 1.75. Abdomen length 3.65, width 3.33. Pedipalp: Fm 1.00, Pt 0.25, Ti 0.75, Ta 1.00, total 3.00. Leg I: Fm 5.00, Pt 1.75, Ti 5.00, Mt 5.00, Ta 2.00, total 18.75. Leg II: Fm 6.35, Pt 2.00, Ti 5.50, Mt 5.25, Ta 2.00, total 21.10. Leg III: Fm 6.00, Pt 1.75, Ti 5.00, Mt 5.00, Ta 1.80, total 19.55. Leg IV: Fm 6.00, Pt 1.00, Ti 4.75, Mt 5.00, Ta 1.50, total 18.25.

*Holotype female:* Color:carapace light orange-brown to yellow-brown, duskier on the edges, darker in fovea, some white setae; sternum orange-brown, darker around border; chelicerae uniformly brownish-red; maxillae light orange-brown lightening distally; labium orange-brown, lightening toward the distal edge; abdomen dorsally yellow-grey to brown, lanceolate medial stripe, hollow in center, sometimes disconnected forming pairs of dots, 2 horizontal lines, laterally, not connecting medially, located caudally, laterocaudal festoon present; ventrally yellowish, dark laterally and caudally; legs orange-brown, darkening distally, annulations present, dark splotches on anterolateral face of femora making long stripe. Carapace: 0.88 times longer than broad; fovea longitudinal, broad, very shallow. Eyes:AER nearly straight; PER slightly recurved; PME larger than AME, PLE largest, ALE smallest; eye diameters, AME 0.20, ALE 0.10, PME 0.35, PLE 0.38; interdistances AME-PME 0.08, PME-ALE 0.10, ALE-PLE 0.36. PME-PME 1.13. ALE-ALE 1.95; ocular quadrangle AME-AME 0.40, PLE-PLE 2.20; clypeus 0.07 high. Mouthparts:chelicerae with a few scattered setae medially and anteriorly; maxillae longer than broad, with tuft of conspicuous setae distally; labium distally rounded. Sternum:1.06 times longer than broad, posteriorly indented. Legs:leg I only slightly shorter than legs II, III and IV; leg formula 2341; scopulae present on all 4 tarsi and metatarsi and tibiae I and II; tarsi I-IV with strong claw tufts; pr claw per foot slightly toothed; spination: leg I, Fm pr 1–1–0, d 1–1–1, rl 1–0–1; Ti d 0, v 2–2–2; Mt v 2–2; leg II, Fm pr 1–0–0, d 1–1–1, rl 1–0–1; Ti v 2–2–2; Mt v 2–2; leg III, Fm pr 1–0–0, d 1–1–1, rl 1–0–1; Ti v 2–2–0; Mt v 2–2; leg IV, Fm pr 1–1–0, d 1–1–1, rl 0–0–1; Ti v 2–2–0; Mt v 2–2. Abdomen:with terminal setal tufts. Pedipalp:claw with 11 teeth. Epigyne:lateral lobes absent, small opening located medially, slightly sinuous, openings located behind this slit, small epigynal pockets present; internally, lateral ducts wide, in contact anteriorly, then separate medially to posteriorly; fertilization ducts located posterolaterally, directed anterolaterally, posterodorsal fold present, covering 1/4th of the internal ducts laterally, extending anteriorly to a point medially (Figs 139–140). *Dimensions:* Total length 8.80 Carapace length 3.70, width 4.20. Sternum length 1.85, width 1.75. Abdomen length 5.10, width 4.00. Pedipalp: Fm 1.00, Pt 1.00, Ti 0.35, Ta 0.60, total 2.95. Leg I: Fm 4.00, Pt 1.80, Ti 4.00, Mt 3.00, Ta 1.00, total 13.80. Leg II: Fm 4.75, Pt 1.75, Ti 4.00, Mt 3.50, Ta 1.70, total 15.70. Leg III: Fm 4.75, Pt 1.50, Ti 4.00, Mt 3.50, Ta 1.25, total 15.00. Leg IV: Fm 4.75, Pt 1.40, Ti 3.75, Mt 3.00, Ta 1.00, total 13.90.

#### Natural History.

This species has been found under bark and on trees at night in scrublands and tropical dry forests (Fig. 190). An adult female was observed eating an adult male.

#### Distribution.

Endemic to the Caicos Bank (Map 10).

### Selenops kalinago sp. n.

urn:lsid:zoobank.org:act:D0F74CAB-9120-45F5-82F0-A6FBD3462B91

http://species-id.net/wiki/Selenops_kalinago

**Map 14.**
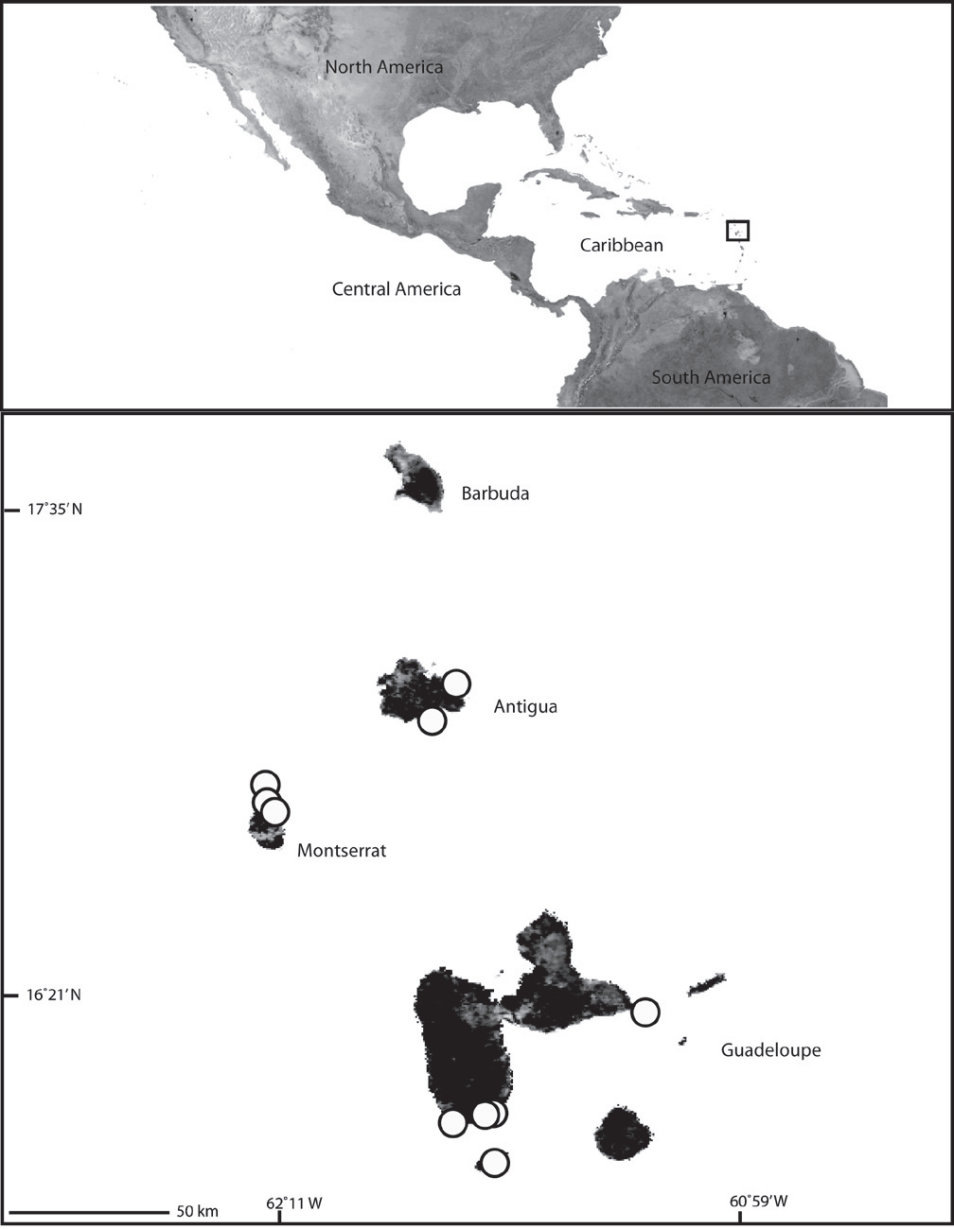
Map of the northern Lesser Antilles (inset) showing the distribution of *Selenops kalinago* sp. n. (white circles).

Figs 143 146 191 204 Map 14

#### Type material.

Holotype female: East of Veranda Resort, Indian Town, Antigua, 17°05'50.2"N, 61°40'53.0"W, 27.II.2007, ~15 m, S. Crews, A. Thibou, under *Bursera* bark (EME sel_753). Paratypes: Male from north side of Silver Hills, Montserrat, 16°48'41.3"N, 62°11'28.7"W, ~375 m, 3.III.2007, S. Crews, C. Fenton, under bark of cinnamon bay trees (EME sel_772).

#### Other material examined.

ANTIGUA:
**Shirley's Heights Lookout:** Nelson's Dockyard Ntl. Park, 17°00'06.7"N, 61°44'57.6"W, 27.II.2007, S. Crews, A. Thibou, under bark of *Bursera*, SCC07_034, 1♀, 1♂, 2 imm. (CAS sel_754–757). **GUADELOUPE: Basse-Terre:** Parc Archélogique des Roches Gravées near Trois Rivieres, 15°58.394'N, 61°38.347'W, 10.XI.2004, S. Crews, under bark of *Psidium guava*, SCC04_064, 1 imm. (EME sel_114); near Vieux Fort on the D6, stop along road at Forêt Domaniale du Littoral, 15°57.943'N, 61°42.517'W, 11.XI.2004, ~27 m, S. Crews, under bark of *Bursera*, SCC04_065, 1 imm. (EME sel_115); Trois-Rivieres, end of Sentier de l'Acomat trail off Rue Nelson Mandela, 15°58'03.0"N, 61°37'50.1"W, ~24 m, 5.III.2007, S. Crews, under bark of sea grape, SCC07_038, 1 ♀, 1 p♀, 2 imm. (EME sel_778-781). **Grande-Terre:** Pointe du Chateaux past St. Francois, 16°14'57.6"N, 61°11'02.6"W, sea level, 7.III.2007, S. Crews, under bark in dry coastal forest, SCC07_040, 1♀, 2♂, 2 imm. (CAS sel_786-790). **Les Saintes:** Terre-de-Haut, top of Le Chameaux, 15°51'28.1" N, 61°35'39.8" W, ~296 m, 6.III.2007, S. Crews, under bark of *Bursera*, Campeche, SCC07_039, 3 imm. (EME sel_783-785). **MONTSERRAT:** same data as paratype, 1♂, 1 p♂, 1♀, 3 imm. (CAS sel_771, 773-777). **Jack Boy Hill:** 16°46'02.1"N, 62°10'17.0"W, 200 m, 2.II.2007, S. Crews, J. Daley, under bark of guava and bay in dry forest, SCC07_035, 2♂, 1p♂, 4♀, 4 imm. (EME sel_758-768). **Sweet Water Ghaut:** 16°47'07.2"N, 62°10'59.8"W, 2.III.2007, S. Crews & J. Daley, under bark, SCC07_036, (CAS sel_769).

#### Etymology.

The specific epithet refers to the indigenous name of the indigenous people that once inhabited several Caribbean islands, including the ones where this species is found. It is to be treated as a noun in apposition.

#### Diagnosis.

**Figures 145–152.**
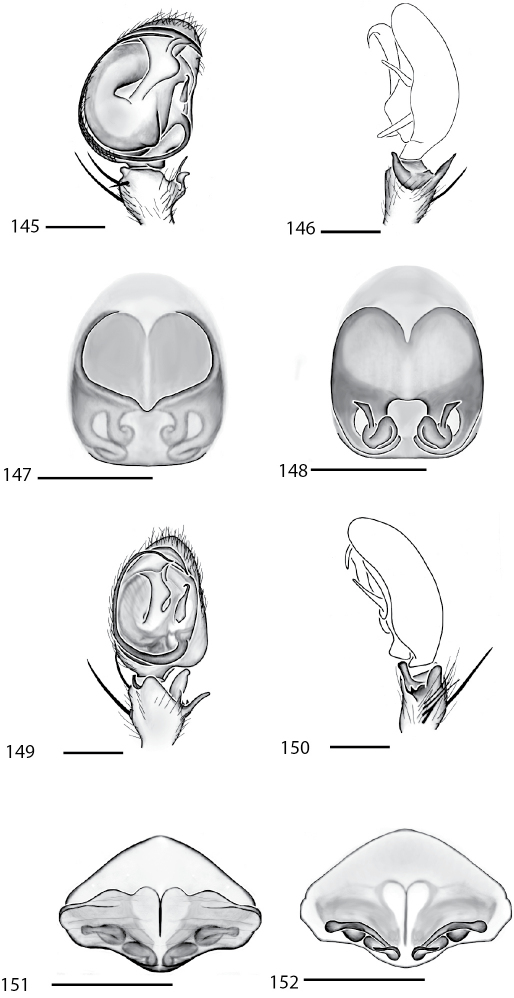
Copulatory organs of *Selenops kalinago* sp. n. male from north side of Silver Hills, Montserrat (EME sel_772) **145–146** *Selenops souliga* sp. n. holotype female from Emilio Wilson Estate, St. Maarten (EME sel_711) **147–148** allotype male from southeast side of island, trail from Back Bay to Geneve Bay, St. Maarten (EME sel_119) **149–150** *Selenops bani* Alayón-García female from Lago Enriquillo, El Azufrada, La Descubierta, Independencia, Dominican Republic (EME sel_190) **151–152, 145, 149** left pedipalp, ventral view, **146, 150** left pedipalp, retrolateral view **147, 151** epigyne, ventral view **148, 152** spermathecae, dorsal view. Scale bar = 0.50 mm.

The females of *Selenops kalinago* sp. n. can be separated from all other species by the shape of the epigynal plate, the small, slit-like genital opening, and internally, the very long sperm ducts and small spermathecae (Figs 143–144). The males can be separated from all other species by the long, thin, finger-like median apophysis (Figs 145–146).

#### Remarks.

There is variation in the coloration of these species as some specimens are light and others are dark. There are also slight differences in the dorsal pattern of the abdomen.

#### Description.

*Paratype male:* Color:carapace brown-yellow with darker marks medially and laterally, more grey in life; sternum light yellow; chelicerae brown, darker laterally; maxillae light yellow-brown, lightening to white distally; labium pale brown; abdomen dorsally grey-tan with darker flecks and spots, festoon pattern caudally; ventrally dusky grey with no markings; legs yellowish with annulations on femora and tibiae, darkening toward tarsus, though annulations still visible. Carapace:0.86 times longer than broad; fovea longitudinal, broad, very shallow. Eyes:AER nearly straight; PER slightly recurved; PME same size as AME, PLE largest, ALE smallest; eye diameters, AME 0.30, ALE 0.10, PME 0.30, PLE 0.40; interdistances AME-PME 0.05, PME-ALE 0.03, ALE-PLE 0.28. PME-PME 1.20. ALE-ALE 1.88; ocular quadrangle AME-AME 0.43, PLE-PLE 1.90; clypeus 0.06 high. Mouthparts:chelicerae with stout setae medially and anteriorly; maxillae longer than broad, with tuft of conspicuous setae distally; labium distally rounded. Sternum:0.88 times longer than broad, posteriorly indented. Legs:leg I only slightly shorter than II and III; leg formula 2134; scopulae present on distal end of all 4 tarsi; tarsi I-IV with strong claw tufts ; claws without teeth; spination: leg I, Fm pr 1–1–1, d 1–1–1, rl 1–1–1; Ti d 1–1–0, pr 1–0–1, rl 1–0–1, v 2–2–2; Mt v 2–2; leg II, Fm pr 1–1–1, d 1–1–1, rl 1–1–1; Ti pr 1–1–1, d 1–1–1, rl 1–1–1, v 2–2–2; Mt v 2–2; leg III, Fm pr 1–1–1, d 1–1–1, rl 1–1–1; Ti pr 1–0–0, d 1–0–0, rl 1–1–0, v 2–2–1; Mt v 2–1; leg IV, Fm pr 1–1–0, d 1–1–1, rl 1–1–1; Ti pr 1–0–0, v 2–2–0, rl 1–1–0; Mt rl 1–0–0, v 2–2–0. Abdomen:without terminal setal tufts. Pedipalp:Fm spination pr 0–0–1, d 0–1–2, rl 0–0–1; cymbium teardrop-shaped ventral view, apex closer to 1 o'clock than 12 o'clock; conductor large, arising medially on long stalk with rounded lateral projection, pointed laterally, extending to 2 o'clock position, curving around retrolateral side; embolus very long, slender, curving around periphery of cymbium, beginning at 4 o'clock, terminating at 1 o'clock; MA located at 3 o'clock, long, thin and finger-like, directed distally, only slightly hooked at end; tibial apophyses barely reaching cymbium; ventral tibial apophysis small, widening distally, flattened in ventral view, curved ventrally in lateral view; dorsal tibial apophysis is slightly larger than ventral tibial apophysis, narrow, tapering to point (Figs 145–146). *Dimensions:* Total length 7.55. Carapace length 3.68, width 4.30. Sternum length 2.00, width 1.75. Abdomen length 3.88, width 3.10. Pedipalp: Fm 1.00, Pt 0.25, Ti 0.25, Ta 1.00, total 2.50. Leg I: Fm 4.50, Pt 1.75, Ti 4.75, Mt 4.25, Ta 1.75, total 17.00. Leg II: Fm 5.75, Pt 1.75, Ti 4.75, Mt 4.60, Ta 1.75, total 18.60. Leg III: Fm 5.00, Pt 1.50, Ti 4.50, Mt 4.00, Ta 1.50, total 16.50. Leg IV: Fm 4.75, Pt 1.00, Ti 4.00, Mt 3.90, Ta 1.50, total 15.15.

*Holotype female:* Color:Carapace light brown with slightly darker marks medially and laterally when preserved, more grey in life; sternum light brown, darker around border; chelicerae brown, darker laterally; maxillae light orange-brown, dark on outer distal edge, white on inner distal edge; labium orange-brown, lightening toward the distal edge; abdomen dorsally grey-tan with darker flecks and spots medially and laterally, festoon pattern caudally; ventrally cream-colored; legs yellowish with annulations on femora and tibiae, darkening toward tarsi, though annulations still visible. Carapace:0.97 times longer than broad. Eyes:AER nearly straight; PER recurved; PME larger than AME, PME same size as PLE, ALE smallest; eye diameters, AME 0.20, ALE 0.18, PME 0.30, PLE 0.30; interdistances AME-PME 0.15, PME-ALE 0.45, ALE-PLE 0.45. PME-PME 1.40. ALE-ALE 2.20; ocular quadrangle AME-AME 0.48, PLE-PLE 2.25; clypeus 0.07 high. Mouthparts:chelicerae with stout setae medially and anteriorly; maxillae longer than broad, with tuft of conspicuous setae distally; labium distally rounded. Sternum: as long as broad, posteriorly indented. Legs:Leg I only slightly shorter than II and III; leg formula 2314; legs I and II with tarsal and metatarsal scopulae; tarsi I-IV with strong claw tufts; claws without teeth; spination: leg I, Fm pr 1–1–0, d 1–1–1, rl 0–1–1 (R); Ti v 2–2–2; Mt v 2–2; leg II, Fm pr 1–0–0, d 1–1–1, rl 0–1–1; Ti v 2–2–2; Mt v 2–2; leg III, Fm pr 1–0–0, d 1–1–1, rl 0–0–1; Ti v 2–2–0; Mt v 2–1; leg IV, Fm pr 1–0–0, d 1–1–1, rl 0–0–1; Ti v 2–2–0; Mt v 2–1. Abdomen:Without terminal setal tufts. Pedipalp:claw with ca.10 teeth. Epigyne:lateral lobes visible from posterior margin to center of epigynal plate, fused, genital openings located behind crescent shaped slit anteriorly; internally, sperm ducts long, narrow, curving laterally to medially, with apex of curve pointing anteriorly, ducts twisted before reaching small lateral spermathecae posteriorly, fertilization ducts located posteriorly, directed anteriorly, posterodorsal fold absent (Figs 143–144). *Dimensions:* Total length 11.05. Carapace length 4.15, width 4.30. Sternum length 2.00, width 2.00. Abdomen length 6.90, width 4.60. Pedipalp: Fm 1.00, Pt 0.50, Ti 0.40, Ta 1.10, total 3. Leg I: Fm 4.40, Pt 2.00, Ti 3.75, Mt 3.00, Ta 1.00, total 14.15. Leg II: Fm 5.00, Pt 1.80, Ti 4.75, Mt 3.00, Ta 1.00, total 15.55. Leg III: Fm 5.00, Pt 1.75, Ti 4.00, Mt 3.50, Ta 0.75, total 14.50. Leg IV: Fm 4.50, Pt 1.50, Ti 3.75, Mt 3.00, Ta 1.00, total 13.75.

#### Natural history.

This species has been collected from under the bark of Campeche, *Bursera*, *Psidium*, bay, sea grape, and other trees with loose bark in dry coastal forests from sea level to 300 m elevation (Figs 191, 204). Egg sacs are single, flat, white discs, attached under bark and guarded by the female, often with a light cocoon of silk around the female and the egg sac.

#### Distribution.

Known from the islands of Antigua, Guadeloupe, including Les Saintes, and Montserrat (Map 14).

### Selenops souliga sp. n.

urn:lsid:zoobank.org:act:998B16EB-F8E5-4840-82B3-6F7B0E803809

http://species-id.net/wiki/Selenops_souliga

Figs 147–150 192–193 205 Map 9

#### Type material.

Holotype female, Emilio Wilson Estate, St. Maarten, 18°02'32.7"N, 63°03'53.1"W, ~ 22m, 20.II.2007, S. Crews, B.M. Nisbeth, on palms, buildings at night, SCC07_026 (EME sel_711). Paratypes: Male, St. Maarten, southeast side of island, trail from Back Bay to Geneve Bay, 18°00.929'N, 63°01.840'W, ~25 m, 13.XI.2004, S. Crews, under debris on ground, SCC04_068 (EME sel_119).

#### Other material examined.

ANGUILLA: Blowing Point:
18°10'18.0"N, 63°05'28.7"W, sea level, 13.II.2007, S. Crews, R. Connor, under bark of sea grape, SCC07_018, 1♀, 1p♂, 1 imm. (CAS sel_705-707); **Long Bay:** Long Bay Beach, 18°11'29.3"N, 63°07'49.7"W, sea level, 13.II.2007, S. Crews, R. Connor, under rocks, on seagrape, in *Agave* along beach, SCC07_015, 1♀, 4 imm. (EME sel_698-702). **Shoal Bay West:** 18°09'52.8"N, 63°09'21.3"W, sea level, 13.II.2007, S. Crews, R. Connor, on palm frond guarding egg sac, SCC07_016, 1♀ (CAS sel_697). **The Cove:** 18°10'14.1"N, 63°07'52.6"W, sea level, 13.II.2007, S. Crews, R. Connor, under bark of sea grape, under rocks, SCC07_017, 1♀, 1 imm. (EME sel_703-704). Windward Point: 18°16'18.2"N, 62°58'05.3"W, sea level-13 m, 12.II.2007, S. Crews, R. Connor, under stone, SCC07_014, 1 imm. (CAS sel_696). **SABA: Giles Quarter Trail:** 1 km up, 17.6151°N, 63.2432°W, 13 m, 12.III.2008, under rocks, J. Slowik, 1p♂, 1 imm. (EME sel_1021-1022). **ST. MAARTEN: Emilio Wilson Park:** same data as holotype, 1♀, 3 imm. (CAS sel_715-717). **Mullet Bay:** Mullet Bay Resort, 18°02'48.0"N, 63°07'29.7"W, ~31 m, 21.II.2007, S. Crews, B.M. Nisbeth, under bark, debris on ground, SCC07_027, 1♂, 3♀, 13 imm. (CAS sel_712-713, 718-730). **Back Bay:** same data as paratype, 1♀, 1♂, 3 imm. (EME sel_116, 120-121, 708-710). **Upper Princess Quarter:** 18°01'48.0"N, 63°02'08.0"W, ~32 m, 21.II.2007, S. Crews, B.M. Nisbeth, under bark of dry, dead trees on hillside, SCC07_028, 2 imm. (EME sel_731-732).

#### Etymology.

The specific epithet comes from the indigenous word Souliga, for the island of St. Maarten, the type locality. It is to be treated as a noun in apposition.

#### Diagnosis.

Females can be separated from all others by the median field of the epigynum, which is heart-shaped, and the huge spermathecae (Figs 147–148). Males can be differentiated from other species by the extremely dorsoventrally flattened palpus and the very small RTA (Figs 149–150).

#### Description.

*Paratype male:* Color:carapace orange-brown, duskier laterally, white setae on sides and in cephalic area; sternum pale yellow; chelicerae orange-brown, with some dusky markings; maxillae pale yellow; labium pale yellow; abdomen dorsally brownish-grey darkening distally, markings similar to female, though not as pronounced distally, chevrons posteriorly, and festoon prominent; ventrally light grey; legs yellowish, becoming slightly darker distally, faint annulations present. Carapace: 0.90 times longer than broad; fovea longitudinal, broad, very shallow. Eyes:AER slightly recurved; PER recurved; PME larger than AME, PLE largest, ALE smallest; eye diameters, AME 0.18, ALE 0.08, PME 0.20, PLE 0.38; interdistances AME-PME 0.03, PME-ALE 0.80, ALE-PLE 0.30. PME-PME 0.98. ALE-ALE 1.45; ocular quadrangle AME-AME 0.35, PLE-PLE 1.60; clypeus 0.08 high. Mouthparts:chelicerae with stout setae medially and anteriorly; maxillae longer than broad, with tuft of conspicuous setae distally; labium distally rounded. Sternum:1.07 times longer than broad, posteriorly indented. Legs:leg I only slightly shorter than leg II; leg formula unknown (at least one leg missing); leg II longest; scopulae present on distal end of all 4 tarsi; tarsi I-IV with strong claw tufts; claws pr claw per foot slightly toothed; spination: leg I, Fm pr 1–1–1, d 1–1–1, rl 1–1–1; Ti d 0–1–0, pr 1–0–1, rl 1–0–1, v 2–2–2; Mt pr 1–0–0, v 2–2, rl 1–0–0; leg II, Fm pr 1–1–1, d 1–1–1, rl 1–1–1; Ti pr 1–0–1, d 0–1–0, rl 1–0–1, v 2–2–2; Mt pr 1–1–0, rl 1–0–0, v 2–2; leg IV, Fm pr 1–1–1, d 1–1–1, rl 1–1–1; Ti pr 1–0–1, v 2–2–0, rl 1–0–1; Mt pr 0–1–0, rl 1–0–0, v 2–2. Abdomen:without tufts of setae. Pedipalp:Fm, spination d 0–1–4; cymbium oval, angled, truncate posterolaterally, extremely dorsoventrally flattened in lateral view; conductor T-shaped, arising from center of bulb on long stalk with laterally directed rounded projection, conductor attached laterally, tapered, pointed retrolaterally; embolus long, slender, beginning at 4 o'clock, terminating at 1 o'clock; MA long, finger-like, hooked distally; RTA small, with 2 branches, dorsal branch directed laterally, rounded distally in lateral view, ventral apophysis truncate distally, with small, basal, thornlike projection; RTA barely reaching cymbium in ventral view (Figs 149–150). *Dimensions:* Total length 6.10. Carapace length 3.00, width 3.35. Sternum length 1.60, width 1.50. Abdomen length 3.10, width 2.30. Pedipalp: Fm 0.80, Pt 0.30, Ti 0.25, Ta 0.75, total 2.10. Leg I: Fm 3.75, Pt 1.50, Ti 4.25, Mt 4.00, Ta 1.75, total 15.25. Leg II: Fm 4.50, Pt 1.65, Ti 4.00, Mt 4.50, Ta 1.75, total 16.04. Leg III: Missing. Leg IV: Fm 4.00, Pt 1.00, Ti 3.50, Mt 3.75, Ta 1.50, total 13.75.

*Holotype female:* Color:carapace light brown with darker marks laterally and mediolaterally, very dark around eyes; sternum light yellow-orange-brown, darker around border; chelicerae brown, dusky markings anteromedially, terminating medially; maxillae light yellow-brown, lightening to white distally; labium light brown lightening distally; abdomen dorsally overall brownish-orange, darker medially, lanceolate stripe, with 3 chevrons extending laterally to darker areas, occurring in the upper 1/3rd of the abdomen, the middle, and the lower 1/3rd, with a sinuous line posteriorly, festoon prominent; ventrally dusky yellow-grey, no markings; legs light brownish-orange, darkening distally, annulations prominent, black streak on anterolateral face of femora I and II. Carapace: 0.90 times longer than broad; fovea longitudinal, broad, very shallow. Eyes:AER nearly straight; PER slightly recurved; PME larger than AME, PLE largest, ALE smallest; eye diameters, AME 0.13, ALE 0.03, PME 0.20, PLE 0.35; interdistances AME-PME 0.03, PME-ALE 0.13, ALE-PLE 0.38. PME-PME 0.98. ALE-ALE 1.68; ocular quadrangle AME-AME 0.38, PLE-PLE 1.75; clypeus 0.09 high. Mouthparts:chelicerae with a few stout setae medially and anteriorly; maxillae longer than broad, with tuft of conspicuous setae distally; labium distally rounded. Sternum:as long as broad, posteriorly indented. Legs:leg I only slightly shorter than legs II, III and IV; leg formula 3241; scopulae present on all 4 tarsi and metatarsi and tibiae I and II; tarsi I-IV with strong claw tufts; pr claw per foot slightly toothed; spination: leg I, Fm pr 1–1–0, d 1–1–1, rl 1–1–1; Ti d 0, v 2–2–2; Mt v 2–2; leg II, Fm pr 1–0–0, d 1–1–1, rl 1–0–1; Ti v 2–2–2; Mt v 2–2; leg III, Fm pr 1–0–0, d 1–1–1, rl 1–1–1; Ti v 2–2–0; Mt v 2–2; leg IV, Fm pr 1–0–0, d 1–1–1, rl 0–0–1; Ti v 2–2–0; Mt v 2–2. Abdomen:with terminal setal tufts. Pedipalp:claw with 8 teeth. Epigyne:median field large nearly heart-shaped opening, genital openings occurring at posterior margin of ‘heart', epigynal pockets absent; internally, spermathecae huge, very heavily sclerotized, with 2 pair of coiled ducts locatedterminally, fertilization ducts located posteriorly, directed anteriorly, posterodorsal fold absent (Figs 147–148). *Dimensions:* Total length 6.60. Carapace length 3.10, width 3.45. Sternum length 1.75, width 1.75. Abdomen length 3.50, width 3.00. Pedipalp: Fm 0.70, Pt 0.25, Ti 0.30, Ta 1.00, total 2.25. Leg I: Fm 3.50, Pt 1.50, Ti 3.00, Mt 2.60, Ta 1.00, total 11.60. Leg II: Fm 4.00, Pt 1.60, Ti 3.50, Mt 2.00, Ta 1.00, total 12.10. Leg III: Fm 4.00, Pt 1.50, Ti 3.00, Mt 2.90, Ta 1.90, total 13.30. Leg IV: Fm 3.90, Pt 1.00, Ti 3.00, Mt 2.80, Ta 1.00, total 11.70.

#### Natural history.

This species has been found under bark, under debris on the ground, under rocks, in the bases of tropical plants, on buildings, fences, and tree trunks at night (Figs 192, 205). The female guards the white, disc-shaped egg sac (Fig. 193).

#### Distribution.

Found in the northern Lesser Antilles on the islands of Anguilla, St. Maarten, and Saba (Map 9).

### Selenops bani

Alayón-García, 1992

http://species-id.net/wiki/Selenops_bani

Figs 151 154 Map 13

Selenops bani Alayón-García 1992: 5, Fig. 4 A-B (♀, not examiend).

#### Type material.

Holotype female: La Laguna, carretera Baní-Manaclar, km 6.5, Peravia, Dominican Republic (IES), not examined.

#### Other material examined.

DOMINICAN REPUBLIC: Independencia: La Descubierta, El Azufrada, north side of Lago Enriquillo, 18°33.751'N, 71°41.853'W, -15 m, 26.XI.2004, S. Crews under rocks along trail to lake, SCC04_084, 2♂, 1♀ (EME sel_188, 190, MNHNSD sel_189).

#### Diagnosis.

**Figures 153–160.**
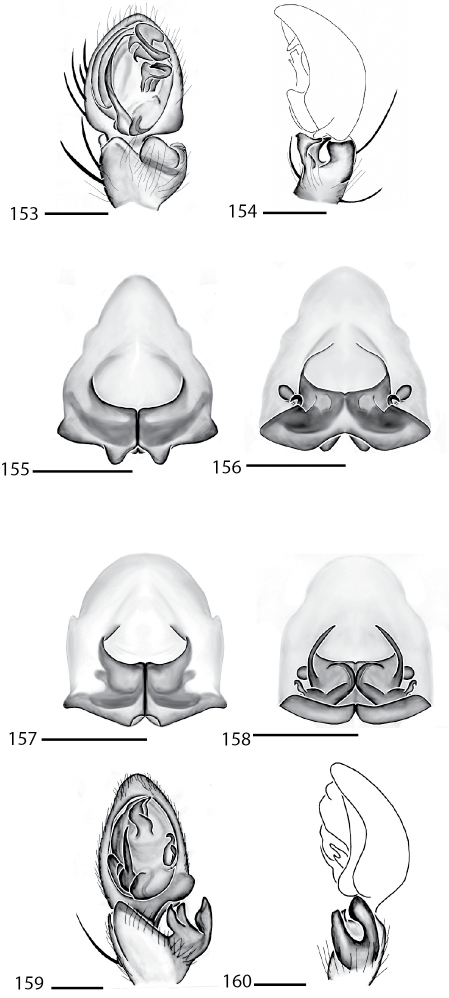
Copulatory organs of *Selenops bani* Alayón-García male from Lago Enriquillo, El Azufrada, La Descubierta, Independencia, Dominican Republic (EME sel_188) **153–154** *Selenops pensilis* Muma holotype female from Grand'Anse, Haiti (MCZ) **155–156** *Selenops enriquillo* sp. n. holotype female from El Azufrada, La Descubierta, Lago Enriquillo, Prov. Independencia, Dominican Republic (EME sel_182) **157–158** male from Isla Cabrito, Lago Enriquillo, Dominican Republic (MNHNSD) **159–160, 153, 159** left pedipalp, ventral view **154, 160** left pedipalp, retrolateral view **155, 157** epigyne, ventral view **156, 158** spermathecae, dorsal view. Scale bar = 0.50 mm.

Females can be separated from other species by the squarish lateral lobes, the sinuous opening which extends the width of the epigynal plate, and the internal genitalic structures; the sperm ducts and spermathecae are very small and simple (Figs 151–152). Males can be distinguished from other species by the c-shaped distal end of the conductor and the ventrally projecting large MA (Figs 153–154).

#### Description.

*Male (sel_188):* Color: carapace light yellowish, slightly darker laterally and medially; sternum pale yellow; chelicerae uniformly light yellow; maxillae pale yellow; labium dusky yellow, lightening distally; abdomen dorsally grey-yellow with some darker flecks; ventrally pale yellow, no markings; legs light yellow, all segments with faint annulations. Carapace:0.91 times longer than broad. Eyes:AER nearly straight; PER recurved; PME larger than AME, PLE largest, ALE smallest; eye diameters, AME 0.08, ALE 0.03, PME 0.18, PLE 0.26; interdistances AME-PME 0.03, PME-ALE 0.08, ALE-PLE 0.23. PME-PME 0.65. ALE-ALE 1.20; ocular quadrangle AME-AME 0.20, PLE-PLE 1.20; clypeus 0.04 high. Mouthparts:chelicerae with a few stout setae medially and anteriorly; maxillae longer than broad, with tuft of conspicuous setae distally; labium distally rounded. Sternum:1.10 times longer than broad, posteriorly indented. Legs:Leg I only slightly shorter than legs II, III and IV; leg formula 3241; present on distal end of all 4 tarsi; tarsi I-IV with strong claw tufts; pr claw per foot slightly toothed; spination: leg I, Fm pr 1–1–1, d 1–1–1, rl 1–1–1; Ti d 1–1–0, pr 1–0–1, rl 1–0–1, v 2–2–2–2; Mt v 2–2; leg II, Fm pr 1–1–1, d 1–1–1, rl 1–1–1; Ti pr 1–0–1, d 1–1–0, rl 1–0–1, v 2–2–2; Mt v 2–2; leg III, Fm pr 1–1–1, d 1–1–1, rl 1–1–1; Ti pr 1–1–0, d 1–0–0, rl 1–1–0, v 2–2–0; Mt pr 1–1–0, d 0, rl 1–0–0, v 2–2–1; leg IV, Fm pr 1–1–0, d 1–1–1, rl 1–1–1; Ti pr 0–0–1, v 2–2–0, rl 1–1–1; Mt pr 1–1–0, rl 1–0–0, v 1–2–2. Abdomen:without terminal setal tufts. Pedipalp:Fm spination pr 0–0–1, d 0–1–2, rl 0–0–1; cymbium oval in ventral view, truncate posterolaterally; conductor curves laterally around side of bulb, tip folded, sinuous, slightly c-shaped, not pointed, projects ventrally; embolus short with, a thickened base, slightly curved, tapered to point, beginning at 6 o'clock, terminating at 11 o'clock, not curving around edge of cymbium, but more medially; MA stout, with blunt hook on triangular base located at 3 o'clock, projecting ventrally; tibial apophyses barely reach cymbium in ventral view, both short, quadrangular, blunt (Figs 153–154). *Dimensions:* Total length 4.23. Carapace length 2.13, width 2.33. Sternum length 1.10, width 1.00 Abdomen length 2.10, width 1.93. Pedipalp: Fm 0.50, Pt 0.25, Ti 0.30, Ta 0.75, total 1.80. Leg I: Fm 3.50, Pt 0.75, Ti 3.60, Mt 3.60, Ta 1.60, total 13.05. Leg II: Fm 4.00, Pt 1.00, Ti 2.75, Mt 4.00, Ta 1.75, total 13.50. Leg III: Fm 4.00, Pt 0.75, Ti 3.75, Mt 3.80, Ta 1.50, total 13.80. Leg IV: Fm 4.00, Pt 0.75, Ti 3.30, Mt 3.75, Ta 1.60, total 13.40.

*Female (sel_190):* Color:carapace brown-yellow with darker marks medially and laterally, more grey in life; sternum dusky yellow, darker around border; chelicerae brown, darker laterally; maxillae light yellow-brown, lightening to white distally; labium dusky yellow, lightening distally; abdomen dorsally grey-tan with a yellowish tinge, darker medially; ventrally dusky grey with no markings; legs light yellow, all segments with faint annulations. Carapace: 0.73 times longer than broad. Eyes:anterior eye row nearly straight; posterior eye row recurved; PME larger than AME, PLE largest, ALE smallest; eye diameters, AME 0.08, ALE 0.05, PME 0.28, PLE 0.30; interdistances AME-PME 0.03, PME-ALE 0.15, ALE-PLE 0.30. PME-PME 0.85 mm. ALE-ALE 1.45; ocular quadrangle AME-AME 0.25, PLE-PLE 1.63; clypeus 0.03 high. Mouthparts:Chelicerae with stout setae medially and anteriorly; maxillae longer than broad, with tuft of conspicuous setae distally; labium distally rounded. Sternum:0.83 times longer than broad, posteriorly indented. Legs:Leg I only slightly shorter than legs II, III and IV; leg formula 2431; legs I and II with tarsal and metatarsal scopulae; tarsus I-IV with strong claw tufts; pr claw per foot slightly toothed; spination: leg I, Fm pr 0–1–1, d 1–1–1, rl 1–1–0; Ti v 2–2–2; Mt v 2–2; leg II, Fm pr 0–0–1, d 1–1–1, rl 1–0–0; Ti v 2–2–2; Mt v 2–2; leg III, Fm pr 1–0–1, d 1–1–1, rl 1–0–0; Ti v 2–2–0; Mt v 2–2; leg IV, Fm pr 0, d 1–1–1, rl 1–0–0; Ti v 2–2–0; Mt v 2–2. Abdomen:Without terminal setal tufts. Pedipalp:Claw with 7 teeth. Epigyne:epigynal plate wider than long; lateral lobes rectangular, partially fused, slightly sinuous anteriorly, with genital openings located medially located medially; internally, very simple, small spermathecae located laterally, connected by long narrow ducts, fertilization ducts located posteriorly, directed laterally, posterodorsal fold absent (Figs 151–152). *Dimensions:* Total length 5.08. Carapace length 2.40, width 3.28. Sternum length 1.25, width 1.50. Abdomen length 2.68, width 2.50. Pedipalp: Fm 0.60, Pt 0.30, Ti 0.35, Ta 0.75, total 2.00. Leg I: Fm 2.80, Pt 0.80, Ti 2.00, Mt 1.90, Ta 1.00, total 8.50. Leg II: Fm 3.20, Pt 1.10, Ti 2.60, Mt 2.00, Ta 1.00, total 9.90. Leg III: Fm 2.20, Pt 1.00, Ti 2.65, Mt 2.10, Ta 0.90, total 8.85. Leg IV: Fm 3.00, Pt 0.80, Ti 2.50, Mt 2.20, Ta 0.8, total 9.30.

#### Remarks.

The leg formulae are different than those given by Alayón-García (1992). This is likely due to variation, and thus attests to the problem with using leg formulae for classification purposes in this genus. This species appears to be most similar to the Cuban endemic, *Selenops vinalesi,* along with *Selenops alemani*, *Selenops submaculosus*, *Selenops simius*, and *Selenops morro* sp. n., the latter being the only species of the aforementioned that occurs on Hispaniola.

#### Natural history.

Collected from under rocks in dry forests.

#### Distribution.

Known only from the Dominican Republic on the island of Hispaniola (Map 13).

### Selenops pensilis

Muma, 1953

http://species-id.net/wiki/Selenops_pensilis

Figs 155–156 Map 13

Selenops pensilis Muma 1953: 42, Fig. 73 (♀, examined).
Selenops pensilis :Alayón-García 1992: 2, Fig. 2 (♀).

#### Type material.

Holotype female: Grand'Anse, Haiti, Uhler (MCZ, examined).

#### Other material examined.

DOMINICAN REPUBLIC: La Altagracia: along 4–5 km S caseta Gran Chorra, near Palmilla, 5.V.1992, 1♀ (MNHNSD); Punta Cana, Punta Cana Resort, 5-8 July 2006, sea level, S. Crews, under rock on egg sac, 1♀ (EME sel_528).

#### Diagnosis.

Though very similar to *Selenops enriquillo* sp. n., the genital openings and median field are more posterior on the epigynal plate. Internally, the ducts and posterodorsal fold are shaped differently (Figs 155–156). Males unknown.

#### Remarks.

This species is closely related to *Selenops enriquillo* sp. n. It differs in genitalic details and molecular genetic makeup. As the specimens from Alayón-García (1992) were unable to be examined, it is unclear if he was referring to *Selenops pensilis* or *Selenops enriquillo* sp. n.

#### Description.

*Holotype female:* Color:carapace (holotype) in bad shape, orange-brown (recent) light brown, duskier markings medially, on edges of cephalic region, 1 dark spot on each side of cephalic region; sternum light yellow-orange-brown, darker around border; chelicerae brown, dusky markings anteromedially terminating halfway down the length of chelicerae; maxillae light orange-brown, dark on outer distal edge, white on inner distal edge; labium brown, lightening distally; abdomen dorsally yellow-grey, medial lanceolate stripe, slightly faded, border only represented by dots, chevron near the end of stripe, festoon present; ventrally cream-colored, dark laterally and posteriorly; legs pale yellow-brown, darkening distally, annulations prominent, long stripe on anterolateral faces of femora, anterior and retrolateral faces of tibia and metatarsus of leg III. Carapace 0.91 times longer than broad; fovea longitudinal, broad, very shallow. Eyes:AER nearly straight; PER recurved; PME larger than AME, PLE largest, ALE smallest; eye diameters, AME 0.23, ALE 0.10, PME 0.33, PLE 0.40; interdistances AME-PME 0.03, PME-ALE 0.20, ALE-PLE 0.36. PME-PME 1.13. ALE-ALE 2.05; ocular quadrangle AME-AME 0.48, PLE-PLE 2.18; clypeus 0.11 high. Mouthparts:chelicerae with stout setae medially and anteriorly; maxillae longer than broad, with tuft of conspicuous setae distally; labium distally rounded. Sternum:0.76 times longer than broad, posteriorly indented. Legs:leg I only slightly shorter than II and III; leg formula 4321 (Muma, 1953); scopulae present on all 4 tarsi and metatarsi and tibiae I and II; tarsi I-IV with strong claw tufts; pr claw per foot slightly toothed; spination: leg I, Fm pr 1–1–0, d 1–1–1, rl 1–1–1; Ti d 0, v 2–2–2; Mt v 2–2; leg II, Fm pr 1–1–0, d 1–1–1, rl 1–1–1; Ti v 2–2–2; Mt v 2–2; leg III, Fm pr 1–1–0, d 1–1–1, rl 1–1–1; Ti v 2–2–0; Mt v 2–2. Abdomen:with terminal setal tufts. Pedipalp:claw with 8 teeth. Copulatory organs:median field U-shaped, depressd, lateral lobes distinct, medially, lobes directed posteriorly, pointed terminally, genital openings located at posterior margin of median field, epigynal pockets present; internally, highly sclerotized ducts, posterodorsal fold fused with internal ducts, small oval spermathecae and fertilization ducts located laterally (Figs 155–156). *Dimensions:* Total length 9.15. Carapace length 4.43, width 4.85. Sternum length 2.50, width 1.90. Abdomen length 4.73, width 4.55. Pedipalp: Fm 1.25, Pt 0.35, Ti 0.80, Ta 1.20, total 3.60. Leg I: Fm 4.90, Pt 1.80, Ti 4.00, Mt 3.75, Ta 1.75, total 16.20. Leg II: Fm 5.00, Pt 2.00, Ti 4.60, Mt 3.75, Ta 1.75, total 17.10. Leg III: Fm 5.00, Pt 1.75, Ti 4.00, Mt 3.75, Ta 1.75, total 16.25. Leg IV: Missing.

#### Natural history.

This species has been found under rocks. The female guards the egg sac.

#### Distribution.

Endemic to the island of Hispaniola (Map 13).

### Selenops enriquillo sp. n.

urn:lsid:zoobank.org:act:FADAB1E3-642B-4F5E-96A9-1760DA78236D

http://species-id.net/wiki/Selenops_enriquillo

Figs 157–160 Map 13

#### Type material.

Holotype female: El Azufrada, La Descubierta, Lago Enriquillo, Prov. Independencia, Dominican Republic, 18°33.751'N, 71°41.853'W, -15 m, 26 XI.2004, S. Crews, under rocks along trail, SCC04_084 (EME sel_182). Paratypes: Male, Isla Cabrito, Lago Enriquillo, Dominican Republic, 6.I.1982, E. Marcano (MNHNSD).

#### Other material examined.

DOMINICAN REPUBLIC: same data as the holotype, 2♀, 1 imm. (CAS sel_183, 185–186). **HAITI: Jacmel:** St. Cyr, 18°14'16.6"N, 72°31'41.2"W, 23.XI.2006, sea level, S. Crews, on trees and wall at night, SCC06_078, 1 imm. (EME sel_661). **JAMAICA: Kingston:** 11.XII.1970, D.B. Denning, 1♀ (AMNH).

#### Etymology.

The specific epithet comes from the name of the cacique Enriquillo who rebelled against the invading Spanish. It is to be treated as a noun in apposition.

#### Diagnosis.

This species is very similar to *Selenops pensilis*, but females can be differentiated by the depressed median field which is located more anterior than in *Selenops pensilis*, and the margin of the depression is not as rounded. Internally, the ducts are heavily sclerotized, but the posterodorsal fold is more distinct (Figs 157–158). Males can be differentiated from other species by having a two-branched MA and a very short, straight embolus (Fig. 159).

#### Remarks.

Although the male and female were not collected at the same time or from the exact same place, the overall similarity in habitus and the amount of collecting historically accomplished from the type locality indicate these are likely the same species. There is variation in the female copulatory organs, including how far posteriorly the lateral lobes extend beyond the epigastric furrow, how far apart or close together these extensions are, and the shape of the posterodorsal fold. Although one species was taken from Kingston, Jamaica, I do not believe this species naturally occurs there.

#### Description.

*Paratype male:* Color:light orange-brown; sternum pale yellow; chelicerae light orange-brown, dusky marking anteromedially continuing half the length of the chelicerae; maxillae pale yellow, lighter distally; labium pale yellow; abdomen dorsally yellow-grey, medial lanceolate stripe, slightly faded, some chevrons, white anteriorly and medially, extending laterally, festoon present; ventrally pale yellow, no markings; legs yellowish-brown, faint annulations present, dark lines on anterolateral faces of legs. Carapace: 0.87 times longer than broad; fovea longitudinal, broad, very shallow. Eyes: AER slightly recurved; PER recurved; PME larger than AME, PLE largest, ALE smallest; eye diameters, AME 0.20, ALE 0.05, PME 0.28, PLE 0.33; interdistances AME-PME 0.05, PME-ALE 0.15, ALE-PLE 0.20. PME-PME 0.98. ALE-ALE 1.68; ocular quadrangle AME-AME 0.38, PLE-PLE 1.73; clypeus 0.10 high. Mouthparts:chelicerae with stout setae medially and anteriorly; maxillae longer than broad, with tuft of conspicuous setae distally; labium distally rounded. Sternum:1.33 times longer than broad, posteriorly indented. Legs:scopulae present on distal end of all 4 tarsi; tarsi I-IV with strong claw tufts; pr claw per foot slightly toothed; spination: leg I, Fm pr 1–1–1, d 1–1–1, rl 1–1–1; Ti d 1–1–0, pr 1–0–1, rl 1–0–1, v 2–2–2; leg II, Fm pr 1–1–1, d 1–1–1, rl 1–1–1. Abdomen:with terminal setal tufts. Pedipalp:Fm, spination d 0–1–4; cymbium oval in ventral view, angled posterolaterally; conductor arising anteromedially, pointed laterally, curving laterally around edge of cymbium; embolus short, arising from round base at 7 o'clock, directed anteriorly, not curving, bladelike, terminating at 10 o'clock; MA bifid, both branches rounded distally, arising at 3 o'clock, directed ventrally; RTA reaching cymbium in ventral view, with two branches, ventral branch bifid, truncate terminally, lateral branch curved slightly ventrally, quadrangular, truncate distally (Figs 159–160). *Dimensions:* Total length 8.10. Carapace length 3.55, width 4.08. Sternum length 2.00, width 1.50. Abdomen length 4.55, width 2.83. Pedipalp: Fm 1.00, Pt 0.25, Ti 0.50, Ta 1.00, total 2.75. Leg I: Fm 5.25, Pt 1.65, Ti 4.75. Leg II: Fm 5.50, Pt 1.75, Ti 5.50, Mt 5.75, Ta 2.00, total 20.50. Leg III: Fm 5.75, Pt 1.75. Leg IV: Missing.

*Holotype female:* Color: carapace brownish-orange with lots of white setae in the cephalic region; sternum orange-brown; chelicerae orange-brown, lighter medially; maxillae light orange-brown, lightening distally; labium orange-brown; abdomen orange-brown to grey, median lanceolate stripe, duskier on edges, festoon present; ventrally grey to cream colored; legs yellow-brown, darkening distally. Carapace: 0.88 times longer than broad; fovea longitudinal, broad, shallow. Eyes: AER nearly straight; PER slightly recurved; PME larger than AME, PLE largest, ALE smallest; eye diameters, AME 0.20, ALE 0.15, PME 0.30, PLE 0.35; interdistances AME-PME 0.10, PME-ALE 0.20, ALE-PLE 0.45. PME-PME 0.60, ALE-ALE 1.50; ocular quadrangle AME-AME 0.40, PLE-PLE 1.85; clypeus 0.08 high. Mouthparts: chelicerae with stout setae medially and anteriorly; maxillae longer than broad, with a tuft of conspicuous setae distally; labium distally rounded. Sternum 1.33 times longer than broad, posteriorly indented. Legs: leg I missing; scopulae present on tarsi; tarsi I-IV with strong claw tufts; spination: leg II, Fm pr 1–1–0, d 1–1–1, rl 0–1–1; Ti v 2–2–2; Mt v 2–2; leg III, Fm pr 1–0–0, d 1–1–1, rl 0; Ti v 2–2–0; Mt v 2–0; leg IV, Fm pr 1–0–0, d 1–1–1, rl 0–0–1; Ti v 1–1; Mt v 2–1. Abdomen: with terminal setal tufts. Pedipalp: claw with 6 teeth. Epigyne: central depression, mostly u-shaped, slightly angular laterally, lateral lobes distinct, genital openings located at posterior margin of median depression, epigynal pockets present; internally, ducts heavily sclerotized, small oval to round spermathecae located laterally, fertilization ducts located posterolaterally, directed laterally, small posterodorsal fold present (Figs 157–158). *Dimensions:* Total length 8.50. Carapace length 3.50, width 4.00. Sternum length 2.00, width, 1.50. Abdomen length 5.00, width 4.00. Pedipalp: Fm 1.20, Pt 1.00, Ti 1.00, Ta 1.20, total 4.40. Leg I: missing. Leg II: Fm 5.00, Pt 1.50, Ti 4.00, Mt 3.25, Ta 1.50, total 15.25. Leg III: Fm 5.00, Pt 1.00, Ti 3.00, Mt 3.00, Ta 1.75, total 13.75. Leg IV: Fm 5.00, Pt 1.50, Ti 3.25, Mt 3.50, Ta 1.50, total 14.75.

#### Natural history.

This species has been found under rocks and on tree trunks at night.

#### Distribution.

Endemic to Hispaniola (Map 13).

### Selenops phaselus

Muma, 1953

http://species-id.net/wiki/Selenops_phaselus

Figs 161–164 Map 13

Selenops phaselus Muma 1953: 42, Figs 71–72 (♂, examined).

#### Type material.

Holotype male: Kenskoff, Haiti, 2.IX.1934, 4500-5500', P.J. Darlington (MCZ, examined).

#### Other material examined.

DOMINICAN REPUBLIC: Pedernales: Sierra de Baoruco, 26 km north of Cabo Rojo, trail off side of road, 18°06.490'N, 71°37.316'W, 24.XI.2004, S. Crews, under bark, SCC04_078, 1♀, 1 imm. (EME sel_ 159, 215); Las Abejas, Parque Nacíonal Sierra de Baoruco, 18°08.804'N, 71°37.164'W, ~1400 m, 24.XI.2004, S. Crews, cloud forest surrounded by pine forest, under bark, SCC04_077, 1♂, 1♀ (CAS sel_158, 160); Parque Nacíonal Sierra de Baoruco, Las Abejas, 18°08'30.2"N, 71°38'12.6"W, 10.X.2006, 1500 m, S. Crews, under bark, SCC06_072, 1♂, 2 imm. (MNHNSD sel_632-634). **Barahona:** Polo Coffee Plantation, 18.07.581 N, 71.16.113 W, 12.VII.2006, 970 m, L. Mahler, on tree, 2♀, 1 imm. (MNHNSD sel_565-566, 585). **HAITI: Kenskoff:** Belot-Montcel, 18°27'11.3"N, 72°21'06.4"W, 20-21.X.2006, 1566 m, S. Crews, E. Fernandez, under rocks, on buildings, stone walls at night, SCC06_076, 5♂, 2♀, 1 imm. (CAS sel_647-654).

#### Diagnosis.

**Figures 161–168.**
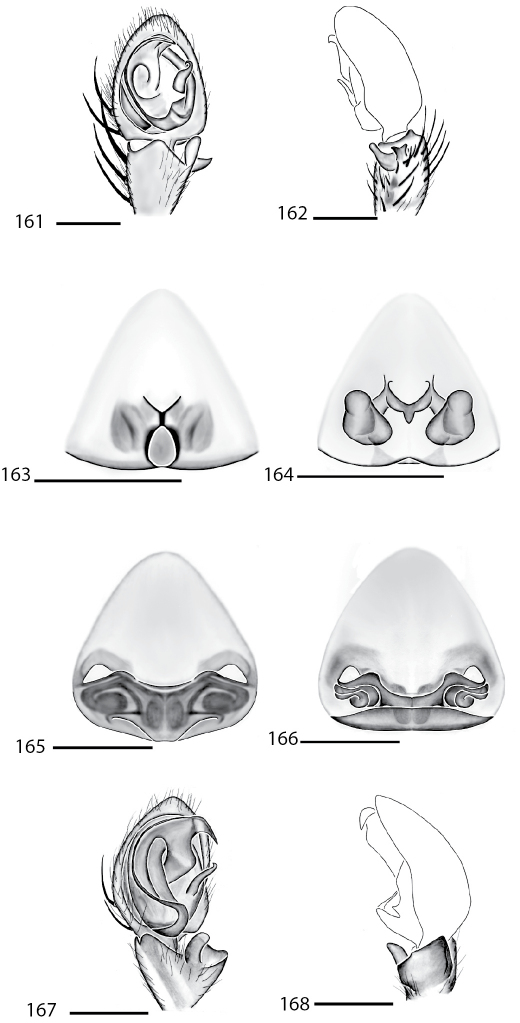
Copulatory organs of *Selenops phaselus* Muma holotype male from Kenskoff, Haiti (MCZ) **161–162** female from 26 km north of Cabo Rojo, Sierra de Baoruco, Pedernales, Dominican Republic (EME sel_159) (**163–164** *Selenops morro* sp. n. holotype female from El Morro, Monte Cristi, Dominican Republic (EME sel_614) **165–166** male from El Morro, Monte Cristi, Dominican Republic (EME sel_582) **167–168, 161, 167** left pedipalp, ventral view **162, 168** left pedipalp, retrolateral view **163, 165** epigyne, ventral view **164, 166** spermathecae, dorsal view. Scale bar = 0.50 mm.

Males can be separated from others by the very small RTA, with a quadrangular lateral apophysis and a rounded ventral apophysis, and the embolus which does not curve around the edge of the cymbium (Figs 161–162). The external female copulatory organs are similar to those of *Selenops insularis*, but the epigynal pockets are as long as the v-shaped genital opening, the internal copulatory organs are shaped differently, and there is no posterodorsal fold (Figs 163–164).

#### Remarks.

The leg formula given by Muma (1953) was 2341. My measurements give 2314. All of the leg lengths are very close, except for leg II, which is quite long. This is further evidence that leg lengths are probably not the best character to use to determine relationships in this genus. There is some variation in the female copulatory organs. In the majority of specimens, the longitudinal line separating the two lateral lobes is visible between the v-shaped genital opening and the epigynal pockets. In one specimen (sel_160), this line is not present, thus the lateral lobes are inconspicuous. There are no other differences noted in this specimen in genitalic or somatic characters. There is some variation in coloration in both males and females. Preserved specimens can range from a yellowish coloration to a darker more brownish-red coloration in both males and females. In life, the spider is a grey-green to blue-green, almost metallic, perfectly matching the wet, cloud forest lichens covering the trees and rocks on which it lives.

#### Description.

*Holotype male:* Color: carapace (holotype) dark red-brown with patches of white setae (recent) uniform brown, some light colored setae; sternum (holotype) orange-brown, darker around border (recent) yellow darker around border; chelicerae (holotype) dark red-brown (recent) yellow-brown with black flecks anterolaterally; maxillae (holotype) light orange-brown, lightening distally (recent) dusky yellow; labium (holotype) orange-brown (recent) light dusky yellow, lightening distally; abdomen dorsally (holotype) yellow-grey with two w-shaped faded patches, one near the top of the abdomen and one near the end, festoon barely visible (recent) dark green-grey with a dark spot medially at most anterior portion of abdomen, some pairs of dark spots and some w-shaped marks, none distinct, dark festoon present; ventrally (holotype) grey, no distinct markings (recent) dusky yellow; legs (holotype) orange (recent) light yellow-tan with some darker annulations, not distinct, femora with dark flecks on anterolateral faces, annulations more obvious on distal part of legs, particularly at the patellar-tibial joint on the tibiae. Carapace: 0.93 times longer than broad; fovea longitudinal, broad, very shallow. Eyes:AER slightly recurved; PER slightly recurved; PME same size as AME, PLE largest, ALE smallest; eye diameters, AME 0.20, ALE 0.08, PME 0.20, PLE 0.23; interdistances AME-PME 0.13, PME-ALE 0.25, ALE-PLE 0.18. PME-PME 1.20. ALE-ALE 2.00; ocular quadrangle AME-AME 0.43, PLE-PLE 2.05; clypeus 0.10 high. Mouthparts:chelicerae with stout setae medially and anteriorly; maxillae longer than broad, with tuft of conspicuous setae distally; labium distally rounded. Sternum:1.09 times longer than broad, posteriorly indented. Legs: leg formula 2314; scopulae present on distal end of all 4 tarsi; tarsi I-IV with strong claw tufts; pr claw per foot slightly toothed; spination: leg I, Fm pr 1–1–0, rl 1–1–0, d 1–1–1; Ti v 2–2–2; Mt v 2–2; Ti and Mt I and II with weak spines; II, Fm pr (L) 1–0–0, (R) 1–1–0, rl 1–1–0, d 1–1–1; Ti v 2–2–2; Mt v 2–2; III, Fm pr 1–0–0, d 1–1–1, rl 1–0–0; Ti v 1–1–0; Mt v 2–0; IV, Fm pr 1–1–0, d 1–1–1, rl 0–1–1; Ti v 1; Mt v 1. Abdomen:without terminal setal tufts. Pedipalp:Fm, spination d 0–1–4; cymbium oval in ventral view; conductor large, arising medially on slightly curved stalk, pointed laterally, attached at lateral margin and medially, forming a circular space where stalk arises; embolus long, curved, though crossing bulb diagonally rather than curving around cymbial margin, basally wide, tapering abruptly, very slender, arising at 5 o'clock, terminating at 1 o'clock; MA wider basally, tapering distally,curving into small distal hook, arising at 3 o'clock; RTA with two apophyses; ventral apophysis curving ventrally, slightly tapered,rounded distally; lateral apophysis curving dorsally, triangular in ventral view, quadrangular in lateral view, slightly bifurcated distally; tibial apophyses barely reaching cymbium in ventral view (Figs 161–162). *Dimensions:* Total length 7.90. Carapace length 3.73, width 4.03. Sternum length 1.75, width 1.60. Abdomen length 4.18, width 3.03. Pedipalp: Fm 1.00, Pt 0.25, Ti 0.30, Ta 0.75, total 2.30. Leg I: Fm 4.75, Pt 1.75, Ti 3.85, Mt 3.30, Ta 1.50, total 15.15. Leg II: Fm 5.80, Pt 1.75, Ti 4.90, Mt 4.00, Ta 1.75, total 18.20. Leg III: Fm 5.25, Pt 1.25, Ti 4.00, Mt 3.75, Ta 1.25, total 15.50. Leg IV: Fm 5.00, Pt 1.00, Ti 3.90, Mt 3.50, Ta 1.50, total 14.90.

*Female (sel_159)*: Color: carapace brown with some darker patches laterally, and some light colored setae; sternum dusky yellow, darker around border; chelicerae brown, darker laterally; maxillae brown, lightening distally; labium brown, lightening distally; abdomen dorsally tan with dark anteriorly, some chevrons caudally (difficult to see due to torn abdomen), dark flecks, festoon present; ventrally dusky grey with no markings; legs yellow to tan with dark annulations, annulations more prominent than in male, flecks on anterolateral face of femora as in male. Carapace: 0.91 times longer than broad; fovea longitudinal, broad, very shallow. Eyes: AER slightly recurved; PER slightly recurved; AME slightly larger than PME, AME largest, ALE smallest; eye diameters, AME 0.18, ALE 0.05, PME 0.15, PLE 0.15; interdistances AME-PME 0.15, PME-ALE 0.13, ALE-PLE 0.30. PME-PME 1.15. ALE-ALE 1.75; ocular quadrangle AME-AME 0.45, PLE-PLE 2.00; clypeus 0.13 high. Mouthparts: chelicerae with stout setae medially and anteriorly; maxillae longer than broad, with tuft of conspicuous setae distally; labium distally rounded. Sternum: 1.14 times longer than broad, posteriorly indented. Legs: leg I only slightly shorter than legs II, III and IV; leg formula 2341; scopulae present on tarsi of all legs and metatarsi of legs I and II; tarsi I-IV with strong claw tufts; pr claw per foot slightly toothed; spination: leg I, Fm pr 1–1–0, d 1–1–1, rl 0; Ti v 2–2–2; Mt v 2–2; leg II, Fm pr 0, d 1–1–1, rl 0; Ti v 2–2–2; Mt v 2–2; leg III, Fm pr 0, d 1–1–1, rl 0; Ti v 1–1–0; Mt v 1–0; leg IV, Fm pr 0, d 1–1–1, rl 0; Ti v 1; Mt v 1. Abdomen: without tufts of setae. Pedipalp: claw with ca. 6 teeth. Epigyne: Epigynal plate triangular, v-shaped opening medially, genital openings located behind this, lateral lobes fused medially, long epigynal pockets present; internally, short ducts lead posterolaterally to round amorphous spermathecae, posterodorsal fold absent (Figs 163–164). *Dimensions:* Total length 6.50. Carapace length 3.40, width 3.73. Abdomen length 3.10, width 2.80. Pedipalp: Fm 0.75, Pt 0.35, Ti 0.45, ta 0.75, total 2.30. Leg I: Fm 3.75, Pt 1.25, Ti 2.75, Mt 2.00, Ta 1.10, total 10.85. Leg II: Fm 4.50, Pt 1.00, Ti 4.00, Mt 2.75, Ta 1.00, total 13.25. Leg III: Fm 5.00, Pt 1.15, Ti 3.00, Mt 2.75, Ta 0.90, total 12.80. Leg IV: Fm 4.00, Pt 1.10, Ti 3.00, Mt 2.75, Ta 1.10, total 11.95.

#### Natural history.

This species is known only from high elevation cloud forests. It has been collected under bark and under rocks, as well as on stone walls and buildings at night.

#### Distribution.

This species appears to be endemic to the southern part of the island of Hispaniola (Map 13).

### Selenops morro sp. n.

urn:lsid:zoobank.org:act:36825C2F-968E-437C-BCDA-F6F72EABBF4C

http://species-id.net/wiki/Selenops_morro

Figs 165 168 206 Map 13

#### Type material.

Holotype female from El Morro, Monte Cristi, Dominican Republic, 19.89233°N, 71.65688°W, 8.X.2006, 20–40 m, S. Crews, under rocks, bark, SCC06_069, (EME sel_614). Paratypes: Male, El Morro, Monte Cristi, Dominican Republic, 19.89233°N, 71.65688°W, 22.VII.2006, 20–40 m, L. Mahler, under rock beside road, (EME sel_582).

#### Other material examined.

DOMINICAN REPUBLIC: Monti Cristi: same data as allotype, 1♀ (MNHNSD sel_580); same data as holotype, 2♀, 3 imm. (CAS sel_610, 615-616, 618).

#### Etymology.

The specific epithet comes from the name of the type locality. It is to be treated as a noun in apposition.

#### Diagnosis.

This small species is most similar to several endemic Cuban species, however females can be differentiated from all other spcies by a sinuous opening extending the width of the epigynal plate, and there appears to be epigynal pockets. These are separated and facing outward rather than facing one another as in other species (Figs 165–166). Males can be separated from other species by the presence of a unique conductor and embolus, both sinuous, the conductor arising behind another sclerite, extending beyond the edge of the bulb, and the embolus is s-shaped (Figs 167–168).

#### Description.

*Paratype male:* Color:carapace light yellowish, slightly darker laterally and medially; sternum pale yellow; chelicerae yellow, dusky markings anteromedially terminating halfway down, curving toward lateral margins, area below this medially is very pale; maxillae pale yellow; labium dusky yellow, lightening distally; abdomen dorsally pale yellow, with 2 curving dusky lines on each edge of anterior margin, lanceolate stripe narrow anteriorly, widening medially where edges of pattern go to lateral margins of abdomen, then return medially above posterior margin of abdomen, medial lanceolate stripe crossed by 3 chevrons and terminating in a small chevron, laterocaudal festoon barely visible; ventrally dusky yellow-grey, no markings; legs light yellow, darkening slightly distally, faint annulations, dusky spots anterolaterally on femora I. Carapace: 0.79 times longer than broad; fovea longitudinal, broad, very shallow. Eyes:AER nearly straight; PER recurved; PME larger than AME, PLE largest, ALE smallest; eye diameters, AME 0.175, ALE 0.05, PME 0.2, PLE 0.28; interdistances AME-PME 0.03, PME-ALE 0.08, ALE-PLE 0.28. PME-PME 0.80. ALE-ALE 1.38; ocular quadrangle AME-AME 0.28, PLE-PLE 1.45; clypeus 0.06 high. Mouthparts:chelicerae with a few stout setae medially and anteriorly; maxillae longer than broad, with tuft of conspicuous setae distally; labium distally rounded. Sternum:1.10 times longer than broad, posteriorly indented. Legs:leg I only slightly shorter than leg II; leg formula 2134; scopulae present on distal end of all 4 tarsi; tarsi I-IV with strong claw tufts; pr claw per foot slightly toothed; spination: leg I, Fm pr 1–1–1, d 1–1–1, rl 1–1–1; Ti d 1–1–0, pr 1–0–1, rl 1–0–1, v 2–2–2–2; Mt pr 1–1–0, v 2–2–0, rl 1–0–0; leg II, Fm pr 1–1–1, d 1–1–1, rl 1–1–1; Ti pr 1–0–1, d 1–1–0, rl 1–0–1, v 2–2–2–2; Mt pr 1–1–0, rl 1–0–0, v 2–2; leg III, Fm pr 1–1–1, d 1–1–1, rl 1–1–1; Ti pr 1–0–1, pr 1–0–1, d 1–1–0, v 2–2; Mt pr 1–1–0, rl 1–0–0, v 2–2; leg IV, Fm pr 1–1–1, d 1–1–1, rl 1–1–1; Ti pr 1–0–1, d 1–0–0, rl 1–0–1, v 2–1; Mt pr 1–1–0, rl 1–1–0, v 2–2. Abdomen:with terminal setal tufts. Pedipalp:Fm, spination d 0–1–4; cymbium oval in ventral view, slightly truncate posterolaterally, conductor arising medially on stout slightly sinuous stalk, anteriorly, conductor extends laterally, somewhat t-shaped, pointed, going beyond edge of cymbium, in other direction, conductor reconnects to bulb; embolus long, sinuous, s-shaped, beginning at 6 o'clock, curving laterally, then back, tapering distally, ending at 1 o'clock; MA narrow, long, curved distally into small single hook, arising at 3 o'clock, directed anterolaterally; RTA with two apophyses, lateral apophysis wide, quadrangular, distally truncate, the ventral apophysis short, narrow, distally rounded; RTA barely reaching cymbium in ventral view (Figs 167–168). *Dimensions:* Total length 4.65. Carapace length 2.55, width 3.23. Sternum length 1.65, width 1.50. Abdomen length 2.10, width 2.00. Pedipalp: Fm 1.00, Pt 0.25, Ti 0.35, Ta 0.75, total 2.35. Leg I: Fm 4.00, Pt 1.50, Ti 4.00, Mt 4.00, Ta 1.65, total 15.15. Leg II: Fm 4.00, Pt 1.25, Ti 4.00, Mt 4.25, Ta 1.75, total 15.25. Leg III: Fm 4.00, Pt 1.00, Ti 4.00, Mt 4.00, Ta 1.60, total 14.60. Leg IV: Fm 3.00, Pt 1.00, Ti 3.00, Mt 3.00, Ta 1.00, total 11.00.

*Holotype female:* Color:carapace dusky yellow, 2 dark smudges in cephalic area, dusky around border, and medially, clypeus darker and black around PLEs extending to edge of carapace; sternum pale yellow; chelicerae same dusky yellow as cephalothorax, with 2 medial stripes joining midway down and curving around, leaving a lighter area below; maxillae pale yellow, lighter distally; labium dusky yellow, lightening distally; abdomen dorsally pale yellow, with 2 curving dusky lines on each corner of anterior margin, lanceolate pattern, begins narrow, widens medially where edges of pattern go to edges of abdomen, then back to the center just above the posterior margin, medial lanceolate stripe crossed by 3 chevrons and terminating in a small chevron, laterocaudal festoon barely visible; ventrally cream colored; legs yellow with prominent annulations, on femora, rings don't always completely connect, but areas are particularly dark where spines emerge. Carapace: 0.95 times longer than broad; fovea longitudinal, broad, very shallow. Eyes:AER nearly straight; PER recurved; PME larger than AME, PLE largest, ALE smallest; eye diameters, AME 0.15, ALE 0.08, PME 0.28, PLE 0.30; interdistances AME-PME 0.03, PME-ALE 0.08, ALE-PLE 0.28. PME-PME 0.98. ALE-ALE 1.63; ocular quadrangle AME-AME 0.33, PLE-PLE 1.63; clypeus 0.08 high. Mouthparts:chelicerae with a few stout setae medially and anteriorly; maxillae longer than broad, with tuft of conspicuous setae distally; labium distally rounded. Sternum:1.07 times longer than broad, posteriorly indented. Legs:leg I only slightly shorter than leg 3; leg formula 3124; scopulae present on tarsi of all legs and metatarsi of legs I and II; tarsi I-IV with strong claw tufts; pr claw per foot slightly toothed; spination: leg I, Fm pr 1–1–1, d 1–1–1, rl 0–1–0; Ti d 0, v 2–2–2; Mt v 2–2; leg II, Fm pr 1–0–0, d 1–1–1, rl 0–1–0; Ti v 2–2–2; Mt v 2–2; leg III, Fm pr 1–0–0, d 1–1–1, rl 0–0–1; Ti v 2–2–0; Mt v 2–2; leg IV, Fm pr 1–0–0, d 1–1–1, rl 0; Ti v 1–2; Mt v 2–1. Abdomen:with terminal setal tufts. Pedipalp:claw with 7 teeth. Epigyne:sinuous margin medially extending entire width of plate, genital openings located behind this at lateral margins, epigynal pockets present, facing laterally rather than medially; internally, ducts small, thin, directed medially to laterally, touching medially, coiling laterally, fertilization ducts located and directed laterally, small, thin posterodorsal fold present covering internally ducts posteromedially (Figs 165–166). *Dimensions:* Total length 6.95. Carapace length 3.15, width 3.33. Sternum length 1.60, width 1.50. Abdomen length 3.80, width 3.30. Pedipalp: Fm 1.00, Pt 0.25, Ti 0.35, Ta 0.80, total 2.40. Leg I: Fm 2.90, Pt 1.00, Ti 3.40, Mt 1.75, Ta 0.85, total 9.90. Leg II: Fm 3.00, Pt 1.00, Ti 2.75, Mt 1.90, Ta 0.90, total 9.55. Leg III: Fm 3.00, Pt 1.00, Ti 3.50, Mt 2.00, Ta 0.90, total 10.40. Leg IV: Fm 3.00, Pt 0.85, Ti 2.50, Mt 2.00, Ta 1.00, total 9.35.

#### Natural history.

Found under rocks and bark in dry thornscrub (Fig. 206).

#### Distribution.

Endemic to Hispaniola and appears to be very narrowly distributed on the Monti Cristi Peninsula located in the central part of the North Coast of Hispaniola (Map 13).

### Selenops simius

Muma, 1953

http://species-id.net/wiki/Selenops_simius

**Figures 169–176.**
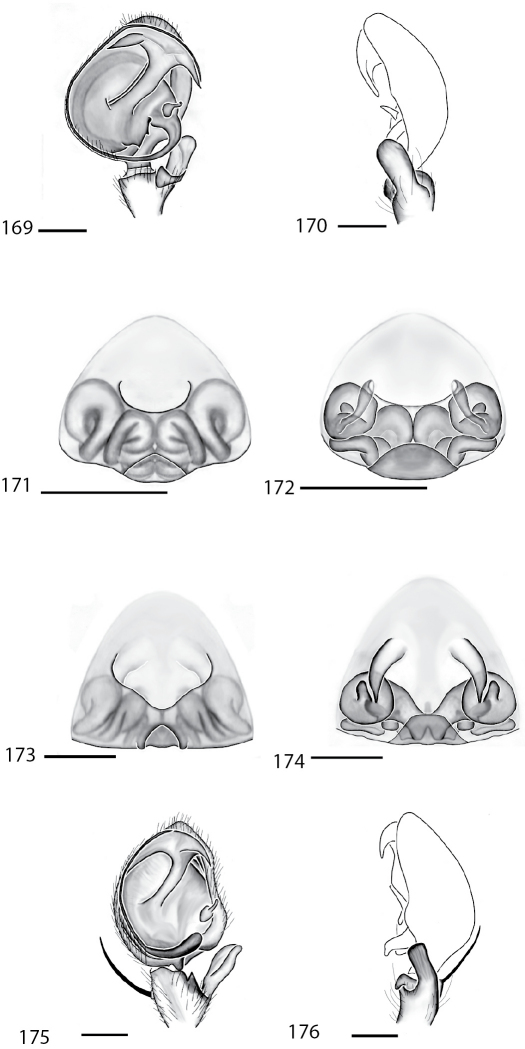
Copulatory organs of *Selenops simius* Muma male holotype from Bimini Island, Bahamas (AMNH) **169–170** female from Bimini Island, Bahamas (AMNH) **171–172** *Selenops submaculosus* Bryant female holotype from Sierra de Casas, Isla de Piños, Cuba (MCZ) **173–174** male from Soledad, Cuba (MCZ) **175–176,** **169, 175** left pedipalp, ventral view **170–176** left pedipalp, retrolateral view **171, 173** epigyne, ventral view **172, 174** spermathecae, dorsal view. Scale bar = 0.50 mm.

**Figures 177–182.**
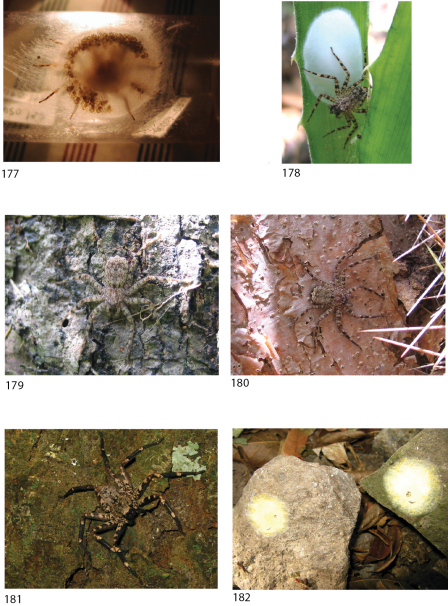
Habitats and natural history of some *Selenops* species. **177** *Selenops arikok* sp. n. from Aruba guarding egg sac with several spiderlings inside **178** *Selenops geraldinae* Corronca eating a fly and guarding her egg sac on a bromeliad, Gaspar Grande Island, Trinidad and Tobago **179** *Selenops willinki* Corronca on a tree trunk, Little Tobago, Trinidad and Tobago. Terminal setal tufts and festoon pattern are visible **180** *Selenops micropalpus* Muma on the trunk of *Bursera simaruba*, in a dry forest, St. Lucia. Terminal setal tufts and festoon pattern are visible **181** *Selenops mexicanus* Keyserling on a large tree trunk outside of Cueva Actun Kan, Guatemala. Note the alternating light and dark leg annulations **182** Egg sacs of *Selenops bifurcatus* Banks on rocks in a wash in dry forest and thornscrub, Zacatan, Guatemala. *Selenops bifurcatus* sometimes guards the yellowish egg sacs, and other times does not.

**Figures 183–188.**
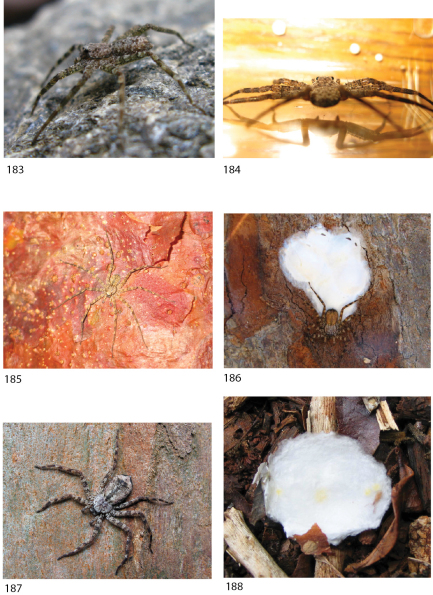
Habitats and natural history of some *Selenops* species **183** *Selenops bifurcatus* Banks on a rock, Zacatan, Guatemala. Note the dorsoventrally flattened habitus **184** *Selenops bifurcatus* Banks showing the very large PLE **185** *Selenops lindborgi* Petrunkevitch adult male on *Bursera simaruba* St. John, United States Virgin Islands. Note the leg annulations **186** *Selenops lindborgi* Petrunkevitch adult female making several egg sacs under the bark of a tamarind tree, Nevis. *Selenops lindborgi* makes several egg sacs and does not guard the egg sac, unlike most other species of *Selenops* **187** *Selenops petrunkevitchi* Alayón-García on a eucalyptus tree, Whitfield Hall, Blue Mountains, Jamaica. Note the striped legs and the festoon pattern at the terminus of the abdomen **188** The egg sac of *Selenops wilmotorum* sp. n. from Hellshire Hills, Jamaica. The egg sac contained a few yellow dried out eggs and a pseudoscorpion.

**Figures 189–194.**
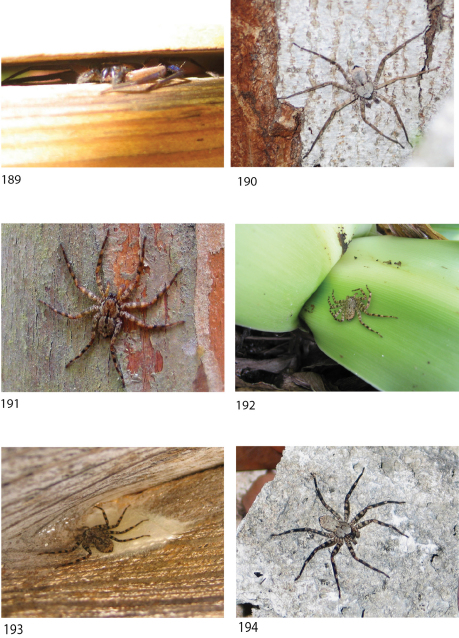
Habitats and natural history of some *Selenops* species. **189** *Selenops insularis* Keyserling, Laguna Kiani, Vieques, Puerto Rico **190** *Selenops baweka* sp. n. adult male on the trunk of a tamarind tree, Turtle Cove, Providenciales, Turks and Caicos Islands **191** *Selenops kalinago* sp. n. on tree trunk, Silver Hills, Montserrat. Note striped legs and how the legs darken distally **192** *Selenops souliga* sp. n., Back Bay, St. Maarten, between the leaves of a plant **193** *Selenops souliga* sp. n. adult female guarding her flat white egg sac inside of a folded palm frond, Anguilla **194** *Selenops simius* Muma, on a limestone rock at night, Cayman Brac, Cayman Islands.

Figs 169–172 194 Map 10

Selenops simius Muma 1953: 27, Figs 46–48 (♂, ♀, examined).
Selenops simius :Alayón-García 2005: 21, Figs 12–15 (♂, ♀).

#### Type material.

Holotype male: South Bimini Island, Bahamas, V.1951, W. Gertsch, M.A. Cazier (AMNH, examined). Paratypes: Female, same data as holotype (AMNH, examined).

#### Other material examined.

BAHAMAS: South Bimini: 2-9.VIII.1951, C.,P. Vaurie, 1♀, 1♂ (AMNH). **Rum Cay:** Port Nelson, 15.III.1953, Hayden, Rabb, Giovannoli, 1♂ (AMNH). **CAYMAN ISLANDS: Cayman Brac:** National Trust House off West End Road, mass grave site, 19°42.019'N, 79°52.084'W, ~2', 3.X.2004, S. Crews, on rocks, trees at night, SCC04_025, 1♀, 2♂, 3 imm. (EME sel_023-028). **Grand Cayman:** Queen Elizabeth II Botanic Park, vic. iguana pens, 19°19.042'N, 81°10.087'W, ~6 m, 2.X.2004, S. Crews, under bark, rocks, SCC04_022, 4 imm. (EME sel_046, 066-067, 080). **Little Cayman:** road across the street from Pirate's Point, 19°39.754'N, 80°06.032'W, 3.X.2004, S. Crews, under fallen palm frond at base of trunk, SCC04_023, 1♀ (CAS sel_022).

#### Diagnosis.

This species is similar to and can be difficult to separate from *Selenops submaculosus*. Males can be reliably separated by the following characters: the cymbium is very round, the conductor is larger and slightly curved upward at the distal point, the embolus extends beyond the cymbium at its point of origin, and the lateral branch of the RTA is slightly rounded distally in lateral view (Figs 169–170). Females can be separated from others by having a shorter, wider and more u-shaped genital opening, rather than a heart-shaped opening, the internal ducts are coiled more medially, and the ducts only come into contact with the posterior margin of the plate medially (Figs 171–172).

#### Remarks.

Muma (1953) reported the female to have the leg formula of 2314, however my measurements indicate 3142, once again attesting to the problems of using leg lengths to examine species relationships. This species is similar to its sister taxon, *Selenops submaculosus*, and can be difficult to distinguish. Muma (1953) distinguished males in the key, but not females. He mentioned that they were very similar, but that it was very easy to distinguish them based on structural differences, but did not suggest how to do so. Alayón-García suggested the two species can be distinguished by their size, with *Selenops simius* being smaller than *Selenops submaculosus*. After examining several specimens, is has been found that this does not always prove to be true. Yet, there are several genitalic characters that can be used to consistently distinguish these species. In the female, these include the genital openings, the shape and position of the internal ducts, and in the male, the shape of the cymbium, the conductor and the RTA.

#### Description.

*Holotype male:* Color: carapace orange-brown, darker medially, dark patches laterally; sternum yellow, darker around border; chelicerae light brown-orange, lighter laterally; labium light brown, lightening distally; abdomen dorsally (holotype) cream with darker grey lanceolate medial stripe and chevrons caudally, festoon present (recent) cream, with two spots in center of abdomen, festoon present; ventrally cream with no markings; legs yellowish, annulations not distinct. Cephalothorax: 0.87 times longer than broad; fovea longitudinal, broad, very shallow. Eyes:AER nearly straight; PER slightly recurved; PME larger than AME, PLE largest, ALE smallest; eye diameters, AME 0.13, ALE 0.06, PME 0.18, PLE 0.20; interdistances AME-PME 0.03, PME-ALE 0.10, ALE-PLE 0.20. PME-PME 0.73. ALE-ALE 1.13; ocular quadrangle AME-AME 0.23, PLE-PLE 1.30; clypeus 0.03 high. Mouthparts:chelicerae with a few stout setae medially and anteriorly; maxillae longer than broad, with tuft of conspicuous setae distally; labium distally rounded. Sternum:as long as broad, posteriorly indented. Legs:leg formula 2314; scopulae present on tarsi of all legs and metatarsi of legs I and II; tarsi I-IV with strong claw tufts; pr claw per foot slightly toothed; spination: leg I, Fm pr 1–1–1, d 1–1–1, rl 1–1–0; Ti d 1–1–0, pr 1–0–1, rl 1–0–1, v 2–2–2; Mt pr 1–1–0, rl 1–1–0, v 2–2; II, Fm pr 1–1–1, d 1–1–1, rl 1–1–1; Ti pr 1–0–1, d 1–1–0, rl 1–0–1, v 2–2–2; Mt pr 1–1–0, rl 1–0–0, v 2–2; III, Fm pr 1–1–1, d 1–1–1, rl 1–1–1; Ti pr 1–0–1, rl 1–0–1, d 1–1–0, v 2–2; Mt pr 1–1–0, rl 1–0–0, v 2–2; IV, Fm pr 1–1–1, d 1–1–1, rl 1–1–1; Ti pr 1–0–1, rl 1–0–1, d 1–0–0, v 2–2; Mt pr 1–1–0, rl 1–0–0, v 2–2–0. Abdomen:with terminal setal tufts. Pedipalp:Fm, spination d 0–1–4; cymbium round to teardrop shaped in ventral view, strongly dorsoventrally compressed; conductor large arising from medially from a nearly straight stalk, T-shaped, extending laterally beyond cymbium, pointed posteriorly at tip; embolus very long, curved, originating at 4 o'clock, terminating at 3 o'clock, extending beyond edge of cymbium at origin; MA arising at 3 o'clock, directed laterally, very small, tapering, slightly hooked distally; RTA with two apophyses; ventral process ¼ the size of lateral process, rounded, curving ventrally, lateral process large, rectangular, curving slightly ventrally, rounded distally in lateral view; tibial apophyses extending at least ¼ length of cymbium in ventral view (Figs 169–170). *Dimensions:* Total length 5.05. Carapace length 2.30, width 2.65. Sternum length 1.60, width 1.60. Abdomen length 2.75, width 1.95. Pedipalp: Fm 1.20, Pt 0.25, Ti 0.30, Ta 1.00, total 2.75. Leg I: Fm 4.75, Pt 1.75, Ti 5.00, Mt 4.75, Ta 2.00, total 18.25. Leg II: Fm 5.00, Pt 1.75, Ti 5.00, Mt 5.00, Ta 2.00, total 18.75. Leg III: Fm 5.50, Pt 1.75, Ti 5.10, Mt 4.80, Ta 2.00, total 19.15. Leg IV: Fm 4.75, Pt 1.40, Ti 4.60, Mt 4.70, Ta 1.85, total 17.30.

*Paratype female:* Color:carapace (paratype) orange-brown, white setae, no noticeable markings (recent) orange-brown, but slightly darker laterally; sternum dusky yellow, darker around border; chelicerae brown, darker laterally, lighter medially; labium light brown lightening distally; abdomen dorsally (paratype) cream to tan with dark spots medially and laterally, in pairs medially, one w-shaped mark caudally, with a slightly upward curving line and a single dot beneath, festoon prominent (recent) two most-prominent dots in center of abdomen, not at the top; ventrally cream; legs yellow-brown with faint darker brown annulations; Carapace: 0.9 times longer than broad; fovea longitudinal, broad, very shallow. Eyes:AER slightly recurved; PER recurved; PME larger than AME, PME largest, ALE smallest; eye diameters, AME 0.20, ALE 0.05, PME 0.28, PLE 0.23; interdistances AME-PME 0.05, PME-ALE 0.10, ALE-PLE 0.35. PME-PME 0.98. ALE-ALE 1.58; ocular quadrangle AME-AME 0.38, PLE-PLE 1.80; clypeus 0.10 high. Mouthparts:chelicerae with a few stout setae medially and anteriorly; maxillae longer than broad, with tuft of conspicuous setae distally; labium distally rounded. Sternum: as long as broad, posteriorly indented. Legs:leg I only slightly shorter than II and III; leg formula 3142; scopulae present on all 4 tarsi and tibia and metatarsus of leg I; tarsi I-IV with strong claw tufts; pr claw per foot slightly toothed; spination: leg I, Fm pr 1–1–0, d 1–1–1, rl 1–0–1; Ti v 2–2–2; Mt v 2–2; leg II, Fm pr 1–0–0, d 1–1–1, rl 1–0–1; Ti v 2–2–2; Mt v 2–2; leg III, Fm pr 1–0–0, d 1–1–1, rl 1–0–1; Ti v 2–2–0; Mt v 2–2; leg IV, Fm pr 0–0–1, d 1–1–1, rl 0; Ti v 2–2–0; Mt v 2–1. Abdomen:with terminal setal tufts. Pedipalp:claw with 9 teeth. Epigyne:u-shaped medial depression, genital openings located at lateral margins, plate sinuous along posterior margin, epigynal pockets present; internally, small ducts lead to large coiled ducts, posterodorsal fold present medially, covering ducts posteromedially, rounded (Figs 171–172). *Dimensions:* Total length 7.48. Carapace length 3.23, width 3.60. Sternum length 1.60, width 1.60. Abdomen length 4.25, width 3.43. Pedipalp: Fm 0.80, Pt 0.25, Ti 0.40, Ta 0.80, total 2.25. Leg I: Fm 3.00, Pt 1.50, Ti 3.10, Mt 2.75, Ta 1.00, total 11.35. Leg II: Fm 3.10, Pt 1.00, Ti 2.80, Mt 2.00, ta 1.00, total 9.90. Leg III: Fm 4.00, Pt 1.00, Ti 3.00, Mt 2.75, Ta 1.10, total 11.85. Leg IV: Fm 3.25, Pt 1.20, Ti 2.75, Mt 2.50, Ta 1.00, total 10.70.

#### Natural history.

This species has been taken under rocks and bricks in dry tropical forest, under bark, within epiphytes, in houses, on rocks and trees at night (Fig. 194), and under fallen palm fronds. The female guards a white, disc-shaped egg sac. One specimen was observed eating a roach at night.

#### Distribution.

Known from mainland Cuba and the surrounding islands and cays, all of the Cayman Islands, and South Bimini and Rum Cay in the Bahamas (Map 10).

### Selenops submaculosus

Bryant, 1940

http://species-id.net/wiki/Selenops_submaculosus

Figs 173–176 195 Map 10

Selenops submaculosus Bryant 1940: 406, pl. 13, Figs 177, 184 (♂, ♀, examined).
Selenops submaculosus :Muma 1953: 26, Figs 43–45 (♂, ♀).
Selenops submaculosus :Alayón-García 2005: 16, Figs 7–10 (♂, ♀).

#### Type material.

Holotype female: Sierra de Casas, Isla de Piños, Cuba, 1915, Barbour, Brooks (MCZ, examined). Paratypes:Male from Soledad, Cuba, II.1925, G. Salt (MCZ).

#### Other material examined.

BAHAMAS: Andros Island: International Field Station, North Blanket Sound, 24°53'51.1"N, 77°55'50.1"W, sea level to 4 m, 12.V.2006, S. Crews, R. Gibson, under bark of *Casuarina*, *Cocoloba diversifolia*, and *Bursera*, SCC06_001, 8♀, 1p♂, 7 imm. (EME sel_286-301);International Field Station, VII.2006, G.B. Edwards, 7♀, 3♂, 4 imm. (CAS sel_556-558, 595-596, 667-669, 671-673, 835-836); Morgan's Bluff Cave, VI.2006, G.B. Edwards, 1♀ (EME sel_666); North Andros, Morgan's Cave at Morgan's Bluff, 25°10'30.1"N, 78°01'26.2"W, ~15 m, 13.V.2006, S. Crews, R. Gibson, under bark and in cave, SCC06_003, 1♀, 1♂, 1 imm. (EME sel_309-311); Owenstown, 24°52'30.1"N, 78°02'03.6"W, ~11 m, 13.V.2006, S. Crews, R. Gibson, under bark of poison wood, gumbay, in coppice, SCC06_002, 1♀, 1♂, 3p♂, 2 imm. (CAS sel_302-308); Pigeon Cay: near Andros Island, 24°52'54.4"N, 77°53'35.5"W, ~15 m, 13.V.2006, S. Crews, under bark of *Casuarina*, SCC06_004, 1♀ (CAS sel_312). **North Bimini:** VII.1947, C.M. Breder, 1♂ (AMNH); Lerner Marine Labs, II-III.1948, E. Breder, 1♂ (AMNH). **South Bimini:** 12.VI.1950, M. Cazier, 1♀ (AMNH); VI.1951, M. Cazier, 1♀ (AMNH); 2-9.VII.1951, C. P. Vaurie, 1♂ (AMNH). **Great Exuma:** near old airport (Bahama Sound subdivision), 23°27'56.0"N, 75°46'24.8"W, ~4 m, 18.V.2006, S. Crews, N. Bottomley, under loose bark, SCC06_011, 1♀ (EME sel_332). Mayaguana **Island:** Abraham Bay, 2.III.1953, Hayden, Rabb, Giovannoli, 1♀ (AMNH). **CAYMAN ISLANDS: Grand Cayman:** Georgetown, ceiling of Beverly Anderson's house, 2.VII.1976, B. Anderson, 1♂ (AMNH). **CUBA: Cabo Cruz:** 1913, Cuban Expedition, 1♀ (MCZ). **Ceiba:** 2.VII.1955, A. Archer, 1♂ (AMNH). **Havana:** 1♀ (USNM). **Santiago de Cuba:** 10.XII.1055, 1♀ (AMNH). **Pinar del Rio:** Sierra de Mesa, 20.I.2006, 1 imm. (EME sel_276). **Soledad:** 1.VIII.1935, B.B. Leavitt, 1♀ (MCZ); Soledad Mountains, 10.III.1925, G. Salt, 1♀ (MCZ). **UNITED STATES:** 1♀ (MCZ). **Florida:** Broward Co., Hollywood, 3197 Taft, XII.2004, S. Diefenbacher, 1♂ (FSU); Lee Co., Fort Myers, 1.V.2003, R. Flowers, 1♀ (FSU); Palm Beach Co., West Palm Beach, Jog Road, hammock, 26.III.2004, D. Benner, 1♂ (FSU); Palm Beach Co., Palm Beach, 7.IX.2001, T. Vitum, in house (kitchen), 1♀ (FSU).

#### Diagnosis.

This species is similar to *Selenops simius* but females can be differentiated by the median field which is more v-shaped to heart-shaped rather than u-shaped, the internal ducts are not as coiled, the sperm ducts are longer and wider, and the posterodorsal fold is more quadrangular, rather than rounded (Figs 173–174). Males can be separated from others by the cymbium which is not as round as in *Selenops simius*, the embolus which does not extend beyond the edge of the cymbium, the MA is larger and directed more ventrally, and the lateral apophysis of the RTA is truncate distally (Figs 175–176).

#### Description.

*Paratype male:* Color:carapace (paratype) brown (recent) yellow-brown, darker at lateral margins, dark at fovea; sternum (paratype) orange-brown, darker around border (recent) light yellow, darker around border; chelicerae (paratype) brown, darker laterally, (recent) dusky yellow, dark grey laterally; maxillae (paratype) yellow-brown (recent) light yellow; labium (paratype) dusky yellow-brown, darker laterally, lightening distally (recent) pale yellow, lightening distally; abdomen dorsally (paratype) light yellow-brown, remnants of medial stripe and caudal w-shaped marks slightly visible, festoon visible (recent) grey-brown, darker laterally, markings similar to female, though less distinct; ventrally (paratype) yellow (recent) light yellow; legs (paratype) orange-brown, markings not visible (recent) lighter than carapace, yellow to cream with dusky annulations slightly visible, anterolateral faces of femora with dusky spots making a stripe. Carapace:0.89 times longer than broad; fovea longitudinal, broad, very shallow. Eyes:AER nearly straight; PER slightly recurved; PME larger than AME, PLE largest, ALE smallest; eye diameters, AME 0.20, ALE 0.06, PME 0.28, PLE 0.38; interdistances AME-PME 0.18, PME-ALE 0.18, ALE-PLE 0.40. PME-PME 1.20. ALE-ALE 1.90; ocular quadrangle AME-AME 0.38, PLE-PLE 2.15; clypeus 0.07 high. Mouthparts:chelicerae with few scattered setae medially and anteriorly; maxillae longer than broad, with tuft of conspicuous setae distally; labium distally rounded. Sternum:as long as broad, posteriorly indented. Legs:leg I only slightly shorter than II and III; leg formula 2314; scopulae present on tarsi of all legs and metatarsi of legs I and II; tarsi I-IV with strong claw tufts; pr claw per foot slightly toothed; spination: leg I, Fm pr 1–1–1, d 1–1–1, rl 1–1–1; Ti d 1–1–0, pr 1–0–1, rl 1–0–1, v 2–2–2–1; Mt pr 1–1–0, rl 1–1–0, v 2–2; leg II, Fm pr 1–1–1, d 1–1–1, rl 1–1–1; Ti pr 1–0–1, d 1–1–0, rl 1–0–1, v 2–2–2; Mt pr 1–1–0, rl 1–0–0, v 2–2; leg III, Fm pr 1–1–1, d 1–1–1, rl 1–1–1; Ti pr 1–0–1, rl 1–0–1, d 1–0–0, v 2–2; Mt pr 1–1–0, rl 1–1–0, v 2–2; leg IV, Fm pr 1–1–1, d 1–1–1, rl 0–1–1; Ti pr 1–0–1, rl 1–0–1, d 1–0–0, v 2–2; Mt pr 1–1–0, rl 1–1–0, v 2–2. Abdomen:without terminal setal tufts. Pedipalp:Fm, spination d 0–1–4; cymbium oval to round in ventral view, slightly truncate posterolaterally; conductor large, arising medially on narrow stalk, T-shaped, pointed laterally; embolus very long, curved, originating at 5 o'clock, ending at 12 o' clock; MA arising at 3 o'clock, directed anterolaterally, small, abruptly tapered, slightly curved; quadrangular, truncate distally; RTA extending 1/5th length of cymbium in ventral view (Figs 175–176). *Dimensions:* Total length 7.35. Carapace length 3.85, width 4.33. Sternum length 1.85, width 1.85. Abdomen length 4.50, width 3.60. Pedipalp: Fm 1.00, Pt 0.25, Ti 0.50, Ta 1.50, total 3.25. Leg I: Fm 5.00, Pt 1.75, Ti 5.00, Mt 5.00, Ta 2.00, total 18.75. Leg II: Fm 6.25, Pt 1.90, Ti 6.00, Mt 5.00, Ta 2.00, total 21.15. Leg III: Fm 6.00, Pt 1.75, Ti 5.00, Mt 5.25, Ta 1.75, total 19.75. Leg IV: Fm 5.75, Pt 1.50, Ti 5.00, Mt 5.00, Ta 1.75, total 18.50.

*Holotype female:* Color:carapace (holotype) red-brown with white setae (recent) yellow-brown to red-brown, sometimes darker in cephalic area, dusky markings laterally and mediolaterally; sternum (holotype) orange-brown, darker around border, (recent) light yellow darker around border; chelicerae (holotype) dark red-brown, darker laterally (recent) same as carapace, darker laterally; maxillae (holotype) orange-brown, lightening distally (recent) dusky yellow, lightening distally; labium (holotype) dark brown, (recent) yellow-brown; abdomen dorsally (holotype) yellow-grey, duskier medially, no markings apparent except laterocaudal festoon (recent) grey-yellow-brown, darker caudally, lanceolate stripe, center of stripe sometimes lighter, thus can appear as two stripes, and sometimes just dots, two dots readily apparent in most specimens, caudally where abdomen is darker, brown w-shaped stripes and festoon apparent; ventrally (holotype) yellow (recent) light yellow; legs (holotype) orange-brown, annulations faded, but visible (recent) lighter than carapace, annulations present; Cephalothorax:0.88 times longer than broad; fovea longitudinal, broad, very shallow. Eyes:AER nearly straight; PER slightly recurved; AME slightly larger than PME, PLE largest, ALE smallest; eye diameters, AME 0.30, ALE 0.18, PME 0.28, PLE 0.45; interdistances AME-PME 0.10, PME-ALE 0.15, ALE-PLE 0.45. PME-PME 1.40. ALE-ALE 2.30; ocular quadrangle AME-AME 0.55, PLE-PLE 2.95; clypeus 0.15 high. Mouthparts:chelicerae with a few stout setae medially and anteriorly; maxillae longer than broad, with tuft of conspicuous setae distally; labium distally rounded. Sternum:as long as broad. Legs:leg I only slightly shorter than legs II, III and IV; leg formula 2134; scopulae present on tarsi of all legs and metatarsi of legs I and II; tarsi I-IV with strong claw tufts; pr claw per foot slightly toothed; spination: leg I, Fm pr 1–1–0, d 1–1–1, rl 0–0–1; Ti v 2–2–2; Mt v 2–2; leg II, Fm pr 1–1–0, d 1–1–1, rl 0–0–1; Ti v 2–2–2; Mt v 2–2; leg III, Fm pr 1–1–0, d 1–1–1, rl 0–0–1; Ti v 2–2–0; Mt v 2–2; leg IV, Fm pr 1–1–0, d 1–1–1, rl 0–0–1; Ti v 2–2–0; Mt v 2–1. Abdomen:with terminal setal tufts. Pedipalp:claw with 8 teeth. Epigyne:median field v-shaped to heart-shaped, genital openings located at lateral margins, epigynal pockets present; internally, sperm ducts extend posteriorly, tapering to rounded ducts, posterodorsal fold present medially, squarish to angular, truncate anteriorly (Figs 173–174). *Dimensions:* Total length 11.13. Carapace length 4.50, width 5.10. Abdomen length 6.63, width 4.95. Pedipalp: Fm 1.75, Pt 0.40, Ti 0.80, Ta 1.40, total 4.35. Leg I: Fm 5.80, Pt 2.00, Ti 4.75, Mt 3.30, Ta 1.00, total 16.85. Leg II: Fm 4.75, Pt 2.00, Ti 4.75, Mt 3.75, Ta 1.65, total 16.90. Leg III: Fm 5.00, Pt 1.75, Ti 4.00, Mt 3.50, Ta 1.50, total 15.75. Leg IV: Fm 5.00, Pt 1.75, Ti 3.75, Mt 3.75, Ta 1.30, total 15.55.

#### Natural History.

**Figures 195–200.**
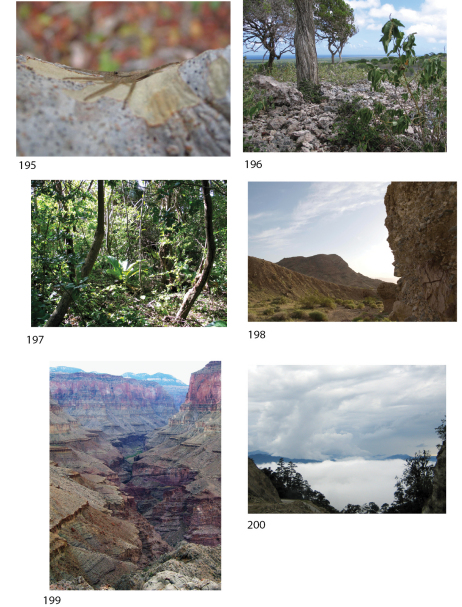
Habitats and natural history of some *Selenops* species. **195** *Selenops submaculosus* Bryant on a tree branch, San Salvador, Bahamas. Note extreme dorsoventral flattening **196** In this thornscrub habitat in Bonaire, *Selenops curazao* Alayón-García can be found under bark and under the limestone rocks on the ground **197** Dry forest habitat on Little Tobago where *Selenops willinki* Corronca is quite common under bark **198** A desert habitat in San Bernardino County California where members of the *Selenops debilis* group Muma can be found on and under boulders **199** Tapeats Canyon, Grand Canyon National Park. *Selenops nesophilus* Chamberlin has been collected at the bottom of the canyon and this is the northernmost collection locality for the genus in North America **200** Cloud forest in Chiapas, México where *Selenops huetocatl* sp. n. can be found.

This species has been found under bark and rocks, on the trunks of trees at night and on the ceiling at the entrance to a cave, as well as inside houses (Fig. 195). One female (sel_673) made an egg sac that contained about 30 eggs, but the eggs were yellow, deflated and gooey. Another female (sel_669) made an egg sac in VII. 2006. Some eggs hatched 13.VIII.2006. There were 42 eggs, but only 2 imms. One female (sel_287) was collected with a palp stuck in the epigynum. The female guards the white disc-shaped egg sac.

#### Distribution.

This species is found in Cuba, the Bahamas, Cayman Islands and southern Florida (Map 10).

## Supplementary Material

XML Treatment for Selenopidae

XML Treatment for Selenops

XML Treatment for Selenops arikok

XML Treatment for Selenops curazao

XML Treatment for Selenops geraldinae

XML Treatment for Selenops willinki

XML Treatment for Selenops banksi

XML Treatment for Selenops micropalpus

XML Treatment for Selenops aztecus

XML Treatment for Selenops gracilis

XML Treatment for Selenops malinalxochitl

XML Treatment for Selenops mexicanus

XML Treatment for Selenops abyssus

XML Treatment for Selenops juxtlahuaca

XML Treatment for Selenops marginalis

XML Treatment for Selenops minutus

XML Treatment for Selenops morosus

XML Treatment for Selenops nigromaculatus

XML Treatment for Selenops makimaki

XML Treatment for Selenops scitus

XML Treatment for Selenops ixchel

XML Treatment for Selenops actophilus

XML Treatment for Selenops debilis

XML Treatment for Selenops nesophilus

XML Treatment for Selenops lepidus

XML Treatment for Selenops chamela

XML Treatment for Selenops petenajtoy

XML Treatment for Selenops huetocatl

XML Treatment for Selenops bifurcatus

XML Treatment for Selenops buscki

XML Treatment for Selenops oricuajo

XML Treatment for Selenops amona

XML Treatment for Selenops lindborgi

XML Treatment for Selenops aissus

XML Treatment for Selenops candidus

XML Treatment for Selenops petrunkevitchi

XML Treatment for Selenops wilmotorum

XML Treatment for Selenops wilsoni

XML Treatment for Selenops beynai

XML Treatment for Selenops insularis

XML Treatment for Selenops insularis

XML Treatment for Selenops marcanoi

XML Treatment for Selenops bocacanadensis

XML Treatment for Selenops oviedo

XML Treatment for Selenops trifidus

XML Treatment for Selenops duan

XML Treatment for Selenops denia

XML Treatment for Selenops guerrero

XML Treatment for Selenops baweka

XML Treatment for Selenops kalinago

XML Treatment for Selenops souliga

XML Treatment for Selenops bani

XML Treatment for Selenops pensilis

XML Treatment for Selenops enriquillo

XML Treatment for Selenops phaselus

XML Treatment for Selenops morro

XML Treatment for Selenops simius

XML Treatment for Selenops submaculosus

## Figures and Tables

**Figures 201–206.**
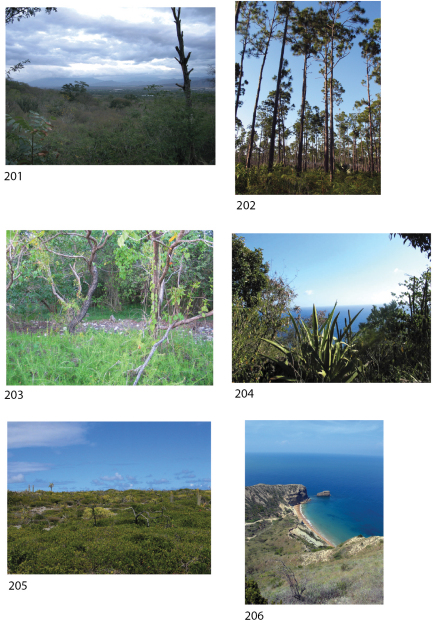
Habitats and natural history of some *Selenops* species. **201** Dry thornscrub and rocky wash where *Selenops bifurcatus* Banks was extremely common, Zacatan, Guatemala **202** Pine/fern habitat, Abaco, Bahamas where *Selenops aissus* Walckenaer can be found **203** Dry forest habitat around Laguna Oviedo, Pedernales, Dominican Republic. The species *Selenops oviedo* sp. n. can be found underneath the rocks **204** Thornscrub habitat, Shirley's Heights Lookout, Antigua, where *Selenops kalinago* sp. n. can be found under bark **205** Blowing Point, Anguilla, where *Selenops souliga* sp. n. can be found under limestone rocks in the thornscrub habitat **206** Coastal thornscrub habitat, El Morro, Dominican Republic. *Selenops insularis* Keyserling can be found under the bark of trees, while *Selenops morro* sp. n. is more common under rocks.
